# Highly Selective C(sp^3^)–H Bond Oxygenation at Remote Methylenic Sites Enabled by Polarity Enhancement

**DOI:** 10.1021/jacs.3c07658

**Published:** 2023-09-26

**Authors:** Sergio Sisti, Marco Galeotti, Filippo Scarchilli, Michela Salamone, Miquel Costas, Massimo Bietti

**Affiliations:** † Dipartimento di Scienze e Tecnologie Chimiche, Università “Tor Vergata”, Via della Ricerca Scientifica, 1, I-00133 Rome, Italy; ‡ QBIS Research Group, Institut de Química Computacional i Catàlisi (IQCC) and Departament de Química, Universitat de Girona, Campus Montilivi, Girona E-17071, Catalonia, Spain

## Abstract

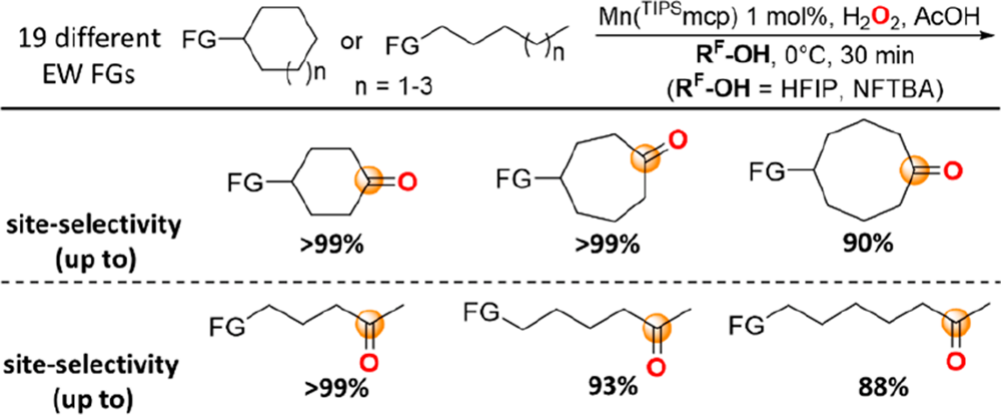

A detailed study on the C(sp^3^)–H bond oxygenation reactions with H_2_O_2_ catalyzed by the [Mn(OTf)_2_(^TIPS^mcp)] complex at methylenic sites of cycloalkyl and 1-alkyl substrates bearing 19 different electron-withdrawing functional groups (EW FGs) was carried out. Oxidations in MeCN were compared to the corresponding ones in the strong hydrogen bond donating (HBD) solvents 1,1,1,3,3,3-hexafluoro-2-propanol (HFIP) and nonafluoro *tert*-butyl alcohol (NFTBA). Formation of the products deriving from oxygenation at the most remote methylenic sites was observed, with yields, product ratios (PR) for oxygenation at the most remote over the next methylenic sites, and associated site-selectivities that significantly increased going from MeCN to HFIP and NFTBA. Unprecedented site-selectivities were obtained in the oxidation of cyclohexyl, cycloheptyl, cyclooctyl, 1-pentyl, 1-hexyl, and 1-heptyl substrates, approaching >99%, >99%, 90%, >99%, 93%, and 88% (PR >99, >99, 9.4, >99, 14, and 7.5) with cyclohexyl-2-pyridinecarboxylate, cycloheptyl-2-pyridinecarboxylate, cyclooctyl-4-nitrobenzenesulfonamide, 1-pentyl-3,5-dinitrobenzoate, 1-hexyl-3,5-dinitrobenzoate, and 1-heptyl-3,5-dinitrobenzoate, respectively. The results are rationalized on the basis of a *polarity enhancement* effect via synergistic electronic deactivation of proximal methylenic sites imparted by the EWG coupled to solvent HB. Compared to previous procedures, *polarity enhancement* provides the opportunity to tune site-selectivity among multiple methylenes in different substrate classes, extending the strong electronic deactivation determined by native EWGs by two carbon atoms. This study uncovers a simple procedure for predictable, high-yielding, and highly site-selective oxidation at remote methylenes of cycloalkyl and 1-alkyl substrates that occurs under mild conditions, with a large substrate scope, providing an extremely powerful tool to be implemented in synthetically useful procedures.

## Introduction

**Scheme 1:**
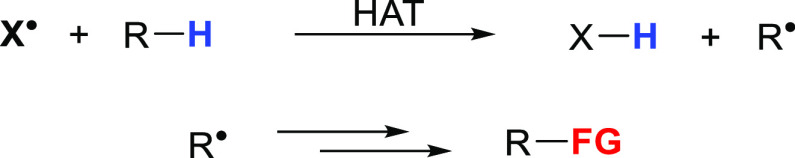
HAT-Based C(sp^3^)–H Bond Functionalization

Procedures for selective functionalization of nonactivated C(sp^3^)–H bonds represent one of the most investigated approaches to develop new synthetic methodologies.^1^ Among the methods employed for this purpose, those based on hydrogen atom transfer (HAT) to radical or radical-like reagents have attracted considerable interest in view of the possibilities they offer to introduce a large variety of functional groups in place of hydrogen under mild conditions.^2^ The reaction is initiated by HAT from a substrate C–H bond to give a carbon radical, which can be then converted into the functionalized product through different radical capture steps (Scheme 1).^3^

Because of the multitude of C(sp^3^)–H bonds typically displayed by organic molecules, the development of procedures for site-selective functionalization of both alkyl and cycloalkyl structural motifs is of great importance, as it can provide straightforward access to functionalized analogues without resorting to lengthy *de novo* syntheses. The latter substrate group appears moreover to be of particular interest in view of the emerging trend for marketed drugs and agrochemicals where isosteric replacement of aryl rings by saturated carbocycles has been employed to improve potency and solubility.^4,5^

**Scheme 2:**
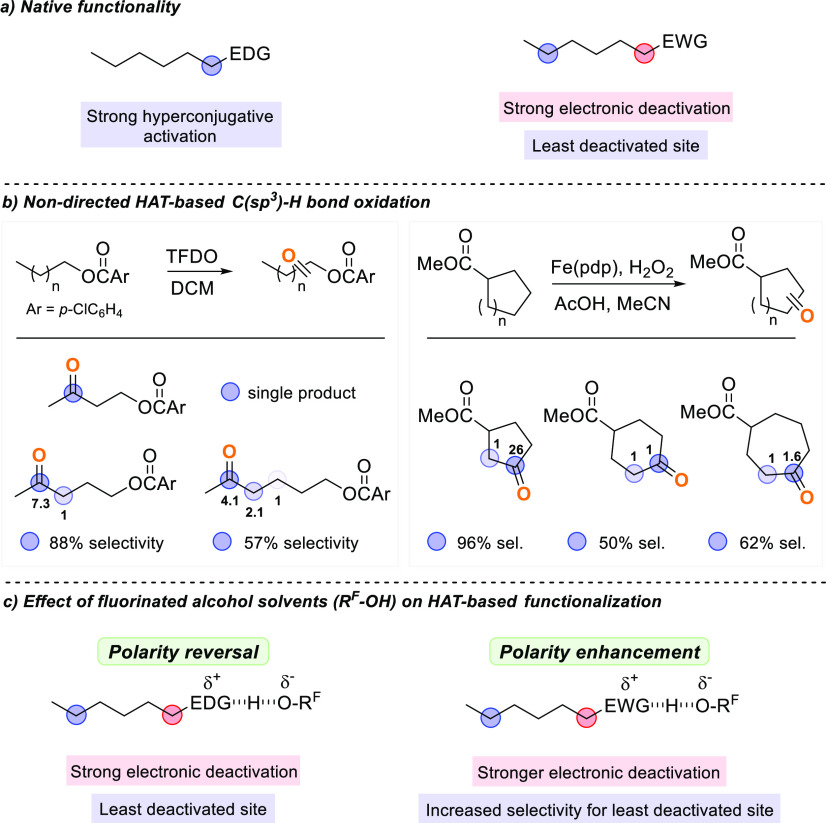
Electronic Effects on HAT-Based C(sp^3^)–H Bond Functionalization Promoted by Electrophilic Reagents: (a) Effect of Native Functionalities; (b) Site-Selective Functionalization at Remote Methylenic Sites; (c) Exploiting Solvent Hydrogen Bonding by Fluorinated Alcohols (R^F^OH) to Govern Site-Selectivity

The factors that govern site-selectivity have been discussed in detail and include bond strengths and electronic, steric, stereoelectronic, hyperconjugative, and torsional effects.^1a,2,3^ In favorable cases, these factors have been shown to synergistically cooperate to promote highly selective functionalizations. The electronic effects of native functionalities are often exploited for this purpose. Since the vast majority of HAT reagents display an electrophilic character, the functional group electronic features can play an important role, with reaction that preferentially occurs from electron-rich C(sp^3^)–H bonds compared to electron-poor ones of similar strength (Scheme 2a).^1a,3^ Along these lines, with substrates bearing electron-donating groups (EDGs) such as amines, amides, alcohols, and ethers, functionalization predominantly occurs at the most electron-rich α-C–H bonds, which are activated toward HAT by hyperconjugative overlap between the heteroatom lone pair and the C–H σ* orbital (polarity matching). On the other hand, the presence of electron-withdrawing groups (EWGs) deactivates the electron-poor α-C–H bonds toward HAT (polarity mismatching). The extent of this deactivation depends on the EW ability and rapidly decreases with increasing distance from the functional group, allowing, only in favorable cases, highly selective functionalization at the most remote and least electronically deactivated site (Scheme 2b). With substrates bearing tertiary C–H bonds that are four or five carbons away from the EWG, high levels of selectivity for functionalization at this site are customarily achieved, a behavior that reflects electronic deactivation coupled to the intrinsically stronger nature of primary and secondary C–H bonds that prevents or limits competitive functionalization at these sites.^3^ Accordingly, with substrates bearing multiple methylene groups, characterized by similar structural features and steric accessibility, highly selective functionalization at the most remote site was generally achieved only with cyclopentyl and 1-butyl derivatives, with the different methylene units that are no longer discriminated when moving further away from the EWG, and with the 1-alkyl derivatives, the most remote and least electronically deactivated primary C–H bonds that generally do not compete to a significant extent because of their high bond dissociation energy.^3^ For example, in the oxygenation of 1-alkyl 4-chlorobenzoates promoted by methyl(trifluoromethyl)dioxirane (TFDO),^6^ exclusive ketonization at the most remote C-3 methylene was observed with the 1-butyl derivative, whereas with the 1-pentyl and 1-hexyl ones, ketoesters deriving from oxidation at C-3 and C-4 in a 1:7.3 ratio and at C-3, C-4, and C-5 in a 1:2.1:4.1 ratio, respectively, were observed (Scheme 2b). Analogously, in the oxygenation of methylcycloalkanecarboxylates with H_2_O_2_ catalyzed by the Fe(pdp) complex,^7^ ketoesters deriving from C–H bond oxidation at C-2 and C-3 in a 1:26 ratio, and at C-3 and C-4 in a (statistically corrected) 1:1 and 1:1.6 ratio, were observed for methylcyclopentanecarboxylate, methylcyclohexanecarboxylate, and methylcycloheptanecarboxylate, respectively. Taken together, these results clearly evidence the current limits associated with the undirected site-selective functionalization of methylenic sites that are more than three carbons away from an EW functional group.

The electron density of C(sp^3^)–H bonds can be also modified by means of medium effects (i.e., taking advantage of hydrogen bonding or acid–base interactions promoted by solvents and/or additives), providing a simple and extremely powerful tool to alter reactivity and site-selectivity in HAT-based functionalization (Scheme 2c).^8^ For example, protonation of an amine or hydrogen bonding to an amine, amide, alcohol, or ether functional group by a strong hydrogen bond donor (HBD) solvent such as a fluorinated alcohol (R^F^OH) has been shown to determine a *polarity reversal*, leading to α-C–H bond deactivation toward electrophilic HAT reagents and consequent functionalization at the most remote and least electronically deactivated site. Initially employed to govern site-selectivity in remote C(sp^3^)–H bond functionalization of aliphatic amines by dioxiranes,^9^ and in HAT from the same substrate class to alkoxyl radicals,^10^ such *polarity reversal* strategies are now customarily employed in synthetically useful procedures.^11^ In contrast, an enhancement in α-C–H bond activation can be achieved following deprotonation or hydrogen bonding to a hydrogen bond acceptor (HBA) solvent or additive of an acidic functional group.^8,12^

Solvent hydrogen bonding can also be employed to increase the electronic deactivation determined by an EWG. Because of their HBA ability, hydrogen bonding to these groups by R^F^OH determines a stronger α-C–H bond deactivation and, as a consequence, increased selectivity for the most remote site through *polarity enhancement* (Scheme 2c). Although this effect is mostly unrecognized, its potential to improve selectivity for remote functionalization has emerged in three recent studies on HAT-based C(sp^3^)-H sulfination^13^ and oxygenation.^14,15^ Analysis of these studies evidences however some main limitations, associated in the former case to a very narrow substrate scope and in the latter ones to the use of dichloroacetic acid as the HBD solvent^14^ and the exclusive functionalization at remote and intrinsically more activated tertiary C(sp^3^)–H bonds.^15^

With these concepts in hand, and in order to demonstrate and uncover the full potential of this *polarity enhancement* effect, herein we report on the results of a detailed study on the C(*s*p^3^)–H bond oxygenation reactions with H_2_O_2_ catalyzed by manganese complexes, targeting methylenic sites of cycloheptyl and 1-hexyl derivatives bearing a variety of EW functional groups (FGs), the structures for which are displayed in Scheme 4 and Scheme 5.

**Scheme 3:**
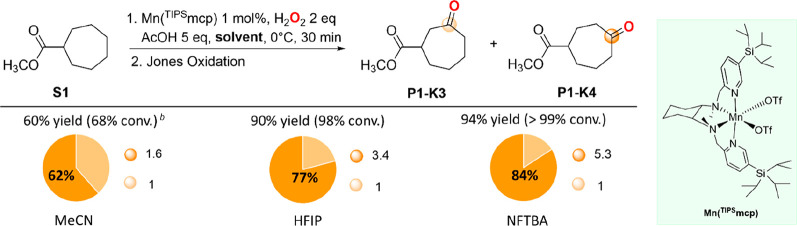
Oxidation of Methyl Cycloheptanecarboxylate (**S1**) with H_2_O_2_ Catalyzed by Mn(^TIPS^mcp) Selectivities are expressed in terms of the ratio between major product and total product yield: **P1-K4**/(**P1-K3** + **P1-K4**). Employing 3.0 equiv of H_2_O_2_.

**Scheme 4:**
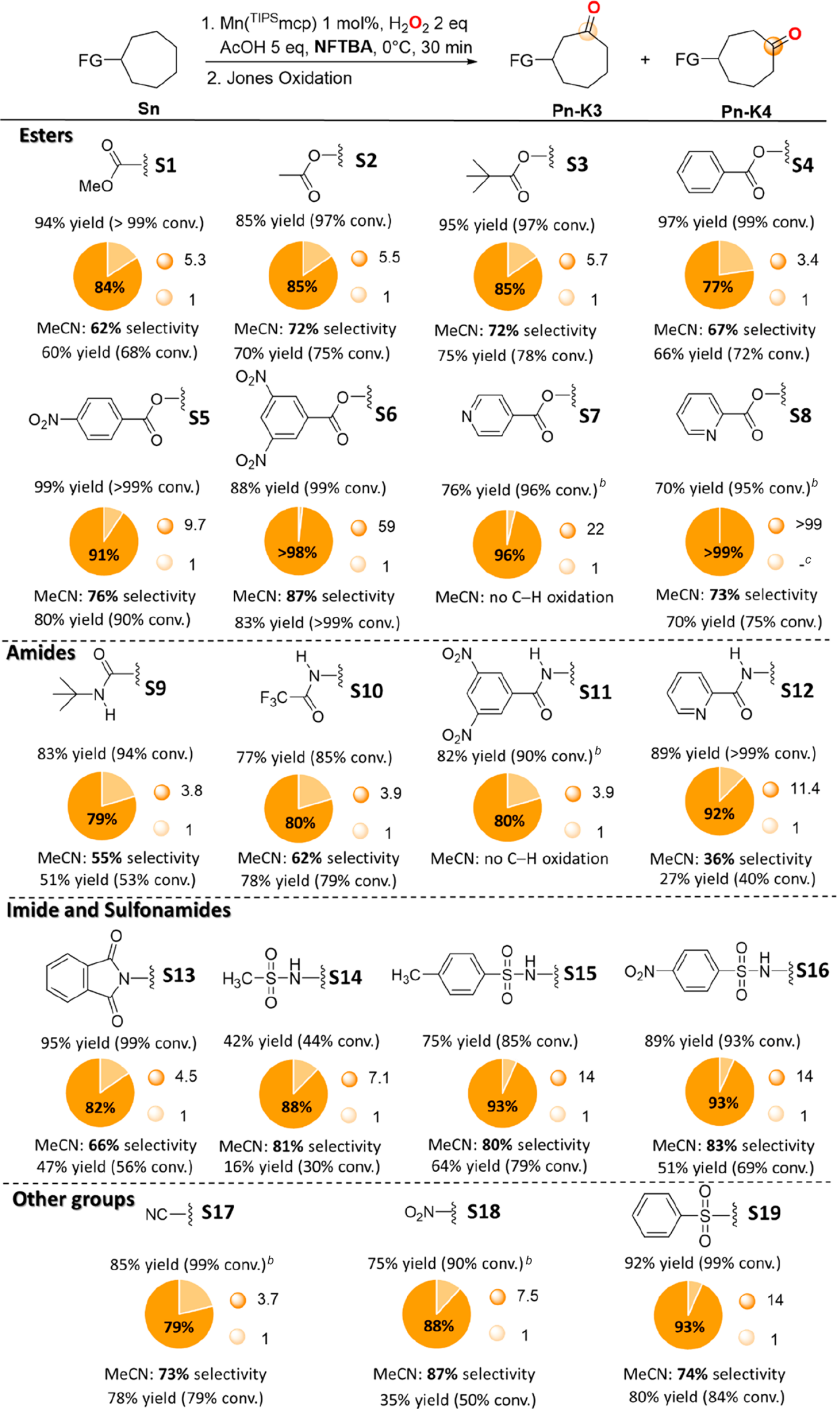
Oxidation of Cycloheptyl Substrates **S1**–**S19** with H_2_O_2_ Catalyzed by Mn(^TIPS^mcp) Selectivities are expressed in terms of the ratio between major product and total product yield: **Pn-K4**/(**Pn-K3** + **Pn-K4**). Employing HFIP as the solvent. Not detected.

**Scheme 5:**
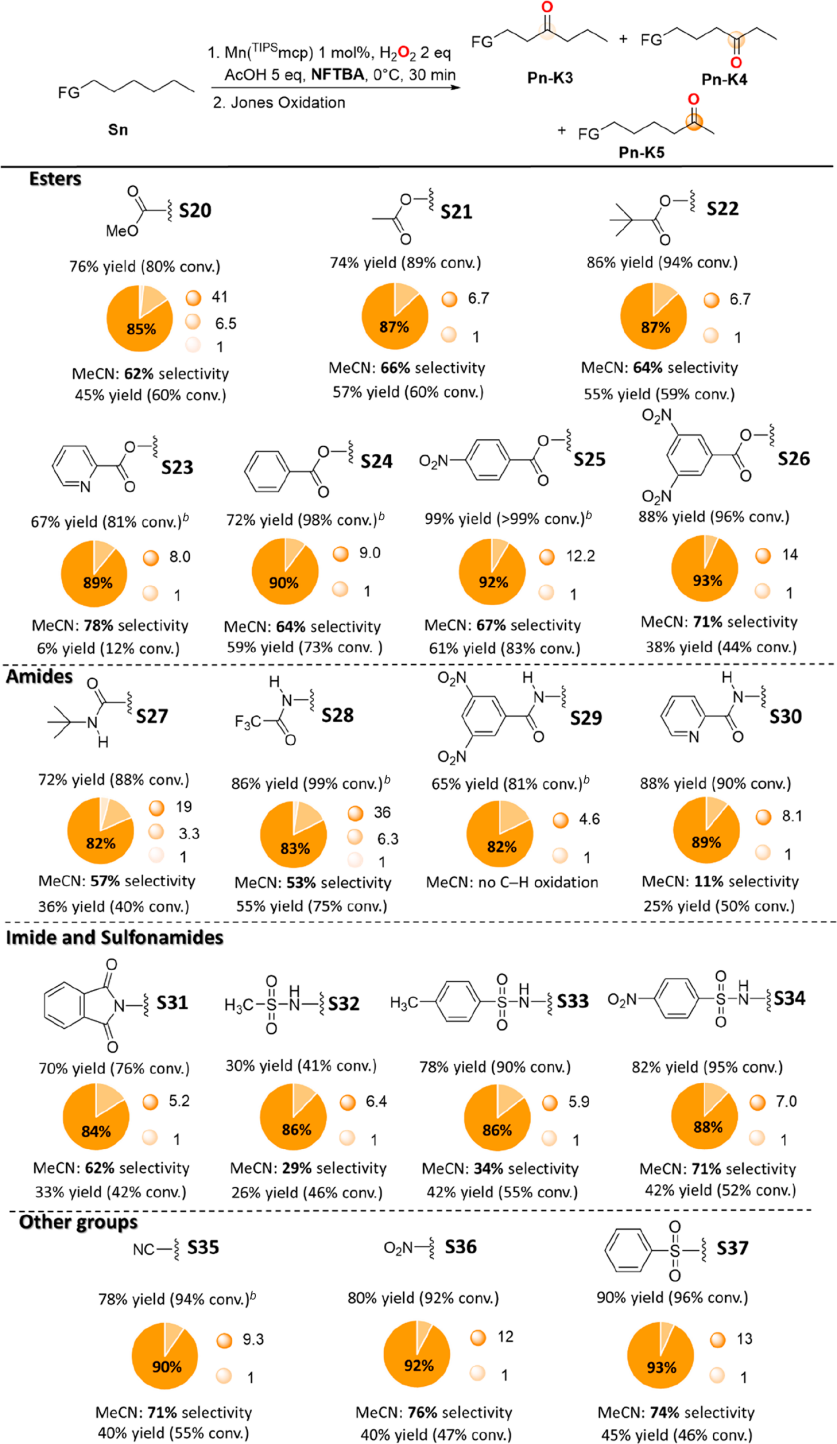
Oxidation of 1-Hexyl Substrates **S20**–**S37** with H_2_O_2_ Catalyzed by Mn(^TIPS^mcp) Selectivities are expressed in terms of the ratio between major product and total product yield: **Pn-K5**/(**Pn-K5** + **Pn-K4** + **Pn-K3** + **CH_3_(CH_2_)_4_CHO**). Employing HFIP as the solvent.

With both substrate groups, the oxidation reactions in MeCN have been compared to the corresponding ones in 1,1,1,3,3,3-hexafluoro-2-propanol (HFIP) and nonafluoro *tert*-butyl alcohol (NFTBA). Compared to HFIP, NFTBA, although employed to date only in a limited number of cases, is characterized by a stronger acidity, and a stronger HBD ability can be envisaged,^16^ making it particularly well suited to expand the concept of *polarity enhancement* in C(sp^3^)–H bond functionalization. With both substrate groups, oxygenation at the most remote over the next methylenic sites has been observed in HFIP and NFTBA, extending the strong electronic deactivation determined by native EWGs displayed in Scheme 2b by two carbon atoms, thus allowing remote C–H bond functionalization of the 1-hexyl and cycloheptyl derivatives with unprecedented levels of site-selectivity (up to 93% and >99%, respectively). In addition, under these conditions, product yields and mass balances are systematically higher than those observed in acetonitrile. Collectively, high yielding and predictable site-selective oxidation of methylenic sites in cycloheptyl and 1-hexyl derivatives, which in the former translates into a transannular functionalization methodology, occurs with a large substrate scope, providing a simple and powerful tool to be implemented in synthetically useful procedures.

## Results and Discussion

Methyl cycloheptanecarboxylate (**S1**) was selected as the model substrate for the reaction optimization. The oxidation of **S1** was initially performed using 3.0 equiv of H_2_O_2_, delivered over 30 min using a syringe pump, in the presence of 5 equiv of a carboxylic acid and 1 mol % of a manganese catalyst, at 0 °C in MeCN as the solvent (0.125 M substrate concentration). C(sp^3^)–H bond oxidation led to the formation of ketonization product mixtures. Under these conditions, reaction optimization identified [Mn(OTf)_2_(^TIPS^mcp)] (from now on indicated as Mn(^TIPS^mcp))^17^ and acetic acid as the best performing catalyst and carboxylic acid, respectively (see Supporting Information, Tables S1–S3). The oxidation of **S1** occurred preferentially at the C-4 over C-3 methylenic site to produce the corresponding ketoester products (**P1**-**K4** and **P1**-**K3**, respectively) in 60% total yield and a 1.6:1 ratio (62% C-4 selectivity, Scheme 3), in excellent agreement with the results of previous studies on the oxidation of **S1** with hydrogen peroxide in MeCN catalyzed by the Fe(pdp) complex (Scheme 2b).^7^ No products arising from competitive oxidation at C-1 and C-2 were observed.

By changing the solvent to HFIP and NFTBA and employing 2.0 equiv of H_2_O_2_, significant increases in product yields and selectivity for oxidation at C-4 were observed, accompanied, however, by the formation of hydroxyester and ketoester product mixtures. In order to simplify product identification and quantitative analysis, the reaction mixtures were subjected to follow-up treatment with chromic acid (Jones oxidation), leading to the formation of a single ketoester product for each oxidizable site by quantitative oxidation of all of the hydroxylated carbons into the corresponding carbonyl groups (see SI). Under these conditions, the C-4 selectivities approached 77% and 84% (3.4 and 5.3 **P1-K4**/**P1-K3** ratios), in HFIP and NFTBA, respectively (Scheme 3). The improvement in product yield (≥90% of combined ketoesters) observed on going from MeCN to R^F^OHs presumably reflects more efficient H_2_O_2_ activation and enhanced electrophilicity of the reactive metal oxo via solvent hydrogen bonding. These high yields are quite remarkable in the framework of C(sp^3^)–H functionalizations, considering in particular that the substrate is used as the limiting reagent. Compared to MeCN, the formation of large amounts of hydroxylation products observed in R^F^OHs, before treatment with chromic acid (see SI, Tables S4 and S5), reflects protection against overoxidation determined by *polarity reversal* (Scheme 2c).^8,11c,11d,11h^ The observed increase in site-selectivity can be instead rationalized on the basis of *polarity enhancement* determined by interaction of the ester group with the HBD solvents that results in stronger deactivation at proximal C–H bonds, increasing selectivity for oxidation at the most remote and least electronically deactivated site (Scheme 2c).

Building on these results, the study was extended to the reactions of cycloheptyl substrates bearing a broad range of FGs (**S2**–**S19**). For all substrates oxygenation was carried out in MeCN, HFIP, and NFTBA and the pertinent results are displayed in Scheme 4. For the sake of simplicity, only the results obtained in NFTBA (generally the best R^F^OH, unless otherwise indicated) are displayed, with comparison to the corresponding yield and selectivity obtained in MeCN (full details of the results obtained in all three solvents are displayed in the SI).

With all substrates, formation of the ketonization products (**Pn-K3** and **Pn-K4**) deriving from oxygenation at C-3 and C-4 in generally excellent overall yield and mass balance was observed, with **Pn-K4**/**Pn-K3** ratios and associated C-4 selectivities that increased going from MeCN to R^F^OHs.

Starting from the ester derivatives and taking **S1** as the reference substrate, comparable selectivities were observed in NFTBA for the oxidation of cycloheptyl acetate and pivalate **S2** and **S3** (Scheme 4, **Pn-K4**/**Pn-K3** between 5.5 and 5.7, 85% selectivity). A slight decrease in selectivity to 77% (**P4-K4**/**P4-K3** = 3.4) was instead observed for cycloheptyl benzoate (**S4**). However, by replacing benzoate with 4-nitrobenzoate as in **S5**, 91% selectivity was obtained (**P5-K4**/**P5-K3** = 9.7), and the use of the 3,5-dinitrobenzoate group, recently popularized by DuBois and Sigman,^14^ as in **S6**, delivered **P6-K4** in an outstanding 98.3% selectivity over **P6-K3** (**P6-K4**/**P6-K3** = 59). The increasingly stronger EW character of these benzoate derivatives determined by introduction of NO_2_ groups is also evidenced by the increase in selectivity observed in MeCN (67%, 76%, and 87%, for **S4**, **S5**, and **S6**, respectively), with the results obtained in R^F^OHs that highlight the potential of this approach in achieving high selectivity for oxygenation at the most remote methylenic site via synergistic electronic deactivation coupled to solvent hydrogen bonding.

Along this line, we reasoned that pyridinecarboxylate esters, characterized by the presence of the HBA pyridine moiety,^18^ could also provide a useful handle to improve selectivity. Gratifyingly, in HFIP, oxidation of cycloheptyl 4-pyridinecarboxylate (**S7**) delivered **P7-K4** in 96% selectivity over **P7-K3** (Scheme 4, **P7-K4**/**P7-K3** = 22), and in the corresponding reaction of cycloheptyl 2-pyridinecarboxylate (**S8**), exclusive formation of the product deriving from oxidation at C-4 (**P8-K4**) in 65% isolated yield was observed, with no detection of the isomeric product deriving from oxidation at C-3 (>99% selectivity, **P8-K4**/**P8-K3** > 99). With both **S7** and **S8**, lower selectivities were observed in NFTBA (**P7-K4**/**P7-K3** = 13 and **P8-K4**/**P8-K3** = 35), a behavior that can be rationalized on the basis of the greater steric bulk of NFTBA compared to HFIP that prevents optimal hydrogen bonding to the pyridine nitrogen atom. It is also worth noting that when the reaction of **S7** was studied in MeCN, exclusive formation of the product deriving from oxidation at the nitrogen center was observed (see SI), evidencing once again the potential of solvent hydrogen bonding to divert site-selectivity in the oxidation of electron-rich sites.

Moving then to the amide and phthalimide derivatives, comparable selectivities were observed in NFTBA for the oxidation of *N-tert*-butylcycloheptanecarboxamide (**S9**), *N*-cycloheptyltrifluoroacetamide (**S10**), *N*-cycloheptyl-3,5-dinitrobenzamide (**S11**), and *N*-cycloheptylphthalimide (**S13**) (Scheme 4, **Pn-K4**/**Pn-K3** between 3.8 and 4.5, 79–82% selectivity). Quite surprisingly, a completely different outcome was obtained when the 3,5-dinitrobenzoyl group was bound to nitrogen instead of oxygen (compare **S6** with **S11**). Significantly larger increases in selectivity were instead observed in the reactions of *N*-cycloheptyl 2-pyridinecarboxamide (**S12**) (92% selectivity, **P12-K4**/**P12-K3** = 11.4) and sulfonamides, approaching **Pn-K4**/**Pn-K3** ratios of 14 and 93% selectivity with the tosyl and nosyl derivatives **S15** and **S16**. Most interestingly, the same trend was also observed in MeCN with the sulfonamide derivatives **S14**–**S16** that displayed selectivities that were in all cases significantly higher than those observed with the amide and imide ones (**S9**, **S10**, and **S13**), approaching 83% in the reaction of **S16**. The low C-4 selectivity observed in MeCN for the reaction of **S12** (36%) reflects competitive oxidation at C-1,^19^ with the corresponding hydroxylation product **P12-A1** being observed among the reaction products in comparable amount to **P12-K3** and **P12-K4** (**P12-A1**:**P12-K3**:**P12-K4** = 1.0:1.0:1.2, see SI). Formation of **P12-A1** indicates that in MeCN the nitrogen atom of **S1** is still sufficiently electron rich to hyperconjugatively activate the C_1_–H bond. Under the same conditions, no reaction was observed for **S11**.

It is also very interesting to compare yields and selectivities obtained in the oxygenation of cycloheptanecarbonitrile (**S17**), nitrocycloheptane (**S18**), and cycloheptyl phenyl sulfone (**S19**) displayed in Scheme 4. In MeCN, a significant increase in selectivity was observed going from **S17** and **S19** to **S18** (73–74% and 87% (**Pn-K4**/**Pn-K3** = 2.7–2.8 and 6.7), respectively), in line with the stronger EW character of NO_2_ compared to the CN and PhSO_2_ groups.^20^ On the other hand, in NFTBA **S19** displayed the largest increase in selectivity (93% selectivity, **P19-K4**/**P19-K3** = 14), whereas no sizable effect was observed for **S18**. This behavior can be accounted for on the basis of the FG HBA ability that increases in the order NO_2_ < CN < PhSO_2_,^18^ highlighting once again the important role played by solvent hydrogen bonding in governing site-selectivity. Of note, the high site-selectivities obtained in HFIP and NFTBA are in parallel with consistently good to excellent product yields (75–92%).

The results obtained in the reactions of 1-hexyl substrates bearing a broad range of FGs (**S20**–**S37**) are displayed in Scheme 5. Also for these compounds, oxygenation was carried out in MeCN, HFIP, and NFTBA, and only the results obtained in NFTBA (generally the best R^F^OH unless otherwise indicated) are displayed, with comparison to the corresponding selectivities in MeCN (full details of the results obtained in all three solvents are displayed in the SI). With all substrates, formation of the ketonization products (**Pn-K4** and **Pn-K5**) deriving from oxygenation at C-4 and C-5 was observed, accompanied in some cases by smaller amounts of **Pn-K3**, deriving from oxygenation at C-3 and, in the specific case of the 1-hexyltrifluoroacetamide, 2-pyridinecarboxamide, methanesulfonamide, and *p*-toluenesulfonamide substrates (**S28**, **S30**, **S32**, and **S33**), by hexanal, deriving from initial oxidation at C-1 (see below). Product yields and selectivities for ketonization at the most remote methylenic site increased in all cases on going from MeCN to R^F^OHs.

Starting from the ester substrates (Scheme 5), with methyl heptanoate (**S20**), 1-hexyl acetate (**S21**), 1-hexyl pivalate (**S22**), 1-hexyl benzoate (**S24**), and 1-hexyl 4-nitrobenzoate (**S25**), comparable selectivities for oxidation at the most remote (C-6 for **S20**, C5 for the 1-hexyl esters) over proximal methylenic sites were observed in MeCN (62–67% selectivity, **Pn-K5**/**Pn-K4** between 1.7 and 2.0). Compared to these substrates, an increase in selectivity was observed for the corresponding reactions of 1-hexyl 2-pyridinecarboxylate (**S23**) and 1-hexyl 3,5-dinitrobenzoate (**S26**) (78% and 71% selectivity (**Pn-K5**/**Pn-K4** = 3.5 and 2.4), respectively). Going from MeCN to NFTBA, a progressive increase in selectivity for oxidation at the most remote methylenic site was observed along the series, approaching 93% in the reaction of **S26** (**P26-K5**/**P26-K4** = 14).

**Scheme 6:**
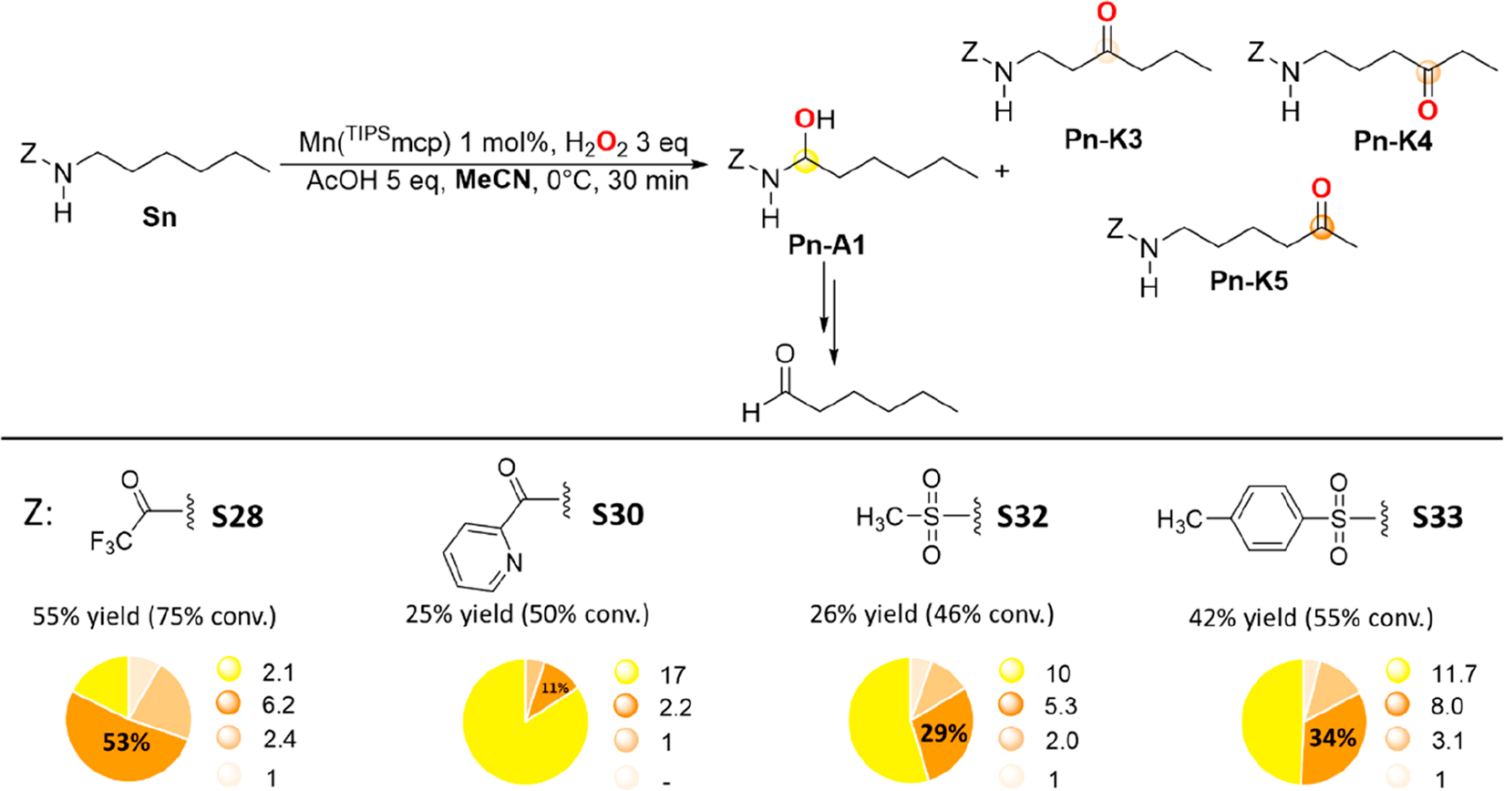
Oxidation of 1-Hexyl Amide and Sulfonamide Substrates **S28**, **S30**, **S32**, and **S33** with H_2_O_2_ Catalyzed by Mn(^TIPS^mcp) In the pie charts, the yellow slice formally represents the amount of product **Pn-A1**, quantified in terms of experimentally observed hexanal, derived from its decomposition in the reaction medium.

Moving then to the 1-hexyl amide, phthalimide, and sulfonamide substrates, the low to very low selectivities observed in MeCN in the reactions of **S28**, **S30**, **S32**, and **S33** (between 11% and 53%), can be accounted for on the basis of competitive oxidation at C-1, with hexanal, observed among the reaction products, that derives from the decomposition of the first formed hydroxylation product (Scheme 6).^19^

In keeping with the discussion outlined above for the oxidation of **S12** in MeCN, despite the EW character of the trifluoroacetyl, 2-pyridinecarbonyl, mesyl, and tosyl groups, the nitrogen atom of these substrates is still sufficiently electron rich to hyperconjugatively activate the C_1_–H bonds. Along this line, with *N*-hexylphthalimide (**S31**) and *N*-hexyl 4-nitrophenylsulfonamide (**S34**), bearing stronger EW groups, products derived from oxidation at C-1 were not detected. Also within this substrate series, no reaction was observed in MeCN for *N*-hexyl-3,5-dinitrobenzamide (**S29**).

Going from MeCN to NFTBA, a significant increase in selectivity for oxidation at the most remote methylenic site was observed for all the amide, imide, and sulfonamide substrates, approaching 88% and 89% in the reactions of **S34** and **S30** (Scheme 5, **Pn-K5**/**Pn-K4** = 7.0 and 8.1, respectively).

With heptanenitrile (**S35**), 1-nitrohexane (**S36**), and 1-hexyl phenyl sulfone (**S37**), comparable selectivities for oxidation at the most remote methylenic site (C-6 for **S35**, C5 for **S36** and **S37**) were observed in MeCN (between 71% and 76%). For all three substrates, a significant increase in selectivity was observed in HFIP and NFTBA (90%, 92%, and 93%, for **S35**, **S36**, and **S37**, approaching, with the latter substrate, a ratio **P37-K5**/**P37-K4** = 13.0). In contrast to the results obtained with the corresponding cycloheptyl derivatives, with these substrates, no clear correlation with FG EW character and HBA ability is observed, pointing toward differences in the transmission of electronic effects within the two substrate series.

With these results in hand, the scope of the *polarity enhancement* effect in achieving selective functionalization at remote methylenic sites of alcohol and amine derivatives was investigated, extending the study to cyclohexyl, cyclooctyl, 1-pentyl, and 1-heptyl substrates. The reactions of the acetate esters (**S38**, **S39**, **S44**, and **S45**), were initially investigated. However, because pyridinecarboxylates, 3,5-dinitrobenzoates, 2-pyridinecarboxamides, and sulfonamides were identified as preferential groups for highly selective remote oxyfunctionalization of alcohol and amine derivatives (see Scheme 4 and Scheme 5), oxidation of the following derivatives was also studied: cycloalkyl 2-pyridinecarboxylates (**S40** and **S41**), cycloalkyl 4-nitrobenzenesulfonamides (**S42** and **S43**), 1-alkyl 3,5-dinitrobenzoates (**S46** and **S47**), 1-alkyl 4-nitrobenzenesulfonamides (**S48** and **S49**), and 1-alkyl 2-pyridinecarboxamides (**S50** and **S51**).

**Scheme 7:**
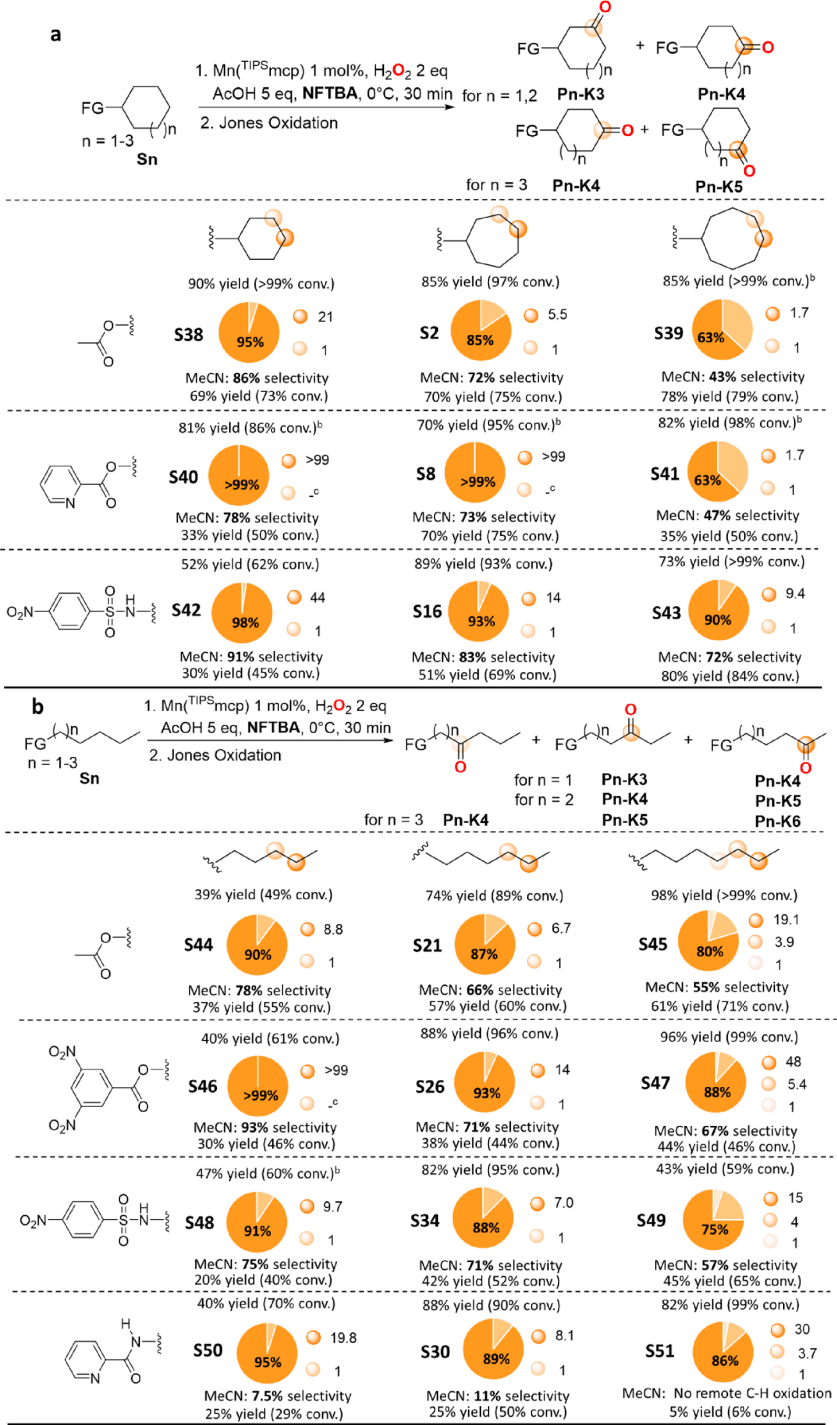
Results Obtained in the Oxidation of (a) Cycloalkyl (**S38**–**S43**) and (b) 1-Alkyl (**S44**–**S51**) Derivatives with H_2_O_2_ Catalyzed by Mn(^TIPS^mcp), with Comparison to the Corresponding Cycloheptyl and 1-Hexyl Derivatives Selectivities are expressed in terms of the ratio between major product and total product yield. With the cyclohexyl and cyclooctyl derivatives, selectivities are statistically corrected for the number of C–H bonds. Employing HFIP as the solvent. Not detected.

Starting from the cycloalkyl acetates (Scheme 7a), a 95% C-4 selectivity (statistically corrected) was observed in NFTBA for oxidation of cyclohexyl acetate (**S38**) (**P38-K4**/**P38-K3** = 21). This result compares with the 85% C-4 selectivity obtained in the corresponding reaction of cycloheptyl acetate (**S2**) (**P2-K4**/**P2-K3** = 5.5) and the 63% C-5 selectivity (statistically corrected) for oxidation of cyclooctyl acetate (**S39**) (**P39-K5**/**P39-K4** = 1.7). Analogously, with the 1-alkylacetates (Scheme 7b), a 90% C-4 selectivity was observed in NFTBA for oxidation of 1-pentylacetate (**S44**) (**P44-K4**/**P44-K3** = 8.8), with the selectivity for oxidation at the most remote methylenic site that decreased to 87% and 80% (**P21-K5**/**P21-K4** = 6.7 and **P45-K6**/(**P45-K5 + P45-K4**) = 3.9), respectively, going to 1-hexyl (**S21**) and 1-heptylacetate (**S45**). These results are in line with the distance dependence of the deactivating electronic effect that decreases with increasing ring size and chain length.^3^ In MeCN, because of the lack of the *polarity enhancement* effect, intrinsically lower selectivities were observed with both substrate groups, accompanied by larger decreases in site-selectivity along the series (86%, 72%, and 43% for **S38**, **S2**, and **S39** and 78%, 66%, and 55%, for **S44**, **S21**, and **S45**, respectively).

Most importantly, significantly higher selectivities for oxidation at the most remote methylenic site were observed in NFTBA when the acetate group was replaced by 2-pyridinecarboxylate for the cycloalkyl substrates and by 3,5-dinitrobenzoate for the 1-alkyl substrates. With the cycloalkyl 2-pyridinecarboxylates (Scheme 7a), the following (statistically corrected) selectivities were observed for the cyclohexyl (**S40**), cycloheptyl (**S8**), and cyclooctyl (**S41**) derivatives: >99%, >99%, and 63%, corresponding to product ratios of >99, >99, and 1.7, respectively. With the 1-alkyl 3,5-dinitrobenzoates (Scheme 7b), the following selectivities were observed for the 1-pentyl (**S46**), 1-hexyl (**S26**), and 1-heptyl (**S47**) derivatives: >99%, 93%, and 88%, corresponding to product ratios of >99, 14, and 7.5, respectively. To the best of our knowledge, these levels of selectivity for remote nondirected C(sp^3^)–H oxidation at methylenic sites of cycloalkyl and 1-alkyl esters are unprecedented. In previous studies of HAT-based functionalization of analogous derivatives, significantly lower selectivities were systematically observed. For example, the highest selectivities approached 75% and 85% (3:1 (statistically corrected) and 5.7:1 **C4**/**C3** product ratios), respectively, in the oxygenation of cyclohexyl and cycloheptyl 4-chlorobenzoates promoted by TFDO,^6^ 84% (5.4:1 product ratio) in the fluorination of cycloheptylbenzoate (**S4**) promoted by a Mn-oxo porphyrin,^21^ 93% (12.5:1 product ratio) in the oxygenation of 1-pentyl 3,5-dinitrobenzoate (**S46**) in 4:1 dichloroacetic acid/H_2_O by the CAN/*cis*-[4,4′-MeO-bpyRuCO_3_] system,^14^ 88% (7.0:1 product ratio) in the chlorination of 1-pentylacetate (**S44**) promoted by the *cis*-2,6-dimethylpiperidinium radical,^22^ and 76% (3.2:1 product ratio) in the oxygenation of 1-hexylacetate (**S21**) by the H_2_O_2_/Fe(mcp) system.^23^

For what concerns the remote oxyfunctionalization of amine derivatives, the following (statistically corrected) selectivities were observed in NFTBA for the cyclohexyl (**S42**), cycloheptyl (**S16**), and cyclooctyl (**S43**) 4-nitrobenzenesulfonamides (Scheme 7a): 98%, 93%, and 90% (corresponding to 44, 14, and 9.4 product ratios), respectively. The latter value is particularly remarkable in the framework of the remote C(sp^3^)–H functionalization of cyclooctyl derivatives. Interestingly, within this substrate group relatively high selectivities were also observed in MeCN (91%, 83%, and 72% for **S42**, **S16**, and **S43**, respectively), pointing toward nosyl as a preferential group for these reactions.

With 1-alkyl substrates (Scheme 7b), the following selectivities were observed in NFTBA for the 1-pentyl (**S48**), 1-hexyl (**S34**), and 1-heptyl (**S49**) 4-nitrobenzenesulfonamides: 91%, 88%, and 75% (9.7, 7.0, and 3.0 product ratios), and for the 1-pentyl (**S50**), 1-hexyl (**S30**), and 1-heptyl (**S51**) 2-pyridinecarboxamides: 95%, 89%, and 86% (19.8, 8.1, and 6.4 product ratios), respectively.

Again, and to the best of our knowledge, the levels of selectivity for remote nondirected C(sp^3^)–H oxidation obtained for the cycloalkylamine, 1-hexylamine, and 1-heptylamine derivatives are unprecedented. For example, in previous studies on HAT-based functionalization of cycloalkylamine derivatives, the highest (statistically corrected) selectivities approached 67% and 79% (2:1 and 3.7:1 product ratio), respectively, in the oxidation of *N*-cycloheptylphthalimide (**S13**) and protonated *N*-cyclohexylmorpholine by the H_2_O_2_/Fe(CF_3_-pdp) system.^5^ On the other hand, with 1-pentylamine derivatives, >99% C-4 selectivity was observed in the oxidation of protonated *N*-pentylmorpholine by the H_2_O_2_/Fe(CF_3_-pdp) system^5^ and of *N*-pentylphthalimide by the H_2_O_2_/Mn(^dMM^pdp) system,^19^ and 91% (9.7:1 product ratio), in the oxidation of *N*-(methylsulfonyl)-*N*-pentylmethanesulfonamide in 4:1 dichloroacetic acid/H_2_O by the CAN/*cis*-[4,4′-MeO-bpyRuCO_3_] system.^14^

**Scheme 8:**
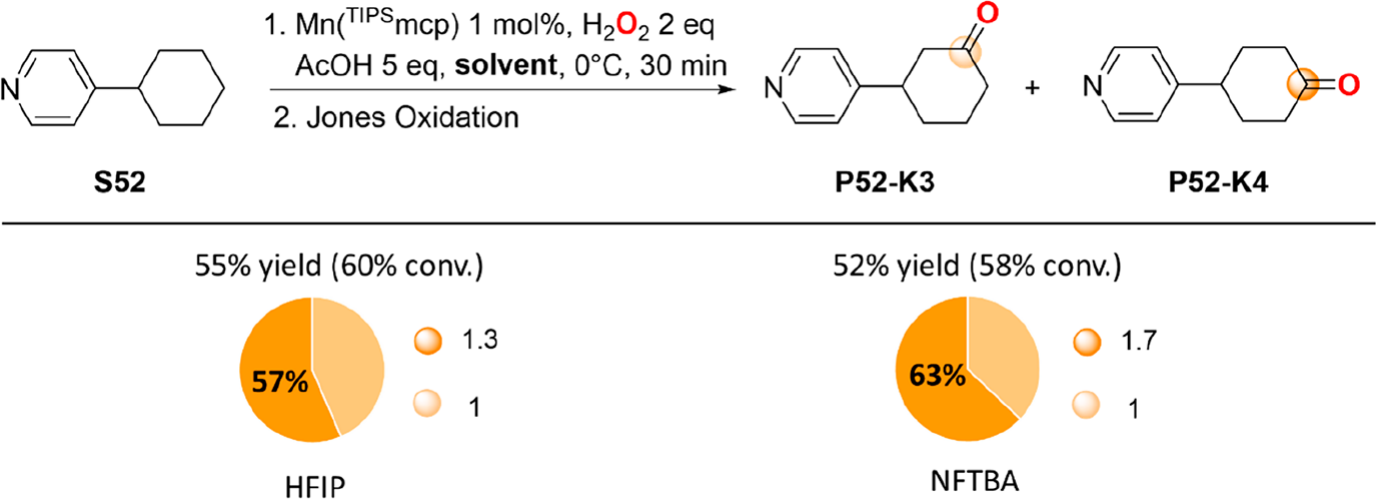
Results Obtained in the Oxidation of 4-Cyclohexylpyridine (**S52**) with H_2_O_2_ Catalyzed by Mn(^TIPS^mcp), in HFIP and NFTBA Selectivities are expressed in terms of the ratio between major product and total product yield and are statistically corrected for the number of C–H bonds.

Finally, the effect of R^F^OHs on the oxygenation site-selectivity was also investigated for the reaction of 4-cyclohexylpyridine (**S52**), and the results are displayed in Scheme 8. No substrate conversion was observed in MeCN, whereas in HFIP and NFTBA formation of the ketone products derived from oxygenation at C-3 and C-4 in moderate yield and selectivity (57% and 63%, respectively, 1.3:1 and 1.7:1 **P52-K4**/**P52-K3** ratios) was observed, accompanied in both cases by very good mass balances. As a matter of comparison, in MeCN a 1:1.3 **P52-K4**/**P52-K3** ratio was recently observed in the oxidation of protonated **S52** by the H_2_O_2_/Fe(CF_3_-pdp) system.^5^

Recent studies on the oxidation of 2-(4-methylpentyl)- and 4-(4-methylpentyl)pyridine with H_2_O_2_ catalyzed by Mn(mcp) have shown that in HFIP hydroxylation selectively occurs at the remote tertiary C–H bond, a behavior that was rationalized in terms of solvent hydrogen bonding to the pyridine nitrogen atom that deactivates proximal sites toward functionalization.^15^ The results obtained with **S52** indicate however that the origin of the selectivity observed in the latter study mostly reflects the presence of an intrinsically more activated tertiary C–H bond and, only to a lesser extent, the *polarity enhancement* effect, highlighting the challenges associated with site-selective secondary C–H bond functionalization. Based on these results, pyridine groups emerge as poor structural motifs for implementing site-selectivity in these reactions via *polarity enhancement*, showing on the other hand that when incorporated into pyridinecarboxylate and pyridinecarboxamide moieties, the two EWG groups synergistically cooperate to promote strong deactivation, providing outstanding selectivities for remote C–H bond functionalization at methylenic sites.

## Conclusions

Unprecedented levels of site-selectivity for nondirected C(sp^3^)–H bond oxygenation at remote methylenic sites were obtained in HFIP and NFTBA, in the oxidation with H_2_O_2_ catalyzed by Mn(^TIPS^mcp) of cycloalkyl and 1-alkyl substrates bearing a variety of EWGs. The results were rationalized on the basis of a *polarity enhancement* effect via synergistic electronic deactivation of proximal sites imparted by the EW functional group coupled to solvent hydrogen bonding to this group. The use of fluorinated alcohol solvents leads moreover to product yields and conversions that are systematically higher than those observed for the corresponding reactions in MeCN. Compared to previously described procedures, *polarity enhancement* provides the opportunity to tune site-selectivity among multiple methylenic groups in different substrate classes, extending the strong electronic deactivation determined by native EWGs by two carbon atoms and allowing highly selective remote C–H bond functionalization of cyclohexyl, cycloheptyl, 1-pentyl, and 1-hexyl substrates, which in the cycloalkyl derivatives translates into a transannular functionalization methodology. Pyridinecarboxylates, 4-nitrobenzoates, 3,5-dinitrobenzoates, 2-pyridinecarboxamides, and sulfonamides emerge as preferential groups for remote oxyfunctionalization of alcohol and amine derivatives. High site-selectivities are also observed in the reactions of the nitro and phenylsulfone derivatives, functional groups that are amenable to straightforward follow-up elaboration. Taken together, this study uncovers a simple procedure for predictable and high-yielding site-selective oxidation at remote methylenic sites of cycloalkyl and 1-alkyl derivatives that occurs under mild conditions, with a large substrate scope, providing a powerful tool to be implemented in synthetically useful procedures.

## Supporting Information Available

Experimental details for the preparation of the substrates, optimization of the oxidation reactions, and details on isolation and characterization of the reaction products (PDF)
