# Supplementary material for: Highly Selective C(sp3)–H Bond Oxygenation at Remote Methylenic Sites Enabled by Polarity Enhancement
Source: J Am Chem Soc. 2023 Sep 26;145(40):22086–96. doi: 10.1021/jacs.3c07658 (PMC10571082; doi:10.1021/jacs.3c07658)
Supplement: Supplementary file 1 — ja3c07658_si_001.pdf [file ja3c07658_si_001.pdf]

Supporting information

Highly Selective C(sp^3)-H Bond Oxygenation at Remote Methylenic Sites Enabled by Polarity Enhancement

Sergio Sisti,[1§] Marco Galeotti,[2§] Filippo Scarchilli,[2] Michela Salamone,[1] Miquel Costas,[2,*] and Massimo Bietti [1,*]

[1] *Dipartimento di Scienze e Tecnologie Chimiche, Università "Tor Vergata", Via della Ricerca Scientifica, 1 I-00133 Rome, Italy.*

[2] *QBIS Research Group, Institut de Química Computacional i Catàlisi (IQCC) and Departament de Química, Universitat de Girona, Campus Montilivi, Girona E-17071, Catalonia, Spain.*

[§]*Equal contribution*

Contents

1. Experimental section ... 3

1.1. Instrumentation ... 3

1.2. Materials ... 3

2. Synthesis of the substrates ... 4

2.1. Synthesis of methyl cycloheptane carboxylate (S1) ... 4

2.2. Synthesis of esters and amides ... 5

2.3. Synthesis of the sulfonamide substrates ... 13

2.4. Synthesis of (phenylsulfonyl)cycloheptane (S19) and (phenylsulfonyl)hexane (S37). ... 16

2.4.1. Synthesis of the sulfides ... 16

2.4.2. Synthesis of the sulfones ... 16

2.5. Synthesis of *N*-cycloheptyl-2,2,2-trifluoroacetamide (S10) and *N*-hexyl-2,2,2-trifluoro acetamide (S28) ... 17

2.6. Synthesis of *N*-(*tert*-butyl)cycloheptanecarboxamide (S9) and *N*-(*tert*-butyl)heptanamide (S27) 18

2.7. Synthesis of cycloheptanecarbonitrile (S17) ... 19

2.8. Synthesis of *N*-cycloheptylphthalimide (S13) and *N*-hexylphthalimide (S31) ... 20

2.9. Synthesis of nitrocycloheptane (S18) ... 21

2.10. Synthesis of 4-cyclohexylpyridine (S52) ... 21

3. Synthesis of the manganese complexes ... 22

4. Oxidation with H_2O_2 catalyzed by Mn complexes ... 23

4.1. General procedure ... 23

4.1.1. Chromic acid oxidation ... 23

4.2. Optimization of the reaction conditions ... 24

4.2.1. Optimization of S1 oxidation in MeCN ... 24

4.2.2. Optimization of S1 oxidation in fluorinated solvents (HFIP and NFTBA) ... 26

4.3. Results obtained in the oxidation of substrates S1-S52 in MeCN and in fluorinated solvents with H_2O_2 catalyzed by Mn(^{TIPS}mcp). ... 27

5. Isolation and characterization of the oxidation products ... 39

5.1. Scale-up oxidation of S1, S2, S7, S8, S20, S21, S26, S38, S40, S41, S43-S47. ... 39

5.2. Products identified by ^{1}H NMR analysis of the catalysis crude mixture ... 50

6. ^{1}H-NMR and ^{13}C-NMR spectra ... 63

6.1. NMR spectra of the substrates ... 63

6.2. NMR spectra of the isolated oxidation products ... 79

6.3. NMR spectra and GC chromatograms of product mixtures ... 92

7. References ... 146

1. Experimental section

1.1. Instrumentation

Gas-chromatographic analyses were carried out for the oxidation reactions with H_2O_2 catalyzed by manganese-oxo complexes using an Agilent 7820A gas chromatograph equipped with an HP-5 capillary column 30m x 0.32 mm x 0.25 µm and a flame ionization detector. GC-MS analyses were performed on an Agilent 7890A gas chromatograph equipped with an HP-5MS capillary column (30 m x 0.25 mm x 0.25 µm) interfaced with an Agilent 5975X mass spectrometer. NH_3 was used as the ionization gas. NMR spectra were taken on a Bruker Ultrashield AVANCE III400 or on a Bruker Ultrashield ASCEND Nanobay spectrometer using standard conditions. Spectra were referenced to the residual proton solvent peak or TMS (tetramethylsilane). High resolution mass spectra (HRMS) were recorded on a Bruker MicroTOF-Q IITM instrument with an ESI source and a quadrupole analyzer at Serveis Tecnics of the University of Girona. Samples were introduced into the mass spectrometer ion source by direct infusion through a syringe pump and were externally calibrated using sodium formate.

1.2. Materials

Reagents and solvents used were of commercially available reagent quality unless stated otherwise and are purchased from SDS, Aldrich, Scharlab and Fluorochem. Sigma-Aldrich HPLC-grade acetonitrile was employed for oxidation catalysis. The hydrogen peroxide solutions employed in the oxidation reactions were prepared by diluting commercially available hydrogen peroxide (50% H_2O_2 solution in water, Aldrich) in MeCN and in fluorinated solvents to achieve a ~1.0 M final concentration. All reagents used were of the highest commercial quality available unless stated otherwise.

2. Synthesis of the substrates

The substrates were prepared following the procedures reported below. Methyl heptanoate (**S20**), heptanenitrile (**S35**) and 1-nitrohexane (**S36**) are commercially available. The spectroscopic data of the pure products were compared with those reported in literature. The characterization of the unknown products was performed by ^{1}H-NMR, ^{13}C-NMR and HRMS.

2.1. Synthesis of methyl cycloheptane carboxylate (S1)

Methyl cycloheptane carboxylate (**S1**) were prepared according to a slightly modification of a reported procedure (**Scheme S1**).[1]

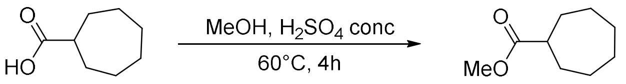

Scheme S1. Synthesis of methyl cycloheptane carboxylate (**S1**).

In a 50 mL two-necked round bottom flask equipped with a condenser system 2.1 g of cycloheptane carboxylic acid (15 mmol), 15 mL of methanol and 20 drops of concentrated H_2SO_4 were added. The reaction mixture was stirred at 60°C. After 4 hours, the complete substrate conversion was checked by TLC and GC and the solution was concentrated under vacuum. The crude residue was taken up in EtOAc (40 mL), extracted with saturated aqueous $NaHCO_3$ (2x20 mL) and then dried over $MgSO_4$. After filtration, the solution was concentrated under vacuum to afford the title compound as a colorless oil (1,85 g, 1.18 mmol, 79% yield), identified as methyl cycloheptane carboxylate (**S1**). The product was used without further purifications (>98% GC purity). Spectroscopic data match those previously reported.[1] ^{1}H-NMR (400 MHz, $CDCl_3$) δ, ppm: 3.65 (s, 3H), 2.48 (tt, 1H), 1.88−1.95(m, 2H), 1.42−1.75 (m, 10H).

2.2. Synthesis of esters and amides

The ester and amide substrates (**S2-S8**, **S11**, **S12**, **S21-S26**, **S29**, **S30**, **S38-S41**, **S44-S47**, **S50**, **S51**) were prepared according to a slight modification of a reported procedure (**Scheme S2**).[2]

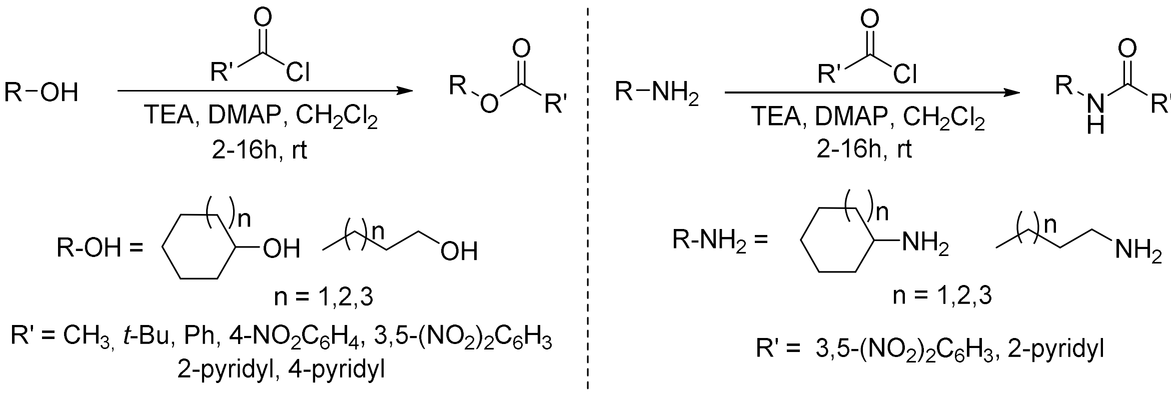

Scheme S2. Synthesis of the ester and amide substrates.

General procedure.

In a 250 mL round-bottom flask equipped with a septum and kept under nitrogen the alcohol or amine substrate (1.0 eq) and anhydrous CH_2Cl_2 (0.1 M) were added. Next, triethylamine (1.5-4.0 eq), and 4-dimethylaminopyridine (0.1 eq) were added to the solution. The reaction was cooled to 0 °C and the corresponding acyl chloride (1.1 eq) was added portion-wise over 10 minutes under magnetic stirring. The reaction was warmed to room temperature and stirred for an additional 16 hours. After complete substrate conversion (monitored by TLC or GC analysis), the reaction mixture was cooled at 0°C and carefully quenched with 75 mL of 2M HCl solution, and the organic layer was separated. Thus 75 mL of a saturated $NaHCO_3$ aqueous solution were then added and the solution was kept under stirring for 30 min to remove the excess of the acyl chloride. At this point, the organic phase was extracted with a saturated $NaHCO_3$ aqueous solution (2x75 mL), brine (2x75 mL) and then dried over $MgSO_4$. After filtration, the solution was concentrated under vacuum and the crude was purified by flash chromatography on silica gel (see below for purification conditions and characterization).

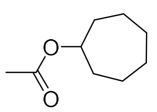

S2: The reaction of 1 g of cycloheptanol (8.8 mmol), gave after flash chromatography over silica gel in hexane-ethyl acetate (15:1) 1.1 g (81% yield) of a colorless liquid in >99% purity (GC), identified as cycloheptyl acetate (**S2**). Spectroscopic data match those previously reported.[3] ^{1}H-NMR (400 MHz, $CDCl_3$) δ, ppm: 4.88 – 4.92 (m, 1H), 2.01 (s, 3H), 0.90 – 1.91 (m, 12H).

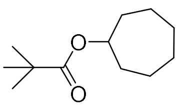

S3: The reaction of 1 g of cycloheptanol (8.8 mmol), gave after flash chromatography over silica gel in hexane-ethyl acetate (50:1) 0,87 g (50% yield) of a colorless liquid in >99% purity (GC), identified as cycloheptyl pivalate (**S3**). Spectroscopic data match those previously reported.[4] ^{1}H-NMR (400 MHz, $CDCl_3$) δ, ppm: 4.99 – 4.88 (m, 1H), 1.91 – 1.80 (m, 2H), 1.74 – 1.57 (m, 8H), 1.53 – 1.43 (m, 2H), 1.20 (s, 9H).

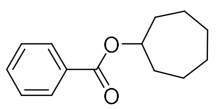

S4: The reaction of 2 g of cycloheptanol (17.7 mmol), gave after gave after flash chromatography over silica gel in hexane-ethyl acetate (50:1) 1.18 g (35% yield) of a colorless liquid in >99% purity (GC), identified as cycloheptyl benzoate (**S4**). Spectroscopic data match those previously reported.[5] ^{1}H-NMR (400 MHz, $CDCl_3$) δ, ppm: 8.07 (dt, 2H), 7.60–7.54 (m, 1H), 7.48–7.43 (m, 2H), 5.27–5.18 (m, 1H), 2.08–1.99 (m, 2H), 1.90–1.80 (m, 2H), 1.80–1.71 (m, 2H), 1.63 (ddd, 4H), 1.54 (ddd, 2H).

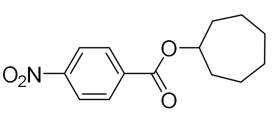

S5: The reaction of 1 g of cycloheptanol (8.8 mmol), gave after flash chromatography over silica gel in hexane-ethyl acetate (50:1) 0,55 g (24% yield) of a white solid in >99% purity (GC), identified as cycloheptyl-4-nitrobenzoate (**S5**). ^{1}H-NMR (400 MHz, $CDCl_3$) δ, ppm: 8.33–8.28 (m, 2H), 8.25–8.20 (m, 2H), 5.30–5.21 (m, 1H), 2.06 (ddd, 2H), 1.91–1.81 (m, 2H), 1.81–1.70 (m, 2H), 1.69–1.61 (m, 4H), 1.60–1.54 (m, 2H). ^{13}C NMR (400 MHz, $CDCl_3$) δ, ppm: 164.01, 150.40, 136.44, 130.61, 123.46, 77.02, 33.78, 28.28, 22.88. HRMS (QTOF) m/z calculated for $C_{14}H_{17}NO_4$ $[M+Na]^+$ 286.1050, found 286.1045.

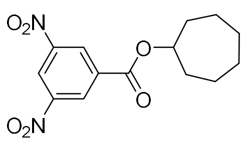

S6: The reaction of 1 g of cycloheptanol (8.8 mmol), gave after flash chromatography over silica gel in hexane-ethyl acetate (10:1) 2,4 g (88% yield) of a white solid in >99% purity (GC), identified as cycloheptyl 3,5-dinitrobenzoate (**S6**). ^{1}H-NMR (400 MHz, $CDCl_3$) δ, ppm: 9.24 (t, 1H), 9.17 (d, 2H), 5.35–5.27 (m, 1H), 2.10 (ddd, 2H), 1.89 (tdd, 2H), 1.83–1.74 (m, 2H), 1.69–1.65 (m, 4H), 1.60 (d, 1H). ^{13}C NMR (400 MHz, $CDCl_3$) δ, ppm: 176.57, 161.81, 148.63, 134.75, 129.38, 122.14, 78.54, 33.78, 28.21, 22.82. HRMS (QTOF) m/z calculated for HRMS (QTOF) m/z calculated for $C_{14}H_{16}N_2O_6$ $[M+Na]^+$ 331.0901, found 331.0901.

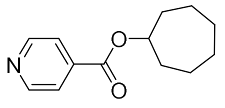

S7: The reaction of 1 g of cycloheptanol (8.8 mmol), gave after flash chromatography over silica gel in hexane-ethyl acetate (5:1) 1.26 g (65% yield) of a yellow pale liquid in >99% purity (GC), identified as cycloheptyl -pyridinecarboxylate (**S7**). ^{1}H-NMR (400 MHz, $CDCl_3$) δ, ppm: 8.79 (dd, 2H), 7.86 (dd, 2H), 5.29–5.19 (m, 1H), 2.04 (ddd, 2H), 1.90–1.82 (m, 2H), 1.82–1.69 (m, 3H), 1.67–1.60 (m, 4H), 1.60–1.50 (m, 2H). ^{13}C{^{1}H}-NMR (400 MHz, $CDCl_3$) δ, ppm: 164.41, 150.49, 138.21, 122.85, 76.81, 33.72, 28.27, 22.85. HRMS (QTOF) m/z calculated for $C_{13}H_{17}NO_2$ $[M+H]^+$ 220.1332, found 220.1330.

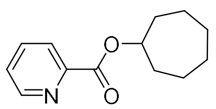

S8: The reaction of 1 g of cycloheptanol (8.8 mmol), gave after flash chromatography over silica gel in hexane-ethyl acetate (2:1) 0.98 g (51% yield) of a yellow pale liquid in >99% purity (GC), identified as cycloheptyl 2-pyridinecarboxylate (**S8**). ^{1}H-NMR (400 MHz, $CDCl_3$) δ, ppm: 8.79 (ddd, 1H), 8.12 (dt, 1H), 7.84 (td, 1H), 7.47 (ddd, 1H), 5.26 (tt, 1H), 2.14–2.06 (m, 2H), 1.9 –1.84 (m, 2H), 1.81–1.71 (m, 2H), 1.66–1.59 (m, 4H), 1.58–1.48 (m, 2H).^{13}C{^{1}H}-NMR (400 MHz, $CDCl_3$) δ, ppm: 164.52, 149.92, 148.81, 136.85, 126.56, 124.99, 77.07, 33.80, 28.15, 22.98. HRMS (QTOF) m/z calculated for $C_{13}H_{17}NO_2$ $[M+Na]^+$ 242.1151, found 242.1153.

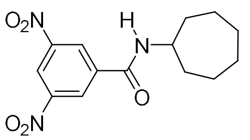

S11: The reaction of 0.5 g of cycloheptylamine (4.4 mmol), gave after crystallization in ethyl acetate 0.7 g (52% yield) of a white solid in >98% purity (GC), identified as *N*-cycloheptyl-3,5-dinitrobenzamide (**S11**). ^{1}H-NMR (400 MHz, $CDCl_3$) δ, ppm: 9.18 (t, 1H), 8.94 (d, 2H), 6.19 (s, 1H), 4.22 (s, 1H), 2.11 (t, 2H), 1.78–1.68 (m, 4H), 1.62 (dd, 6H).^{13}C{^{1}H}-NMR (400 MHz, $CDCl_3$) δ, ppm: 161.57, 148.68, 138.50, 127.07,

120.88, 52.04, 35.10, 27.94, 21.12. HRMS (QTOF) m/z calculated for HRMS (QTOF) m/z calculated for $C_{14}H_{17}N_3O_5$ $[M+Na]^+$ 330.1060, found 330.1060.

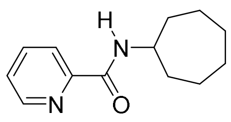

S12: The reaction of 0.5 g of cycloheptylamine (4.4 mmol), gave after flash chromatography over silica gel in DCM:MeOH (150:1) 0.67 g (69% yield) of a white solid in >99% purity (GC), identified as cycloheptyl 2-pyridinecarboxamide (**S12**). Spectroscopic data match those previously reported.[6] ^{1}H-NMR (400 MHz, $CDCl_3$) δ, ppm: 8.56 (d, 1H), 8.22 (d, 1H), 8.04 (s, 1H), 7.86 (td, 1H), 7.43 (ddd, 1H), 4.17 (ddt, 1H), 2.09–2.01 (m, 2H), 1.65 (ddt, 10H).

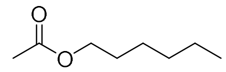

S21: The reaction of 1 g of 1-hexanol (9.8 mmol), gave after flash chromatography over silica gel in hexane-ethyl acetate (20:1) 1.2 g (83% yield) of a colorless liquid in >99% purity (GC), identified as 1-hexyl acetate (**S21**). Spectroscopic data match those previously reported.[7] ^{1}H-NMR (400 MHz, $CDCl_3$) δ, ppm: 0.89 (t, 3H), 1.28 – 1.37 (m, 6H), 1.60 – 1.65 (m, 2H), 2.05 (s, 3H), 4.07 (t, 2H).

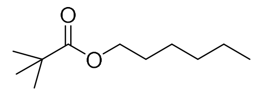

S22: The reaction of 1 g of 1-hexanol (9.8 mmol), gave after flash chromatography over silica gel in hexane-ethyl acetate (50:1) 1.5 g (82% yield) of a colorless liquid in >99% purity (GC), identified as 1-hexyl pivalate (**S22**). Spectroscopic data match those previously reported.[8] ^{1}H-NMR (400 MHz, $CDCl_3$) δ, ppm: 0.90 (t, 3H), 1.20 (s, 9H), 1.36 – 1.27 (m, 6H), 1.67 –1 .59 (m, 2H), 4.06 (t, 2H).

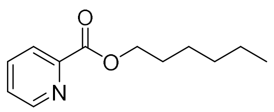

S23: The reaction of 1 g of 1-hexanol (9.8 mmol), gave after flash chromatography over silica gel in hexane-ethyl acetate (2:1) 3.5 g (80% yield) of a yellow pale liquid in >99% purity (GC), identified as 1-hexyl 2-pyridinecarboxylate (**S23**). ^{1}H-NMR (400 MHz, $CDCl_3$) δ, ppm: 8.80–8.76 (m, 1H), 8.14 (d, 1H), 7.85 (td, 1H), 7.48 (ddd, 1H), 4.43 (t, 2H), 1.85 (dd, 2H), 1.49 – 1.41 (m, 2H), 1.37 –1.31 (m, 4H), 0.91 (dd, 3H). $^{13}C\{^{1}H\}$-NMR (400 MHz, $CDCl_3$) δ, ppm: 165.26, 149.87, 148.30, 136.97, 126.76, 125.08, 66.13, 31.45, 28.65, 25.57, 22.53, 14.00. HRMS (QTOF) m/z calculated for $C_{13}H_{17}NO_2$ [M+Na]+ 230.1151, found 230.1155.

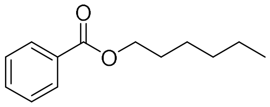

S24: The reaction of 1.5 g of 1-hexanol (14.7 mmol), gave after flash chromatography over silica gel in hexane-ethyl acetate (50:1) 2.2 g (73% yield) of a colorless liquid in >99% purity (GC), identified as 1-hexyl benzoate (**S24**). Spectroscopic data match those previously reported.[9] ^{1}H-NMR (400 MHz, $CDCl_3$) δ, ppm: 8.07 (dd, 2H), 7.58 (ddd, 1H), 7.46 (dd, 2H), 4.34 (t, 2H), 1.85 – 1.73 (m, 2H), 1.53 – 1.39 (m, 2H), 1.40 – 1.30 (m, 4H), 0.97 – 0.88 (m, 3H).

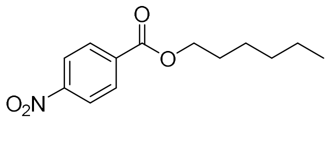

S25: The reaction of 1.5 g of 1-hexanol (14.7 mmol), gave after flash chromatography over silica gel in hexane-ethyl acetate (50:1) 2.5 g (70% yield) of a yellow pale liquid in >99% purity (GC), identified as 1-hexyl 4-nitrobenzoate (**S25**). Spectroscopic data match those previously reported.[9] ^{1}H-NMR (400 MHz, $CDCl_3$) δ, ppm: 8.34 – 8.29 (m, 2H), 8.25 – 8.20 (m, 2H), 4.39 (t, 2H), 1.86 – 1.77 (m, 2H), 1.52 – 1.42 (m, 2H), 1.40 – 1.32 (m, 4H), 0.96 – 0.90 (m, 3H).

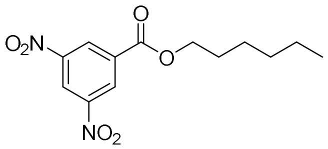

S26: The reaction of 1.5 g of 1-hexanol (14.7 mmol), gave after flash chromatography over silica gel in hexane-ethyl acetate (50:1) 3.5 g (80% yield) of a white solid in >99% purity (GC), identified as 1-hexyl 3,5-dinitrobenzoate (**S26**). Spectroscopic data match those previously reported.[10] ^{1}H-NMR (400 MHz, $CDCl_3$) δ, ppm: 9.22 (t, 1H), 9.16 (d, 2H), 4.45 (t, 2H), 1.83 (p, 2H), 1.51−1.40 (m, 2H), 1.43−1.30 (m, 4H), 0.98−0.85 (t, 3H).

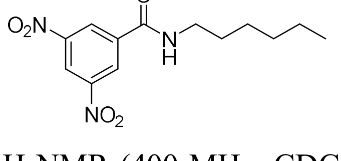

S29: The reaction of 0.5 g of hexylamine (4.9 mmol), gave after crystallization in ethyl acetate 0.8 g (55% yield) of a white solid in >98% purity (GC), identified as *N*-hexyl-3,5-dinitrobenzamide (**S29**). ^{1}H-NMR (400 MHz, $CDCl_3$) δ, ppm: 9.19 (t, 1H), 8.96 (d, 2H), 6.34 (s, 1H), 3.56 (dd, 2H), 1.69 (dd, 2H), 1.43 (dd, 2H), 1.37 (dt 4H), 0.93 (t, 3H). $^{13}C\{^{1}H\}$-NMR (400 MHz, $CDCl_3$) δ, ppm: 162.66, 148.67, 138.20, 127.06, 120.98, 40.86, 31.43, 29.44, 26.63, 22.54, 14.00. HRMS (QTOF) m/z calculated for $C_{13}H_{17}N_3O_5$ $[M+Na]^+$ 318.1060, found 318.1053.

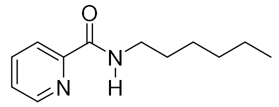

S30: The reaction of 0.5 g of hexylamine (4.9 mmol), gave after flash chromatography over silica gel in DCM:MeOH (150:1) 0.7 g (69% yield) of a white solid in >99% purity (GC), identified as *N*-hexyl 2-pyridinecarboxamide (**S30**). Spectroscopic data match those previously reported.[11] ^{1}H-NMR (400 MHz, $CDCl_3$) δ, ppm: 8.56 (ddd, *J* = 4.7, 1.6, 0.9 Hz, 1H), 8.25 – 8.19 (m, 1H), 8.07 (s, 1H), 7.86 (td, 1H), 7.43 (ddd, 1H), 3.49 (dd, 2H), 1.67 (dd, 2H), 1.45 – 1.31 (m, 6H), 0.96–0.85 (m, 3H).

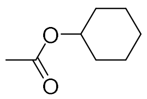

S38: The reaction of 1 g of cyclohexanol (10 mmol), gave after flash chromatography over silica gel in hexane-ethyl acetate (20:1) 1.1 g (77% yield) of a colorless liquid in >99% purity (GC), identified as cyclohexyl acetate (**S38**). Spectroscopic data match those previously reported.[12] ^{1}H-NMR (400 MHz, $CDCl_3$) δ, ppm: 4.71 – 4.76 (m, 1H), 2.03 (s, 3H), 1.84 – 1.87 (m, 2H), 1.70 – 1.75 (m, 2H), 1.52 – 1.57 (s, 2H), 1.32 – 1.44 (m, 3H), 1.21 − 1.28 (m, 1H).

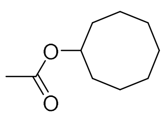

S39: The reaction of 1 g of cyclooctanol (7.8 mmol), gave after flash chromatography over silica gel in hexane-ethyl acetate (20:1) 0.98 g (74% yield) of a colorless liquid in >99% purity (GC), identified as cyclooctyl acetate (**S39**). Spectroscopic data match those previously reported.[12] ^{1}H-NMR (400 MHz, $CDCl_3$) δ, ppm: 4.92 (m, 1H). 2.03 (s, 3H), 1.85 – 1.47 (m, 14H).

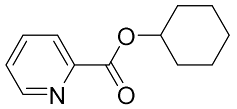

S40: The reaction of 0.5 g of cyclohexanol (6.0 mmol), gave after flash chromatography over silica gel in DCM:MeOH (150:1) 0.42 g (41% yield) of a yellow solid in >98% purity (GC), identified as cyclohexyl 2-pyridinecarboxylate (**S40**). ^{1}H-NMR (400 MHz, $CDCl_3$) δ, ppm: 8.80 (ddd, 1H), 8.14 (dt, 1H), 7.85 (td, 1H), 7.48 (ddd, 1H), 5.16 – 5.06 (m, 1H), 2.11 – 2.01 (m, 2H), 1.89 – 1.80 (m, 2H), 1.69 – 1.61 (m, 3H), 1.51 – 1.41 (m, 2H), 1.32 (tdd, 1H). $^{13}C\{^{1}H\}$-NMR (400 MHz, $CDCl_3$) δ, ppm: 164.54, 149.88, 148.67, 136.95, 126.63, 125.07, 74.58, 31.67, 25.36, 24.06. HRMS (QTOF) m/z calculated for $C_{12}H_{15}NO_2$ $[M+Na]^+$ 228.0995, found 228.0992.

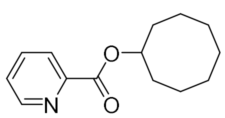

S41: The reaction of 2 g of cyclooctanol (15.6 mmol), gave after flash chromatography over silica gel in hexane-ethyl acetate (2:1) 2.7 g (74% yield) of an orange liquid in >98% purity (GC), identified as cyclooctyl 2-pyridinecarboxylate (**S41**). ^{1}H-NMR (400 MHz, $CDCl_3$) δ, ppm: 8.78 (ddd, 1H), 8.12 (dt, 1H), 7.84 (td, 1H), 7.46 (ddd, 1H), 5.33 – 5.25 (m, 1H), 2.02 – 1.91 (m, 4H), 1.85 – 1.75 (m, 2H), 1.68 – 1.51 (m, 8H). ^{13}C{^{1}H}-NMR (400 MHz, $CDCl_3$) δ, ppm: 164.50, 149.88, 148.83, 136.88, 126.57, 124.99, 77.09, 31.75, 27.01, 25.45, 23.13. HRMS (QTOF) m/z calculated for $C_{14}H_{19}NO_2$ $[M+Na]^+$ 256.1308, found 256.1300.

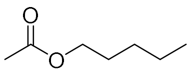

S44: The reaction of 1 g of 1-pentanol (11.4 mmol), gave after flash chromatography over silica gel in hexane-ethyl acetate (25:1) 1.2 g (81% yield) of a colorless liquid in >99% purity (GC), identified as 1-pentyl acetate (**S44**). Spectroscopic data match those previously reported.[13] ^{1}H-NMR (400 MHz, $CDCl_3$) δ, ppm: 4.01 (t, 2H), 2.00 (s, 3H), 1.57 – 1.60 (m, 2H), 1.27 – 1.31 (m, 4H), 0.87 (t, 3H)

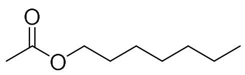

S45: The reaction of 1 g of 1-heptanol (8.6 mmol), gave after flash chromatography over silica gel in hexane-ethyl acetate (20:1) 1.1 g (81% yield) of a colorless liquid in >99% purity (GC), identified as 1-heptyl acetate (**S45**). Spectroscopic data match those previously reported.[14] ^{1}H-NMR (400 MHz, $CDCl_3$) δ, ppm: 4.03 (t, 2H), 2.02 (s, 3H), 1.60 (m, 2H), 1.35–1.23 (m, 8H), 0.86 (t, 3H).

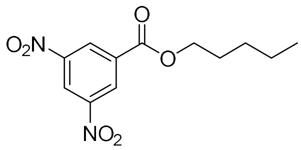

S46: The reaction of 0.5 g of 1-pentanol (5.7 mmol), gave after flash chromatography over silica gel in hexane-ethyl acetate (15:1) 0.79 g (49% yield) of a white solid in >99% purity (GC), identified as 1-pentyl 3,5-dinitrobenzoate (**S46**) Spectroscopic data match those previously reported.[10] ^{1}H-NMR (400 MHz, $CDCl_3$) δ, ppm: 9.25 (t, 1H), 9.18 (d, 2H), 4.48 (t, 2H), 1.91–1.82 (m, 2H), 1.49–1.40 (m, 4H), 0.98 (t, 3H).

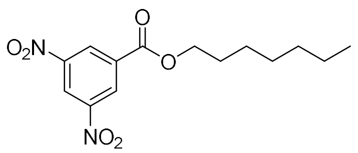

S47: The reaction of 0.5 g of 1-heptanol (4.3 mmol), gave after flash chromatography over silica gel in hexane-ethyl acetate (15:1) 0.85 g (64% yield) of a white solid in >99% purity (GC), identified as 1-heptyl 3,5-dinitrobenzoate (**S47**). ^{1}H-NMR (400 MHz, $CDCl_3$) δ, ppm: 9.25 (t, 1H), 9.18 (d, 2H), 4.48 (t, 2H), 1.91–1.82 (m, 2H), 1.49–1.40 (m, 4H), 0.98 (t, 3H).^{13}C{^{1}H}-NMR (400 MHz, $CDCl_3$) δ, ppm: 162.55, 148.68, 134.19, 129.41, 122.29, 67.16, 31.67, 28.88, 28.55, 25.85, 22.57, 14.05. HRMS (QTOF) m/z calculated for $C_{14}H_{18}N_2O_6$ $[M-H]^-$ 309.1092, found 309.1079.

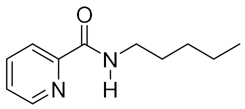

S50: The reaction of 0.5 g of pentylamine (5.74 mmol), gave after flash chromatography over silica gel in DCM:MeOH (100:1) 0.74 g (67% yield) of a yellow liquid in >99% purity (GC) identified as *N*-pentyl 2-pyridinecarboxamide (**S50**). ^{1}H-NMR (400 MHz, $CDCl_3$) δ, ppm: 8.58–8.55 (m, 1H), 8.22 (d, 1H), 8.07 (s, 1H), 7.86 (td, 1H), 7.43 (ddd, 1H), 3.49 (dd, 2H), 1.69–1.62 (m, 2H), 1.43–1.36 (m, 4H), 0.96–0.90 (m, 3H). ^{13}C{^{1}H}-NMR (400 MHz, $CDCl_3$) δ, ppm: 164.19, 150.11, 147.98, 137.31, 126.00, 122.18, 39.44, 29.37, 29.16, 22.40, 13.99. HRMS (QTOF) m/z calculated for $C_{11}H_{16}N_2O$ $[M+Na]^+$ 215.1155, found 215.1149.

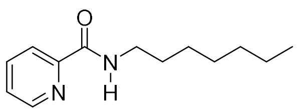

S51: The reaction of 0.5 g of heptylamine (4.34 mmol), gave after flash chromatography over silica gel in DCM:MeOH (100:1) 0.61 g (64% yield) of a yellow liquid in >99% purity (GC), identified as *N*-heptyl 2-pyridinecarboxamide (**S51**). ^{1}H-NMR (400 MHz, $CDCl_3$) δ, ppm: 8.57 (d, 1H), 8.22 (d, 1H), 8.07 (s, 1H), 7.86 (td, 1H), 7.44 (ddd, 1H), 3.49 (dd, 2H), 1.69–1.64 (m, 2H), 1.45–1.29 (m, 8H), 0.90 (t, 3H). ^{13}C{^{1}H}-NMR (400 MHz, $CDCl_3$) δ, ppm: 164.19, 150.12, 147.98, 137.31, 125.99, 122.18, 39.48, 31.75, 29.68, 29.01, 26.99, 22.61, 14.07. HRMS (QTOF) m/z calculated for $C_{13}H_{20}N_2O$ $[M+Na]^+$ 243.1468, found 243.1473.

2.3. Synthesis of the sulfonamide substrates

The sulfonamide substrates (**S14-S16**, **S32-S34**, **S42**, **S43**, **S48**, **S49**) were prepared according to a slight modification of a reported procedure (**Scheme S3**).[15]

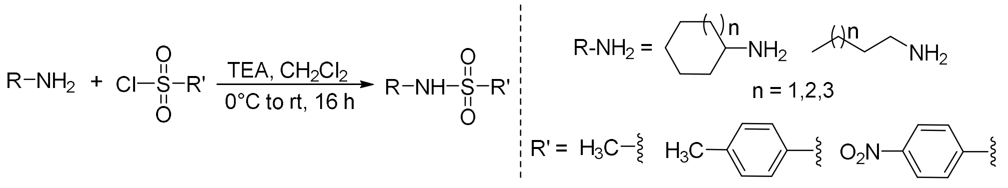

Scheme S3. Synthesis of the sulfonamide substrates.

In a 50 mL round-bottom flask equipped with a septum and kept under nitrogen the amine substrate (1.0 eq) and CH_2Cl_2 (1.0 M) were added. Next, triethylamine (1.2 eq) was added to the solution. The reaction was cooled to 0 °C and the corresponding sulfonyl chloride (1.1 eq) was added portion-wise over 10 minutes under magnetic stirring. The reaction was warmed to room temperature and stirred overnight. After complete substrate conversion (monitored by TLC or GC analysis), the reaction was washed with water (15 mL) and the aqueous phase was then separated and extracted with CH_2Cl_2 (2x15 mL) The combined organic extracts were washed with 2M HCl (2x30 mL), brine (2x30 mL) and then dried over $MgSO_4$. After filtration, the solution was concentrated under vacuum and the crude was purified by flash chromatography on silica gel (see below for purification conditions and characterization).

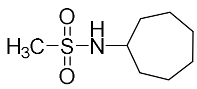

S14: The reaction of 0.5 g of cycloheptylamine (4.4 mmol), gave after flash chromatography over silica gel in hexane-ethyl acetate (2:1) 0.72 g (86% yield) of a pale yellow oil in >99% purity (GC), identified as *N*-cycloheptylmethanesulfonamide (**S14**). ^{1}H-NMR (400 MHz, $CDCl_3$) δ, ppm: 4.24 (d, 1H), 3.56 (ddq, 1H), 3,01 (s, 3H), 2.07–1.97 (m, 2H), 1.67–1.46 (m, 11H), ^{13}C{^{1}H}-NMR (400 MHz, $CDCl_3$) δ, ppm: 55.11, 41.80, 36.47, 27.86, 23.54. HRMS (QTOF) m/z calculated for $C_8H_{17}NO_2S$ $[M+Na]^+$ 214.0872, found 214.0870.

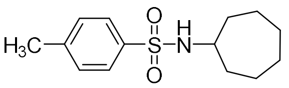

S15: The reaction of 1 g of cycloheptylamine (8.8 mmol), gave after flash chromatography over silica gel in hexane-ethyl acetate (5:1) 2 g (85% yield) of a pale yellow solid in >99% purity (GC), identified as *N*-cycloheptyl-4-methylbenzenesulfonamide (**S15**). Spectroscopic data match those previously reported.[15] ^{1}H-NMR (400 MHz, $CDCl_3$) δ, ppm: 7.75 (d, 2H), 7.29 (d,2H), 4.31 (d,1H), 3.35 (m, 1H), 2.45 (s, 3H), 1.81-1.75 (m, 2H), 1.56-1.33 (m, 10H).

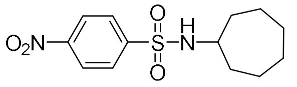

S16: The reaction of 0.5 g of cycloheptylamine (4.4 mmol), gave after flash chromatography over silica gel in hexane-ethyl acetate (3:1) 0.89 g (68% yield) of a pale yellow solid in >99% purity (GC), identified as *N*-cycloheptyl-4-nitrobenzenesulfonamide (**S16**). ^{1}H-NMR (400 MHz, $CDCl_3$) δ, ppm: 8.41–8.35 (m, 2H), 8.11–8.06 (m, 2H), 4.61 (d, 1H), 3.46 (qt, 1H), 1.86–1.78 (m, 2H), 1.58–1.35 (m, 10H). ^{13}C{^{1}H}-NMR (400 MHz, $CDCl_3$) δ, ppm: 149.93, 147.27, 128.14, 124.37, 55.36, 36.02, 27.80, 23.44. HRMS (QTOF) m/z calculated for $C_{13}H_{18}N_2O_4S$ $[M+Na]^+$ 321.0879, found 321.0880.

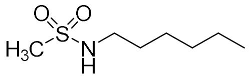

S32: The reaction of 1 g of hexylamine (9.9 mmol), gave after flash chromatography over silica gel in hexane-ethyl acetate (2:1) 1.5 g (84% yield) of a pale yellow liquid in >99% purity (GC), identified as *N*-hexylmethanesulfonamide (**S32**). Spectroscopic data match those previously reported.[16] ^{1}H-NMR (400 MHz, $CDCl_3$) δ, ppm: 4.64 (s, 1H), 3.15–3.09 (m, 2H), 2.96 (s, 3H), 1.59–1.53 (m, 2H), 1.31 (s, 6H), 0.89 (t, 3H).

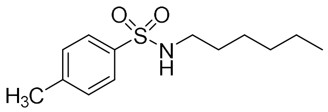

S32: The reaction of 1 g of hexylamine (9.9 mmol), gave after flash chromatography over silica gel in hexane-ethyl acetate (5:1) 2.1 g (83% yield) of a pale yellow solid in >99% purity (GC), identified as *N*-hexyl-4-methylbenzenesulfonamide (**S32**). Spectroscopic data match those previously reported.[17] ^{1}H-NMR (400 MHz, $CDCl_3$) δ, ppm: 7.76 (d, 2H), 7.31 (d, 2H), 4.71 (t, 1H), 2.92 (q, 2H), 2.43 (s, 3H), 1.20–1.47 (m, 8H) 0.84 (t, 3H).

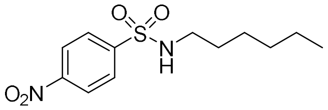

S33: The reaction of 1 g of hexylamine (9.9 mmol), gave after flash chromatography over silica gel in hexane-ethyl acetate (3:1) 2.2 g (78% yield) of a pale yellow solid in >99% purity (GC), identified as *N*-hexyl-4-nitrobenzenesulfonamide (**S33**). Spectroscopic data match those previously reported.[18] ^{1}H-NMR (400 MHz, $CDCl_3$) δ, ppm: 8.37 (d, 2H), 8.05 (d, 2H), 4.51 (s, 1H), 3.02 (td, 2H), 1.52–1.43 (m, 2H), 1.31–1.16 (m, 6H), 0.85 (t, 3H).

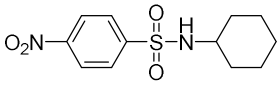

S42: The reaction of 0.5 g of cyclohexylamine (5.0 mmol), gave after flash chromatography over silica gel in hexane-ethyl acetate (4:1) 1.2 g (84% yield) of a pale yellow solid in >99% purity (GC), identified as *N*-cyclohexyl-4-nitrobenzenesulfonamide (**S42**). Spectroscopic data match those previously reported.[19] ^{1}H-NMR (400 MHz, $CDCl_3$) δ, ppm: 8.31–8.28 (d, 2H), 8.06–8.02 (d, 2H), 4.75 (d, 1H), 3.15–3.11 (m, 1H), 1.70–1.58 (m, 4H), 1.26–1.08 (m, 4H), 1.05–1.03 (m, 2H) 1H NMR (400 MHz, CDCl3) δ 2.32 – 2.22 (m, 1H), 1.78 (dd, J = 14.5, 7.6 Hz, 1H), 1.35 – 1.15 (m, 2H).

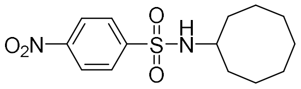

S43: The reaction of 2 g of cyclooctylamine (15.7 mmol), gave after flash chromatography over silica gel in hexane-ethyl acetate (5:1) 3.1 g (63% yield) of a pale yellow solid in >99% purity (GC), identified as *N*-cyclooctyl-4-nitrobenzenesulfonamide (**S43**). ^{1}H-NMR (400 MHz, $CDCl_3$) δ, ppm: 8.41–8.36 (m, 2H), 8.11–8.06 (m, 2H), 4.60 (s, 1H), 3.51 (ddt, 1H), 1.80–1.70 (m, 2H), 1.58–1.48 (m, 9H), 1.47–1.36 (m, 3H). $^{13}C\{^1H\}$-NMR (400 MHz, $CDCl_3$) δ, ppm: 149.92, 147.30, 128.14, 124.36, 54.46, 32.77, 27.11, 25.16, 23.09. HRMS (QTOF) m/z calculated for $C_{14}H_{20}N_2O_4S$ $[M+Na]^+$ 335.1036, found 335.1035.

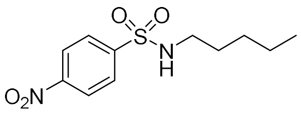

S48: The reaction of 1 g of pentylamine (11.5 mmol), gave after flash chromatography over silica gel in hexane-ethyl acetate (3:1) 2.1 g (67% yield) of a pale yellow solid in >99% purity (GC), identified as *N*-pentyl-4-nitrobenzenesulfonamide (**S48**). Spectroscopic data match those previously reported.[20] ^{1}H-NMR (400 MHz, $CDCl_3$) δ, ppm: 8.41-8.37 (d, 2H), 8.31-8.27 (d, 2H), 2.29 (t, 2H), 1.55–1.62 (m, 2H), 1.20–1.33 (m, 4H), 0.87 (t, 3H).

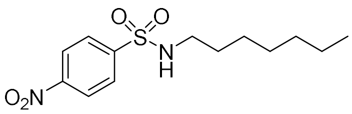

S49: The reaction of 1 g of heptylamine (8.7 mmol), gave after flash chromatography over silica gel in hexane-ethyl acetate (3:1) 1.3 g (50% yield) of a pale yellow solid in >99% purity (GC), identified as *N*-heptyl-4-nitrobenzenesulfonamide (**S49**). Spectroscopic data match those previously reported.[20] ^{1}H-NMR (400 MHz, $CDCl_3$) δ, ppm: 8.42–8.34 (m, 2H), 8.11–8.05 (m, 2H), 4.63 (t, 1H), 3.04 (dd, 2H), 1.55–1.45 (m, 2H), 1.26 (dd, 8H), 0.88 (t, 3H).

2.4. Synthesis of (phenylsulfonyl)cycloheptane (S19) and (phenylsulfonyl)hexane (S37).

(phenylsulfonyl)cycloheptane (**S19**) and (phenylsulfonyl)hexane (**S37**) have been synthetized over two steps according to slight modifications of reported procedures (**Scheme S4**).[21,10]

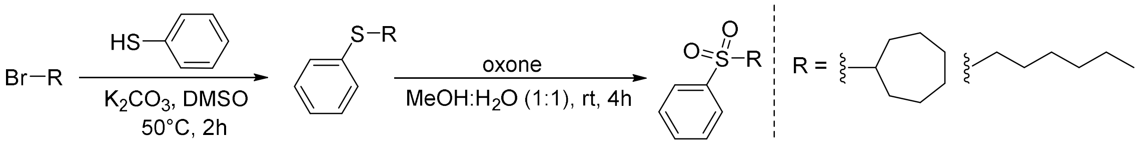

Scheme S4. Synthesis of the sulfone substrates.

2.4.1. Synthesis of the sulfides

In a 100 mL two necked round bottom flask equipped with a condenser system thiophenol (1 eq) and DMSO (0.7 M) were added. Next, K_2CO_3 (1.5 eq.) and alkyl bromide substrate (bromocycloheptane or 1-bromohexane, 1.0 eq.) were added. After 10 min at room temperature, the reaction mixture was heated to 50°C and kept under stirring for 2h. After complete substrate conversion (monitored by TLC or GC analysis), Et_2O was added (25 mL) and the organic phase was extracted with water (3x50 mL). Then the organic extract were dried over $MgSO_4$. After filtration, the solution was concentrated under vacuum and the crude compound was used for the next step without further purifications.

2.4.2. Synthesis of the sulfones

In a 250 mL round bottom flask the sulfide substrate (cycloheptyl(phenyl)sulfide or hexyl(phenyl)sulfide 1.0 eq) was dissolved in 1:1 methanol:water (0.2 M)). Oxone (3 eq) was added and the reaction was stirred at room temperature for 4 hours. After complete substrate conversion (monitored by TLC or GC analysis), the crude reaction mixture was concentrated under vacuum to remove the methanol and then extracted with CH_2Cl_2 (3x30 mL). The combined organic layers were dried over $MgSO_4$, filtrated and concentrated under vacuum. The crude compound was then purified by flash chromatography on silica gel (see below for purification conditions and characterization).

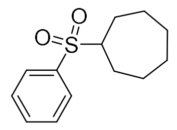

S19: The reaction of 3 g of bromocycloheptane (16.9 mmol), gave after flash chromatography over silica gel in hexane-ethyl acetate (10:1) 1.5 g (37% yield) of a white solid in >99% purity (GC), identified as (phenylsulfonyl)cycloheptane (**S19**). ^{1}H-NMR (400 MHz, $CDCl_3$) δ, ppm: 7.92–7.88 (m, 2H), 7.66 (ddd, 1H), 7.58 (t, 2H), 3.12–3.03 (m, 1H), 2.17 (dtd, 2H), 1.78 (ddd, 2H), 1.69 (ddd, 2H), 1.57–1.41 (m, 6H). $^{13}C\{^1H\}$-NMR (400 MHz, $CDCl_3$) δ, ppm: 137.81, 133.43, 129.01, 128.95, 65.24, 28.00, 27.22, 25.77. HRMS (QTOF) m/z calculated for $C_{13}H_{18}O_2S$ $[M+Na]^+$ 261.0920, found 261.0920.

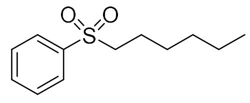

S37: The reaction of 3 g of 1-bromohexane (18.2 mmol), gave after flash chromatography over silica gel in hexane-ethyl acetate (15:1) 1.7 g (41% yield) of a colorless oil in >99% purity (GC), identified as (phenylsulfonyl)hexane (**S37**). Spectroscopic data match those previously reported.[22] ^{1}H-NMR (400 MHz, $CDCl_3$) δ, ppm: 7.93-7.86 (m, 2H), 7.68-7.61 (m, 1H), 7.60-7.52 (m, 2H), 3.13-3.01 (m, 2H), 1.77-1.60 (m, 2H), 1.38-1.29 (m, 2H), 1.29-1.18 (m, 4H), 0.84 (t, 3H).

2.5. Synthesis of *N*-cycloheptyl-2,2,2-trifluoroacetamide (S10) and *N*-hexyl-2,2,2-trifluoro acetamide (S28)

N-cycloheptyl-2,2,2-trifluoroacetamide (**S10**) and *N*-hexyl-2,2,2-trifluoro acetamide (**S28**) were prepared according to a slight modification of a reported procedure (**Scheme S5**).[23]

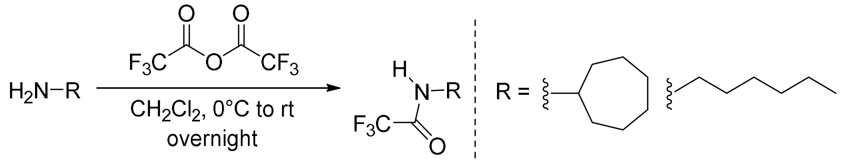

Scheme S5. Synthesis of the 2,2,2-trifluoroacetamide substrates.

In a 50 mL round-bottom flask equipped with a septum and kept under nitrogen the amine substrate (cycloheptylamine or 1-hexylamine 1.0 eq.) and CH_2Cl_2 (1 M) were added. Then the reaction was cooled to 0 °C and trifluoroacetic anhydride (1.0 eq.) was added dropwise over 10 minutes under magnetic stirring. The reaction was warmed to room temperature and stirred overnight. After complete substrate conversion (monitored by TLC or GC analysis), the reaction was washed with water (10 mL), then the aqueous phase was separated and extracted with CH_2Cl_2 (3x 10 mL). The combined organic extracts were washed with 2M HCl (2x 20 mL), saturated $NaHCO_3$ aqueous

solution (2x 20 mL) and then dried over $MgSO_4$. After filtration, the solution was concentrated under vacuum and the crude compound was purified by flash chromatography on silica gel (see below for purification conditions).

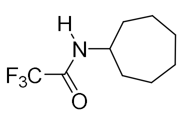

S10: The reaction of 1 g of cycloheptylamine (8.8 mmol), gave after flash chromatography over silica gel in hexane-ethyl acetate (1:1) 1.4 g (76% yield) of a yellow pale solid in >99% purity (GC), identified as *N*-cycloheptyl-2,2,2-trifluoroacetamide (**S10**) Spectroscopic data match those previously reported.[23] ^{1}H-NMR (400 MHz, $CDCl_3$) δ, ppm: 6.21 (bs, 1H), 3.98 (m, 1H), 2.06–1.88 (m, 2H), 1.73-1.41 (m, 10H).

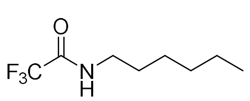

S28: The reaction of 1 g of hexylamine (9.9 mmol), gave after flash chromatography over silica gel in hexane-ethyl acetate (1:1) 2.2 g (78% yield) of a yellow pale oil in >99% purity (GC), identified as *N*-hexyl-2,2,2-trifluoroacetamide (**S28**). Spectroscopic data match those previously reported.[24] ^{1}H-NMR (400 MHz, $CDCl_3$) δ, ppm: 6.56 (bs, 1H), 3.34 (q, 2H), 1.57 (dt, 2H), 1.41–1.26 (m, 6H), 0.88 (t, 3H).

2.6. Synthesis of *N*-(*tert*-butyl)cycloheptanecarboxamide (S9) and *N*-(*tert*-butyl)heptanamide (S27)

N-(*tert*-butyl)cycloheptanecarboxamide (**S9**) and *N*-(*tert*-butyl)heptanamide (**S27**) were prepared according to a slight modification of a reported procedure (**Schema S6**).[25]

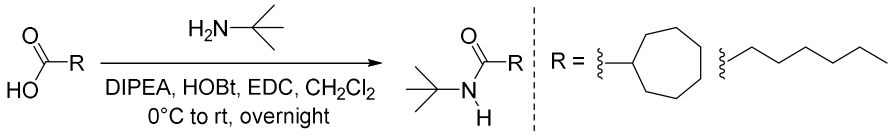

Scheme S6. Synthesis of *N*-*tert*-butyl amide substrates.

In a 250 mL round-bottom flask equipped with a septum and kept under nitrogen tertbutylamine (1.2 eq.) and CH_2Cl_2 (0.1 M) were added. with respect to the carboxylic acid). Then the reaction was cooled to 0 °C and sequentially DIPEA (1.2 eq.), carboxylic acid (cycloheptane carboxylic acid or heptanoic acid, 1.0 eq.), HOBt (1.2 eq.), and EDC•HCl (1.2 quiv) were added. The reaction mixture was warmed to rt and stirred overnight. After complete substrate conversion (monitored by TLC or GC analysis), the reaction mixture was washed with a saturated aqueous solution of $NaHCO_3$ (2x25 mL), an aqueous solution of citric acid (2x25 mL) , and with brine (2x 25 mL). Then the aqueous layer was extracted with CH_2Cl_2.(3x 100 mL) and the combined organic layers were then dried over $MgSO_4$. After filtration, the solution was concentrated under vacuum and the

crude compound was purified by flash chromatography on silica gel (see below for purification conditions and characterization).

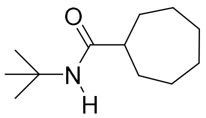

S9: The reaction of 1.0 g of cycloheptane carboxylic acid (7.0 mmol), gave after flash chromatography over silica gel in hexane-ethyl acetate (2:1) 0.97 g (70 % yield) of a white solid in >99% purity (GC), identified as *N*-(*tert*-butyl)cycloheptanecarboxamide (**S9**). Spectroscopic data match those previously reported.[26] ^{1}H-NMR (400 MHz, $CDCl_3$) δ, ppm: 5.16 (br s, 1H), 2.04–2.10 (m, 1H), 1.80−1.87 (m, 2H), 1.72−1.77 (m, 2H), 1.50−1.66 (m, 6H), 1.39−1.49 (m, 2H), 1.32 (m, 9H).

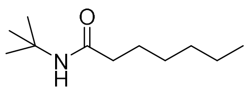

S27: The reaction of 1.0 g of hexanoic acid (8.6 mmol), gave after flash chromatography over silica gel in hexane-ethyl acetate (1:1) 0.75 g (47% yield) of a yellow pale oil in >98% purity (GC), identified as *N*-(*tert*-butyl)heptanamide (**S27**). Spectroscopic data match those previously reported.[27] ^{1}H-NMR (400 MHz, $CDCl_3$) δ, ppm: 5.16 (br s, 1H), 2.04–2.10 (m, 1H), 1.80−1.87 (m, 2H), 1.72−1.77 (m, 2H), 1.50−1.66 (m, 6H), 1.39−1.49 (m, 2H), 1.32 (m, 9H).

2.7. Synthesis of cycloheptanecarbonitrile (S17)

Cycloheptanecarbonitrile (**S17**) was prepared according to a slight modification of a reported procedure (**Scheme S7**).[28]

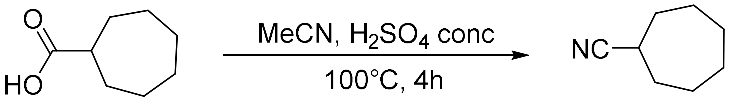

Scheme S7. Synthesis of cycloheptanecarbonitrile.

In a 100 mL two-necked round bottom flask equipped with a condenser system 1.0 g of cycloheptane carboxylic acid (7 mmol) 35 mL of acetonitrile (0.2 M), and 30 drops of concentrated H_2SO_4 were added. The reaction was stirred at 100 °C for 4 hours. After complete substrate conversion (monitored by TLC and GC), the solution was concentrated under vacuum. The crude residue was taken up in CH_2Cl_2 (50 mL), extracted two times with saturated aqueous $NaHCO_3$ (25 mL) and then dried over $MgSO_4$. After filtration, the solution was concentrated under vacuum and the crude was purified by flash chromatography on silica gel (hexane:AcOEt 5:1) to afford the title compound as a colorless oil (0,39 g, 3,15 mmol, 45% yield, >98% GC purity), identified as

cycloheptanecarbonitrile (**S17**). ^{1}H-NMR (400 MHz, $CDCl_3$) δ, ppm: 2.79 (tt, 1H), 1.96–1.82 (m, 4H), 1.80–1.70 (m, 2H), 1.67–1.53 (m, 6H). Spectroscopic data match those previously reported.[29]

2.8. Synthesis of *N*-cycloheptylphthalimide (S13) and *N*-hexylphthalimide (S31)

N-cycloheptylphthalimide (**S13**) and *N*-hexylphthalimide (**S31**) were prepared according to a slight modification of a reported procedure (**Scheme S8**).[30]

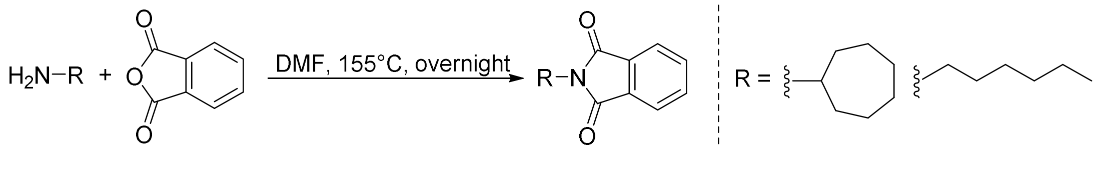

Scheme S8. Synthesis of imide substrates.

In a 50 mL two-necked round bottom flask equipped with a condenser system the amine substrate (cycloheptylamine or 1-hexylamine 1.0 eq.) and DMF (0.6 M) were added. Next, phthalic anhydride (1.5 eq) was added and the reaction mixture was heated to 155°C overnight. After complete substrate conversion (monitored by TLC and GC), the solution was cooled at rt, poured into 50 mL of 2M HCl, extracted with Et_2O (2x50 mL) and then dried over $MgSO_4$. After filtration, the solution was concentrated under vacuum and the crude was purified by flash chromatography on silica gel (see below for purification conditions and characterization).

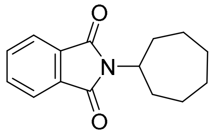

S13: The reaction of 1 g of cycloheptylamine (8.8 mmol), gave after flash chromatography over silica gel in hexane-ethyl acetate (10:1) 1.8 g (84% yield) of a white solid in >98% purity (GC), identified as *N*-cycloheptylphthalimide (**S13**). Spectroscopic data match those previously reported.[31] ^{1}H-NMR (400 MHz, $CDCl_3$) δ, ppm: 7.80 (dd, 2H), 7.68 (dd, 2H), 4.26 (tt, 1H), 2.29–2.22 (m, 2H), 1.84–1.78 (m, 4H), 1.69–1.59 (m, 4H), 1.54–1.48 (m, 2H).

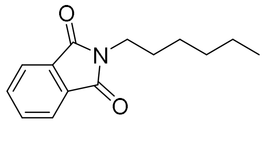

S31: The reaction of 1 g of hexylamine (9.9 mmol), gave after flash chromatography over silica gel in hexane-ethyl acetate (10:1) 1.9 g (83% yield) of a white solid in >98% purity (GC), identified as *N*-hexylphthalimide (**S31**). Spectroscopic data match those previously reported.[32] ^{1}H-NMR (400 MHz, $CDCl_3$) δ, ppm: 7.84–7.82 (m, 2H), 7.69–7.10 (m, 2H), 3.69–3.65 (m, 2H), 1.66 (d, 2H), 1.30 (s, 6H), 0.87 (d, 3H).

2.9. Synthesis of nitrocycloheptane (S18)

Nitrocycloheptane (**S18**) was prepared according to a slightly modified reported procedure (**Scheme S9**).[33]

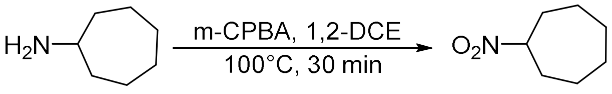

Scheme S9. Synthesis of nitrocycloheptane.

In a 100 mL tree-necked round bottom flask equipped with a condenser system 6.9 g of *m*-chloroperbenzoic acid (6.9 g, 40 mmol) and 15 mL of 1,2-dichloroethane (0.4 M) were added. Next, 1.13 g (10 mmol) of cycloheptylamine were added dropwise. The reaction was stirred at 100 °C for 30 minutes. After complete substrate conversion (monitored by TLC and GC), the reaction mixture was cooled, filtered, washed with 1 M NaOH (3x 25 mL) and then dried over $MgSO_4$. After filtration, the solution was concentrated under vacuum and the crude was purified by flash chromatography on silica gel (hexane:AcOEt 20:1) to afford the title compound as a yellow oil (0,32 g, 3,15 mmol, 22% yield, >98% GC purity), identified as nitrocycloheptane (**S18**). Spectroscopic data match those previously reported.[33] ^{1}H-NMR (400 MHz, $CDCl_3$) δ, ppm: 4.60 (tt, 1H), 2.30–2.20 (m, 2H), 2.17–2.06 (m, 2H), 1.87–1.78 (m, 2H), 1.65–1.59 (m, 4H), 1.56–1.47 (m, 2H).

2.10. Synthesis of 4-cyclohexylpyridine (S52)

4-Cyclohexylpiridine (**S52**) was prepared according to a slight modification of a reported procedure (**Scheme S10**).[34]

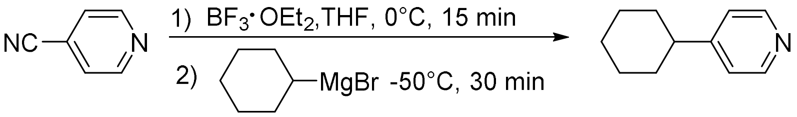

Scheme S10. Synthesis of 4-cyclohexylpiridine.

In a 25 mL round-bottom flask equipped with a septum and kept under nitrogen a solution of isonicotinonitrile (0.208 g, 2 mmol) in dry THF (4 mL) was added and cooled to 0 °C. Next, $BF_3{\cdot}OEt_2$ (312 mg, 2.2 mmol) was added dropwise and the mixture was stirred for 15 min. Then, the reaction mixture was cooled to -50 °C. A THF solution of cyclohexylmagnesium bromide (1.2 mmol) was added dropwise and the resulting mixture was stirred at the same temperature for 30 min. After this time, the reaction was quenched with 5 mL of a saturated NH_4Cl aqueous solution, extracted with ethyl acetate (3x 5mL) and then dried over $MgSO_4$. After filtration, the solution was concentrated under vacuum and the crude was purified by flash chromatography on silica gel

(hexane:AcOEt 3:1) to afford the title compound as a yellow oil (0.071 g, 0.44 mmol, 22% yield, >98% GC purity), identified as 4-cyclohexylpyridine (**S52**). Spectroscopic data match those previously reported.[34] ^{1}H-NMR (400 MHz, $CDCl_3$) δ, ppm: 8.46 (d, 2H), 7.10 (d, 2H), 2.54–2.41 (m, 1H), 1.95–1.76 (m, 4 H), 1.76–1.71 (m, 1H), 1.47–1.33 (m, 4H), 1.32–1.16 (m, 1H).

3. Synthesis of the manganese complexes

The complexes [Mn(^{TIPS}mcp)],[30] [Mn(^{TIPS}pdp)],[30] [Mn(^{dMM}pdp)],[35] and [Mn(^{Me2N}pdp)][35] ,were prepared according to the reported procedures (**Figure S1**).

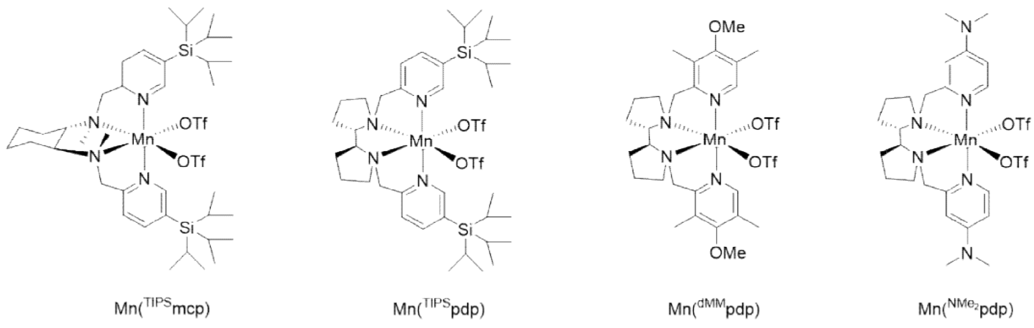

Figure S1. Structures of the manganese catalysts employed in this work.

4. Oxidation with H_2O_2 catalyzed by Mn complexes

4.1. General procedure

The following procedure was used for aliphatic C−H bond oxidations of substrates **S1-S52** with H_2O_2 catalyzed by Mn catalysts unless otherwise noted. A MeCN, HFIP (1,1,1,3,3,3-hexafluoro-2-propanol) or NFTBA (nonafluoro-*tert*-butyl alcohol) solution (400 μL, 0.125 M) of the substrate (50 μmol, 1.0 eq) and the Mn catalyst (0.5 μmol, 1 mol%) was prepared in a 10 mL vial equipped with a stirring bar and thermostated at $T = 0^{\circ}C$ (ice bath). The carboxylic acid (5 eq) was added directly to the solution. Then ~1.0 M H_2O_2 solution in the pertinent solvent was directly added by syringe pump over 30 minutes. At this point 0.5 eq of internal standard (biphenyl) was added and the solution was quickly filtered through a small silica or basic alumina plug, which was subsequently rinsed with 2 x 1 mL of EtOAc. GC analysis of the solution afforded the substrate conversions and product yields relative to the internal standard integration. Reaction products were identified by ^{1}H NMR analysis of the catalysis crude mixture.

For the oxidation reactions carried out in HFIP and NFTBA, the formation of a complex mixture of secondary alcohols and ketones was observed. In order to simplify product identification (GC and ^{1}H-NMR analysis) and quantitative GC analysis, by decreasing the number of products, thus preventing overlap between the GC peaks of isomeric and stereoisomeric alcohol products, the reaction mixture was subjected to follow-up oxidation with chromic acid, leading to the formation of a single carbonyl group for each oxidizable position.[36]

4.1.1. Chromic acid oxidation

To a stirring solution of the crude material in acetone (2.5 mL) at r.t., 0.5 mL of Jones reagent (prepared according to a reported procedure)[36] were added dropwise, leading to the formation of a characteristic orange solution. The reaction mixture was stirred at r.t. for 15 min. Then 2-propanol (1 mL) and a saturated aqueous solution of $NaHCO_3$ (3 mL) were added. The blue-green solid formed was filtered off through a medium porosity glass funnel and the filtrate was extracted with diethyl ether (2 × 5 mL). The combined organic phases were dried over $MgSO_4$, filtered, and concentrated at reduced pressure.

4.2. Optimization of the reaction conditions

Unless otherwise noted, the optimization experiments for the oxidation of methyl cycloheptane carboxylate (**S1**) were carried out following the general procedure reported above. Selectivities are expressed in terms of the ratio between major product and total product yield: **Pn-K4**/(**Pn-K3** + **Pn-K4**).

4.2.1. Optimization of S1 oxidation in MeCN

Table S1. Screening of the catalysts.[a]

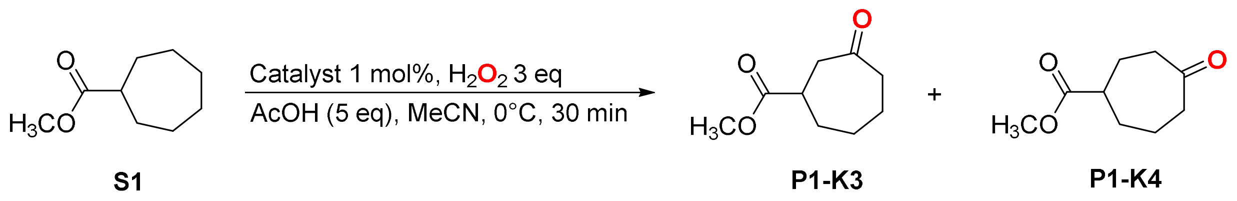

Entry	Catalyst	Conv./%	Total yield%	P1-K4:P1-K3	P1-K4 % selectivity
**1**	**Mn^{TIPS}mcp**	**68**	**60**	**1.6:1**	**62**
2	Mn^{TIPS}pdp	67	52	1.6:1	62
3	Mn^{DMM}mcp	73	53	1.3:1	57
4	Mn^{Me_2N}pdp	42	36	1.5:1	60

[a]Conversion and total yields were determined by GC and were averaged over at least two independent experiments.

Table S2. Screening of the carboxylic acid amount.[a]

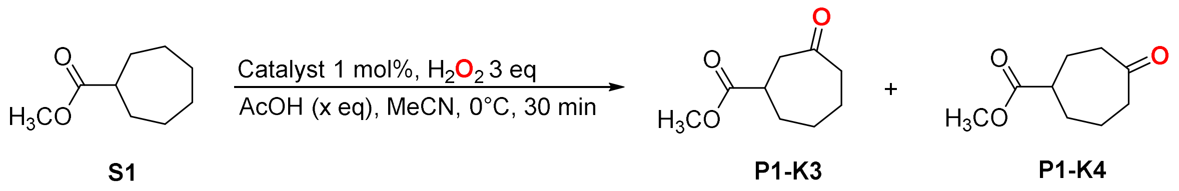

Entry	Amounth of acid	Conv./%	Total yield%	P1-K4:P1-K3	P1-K4% selectivity
1	2 eq	62	55	1.6:1	62
**2**	**5 eq**	**68**	**60**	**1.6:1**	**62**
3	10 eq	69	61	1.6:1	62

[a]Conversion and total yields were determined by GC and were averaged over at least two independent experiments.

Table S3. Screening of the carboxylic acids.[a]

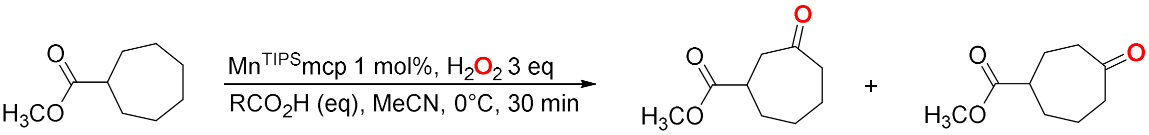

S1 **P1-K3** **P1-K4**

Entry	RCO_2H	Amounth of acid	Conv./%	Total yield%	P1-K4:P1-K3	P1-K4% selectivity
**1**	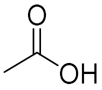	**5 eq**	**68**	**60**	**1.6:1**	**62**
2	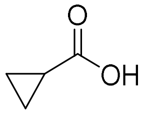	5 eq	78	65	1.4:1	58
3	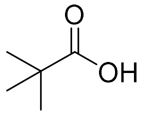	5 eq	75	65	1.4:1	58
4	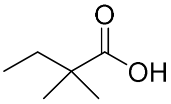	5 eq	60	53	1.5:1	60
5	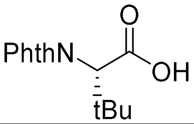	1 eq	70	58	1.5:1	60

[a]Conversion and total yields were determined by GC and were averaged over at least two independent experiments.

4.2.2. Optimization of S1 oxidation in fluorinated solvents (HFIP and NFTBA)

Table S4. Screening of the equivalents of H_2O_2 in HFIP.[a]

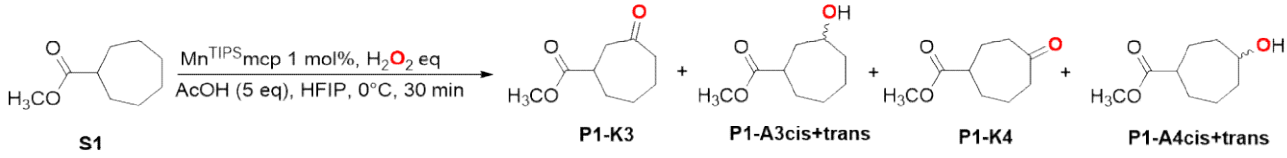

Entry	Amount of H_2O_2	Conv./%	Total yield%	%P1-K3	%P1-A3cis+trans	%P1-K4	%P1-A4cis+trans	C4:C3
1	1 eq	65	62	-	14.0	4.0	44.0	3.4:1
**2**	**2 eq**	**98**	**90**	**17.0**	**3.6**	**58.0**	**11.4**	**3.4:1**
3	3 eq	>99	91	21.0	-	70.0	-	3.3:1

[a]Conversion and total yields were determined by GC and were averaged over at least two independent experiments

Table S5. Screening of the equivalents of H_2O_2 in NFTBA.[a]

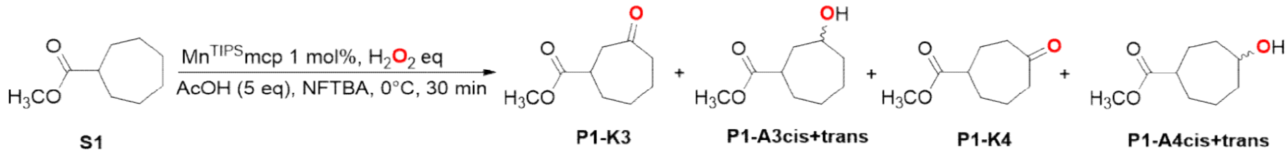

Entry	Amount of H_2O_2	Conv./%	Total yield%	%P1-K3	%P1-A3cis+trans	%P1-K4	%P1-A4cis+trans	C4:C3
1	1 eq	70	63	-	10.0	4.0	49.0	5.3:1
2[b]	2 eq	40	37	-	6.0	3.0	28.0	5.2:1
**3**	**2 eq**	**>99**	**94**	**12.0**	**3.0**	**58.0**	**21.0**	**5.3:1**
4	3 eq	>99	92	14.0	1.0	67.0	10.0	5.1:1

[a]Conversion and total yields were determined by GC and were averaged over at least two independent experiments.[b] Reaction carried out without the addition of carboxylic acid.

4.3. Results obtained in the oxidation of substrates S1-S52 in MeCN and in fluorinated solvents with H_2O_2 catalyzed by Mn(^{TIPS}mcp).

Table S6. Oxidation of cycloheptyl substrates **S1-S8**.[a]

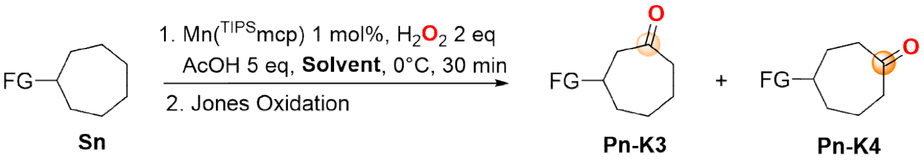

Esters	MeCN[b]	HFIP	NFTBA
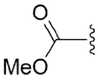 S1	60% yield (68% conv.) 62%; 1.6 : 1	90% yield (98% conv.) 77%; 3.4 : 1	94% yield (>99% conv.) 84%; 5.3 : 1
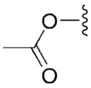 S2	70% yield (75% conv.) 72%; 2.6 : 1	81% yield (97% conv.) 81%; 4.3 : 1	85% yield (97% conv.) 85%; 5.5 : 1
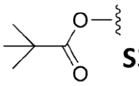 S3	75% yield (78% conv.) 72%; 2.6 : 1	91% yield (>99% conv.) 82%; 4.5 : 1	95% yield (97% conv.) 85%; 5.7 : 1
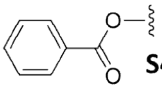 S4	66% yield (72% conv.) 67%; 2.0 : 1	No C–H oxidation[c]	97% yield (99% conv.) 77%; 3.4 : 1
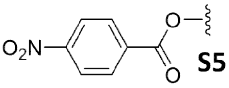 S5	80% yield (90% conv.) 76%; 3.8 : 1	99% yield (>99% conv.) 86%; 6.0 : 1	99% yield (>99% conv.) 91%; 9.7 : 1
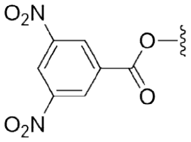 S6	83% yield (>99% conv.) 87%; 6.8 : 1	95% yield (>99% conv.) 98%; 48 : 1	88% yield (99% conv.) >98%; 59 : 1
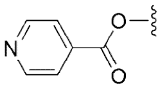 S7	No C–H oxidation[d]	76% yield (96% conv.) 96%; 22 : 1	95% yield (97% conv.) 93%; 13 : 1
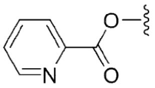 S8	70% yield (75% conv.) 73%; 2.7 : 1	70% yield (95% conv.) >99%; >99 : –[e]	97% yield (>99% conv.) 97%; 35 : 1

[a]Conversion and total yields were determined by GC and were averaged over at least two independent experiments.[b] Employing 3.0 eq of H_2O_2.[c] 0% conv. and yield. [d] Formation of the product derived by the oxidation of the pyridine nitrogen (**P7-Noxy**), (69% isolated yield).[e] Not detected.

Table S7. Oxidation of cycloheptyl substrates **S9-S12**.[a]

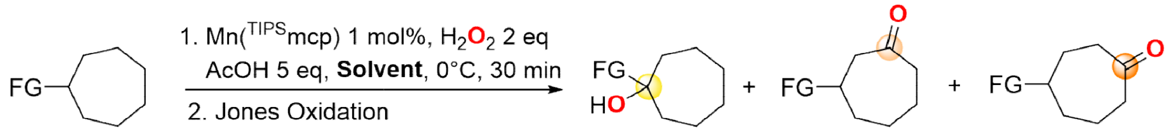

Sn **Pn-A1** **Pn-K3** **Pn-K4**

Amides	MeCN[b]	HFIP	NFTBA
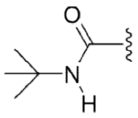 **S9**	51% yield (53% conv.); 55%; 1.2; 1	86% yield (>99% conv.); 72%; 2.6; 1	83% yield (94% conv.); 79%; 3.8; 1
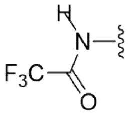 **S10**	78% yield (79% conv.); 62%; 1.6; 1	85% yield (>99% conv.); 75%; 3.0; 1	77% yield (85% conv.); 80%; 3.9; 1
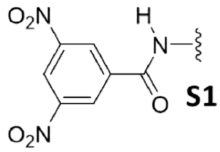 **S11**	No C–H oxidation[c]	82% yield (90% conv.); 80%; 3.9; 1	85% yield (95% conv.); 78%; 3.6; 1
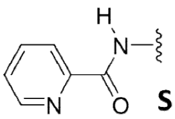 **S12**	27% yield (40% conv.); 36%; 1.0; 1.2; 1.1	98% yield (>99% conv.); 81%; 4.4; 1	89% yield (>99% conv.); 92%; 11.4; 1

[a]Conversion and total yields were determined by GC and were averaged over at least two independent experiments.[b] Employing 3.0 eq of H_2O_2.[c] 0% conv. and yield (since the compound was poorly soluble in MeCN, the reaction was carried out with 300 µl of MeCN+100 µl of CH_2Cl_2).

Table S8. Oxidation of cycloheptyl substrates **S13-S16**.[a]

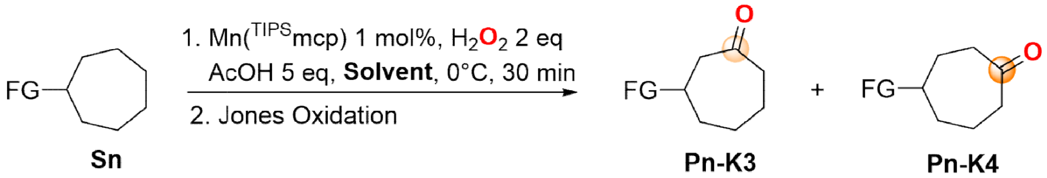

Imides and Sulfonamides	MeCN[b]	HFIP	NFTBA
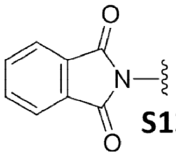 **S13**	47% yield (56% conv.); 66%; 1.9 : 1	80% yield (98% conv.); 80%; 4.0 : 1	95% yield (99% conv.); 82%; 4.5 : 1
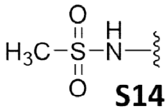 **S14**	16% yield (30% conv.); 81%; 4.3 : 1	46% yield (62% conv.); 82%; 4.5 : 1	42% yield (44% conv.); 88%; 7.1 : 1
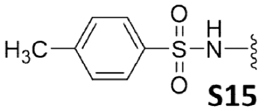 **S15**	64% yield (79% conv.); 80%; 4.0 : 1	59% yield (70% conv.); 89%; 7.8 : 1	75% yield (85% conv.); 93%; 14 : 1
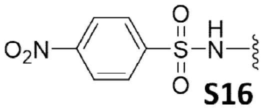 **S16**	51% yield (69% conv.); 83%; 4.9 : 1	87% yield (98% conv.); 91%; 10 : 1	89% yield (93% conv.); 93%; 14 : 1

[a]Conversion and total yields were determined by GC and were averaged over at least two independent experiments.[b] Employing 3.0 eq of H_2O_2.

Table S9. Oxidation of cycloheptyl substrates **S17-S19**.[a]

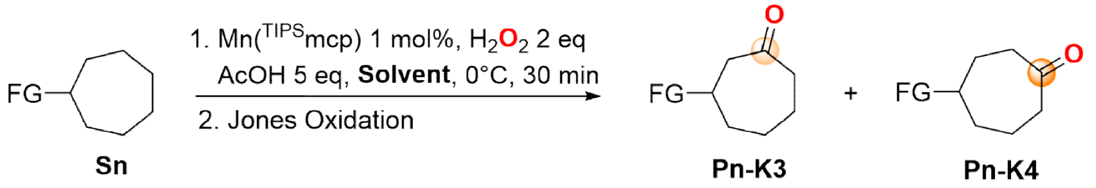

Other groups	MeCN[b]	HFIP	NFTBA
NC— **S17**	78% yield (79% conv.); 73%; 2.7 : 1	85% yield (99% conv.); 79%; 3.7 : 1	54% yield (63% conv.); 78%; 3.5 : 1
O_2N— **S18**	35% yield (50% conv.); 87%; 6.7 : 1	75% yield (90% conv.); 88%; 7.5 : 1	70% yield (90% conv.); 88%; 7.1 : 1
PhS(O)(O)— **S19**	80% yield (84% conv.); 74%; 2.9 : 1	90% yield (>99% conv.); 92%; 11 : 1	92% yield (99% conv.); 93%; 14 : 1

[a]Conversion and total yields were determined by GC and were averaged over at least two independent experiments.[b] Employing 3.0 eq of H_2O_2.

Table S10. Oxidation of 1-hexyl substrates **S20-S26**.[a]

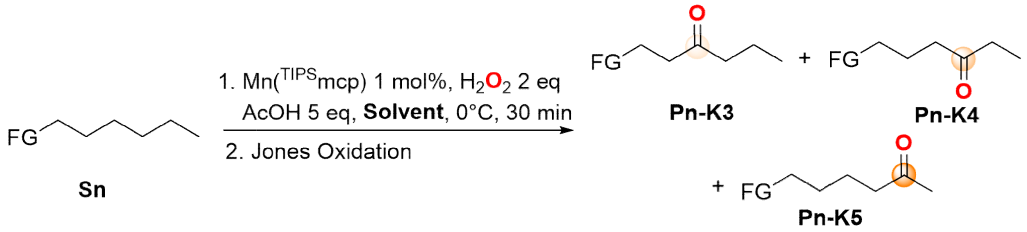

Esters	MeCN[b]	HFIP	NFTBA
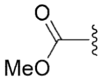 **S20**	45% yield (60% conv.)<br>62%<br>5.6<br>2.4<br>1	88% yield (>99% conv.)<br>80%<br>28<br>6.0<br>1	76% yield (80% conv.)<br>85%<br>41<br>6.5<br>1
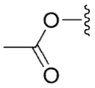 **S21**	57% yield (60% conv.)<br>66%<br>8.2<br>3.3<br>1	82% yield (>99% conv.)<br>82%<br>4.7<br>1	74% yield (89% conv.)<br>87%<br>6.7<br>1
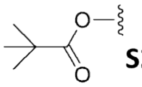 **S22**	55% yield (59% conv.)<br>64%<br>6.3<br>2.5<br>1	88% yield (>99% conv.)<br>83%<br>43<br>7.5<br>1	86% yield (94% conv.)<br>87%<br>6.7<br>1
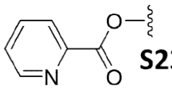 **S23**	6% yield (12% conv.)<br>78%<br>12<br>2.3<br>1	67% yield (81% conv.)<br>89%<br>8.0<br>1	57% yield (85% conv.)<br>88%<br>7.5<br>1
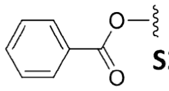 **S24**	59% yield (73% conv.)<br>64%<br>7.4<br>3.0<br>1	72% yield (98% conv.)<br>90%<br>9.0<br>1	75% yield (99% conv.)<br>90%<br>8.8<br>1
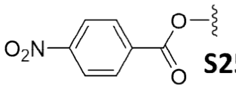 **S25**	61% yield (83% conv.)<br>67%<br>11.9<br>4.4<br>1	99% yield (>99% conv.)<br>92%<br>12.2<br>1	99% yield (>99% conv.)<br>92%<br>10.9<br>1
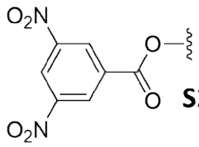 **S26**	38% yield (44% conv.)<br>71%<br>11.3<br>3.7<br>1	65% yield (75% conv.)<br>92%<br>11.8<br>1	88% yield (96% conv.)<br>93%<br>14<br>1

[a]Conversion and total yields were determined by GC and were averaged over at least two independent experiments.[b] Employing 3.0 eq of H_2O_2

Table S11. Oxidation of 1-hexyl substrates **S27-S30**.[a]

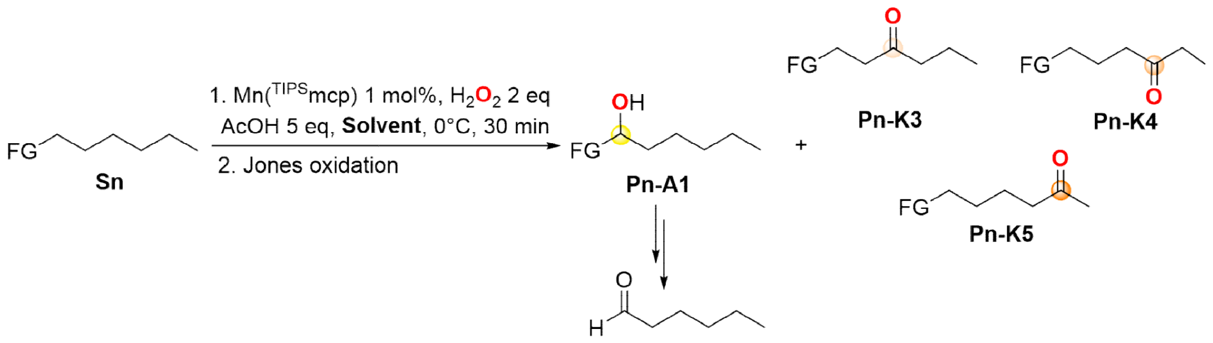

Amides	MeCN[b]	HFIP	NFTBA
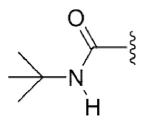 **S27**	36% yield (40% conv.); 57%; 3.4; 1.6; 1	91% yield (>99% conv.); 80%; 22; 4.5; 1	72% yield (88% conv.); 82%; 19; 3.3; 1
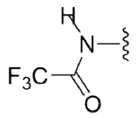 **S28**	55% yield (75% conv.); 53%; 2.1; 6.2; 2.4; 1	86% yield (99% conv.); 83%; 36; 6.3; 1	75% yield (87% conv.); 82%; 35; 6.5; 1
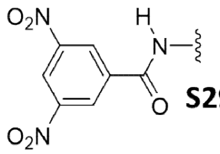 **S29**	No C–H oxidation[c]	65% yield (81% conv.); 82%; 4.6; 1	62% yield (70% conv.); 67%; 2.0; 1
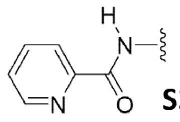 **S30**	25% yield (50% conv.); 11%; 17; 2.2; 1; -	81% yield (98% conv.); 87%; 6.7; 1	88% yield (90% conv.); 89%; 8.1; 1

[a]Conversion and total yields were determined by GC and were averaged over at least two independent experiments. [b] Employing 3.0 eq of H_2O_2 [c] 0% conv. and yield (the compound is not very soluble in MeCN, reaction carried out with 300 μl of MeCN+100 μl of CH_2Cl_2).

Table S12. Oxidation of 1-hexyl substrates **S31-S34**.[a]

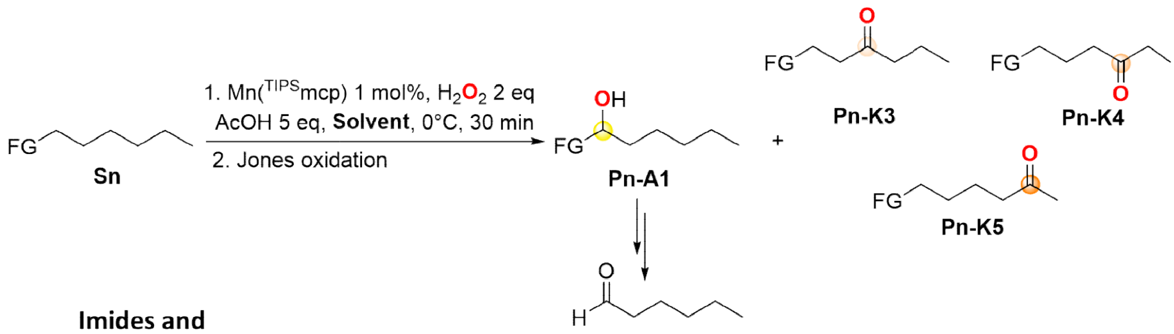

Imides and Sulfonamides	MeCN[b]	HFIP	NFTBA
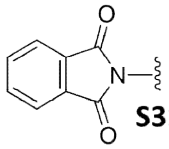 **S31**	33% yield (42% conv.); 62%; 1.6; 1	85% yield (>99% conv.); 81%; 4.2; 1	70% yield (76% conv.); 84%; 5.2; 1
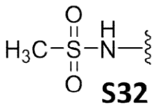 **S32**	26% yield (46% conv.); 29%; 10; 5.3; 2.0; 1	43% yield (60% conv.); 82%; 4.6; 1	30% yield (41% conv.); 86%; 6.4; 1
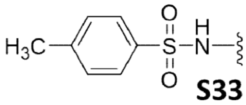 **S33**	42% yield (55% conv.); 34%; 11.7; 8.0; 3.1; 1	84% yield (97% conv.); 83%; 4.9; 1	78% yield (90% conv.); 86%; 5.9; 1
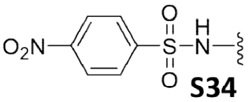 **S34**	42% yield (52% conv.); 71%; 9.7; 2.9; 1	87% yield (>99% conv.); 86%; 5.9; 1	82% yield (95% conv.); 88%; 7.0; 1

[a]Conversion and total yields were determined by GC and were averaged over at least two independent experiments.[b] Employing 3.0 eq of H_2O_2.

Table S13. Oxidation of 1-hexyl substrates **S35-S37**.[a]

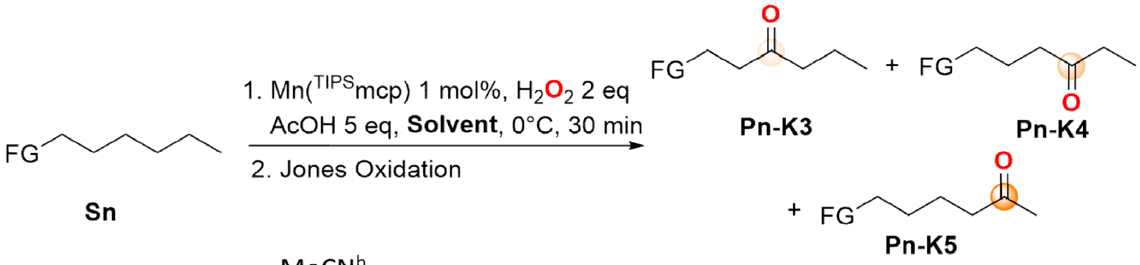

Other groups	MeCN[b]	HFIP	NFTBA
NC— **S35**	40% yield (55% conv.)<br>71%; 12; 3.8; 1	78% yield (94% conv.)<br>90%; 9.3; 1	59% yield (73% conv.)<br>88%; 7.6; 1
O_2N— **S36**	40% yield (47% conv.)<br>76%; 3.2; 1	70% yield (90% conv.)<br>91%; 9.6; 1	80% yield (92% conv.)<br>92%; 12; 1
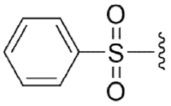 **S37**	45% yield (46% conv.)<br>74%; 11.5; 3.1; 1	99% yield (>99% conv.)<br>92%; 11.6; 1	90% yield (96% conv.)<br>93%; 13; 1

[a]Conversion and total yields were determined by GC and were averaged over at least two independent experiments.[b] Employing 3.0 eq of H_2O_2.

Table S14. Oxidation of cyclohexyl substrates **S38**, **S40**, **S42**, **S52**.[a]

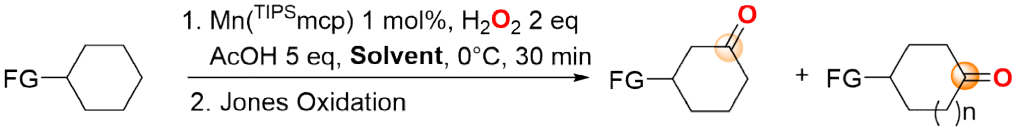

Sn **Pn-K3** **Pn-K4**

		MeCN[b]	HFIP	NFTBA
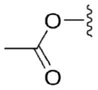	**S38**	69% yield (73% conv.); 86%; 6.0 : 1	81% yield (97% conv.); 94%; 15 : 1	90% yield (>99% conv.); 95%; 21 : 1
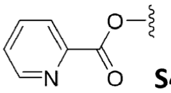	**S40**	33% yield (50% conv.); 78%; 3.6 : 1	81% yield (86% conv.); >99%; 99 : -[b]	68% yield (89% conv.); >99%; 99 : -[b]
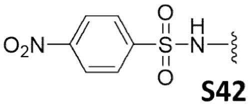	**S42**	30% yield (45% conv.); 91%; 10 : 1	50% yield (59% conv.); 97%; 30 : 1	52% yield (62% conv.); 98%; 44 : 1
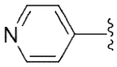	**S52**	No C–H oxidation[c]	55% yield (60% conv.); 57%; 1.3 : 1	52% yield (58% conv.); 63%; 1.7 : 1

[a]Conversion and total yields were determined by GC and were averaged over at least two independent experiments.[b] Employing 3.0 eq of H_2O_2[c] 0% conv. and yield.

Table S15. Oxidation of cyclooctyl substrates **S39**, **S41**, **S43**.[a]

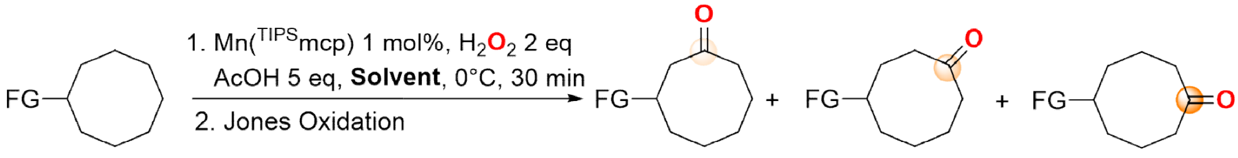

Sn **Pn-K3** **Pn-K4** **Pn-K5**

	MeCN[b]	HFIP	NFTBA
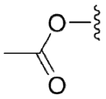 **S39**	78% yield (79% conv.); 43%; 2.6 : 2.5 : 1	85% yield (>99% conv.); 63%; 1.7 : 1	88% yield (>99% conv.); 60%; 1.5 : 1
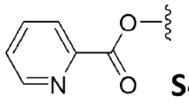 **S41**	35% yield (50% conv.); 47%; 3.4 : 2.8 : 1	82% yield (98% conv.); 63%; 1.7 : 1	99% yield (>99% conv.); 60%; 1.5 : 1
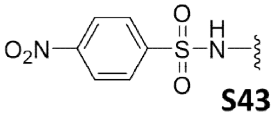 **S43**	80% yield (84% conv.); 72%; 2.6 : 1	75% yield (>99% conv.); 86%; 6.4 : 1	73% yield (>99% conv.); 90%; 9.4 : 1

[a]Conversion and total yields were determined by GC and were averaged over at least two independent experiments.[b] Employing 3.0 eq of H_2O_2.

Table S16. Oxidation of 1-pentyl substrates **S44**, **S46**, **S48**, **S50**.[a]

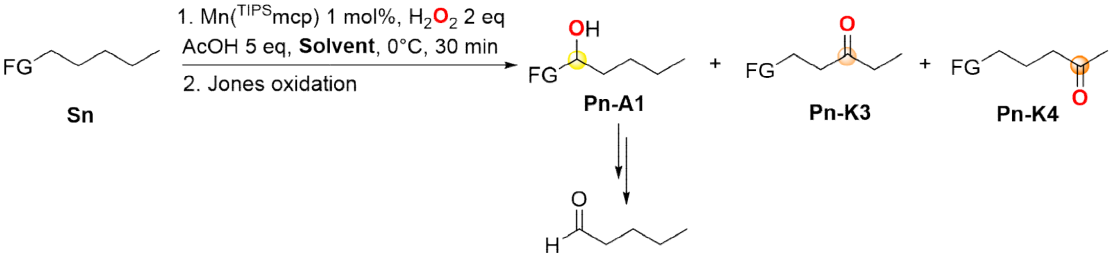

	MeCN[b]	HFIP	NFTBA
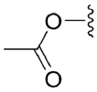 **S44**	37% yield (55% conv.)<br>78%<br>3.6<br>1	82% yield (90% conv.)<br>88%<br>7.1<br>1	39% yield (49% conv.)<br>90%<br>8.8<br>1
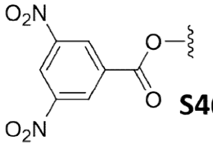 **S46**	30% yield (46% conv.)<br>93%<br>13<br>1	65% yield (90% conv.)<br>99%<br>82<br>1	40% yield (61% conv.)<br>>99%<br>>99<br>-[b]
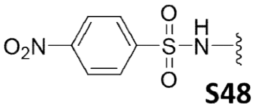 **S48**	20% yield (40% conv.)<br>75%<br>3.0<br>1	47% yield (60% conv.)<br>91%<br>9.7<br>1	31% yield (48% conv.)<br>87%<br>6.9<br>1
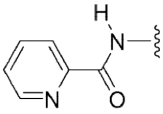 **S50**	25% yield (29% conv.)<br>7.5%<br>47<br>4.0<br>1	50% yield (75% conv.)<br>91%<br>9.6<br>1	40% yield (70% conv.)<br>95%<br>19.8<br>1

[a]Conversion and total yields were determined by GC and were averaged over at least two independent experiments.[b] Employing 3.0 eq of H_2O_2.

Table S17. Oxidation of 1-heptyl substrates **S45**, **S47**, **S49**, **S51**.[a]

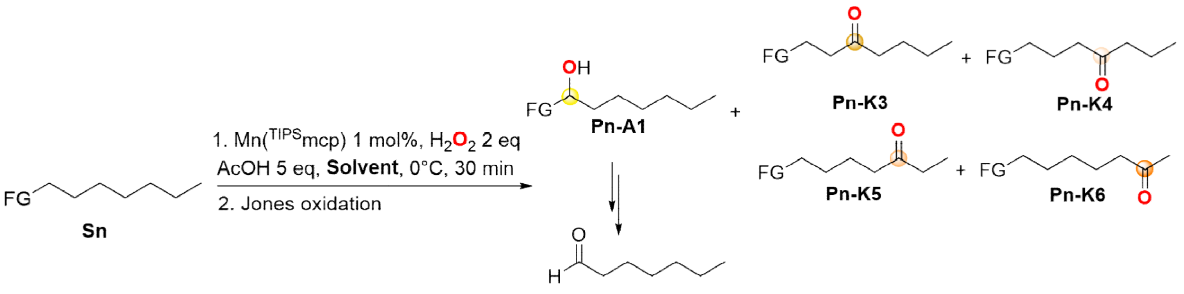

Substrate	MeCN[b]	HFIP	NFTBA
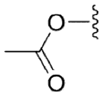 **S45**	61% yield (71% conv.); 55%; 10, 4.6, 2.9, 1	87% yield (>99% conv.); 76%; 14.9, 3.6, 1	98% yield (>99% conv.); 80%; 19.1, 3.9, 1
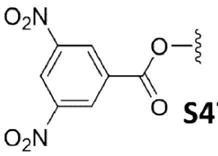 **S47**	44% yield (46% conv.); 67%; 199, 56, 41, 1	93% yield (>99% conv.); 85%; 35, 5.2, 1	96% yield (>99% conv.); 88%; 48, 5.4, 1
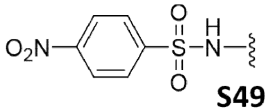 **S49**	45% yield (65% conv.); 57%; 12.5, 6.9, 1.5, 1	78% yield (95% conv.); 74%; 29, 9.0, 1	43% yield (59% conv.); 75%; 15, 4, 1
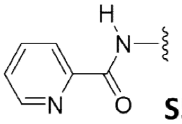 **S51**	5% yield (6% conv.); 99, -[c]	97% yield (99% conv.); 77%; 79, 17, 5.9, 1	82% yield (99% conv.); 86%; 30, 3.7, 1

[a]Conversion and total yields were determined by GC and were averaged over at least two independent experiments.[b] Employing 3.0 eq of H_2O_2[c] Not detected.

5. Isolation and characterization of the oxidation products

The products deriving from the oxidation of the substrates that gave the best results in terms of site selectivity have been isolated by scale-up oxidation of the respective substrates: **P8-K3** and **P8-K4** for the cycloheptyl substrates; **P26-K4** and **P26-K5** for the 1-hexyl substrates, **P40-K3, P40-K4** for the cyclohexyl substrates; **P43-K4**, **P43-K5** for the cyclooctyl substrates; **P46-K3**, **P46-K4** for the 1-pentyl substrates and **P47-K4**, **P47-K5** and **P47-K6** for the 1 heptyl substrates. The products deriving from the oxidation of the substrates used in the optimizations of each series of cylcloheptyl, 1-hexyl, cyclohexyl, cyclooctyl, 1-pentyl and 1-heptyl substrates were also isolated, namely: **P1-K3**, **P1-K4**, **P2-K3**, **P2-K4**, **P20-K3**, **P20-K4**, **P20-K5**, **P21-K4**, **P21-K5**, **P38-K4**, **P41-K3**,**P41-K4, P41-K5**, **P44-K3**, **P44-K4**, **P45-K5**, **P45-K6**, **P47-K4**, **P47-K5** and **P47-K6** respectively. The spectroscopic data of the isolated products were compared with those reported in the literature. Characterization of the unknown products was performed by ^{1}H-NMR, ^{13}C-NMR and HRMS.

The products deriving from the oxidation of the other substrates were identified and characterized by ^{1}H-NMR analysis of the catalysis crude mixture in MeCN, HFIP or NFTBA. Pentanal, hexanal and heptanal were identified by comparison with authentic samples.

5.1. Scale-up oxidation of S1, S2, S7, S8, S20, S21, S26, S38, S40, S41, S43-S47.

General procedure. A MeCN, HFIP or NFTBA 4 mL solution of substrate (1.0 eq, 0.125 M), Mn catalyst (1 mol%) was prepared in a 25 mL round bottom flask equipped with a magnetic stirring bar. Next, acetic acid (5 eq) were added and the resulting mixture was cooled at 0°C (ice bath). Then ~1.0 M H_2O_2 solution in the pertinent solvent (2.0 eq) was directly added by syringe pump over 30 minutes. Then the solvent was evaporated at reduced pressure and the crude was rinsed with 5 mL of CH_2Cl_2. The resulting organic solution was extracted two times with 2 x 5 mL saturated $NaHCO_3$ aqueous solution and one time with 5 mL of brine. The reunited organic phases were dried with MgSO4, filtered and then evaporated at reduced pressure. For the oxidation reactions performed in the fluorinated solvents, before the flash chromatographic on silica gel purification, the reaction mixture has been subjected to follow-up oxidation with chromic acid following the procedure reported above (**paragraph 4.1.1.**).

Reactions were performed on a 0.505 mmol scale.

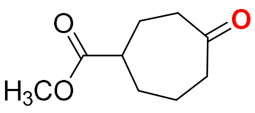

P1-K4

Prepared following the general procedure reported above. Conditions: 5 mg of Mn(^{TIPS}mcp) (0.00505 mmol, 1 mol%),79 mg of **S1** (0.505 mmol, 1.0 eq), 1.12 mL of 0.903 M H_2O_2 solution in NFTBA (1.01 mmol, 2 eq), 4 mL of NFTBA at 0 °C for 30 min. The crude obtained by scale up oxidation of **S1** was oxidized by chromic acid. The purification by flash chromatography over silica gel (hexane-EtOAc 5:1) afforded 55 mg of **P1-K4**, (0.32 mmol, 63% yield) as a colorless oil. Spectroscopic data match those previously reported.[4] ^{1}H-NMR (400 MHz, $CDCl_3$) δ, ppm: 1H NMR (400 MHz, CDCl3) δ 3.72 (s, 3H), 2.64 (ddd, J = 15.9, 7.3, 2.9 Hz, 1H), 2.59 – 2.51 (m, 3H), 2.18 – 2.06 (m, 2H), 1.88 –2.03 (m, 2H), 1.83 – 1.62 (m, 3H).

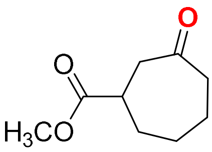

P1-K3

Prepared following the general procedure reported above. Conditions: 5 mg of Mn(^{TIPS}mcp) (0.00505 mmol, 1 mol%),79 mg of **S1** (0.505 mmol, 1.0 eq), 1.12 mL of 0.903 M H_2O_2 solution in NFTBA (1.01 mmol, 2 eq), 4 mL of NFTBA at 0 °C for 30 min. The crude obtained by scale up oxidation of **S1** was oxidized by chromic acid. The purification by flash chromatography over silica gel (hexane-EtOAc 5:1) afforded 10 mg of **P1-K3**, (0.059 mmol, 12% yield) as a colorless oil. Spectroscopic data match those previously reported.[4] ^{1}H-NMR (400 MHz, $CDCl_3$) δ, ppm: 3.72 (s, 3H), 2.88 – 2.68 (m, 3H), 2.61 – 2.45 (m, 2H), 2.16 – 2.09 (m, 1H), 2.00 – 1.77 (m, 3H), 1.69 (tddd, J = 12.2, 10.0, 4.0, 1.9 Hz, 1H), 1.57 – 1.51 (m, 1H).

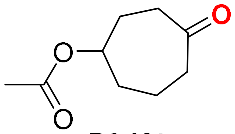

P2-K4

Prepared following the general procedure reported above. Conditions: 5 mg of Mn(^{TIPS}mcp) (0.00505 mmol, 1 mol%),79 mg of **S2** (0.505 mmol, 1.0 eq), 1.07 mL of 0.940 M H_2O_2 solution in NFTBA (1.01 mmol, 2 eq), 4 mL of NFTBA at 0 °C for 30 min. The crude obtained by scale up oxidation of **S2** was oxidized by chromic acid. The purification by flash chromatography over silica gel (hexane-EtOAc 5:1) afforded 62 mg of **P2-K4**, (0.36 mmol, 71% yield) as a colorless oil. Spectroscopic data match those previously reported. [37] ^{1}H-NMR (400 MHz, $CDCl_3$) δ, ppm: 4.93 (m, 1H), 2.58 (m, 1H), 2.46 (m, 2H), 2.39 (m, 1H), 2.05 (s, 3H), 1.92 – 1.82 (m, 4H), 1.80 – 1.73 (m, 1H), 1.67 – 1.58 (m, 1H).

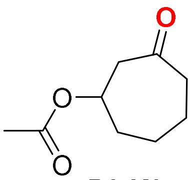

P2-K3

Prepared following the general procedure reported above. Conditions: 5 mg of Mn(^{TIPS}mcp) (0.00505 mmol, 1 mol%),79 mg of **S2** (0.505 mmol, 1.0 eq), 1.07 mL of 0.940 M H_2O_2 solution in NFTBA (1.01 mmol, 2 eq), 4 mL of NFTBA at 0 °C for 30 min. The crude obtained by scale up oxidation of **S2** was oxidized by chromic acid. The purification by flash chromatography over silica gel (hexane-EtOAc 5:1) afforded 11 mg of **P2-K3**, (0.065 mmol, 13% yield) as a colorless oil. Spectroscopic data match those previously reported.[38] ^{1}H-NMR (400 MHz, $CDCl_3$) δ, ppm: 5.12 (m, 1H), 2.78 (m, 2H), 2.49 (m, 2H), 2.07 (s, 3H), 1.95 (m, 1H), 1.85–1.80 (m, 2H), 1.74–1.56 (m, 3H).

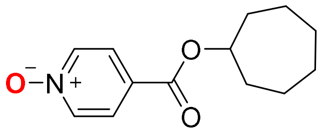

P7-Noxy

Prepared following the general procedure reported above. Conditions: 5 mg of Mn(^{TIPS}mcp) (0.00505 mmol, 1 mol%),111 mg of **S7** (0.505 mmol, 1.0 eq), 1.67 mL of 0.905 M H_2O_2 solution in MeCN (1.51 mmol, 3 eq), 4 mL of MeCN at 0 °C for 30 min. The crude obtained by scale up oxidation of **S7** was oxidized by chromic acid. The purification by flash chromatography over silica gel (DCM: MeOH 100:1) afforded 82 mg of **P7-Noxy**, (0.35 mmol, 69% yield) as a white solid. ^{1}H-NMR (400 MHz, $CDCl_3$) δ, ppm: 8.21 (d, J = 7.1 Hz, 2H), 7.86 (d, J = 7.1 Hz, 2H), 5.21 – 5.13 (m, 1H), 2.00 (ddd, J = 13.0, 7.7, 3.4 Hz, 2H), 1.84 – 1.76 (m, 2H), 1.75 – 1.66 (m, 2H), 1.63 – 1.56 (m, 4H), 1.55 – 1.42 (m, 2H). ^{13}C NMR (400 MHz, $CDCl_3$) δ ppm: 162.62, 139.36, 127.47, 126.34, 77.08, 33.72, 28.23, 22.80. HRMS (QTOF) m/z calculated for $C_{13}H_{17}NO_3$ $[M+Na]^+$ 258.1101, found 258.1095.

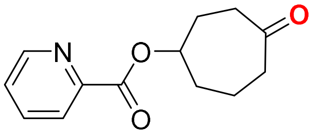

P8-K4

Prepared following the general procedure reported above. Conditions: 5 mg of Mn(^{TIPS}mcp) (0.00505 mmol, 1 mol%),111 mg of **S8** (0.505 mmol, 1.0 eq), 1.67 mL of 0.905 M H_2O_2 solution in MeCN (1.51 mmol, 3 eq), 4 mL of MeCN at 0 °C for 30 min. The purification by flash chromatography over silica gel (hexane-EtOAc 1:2) afforded 61 mg of **P8-K4**, (0.26 mmol, 51% yield) as a white solid. ^{1}H-NMR (400 MHz, $CDCl_3$) δ, ppm: 8.80 (d, *J* = 4.1 Hz, 1H), 8.12 (d, *J* = 7.8 Hz, 1H), 7.87 (td, *J* = 7.7, 1.7 Hz, 1H), 7.50 (m, *J* = 7.6, 4.7, 1.0 Hz, 1H), 5.39 – 5.27 (m, 1H), 2.79 – 2.71 (m, 1H), 2.62 – 2.53 (m, 3H), 2.20 – 2.12 (m, 3H), 2.06 (m, *J* = 14.0, 5.0, 2.2 Hz, 2H), 1.84 – 1.74 (m, 1H) ^{13}C NMR (400 MHz, $CDCl_3$) δ ppm: 213.20, 164.20, 150.06, 148.19, 137.01, 126.92, 125.13, 75.13, 43.47, 37.93, 34.88, 28.96, 19.22. HRMS (QTOF) m/z calculated for $C_{13}H_{15}NO_3$ $[M+Na]^+$ 256.0944, found 256.0964.

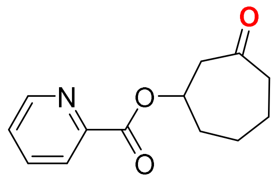

P8-K3

Prepared following the general procedure reported above. Conditions: 5 mg of Mn(^{TIPS}mcp) (0.00505 mmol, 1 mol%),111 mg of **S8** (0.505 mmol, 1.0 eq), 1.67 mL of 0.905 M H_2O_2 solution in MeCN (1.51 mmol, 3 eq), 4 mL of MeCN at 0 °C for 30 min. The purification by flash chromatography over silica gel (hexane-EtOAc 1:2) afforded 22 mg of **P8-K3**, (0.093 mmol, 18% yield) as a white solid. ^{1}H-NMR (400 MHz, $CDCl_3$) δ, ppm: 8.80 (d, J = 4.7 Hz, 1H), 8.09 (d, J = 7.9 Hz, 1H), 7.86 (td, J = 7.8, 1.7 Hz, 1H), 7.50 (m, J = 7.6, 4.7, 1.1 Hz, 1H), 5.49 (tt, J = 8.4, 2.9 Hz, 1H), 3.01 (m, J = 17.7, 15.3, 5.5 Hz, 2H), 2.71 – 2.55 (m, 2H), 2.27 – 2.06 (m, 2H), 1.96 – 1.75 (m, 4H). ^{13}C NMR (400 MHz, $CDCl_3$) δ ppm: 210.04, 164.03, 150.10, 148.01, 137.01, 126.96, 125.15, 71.09, 48.52, 44.43, 35.30, 25.08, 23.91. HRMS (QTOF) m/z calculated for $C_{13}H_{15}NO_3$ $[M+Na]^+$ 256.0944, found 256.0964.

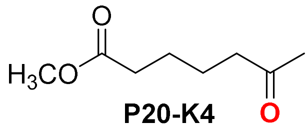

P20-K4

Prepared following the general procedure reported above. Conditions: 5 mg of Mn(^{TIPS}mcp) (0.00505 mmol, 1 mol%),73 mg of **S20** (0.505 mmol, 1.0 eq), 1.11 mL of 0.910 M H_2O_2 solution in NFTBA (1.01 mmol, 2 eq), 4 mL of NFTBA at 0 °C for 30 min. The crude obtained by scale up oxidation of **S20** was oxidized by chromic acid. The purification by flash chromatography over silica gel (hexane-EtOAc 3:1) afforded 51 mg of **P20-K4**, (0.32 mmol, 64% yield) as a colorless oil. Spectroscopic data match those previously reported.[4] ^{1}H-NMR (400 MHz, $CDCl_3$) δ, ppm: 3.67 (s, 3H), 2.48 (t, J = 7.2 Hz, 2H), 2.42 (q, J = 7.2 Hz, 2H), 2.34 (t, J = 7.2 Hz, 2H), 1.90 (ap, J = 7.2 Hz, 2H), 1.05 (t, J = 7.2 Hz, 3H).

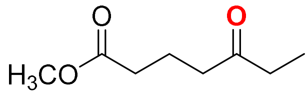

P20-K3

Prepared following the general procedure reported above. Conditions: 5 mg of Mn(^{TIPS}mcp) (0.00505 mmol, 1 mol%),73 mg of **S20** (0.505 mmol, 1.0 eq), 1.11 mL of 0.910 M H_2O_2 solution in NFTBA (1.01 mmol, 2 eq), 4 mL of NFTBA at 0 °C for 30 min. The crude obtained by scale up oxidation of **S20** was oxidized by chromic acid. The purification by flash chromatography over silica gel (hexane-EtOAc 3:1) afforded 8.3 mg of **P20-K3**, (0.053 mmol, 10% yield) as a colorless oil. Spectroscopic data match those previously reported.[4] ^{1}H-NMR (400 MHz, $CDCl_3$) δ, ppm: 3.68 (s, 3H), 2.72 (t, J = 6.4 Hz, 2H), 2.59 (t, J = 6.4 Hz, 2H), 2.43 (t, J = 7.2 Hz, 2H), 1.62 (aq, J = 7.2 Hz, 2H), 0.92 (t, J = 7.2 Hz, 3H).

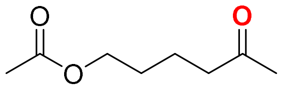

P21-K4

Prepared following the general procedure reported above. Conditions: 5 mg of Mn(^{TIPS}mcp) (0.00505 mmol, 1 mol%),73 mg of **S21** (0.505 mmol, 1.0 eq), 1.12 mL of 0.903 M H_2O_2 solution in NFTBA (1.01 mmol, 2 eq), 4 mL of NFTBA at 0 °C for 30 min. The crude obtained by scale up oxidation of **S21** was oxidized by chromic acid. The purification by flash chromatography over silica gel (hexane-EtOAc 2:1) afforded 57 mg of **P21-K4**, (0.36 mmol, 71% yield) as a colorless oil. Spectroscopic data match those previously reported.[39] ^{1}H-NMR (400 MHz, $CDCl_3$) δ, ppm: 4.05 (t, J = 6.0 Hz, 2H), 2.46 (t, J = 6.8 Hz, 2H), 2.14 (s, 3H), 2.04 (s, 3H), 1.60–1.66 (m, 4H).

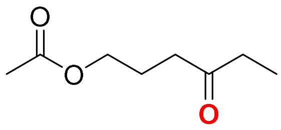

P21-K3

Prepared following the general procedure reported above. Conditions: 5 mg of Mn(^{TIPS}mcp) (0.00505 mmol, 1 mol%),73 mg of **S21** (0.505 mmol, 1.0 eq), 1.12 mL of 0.903 M H_2O_2 solution in NFTBA (1.01 mmol, 2 eq), 4 mL of NFTBA at 0 °C for 30 min. The crude obtained by scale up oxidation of **S21** was oxidized by chromic acid. The purification by flash chromatography over silica gel (hexane-EtOAc 2:1) afforded 11 mg of **P21-K3**, (0.070 mmol, 14% yield) as a colorless oil. Spectroscopic data match those previously reported.[39] ^{1}H-NMR (400 MHz, $CDCl_3$) δ, ppm: 4.07 (t, J = 6.0 Hz 2H), 2.50 (t, J = 6.8 Hz, 2H), 2.45 (q, J = 7.2 Hz, 2H), 2.2-1.5 (m, 2H), 2.06 (s, 3H), 1.05 (t, J = 7.0 Hz, 3H).

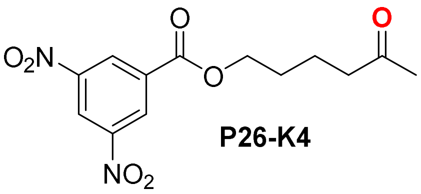

P26-K4

Prepared following the general procedure reported above. Conditions: 5 mg of Mn(^{TIPS}mcp) (0.00505 mmol, 1 mol%),73 mg of **S26** (0.505 mmol, 1.0 eq), 1.12 mL of 0.903 M H_2O_2 solution in HFIP (1.01 mmol, 2 eq), 4 mL of HFIP at 0 °C for 30 min. The crude obtained by scale up oxidation of **S26** was oxidized by chromic acid. The purification by flash chromatography over silica gel (hexane-EtOAc 1:1) afforded 121 mg of **P26-K4**, (0.390 mmol, 77% yield) as a white solid. Spectroscopic data match those previously reported.[10] ^{1}H-NMR (400 MHz, $CDCl_3$) δ, ppm: 9.17 (t, J = 2.2 Hz, 1H), 9.10 (d, J = 2.2 Hz, 2H), 4.42 (t, J = 6.5 Hz, 2H), 2.53 (t, J = 7.1 Hz, 2H), 2.13 (s, 3H), 1.85 – 1.71 (m, 4H).

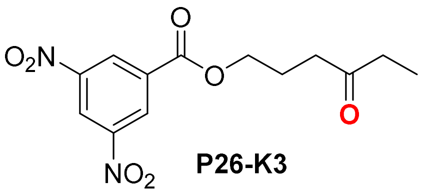

P26-K3

Prepared following the general procedure reported above. Conditions: 5 mg of Mn(^{TIPS}mcp) (0.00505 mmol, 1 mol%),73 mg of **S26** (0.505 mmol, 1.0 eq), 1.12 mL of 0.903 M H_2O_2 solution in HFIP (1.01 mmol, 2 eq), 4 mL of HFIP at 0 °C for 30 min. The crude obtained by scale up oxidation of **S26** was oxidized by chromic acid. The purification by flash chromatography over silica gel (hexane-EtOAc 1:1) afforded 7 mg of **P26-K3**, (0.023 mmol, 5% yield) as a white solid. Spectroscopic data match those previously reported.[10] ^{1}H-NMR (400 MHz, $CDCl_3$) δ, ppm: 9.21 (t, J = 2.2 Hz, 1H), 9.12 (d, J = 2.2 Hz, 2H), 4.46 (t, J = 6.5 Hz, 2H), 2.60 (t, J = 7.0 Hz, 2H), 2.47 (q, J = 7.3 Hz, 2H), 2.12 (m, 2H), 1.06 (t, J = 7.3 Hz, 3H).

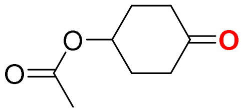

P38-K4

Prepared following the general procedure reported above. Conditions: 5 mg of Mn(^{TIPS}mcp) (0.00505 mmol, 1 mol%), 72 mg of **S38** (0.505 mmol, 1.0 eq), 1.12 mL of 0.903 M H_2O_2 solution in NFTBA (1.01 mmol, 2 eq), 4 mL of NFTBA at 0 °C for 30 min. The crude obtained by scale up oxidation of **S38** was oxidized by chromic acid. The purification by flash chromatography over silica gel (hexane-EtOAc 3:1) afforded 61 mg of **P38-K4**, (0.32 mmol, 77% yield) as a colorless liquid. Spectroscopic data match those previously reported. [40] ^{1}H-NMR (400 MHz, $CDCl_3$) δ, ppm: 5.16 (m, 1H), 2.57-2.47 (m, 2H), 2.38-2.32 (m, 2H), 2.12 (s, 3H), 2.08-2.03 (m, 4H).

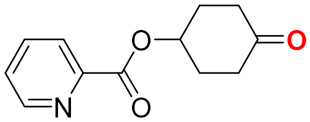

P40-K4

Prepared following the general procedure reported above. Conditions: 5 mg of Mn(^{TIPS}mcp) (0.00505 mmol, 1 mol%), 104 mg of **S40** (0.505 mmol, 1.0 eq), 1.65 mL of 0.920 M H_2O_2 solution in MeCN (1.52 mmol, 3 eq), 4 mL of MeCN at 0 °C for 30 min. The purification by flash chromatography over silica gel (hexane-EtOAc 1:3) afforded 41 mg of **P40-K4**, (0.185 mmol, 37% yield) as a white solid. ^{1}H NMR (400 MHz, $CDCl_3$) δ ppm: 8.79 (dd, J = 4.7, 0.6 Hz, 1H), 8.13 (d, J = 7.8 Hz, 1H), 7.87 (td, J = 7.7, 1.7 Hz, 1H), 7.50 (m, J = 7.6, 4.8, 1.0 Hz, 1H), 5.54 – 5.47 (m, 1H), 2.70 – 2.61 (m, 2H), 2.50 – 2.42 (m, 2H), 2.33 – 2.22 (m, 4H). ^{13}C NMR (400 MHz, $CDCl_3$) δ 209.46, 164.47, 150.07, 148.04, 137.02, 127.01, 125.13, 70.42, 37.47, 30.42. HRMS (QTOF) m/z calculated for $C_{12}H_{13}NO_3$ $[M+Na]^+$ 242.0788, found 242.0785.

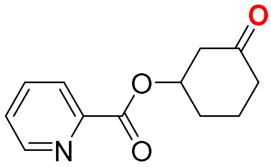

P40-K3

Prepared following the general procedure reported above. Conditions: 5 mg of Mn(^{TIPS}mcp) (0.00505 mmol, 1 mol%), 104 mg of **S40** (0.505 mmol, 1.0 eq), 1.65 mL of 0.920 M H_2O_2 solution in MeCN (1.52 mmol, 3 eq), 4 mL of MeCN at 0 °C for 30 min. The purification by flash chromatography over silica gel (hexane-EtOAc 1:3) afforded 7 mg of **P40-K3**, (0.032 mmol, 6% yield) as a white solid. ^{1}H-NMR (400 MHz, $CDCl_3$) δ, ppm: 8.80 (d, J = 4.0 Hz, 1H), 8.10 (d, J = 7.8 Hz, 1H), 7.86 (td, J = 7.7, 1.7 Hz, 1H), 7.50 (m, J = 7.6, 4.7, 1.1 Hz, 1H), 5.58 – 5.51 (m, 1H), 2.85 (dd, J = 14.7, 4.6 Hz, 1H), 2.74 (dd, J = 14.7, 7.4 Hz, 1H), 2.45 (t, J = 6.6 Hz, 2H), 2.24 – 2.10 (m, 3H), 1.92 – 1.84 (m, 1H). ^{13}C NMR (400 MHz, $CDCl_3$) δ 207.84, 164.02, 150.07, 147.87, 137.02, 127.01, 125.23, 72.99, 46.71, 40.88, 29.54, 20.75. HRMS (QTOF) m/z calculated for $C_{12}H_{13}NO_3$ $[M+Na]^+$ 242.0788, found 242.0785.

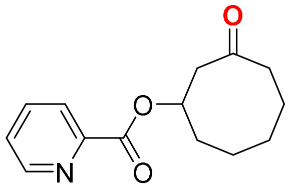

P41-K3

Prepared following the general procedure reported above. Conditions: 5 mg of Mn(^{TIPS}mcp) (0.00505 mmol, 1 mol%), 118 mg of **S41** (0.505 mmol, 1.0 eq), 1.65 mL of 0.920 M H_2O_2 solution in MeCN (1.52 mmol, 3 eq), 4 mL of MeCN at 0 °C for 30 min. The purification by flash chromatography over silica gel (hexane-EtOAc 1:2) afforded 9 mg of **P41-K3**, (0.037 mmol, 7% yield) as a white solid. ^{1}H-NMR (400 MHz, $CDCl_3$) δ, ppm: 8.80 (d, J = 3.8 Hz, 1H), 8.16 (d, J = 7.9 Hz, 1H), 7.87 (td, J = 7.7, 1.7 Hz, 1H), 7.50 (m, J = 7.6, 4.7, 1.1 Hz, 1H), 5.54 (tt, J = 10.3, 4.0 Hz, 1H), 3.09 – 3.01 (m, 1H), 2.92 – 2.84 (m, 1H), 2.65 – 2.39 (m, 2H), 2.24 – 1.83 (m, 5H), 1.74 (td, J = 10.1, 4.9 Hz, 1H), 1.48 (ddd, J = 12.4, 7.0, 3.8 Hz, 1H), 1.32 – 1.21 (m, 1H). ^{13}C NMR (400 MHz, $CDCl_3$) δ 211.41, 164.21, 149.98, 148.17, 137.02, 126.91, 125.32, 73.39, 45.83, 43.57, 32.05, 27.46, 24.12, 20.79. HRMS (QTOF) m/z calculated for $C_{14}H_{17}NO_3$ $[M+Na]^+$ 270.1101, found 270.1122.

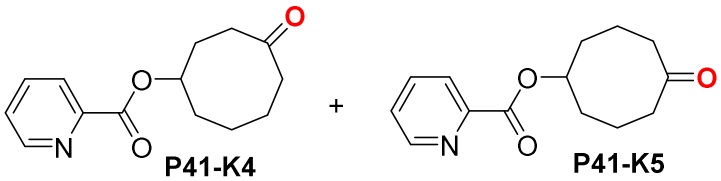

P41-K4 + P41-K5

Prepared following the general procedure reported above. Conditions: 5 mg of Mn(^{TIPS}mcp) (0.00505 mmol, 1 mol%), 118 mg of **S41** (0.505 mmol, 1.0 eq), 1.65 mL of 0.920 M H_2O_2 solution in MeCN (1.52 mmol, 3 eq), 4 mL of MeCN at 0 °C for 30 min. The purification by flash chromatography over silica gel (hexane-EtOAc 1:2) afforded 42 mg of **P41-K4+P41-K5**, (0.170 mmol, 34% yield) as a white solid. ^{1}H-NMR (400 MHz, $CDCl_3$) δ, ppm: 1H NMR (400 MHz, CDCl3) δ 8.74 (dd, J = 9.1, 4.7 Hz), 8.06 (d, J = 7.8 Hz), 7.82 (t, J = 7.7 Hz),

7.45 (dd, J = 12.2, 5.4 Hz), 5.22 (dt, J = 12.0, 6.0 Hz), 4.95 (dt, J = 6.9, 3.5 Hz), 2.60 – 2.38 (m), 2.36 – 2.29 (m), 2.11 – 1.93 (m), 1.90 (m), 1.78 (m), 1.64 (m).^{13}C NMR (400 MHz, $CDCl_3$) δ, ppm: 216.25, 215.59, 164.33, 164.20, 150.01, 149.85, 148.22, 136.94, 126.83, 125.05, 75.20, 74.94, 41.89, 40.70, 38.87, 33.35, 30.44, 28.48, 27.69, 22.66, 22.28. HRMS (QTOF) m/z calculated for $C_{14}H_{17}NO_3$ $[M+Na]^+$ 270.1101, found 270.1122.

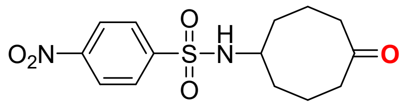

P43-K5

Prepared following the general procedure reported above. Conditions: 5 mg of Mn(^{TIPS}mcp) (0.00505 mmol, 1 mol%), 158 mg of **S43** (0.505 mmol, 1.0 eq), 1.13 mL of 0.897 M H_2O_2 solution in NFTBA (1.01 mmol, 2 eq), 4 mL of NFTBA at 0 °C for 30 min. The crude obtained by scale up oxidation of **S43** was oxidized by chromic acid. The purification by flash chromatography over silica gel (hexane-EtOAc 1:1) afforded 98 mg of **P43-K5**, (0.30 mmol, 59% yield) as a white solid. ^{1}H-NMR (400 MHz, $CDCl_3$) δ, ppm: 8.40 – 8.36 (m, 2H), 8.11 – 8.06 (m, 2H), 5.22 (d, J = 7.8 Hz, 1H), 3.51 (ddq, J = 11.8, 7.9, 4.0 Hz, 1H), 2.59 – 2.33 (m, 4H), 2.30 – 2.12 (m, 1H), 2.10 – 1.97 (m, 1H), 1.90 – 1.74 (m, 2H), 1.68 – 1.50 (m, 2H), 1.46 – 1.29 (m, 2H).^{13}C NMR (400 MHz, $CDCl_3$) δ, ppm: 214.76, 149.99, 147.01, 128.21, 124.43, 52.37, 44.57, 43.10, 32.99, 27.83, 22.80, 20.08. HRMS (QTOF) m/z calculated for $C_{14}H_{18}N_2O_5S$ $[M+Na]^+$ 349.0829, found 349.0821.

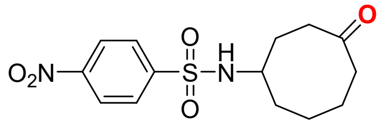

P43-K4

Prepared following the general procedure reported above. Conditions: 5 mg of Mn(^{TIPS}mcp) (0.00505 mmol, 1 mol%), 158 mg of **S43** (0.505 mmol, 1.0 eq), 1.13 mL of 0.897 M H_2O_2 solution in NFTBA (1.01 mmol, 2 eq), 4 mL of NFTBA at 0 °C for 30 min. The crude obtained by scale up oxidation of **S43** was oxidized by chromic acid. The purification by flash chromatography over silica gel (hexane-EtOAc 1:1) afforded 7 mg of **P43-K4**, (0.021 mmol, 4% yield) as a yellow solid. ^{1}H-NMR (400 MHz, $CDCl_3$) δ, ppm: 8.40 – 8.36 (m, 2H), 8.14 – 8.09 (m, 2H), 5.60 (d, J = 7.9 Hz, 1H), 3.88 – 3.79 (m, 1H), 2.87 – 2.76 (m, 1H), 2.49 – 2.38 (m, 1H), 2.37 – 2.14 (m, 3H), 1.94 (qdd, J = 15.9, 11.7, 6.3 Hz, 2H), 1.83 – 1.65 (m, 3H), 1.61 – 1.49 (m, 2H). ^{13}C NMR (400 MHz, $CDCl_3$) δ, ppm: 216.60, 150.01, 146.96, 128.11, 124.45, 53.87, 40.06, 39.66, 31.71, 28.80, 28.58, 22.98. HRMS (QTOF) m/z calculated for $C_{14}H_{18}N_2O_5S$ $[M+Na]^+$ 349.0829, found 349.0821.

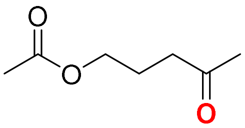

P44-K4

Prepared following the general procedure reported above. Conditions: 5 mg of Mn(TIPSmcp) (0.00505 mmol, 1 mol%), 66 mg of **S44** (0.505 mmol, 1.0 eq), 1.12 mL of 0.903 M H_2O_2 solution in NFTBA (1.01 mmol, 2 eq), 4 mL of NFTBA at 0 °C for 30 min. The crude obtained by scale up oxidation of **S44** was oxidized by chromic acid. The purification by flash chromatography over silica gel (hexane-EtOAc 5:1) afforded 27 mg of **P44-K4**, (0.19 mmol, 38% yield) as a colorless liquid, Spectroscopic data match those previously reported.[41] ^{1}H-NMR (400 MHz, $CDCl_3$) δ, ppm: 4.12 (t, J = 6.4 Hz, 2H), 2.54 (t, J = 7.2 Hz, 2H), 2.17 (s, 3H), 2.05 (s, 3H),1.93 (m, 2H).

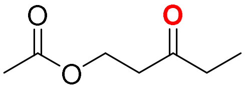

P44-K3

Prepared following the general procedure reported above. Conditions: 5 mg of Mn(TIPSmcp) (0.00505 mmol, 1 mol%), 66 mg of **S44** (0.505 mmol, 1.0 eq), 1.12 mL of 0.903 M H_2O_2 solution in NFTBA (1.01 mmol, 2 eq), 4 mL of NFTBA at 0 °C for 30 min. The crude obtained by scale up oxidation of **S44** was oxidized by chromic acid. The purification by flash chromatography over silica gel (hexane-EtOAc 5:1) afforded 5 mg of **P44-K3**, (0.035 mmol, 7% yield) as a colorless liquid. Spectroscopic data match those previously reported. [42] ^{1}H-NMR (400 MHz, $CDCl_3$) δ, ppm: 5.29 (m, 1H), 2.79 (m, 2H), 2.56 (m, 2H), 2.44 (q, J = 12.0, 8.0 Hz, 2H), 2.07 (s, 3H), 1.27 (d, J = 8.0 Hz, 3H) and 1.05 (t, J = 8.0 Hz, 3H).

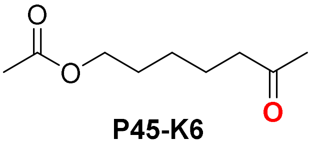

P45-K6

Prepared following the general procedure reported above. Conditions: 5 mg of Mn(TIPSmcp) (0.00505 mmol, 1 mol%), 80 mg of **S45** (0.505 mmol, 1.0 eq), 1.06 mL of 0.955 M H_2O_2 solution in NFTBA (1.01 mmol, 2 eq), 4 mL of NFTBA at 0 °C for 30 min. The crude obtained by scale up oxidation of **S45** was oxidized by chromic acid. The purification by flash chromatography over silica gel (hexane-EtOAc 3:1) afforded 65 mg of **P45-K5**, (0.38 mmol, 75% yield) as a colorless liquid. Spectroscopic data match those previously reported.[39] ^{1}H-NMR (400 MHz, $CDCl_3$) δ, ppm: 4.03 (t, J = 6.5 Hz, 2H), 2.43 (t, J = 6.4 Hz, 2H), 2.13 (s, 3H), 2.03 (s, 3H), 1.90-1.17 (m, 6H).

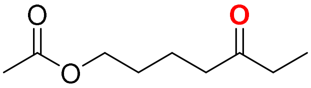

P45-K5

Prepared following the general procedure reported above. Conditions: 5 mg of Mn(TIPSmcp) (0.00505 mmol, 1 mol%), 80 mg of **S45** (0.505 mmol, 1.0 eq), 1.06 mL of 0.955 M H_2O_2 solution in NFTBA (1.01 mmol, 2 eq), 4 mL of NFTBA at 0 °C for 30 min. The crude obtained by scale up oxidation of **S45**

was oxidized by chromic acid. The purification by flash chromatography over silica gel (hexane-EtOAc 3:1) afforded 10 mg of **P45-K4**, (0.060 mmol, 12% yield) as a colorless liquid. Spectroscopic data match those previously reported.[39] ^{1}H-NMR (400 MHz, $CDCl_3$) δ, ppm: 4.05 (t, J = 6.5 Hz, 2H), 2.65-2.22 (m, 4H), 2.03 (s, 3H), 1.78-1.45 (m,4H), 1.05 (t, J = 6.5 Hz, 3H).

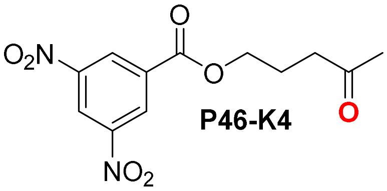

P46-K4

Prepared following the general procedure reported above. Conditions: 5 mg of Mn(^{TIPS}mcp) (0.00505 mmol, 1 mol%), 142 mg of **S46** (0.505 mmol, 1.0 eq), 1.60 mL of 0.949 M H_2O_2 solution in MeCN (1.52 mmol, 3 eq), 4 mL of MeCN at 0 °C for 30 min. The purification by flash chromatography over silica gel (hexane-EtOAc 1:2) afforded 58 mg of **P46-K4**, (0.196 mmol, 39% yield) as a whte solid. Spectroscopic data match those previously reported.[10] ^{1}H-NMR (400 MHz, $CDCl_3$) δ, ppm: 9.22 (t, J = 2.2 Hz, 1H), 9.13 (d, J = 2.2 Hz, 2H), 4.46 (t, J = 6.5 Hz, 2H), 2.63 (t, J = 7.0 Hz, 2H), 2.19 (s, 3H), 2.12 (p, J = 6.7 Hz, 2H).

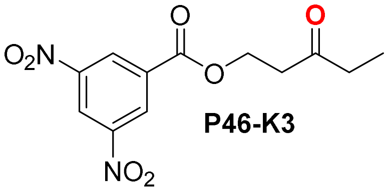

P46-K3

Prepared following the general procedure reported above. Conditions: 5 mg of Mn(^{TIPS}mcp) (0.00505 mmol, 1 mol%), 142 mg of **S46** (0.505 mmol, 1.0 eq), 1.60 mL of 0.949 M H_2O_2 solution in MeCN (1.52 mmol, 3 eq), 4 mL of MeCN at 0 °C for 30 min.. The purification by flash chromatography over silica gel (hexane-EtOAc 1:2) afforded 4 mg of **P46-K3**, (0.015 mmol, 63% yield) as a whte solid. Spectroscopic data match those previously reported.[10] ^{1}H-NMR (400 MHz, $CDCl_3$) δ, ppm: 9.22 (t, J = 2.2 Hz, 1H), 9.11 (d, J = 2.2 Hz, 2H), 4.72 (t, J = 6.2 Hz, 2H), 2.97 (t, J = 6.3 Hz, 2H), 2.54 (q, J = 7.3 Hz, 2H), 1.12 (t, J = 7.3 Hz, 3H).

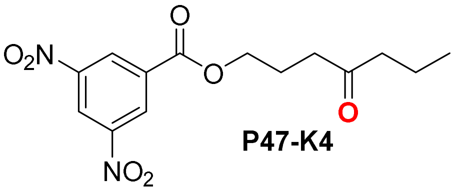

P47-K4

Prepared following the general procedure reported above. Conditions: 5 mg of Mn(^{TIPS}mcp) (0.00505 mmol, 1 mol%), 157 mg of **S47** (0.505 mmol, 1.0 eq), 1.12 mL of 0.906 M H_2O_2 solution in HFIP (1.01 mmol, 2 eq), 4 mL of HFIP at 0 °C for 30 min. The crude obtained by scale up oxidation of **S47** was oxidized by chromic acid. The purification by flash chromatography over silica gel (hexane-EtOAc 1:3) afforded 7 mg of **P47-K4**, (0.022 mmol, 4% yield) as a white solid. ^{1}H-NMR (400 MHz, $CDCl_3$) δ, ppm: 9.25 (t, J = 2.1 Hz, 1H), 9.16 (d, J = 2.1 Hz, 2H), 4.49 (t, J = 6.6 Hz, 2H), 2.62 (t, J = 7.0 Hz, 2H), 2.45 (t, J = 7.3 Hz, 2H), 2.15 (q, J = 6.8 Hz, 2H), 1.62 (dd, J = 14.7, 7.3 Hz, 2H), 0.94 (t, J = 7.4 Hz, 3H). ^{13}C NMR

(400 MHz, $CDCl_3$) δ, ppm: 209.36, 162.46, 148.68, 133.90, 129.40, 122.40, 66.30, 44.85, 38.68, 22.55, 17.30, 13.72. HRMS (QTOF) m/z calculated for $C_{14}H_{16}N_2O_7$ $[M+Na]^+$ 347.0850, found 347.0850.

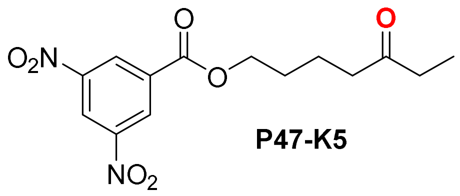

Prepared following the general procedure reported above. Conditions: 5 mg of Mn(^{TIPS}mcp) (0.00505 mmol, 1 mol%), 157 mg of **S47 (**0.505 mmol, 1.0 eq), 1.12 mL of 0.906 M H_2O_2 solution in HFIP (1.01 mmol, 2 eq), 4 mL of HFIP at 0 °C for 30 min. The crude obtained by scale up oxidation of **S47** was oxidized by chromic acid. The purification by flash chromatography over silica gel (hexane-EtOAc 1:3) afforded 7 mg of **P47-K5**, (0.022 mmol, 4% yield) as a white solid. ^{1}H-NMR (400 MHz, $CDCl_3$) δ, ppm: 9.25 (t, J = 2.1 Hz, 1H), 9.18 (d, J = 2.1 Hz, 2H), 4.48 (t, J = 6.4 Hz, 2H), 2.58 – 2.42 (m, 4H), 1.91 – 1.82 (m, 2H), 1.82 – 1.73 (m, 2H), 1.09 (t, J = 7.3 Hz, 3H) ^{13}C NMR (400 MHz, $CDCl_3$) δ 210.71, 162.51, 148.68, 134.00, 129.42, 122.36, 66.64, 41.43, 36.05, 28.10, 20.00, 7.83. HRMS (QTOF) m/z calculated for $C_{14}H_{16}N_2O_7$ $[M+Na]^+$ 347.0850, found 347.0850.

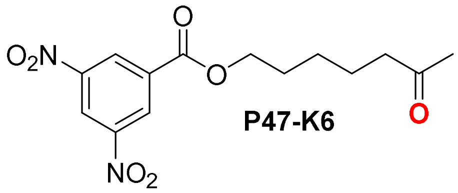

Prepared following the general procedure reported above. Conditions: 5 mg of Mn(^{TIPS}mcp) (0.00505 mmol, 1 mol%), 157 mg of **S47 (**0.505 mmol, 1.0 eq), 1.12 mL of 0.906 M H_2O_2 solution in HFIP (1.01 mmol, 2 eq), 4 mL of HFIP at 0 °C for 30 min. The crude obtained by scale up oxidation of **S47** was oxidized by chromic acid. The purification by flash chromatography over silica gel (hexane-EtOAc 1:3) afforded 76 mg of **P47-K6**, (0.23 mmol, 46% yield) as a white solid. ^{1}H-NMR (400 MHz, $CDCl_3$) δ, ppm: 9.25 (t, J = 2.1 Hz, 1H), 9.18 (d, J = 2.1 Hz, 2H), 4.48 (t, J = 6.7 Hz, 2H), 2.55 – 2.46 (m, 2H), 2.18 (s, 3H), 1.92 – 1.83 (m, 2H), 1.74 – 1.65 (m, 2H), 1.52 – 1.45 (m, 2H). ^{13}C NMR (400 MHz, $CDCl_3$) δ, ppm: 208.49, 162.51, 148.68, 134.06, 129.42, 122.34, 66.74, 43.33, 29.98, 28.44, 25.46, 23.19. HRMS (QTOF) m/z calculated for $C_{14}H_{16}N_2O_7$ $[M+Na]^+$ 347.0850, found 347.0851.

5.2. Products identified by ^{1}H NMR analysis of the catalysis crude mixture

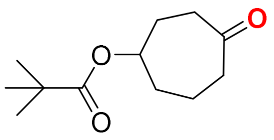

P3-K4

P3-K4 was identified by ^{1}H-NMR analysis of the catalysis crude mixture in NFTBA (**figure S63-S64**). Spectroscopic data match those previously reported.[4] ^{1}H-NMR (400 MHz, $CDCl_3$) δ, ppm: 5.04 (ddd, *J* = 10.4, 7.3, 3.4 Hz, 1H), 2.69 (ddd, *J* = 15.8, 9.2, 3.6 Hz, 1H), 2.55 (dd, *J* = 6.9, 4.8 Hz, 2H), 2.46 (ddd, *J* = 15.8, 8.3, 3.6 Hz, 1H), 2.02 – 1.85 (m, 5H), 1.80 – 1.68 (m, 1H), 1.23 (s, 9H).

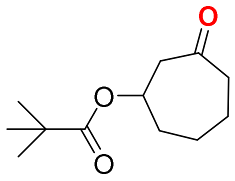

P3-K3

P3-K3 was identified byc^{1}H-NMR analysis of the catalysis crude mixture in NFTBA (**figure S63-S65**), Spectroscopic data match those previously reported.[4] ^{1}H-NMR (400 MHz, $CDCl_3$) δ, ppm: 5.23 – 5.14 (m, 1H), 3.15 – 3.00 (m, 1H), 2.82 (t, J = 5.8 Hz, 1H), 2.05 (m, J = 6.2, 4.1 Hz, 3H), 1.23 (s, 9H).

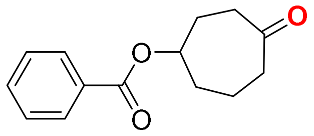

P4-K4

P4-K4 was identified by ^{1}H-NMR analysis of the catalysis crude mixture in MeCN (**figure S66-S67**), Spectroscopic data match those previously reported.[31] ^{1}H-NMR (400 MHz, $CDCl_3$) δ, ppm: 8.06 (d, J = 8.0 Hz), 7.60 (dd, J = 12.0, 9.4 Hz), 7.48 (t, J = 7.7 Hz) (overlapped aromatic peaks of **P4-K3**, **P4-K4** and **S4**), 5.32 (tt, J = 7.6, 3.9 Hz, 1H), 2.83 – 2.72 (m, 1H), 2.65 – 2.50 (m, 3H), 2.17 – 2.09 (m, 2H), 2.08 – 1.98 (m, 4H).

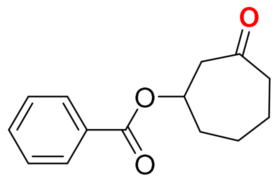

P4-K3

P4-K3 was identified by ^{1}H-NMR analysis of the catalysis crude mixture in MeCN (**figure S66-S68**), Spectroscopic data match those previously reported.[31] ^{1}H-NMR (400 MHz, $CDCl_3$) δ, ppm: 8.06 (d, J = 8.0 Hz), 7.60 (dd, J = 12.0, 9.4 Hz), 7.48 (t, J = 7.7 Hz) (overlapped aromatic peaks of **P4-K3**, **P4-K4** and **S4**), 5.50 – 5.39 (m, 1H), 3.07 – 2.89 (m, 2H), 2.28 – 2.15 (m, 2H).

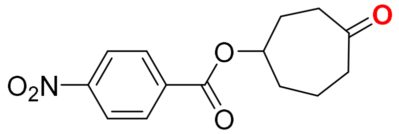

P5-K4

P5-K4 was identified by ^{1}H-NMR analysis of the catalysis crude mixture in HFIP (**figure S69-S70**), ^{1}H NMR (400 MHz, $CDCl_3$) δ ppm: 8.35 – 8.31 (m, 2H), 8.25 – 8.20 (m, 2H), 5.38 – 5.29 (m, 1H), 2.79 – 2.69 (m, 1H), 2.66 – 2.51 (m, 3H), 2.14 (dt, J = 8.2, 5.0 Hz, 2H), 2.10 – 1.97 (m, 3H), 1.90 – 1.73 (m, 1H).

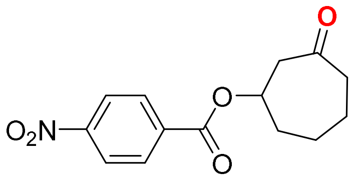

P5-K3

P5-K3 was identified by ^{1}H-NMR analysis of the catalysis crude mixture in HFIP (**figure S69-S71**), ^{1}H NMR (400 MHz, $CDCl_3$) δ ppm: 8.35 – 8.31 (m), 8.25 – 8.20 (m) (partially overlapped with the aromatic peaks of **P5-K4**), 5.47 (s, 1H), 3.03 – 2.96 (m, 1H).

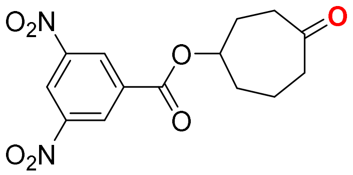

P6-K4

P6-K4 was identified by ^{1}H-NMR analysis of the catalysis crude mixture in MeCN (**figure S72-S73**).^{1}H-NMR (400 MHz, $CDCl_3$) δ, ppm: 9.26 (t, J = 2.1 Hz, 1H), 9.16 (d, J = 2.1 Hz, 2H), 5.35 (ddd, J = 11.8, 8.2, 3.6 Hz, 1H), 2.78 – 2.68 (m, 1H), 2.61 (ddd, J = 13.1, 10.6, 4.9 Hz, 3H), 2.24 – 2.13 (m, 2H), 2.05 (tdd, J = 13.8, 9.8, 7.6 Hz, 2H), 1.95 – 1.77 (m, 2H).

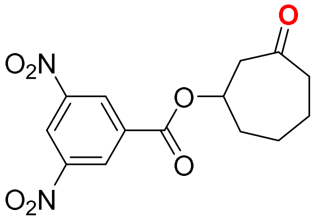

P6-K3

P6-K3 was identified by ^{1}H-NMR analysis of the catalysis crude mixture in MeCN (**figure S72-S74**), ^{1}H NMR (400 MHz, $CDCl_3$) δ, ppm: 9.26 (t, J = 2.1 Hz) (overlapped with the aromatic signals of **P6-K3**), 9.12 (d, J = 2.1 Hz, 2H), 5.50 (ddd, J = 10.7, 6.9, 3.1 Hz, 1H), 3.06 (m, 2H).

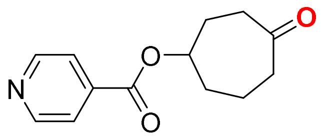

P7-K4

P7-K4 was identified by ^{1}H-NMR analysis of the catalysis crude mixture in NFTBA (**figure S75-S76**),^{1}H-NMR (400 MHz, $CDCl_3$) δ, ppm: 8.81 (dd, J = 4.4, 1.6 Hz, 1H), 7.86 (dd, J = 4.4, 1.6 Hz, 1H), 5.38 – 5.29 (m, 1H), 2.74 (ddd, J = 9.0, 7.8, 4.9 Hz, 1H), 2.65 – 2.51 (m, 2H), 2.18 – 1.95 (m, 5H), 1.91 – 1.75 (m, 1H).

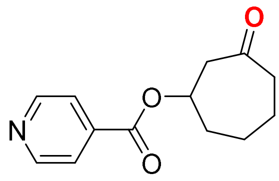

P7-K3

P7-K3 was identified by ^{1}H-NMR analysis of the catalysis crude mixture in NFTBA (**figure S75-S77**). ^{1}H-NMR (400 MHz, $CDCl_3$) δ, ppm: 8.81 (dd, J = 4.4, 1.6 Hz), 7.86 (dd, J = 4.4, 1.6 Hz) (overlapped with the aromatic signals of **P7-K4** signals), 5.46 (s, 1H), 3.03 – 2.93 (m, 2H).

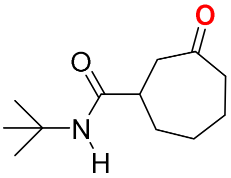

P9-K3

+

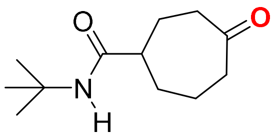

P9-K4

P9-K3 and **P9-K4** were identified by ^{1}H-NMR analysis of the catalysis crude mixture in MeCN (**figure S78.**). ^{1}H-NMR (400 MHz, $CDCl_3$) δ, ppm: 5.25 (s), 5.32 (s) 2.90 (m), 2.71 – 2.59 (m), 1.36 (s), 1.35 (s).

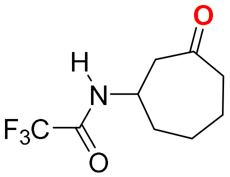

P10-K3

+

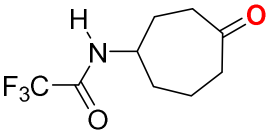

P10-K4

P10-K3 and **P10-K4** were identified by ^{1}H-NMR analysis of the catalysis crude mixture in MeCN (**figure S79.**). ^{1}H-NMR (400 MHz, $CDCl_3$) δ, ppm: 6.53 (s), 6.38 (s), 4.38 – 4.22 (m), 4.05 (tdd, J = 11.1, 7.7, 3.6 Hz, 4H), 3.00 – 2.91 (m), 2.76 – 2.45 (m).

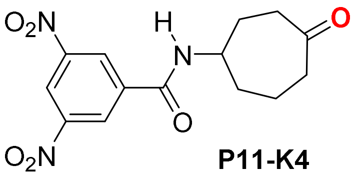

P11-K4

P11-K4 was identified by ^{1}H-NMR analysis of the catalysis crude mixture in HFIP (**figure S80-S81**).^{1}H-NMR (400 MHz, $CDCl_3$) δ, ppm: 9.20 (t, J = 2.0 Hz, 1H), 8.95 (t, J = 2.6 Hz, 2H), 6.35 (d, 1H), 4.26 (dd, J = 7.7, 3.4 Hz, 1H), 2.83 – 2.49 (m, 3H), 2.30 (m, 3H), 1.83 (m, 3H).

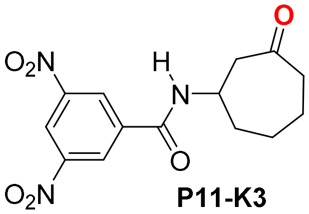

P11-K3

P11-K3 was identified by ^{1}H-NMR analysis of the catalysis crude mixture in HFIP (**figure S80-S82**).^{1}H-NMR (400 MHz, $CDCl_3$) δ, ppm: 6.56 (s, 1H), 4.52 (m, 1H), 3.21-3.05 (m, 2H), 2.05-1.99 (m, 3H).

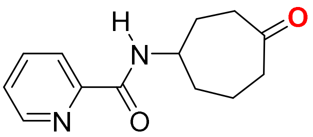

P12-K4

P12-K4 was identified by ^{1}H-NMR analysis of the catalysis crude mixture in NFTBA (**figure S83-S84**). ^{1}H-NMR (400 MHz, $CDCl_3$) δ, ppm: 8.57 (d, J = 4.7 Hz, 1H), 8.22 (d, J = 7.9 Hz, 1H), 8,12 (s, 1H), 7.88 (td, J = 7.7, 1.7 Hz, 1H), 7.46 (ddd, J = 7.6, 4.8, 1.2 Hz, 1H), 4.22 (m, 1H), 2.73 – 2.50 (m, 4H), 2.21 (m, 2H), 1.98 (m, 1H), 1.93 – 1.76 (m, 2H), 1.75 – 1.61 (m, 1H).

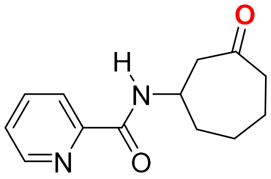

P12-K3

P12-K3 was identified by ^{1}H-NMR analysis of the catalysis crude mixture in NFTBA (**figure S83-S85**). ^{1}H-NMR (400 MHz, $CDCl_3$) δ, ppm: 4.58 – 4.29 (m, 1H), 2.98 – 2.94 (m, 1H), 2.81-2.75 (m, 1H).

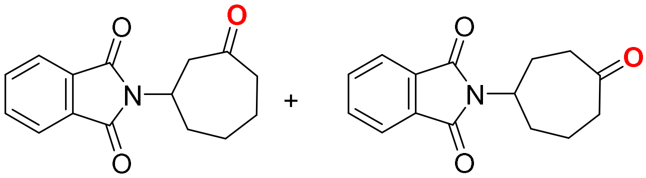

P13-K3 P13-K4

P13-K3 and **P13-K4** were identified by ^{1}H-NMR analysis of the catalysis crude mixture in HFIP (**figure S86-S87**), Spectroscopic data match those previously reporte.[31] ^{1}H-NMR (400 MHz, $CDCl_3$) δ, ppm: 7.84 (ddd, J = 10.6, 5.5, 3.1 Hz, 1H), 7.72 (ddd, J = 13.9, 5.5, 3.1 Hz, 1H), 4.52 (tt, J = 12.0, 2.8 Hz, 1H), 4.29 (tt, J = 10.9, 3.9 Hz, 1H), 3.68 (dd, J = 14.9, 12.2 Hz, 1H), 2.71 – 2.56 (m, 1H), 2.51 – 2.37 (m, 1H), 2.35 – 2.21 (m, 1H), 2.12 – 1.95 (m, 1H), 1.93 – 1.77 (m, 1H), 1.70 (ddd, J = 14.5, 10.2, 5.2 Hz, 1H).

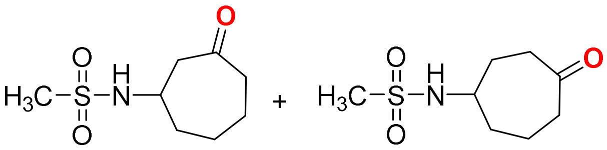

P14-K3 P14-K4

P14-K3 and **P14-K4** were identified by ^{1}H-NMR analysis of the catalysis crude mixture in MeCN (**figure S88**). ^{1}H-NMR (400 MHz, $CDCl_3$) δ, ppm: 1H NMR (400 MHz, CDCl3) δ 4.38 (d, J = 5.9 Hz, 1H), 4.33 (d, J = 7.9 Hz, 2H), 3.87 (dtd, J = 10.3, 6.9, 3.0 Hz, 1H), 3.69 – 3.59 (m, 2H), 2.92 (dd, J = 13.7, 2.7 Hz, 1H), 2.75 – 2.68 (m, 2H), 2.62 – 2.50 (m, 9H).

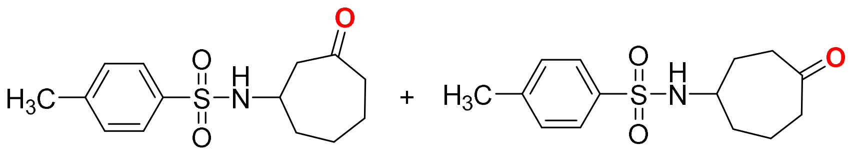

P15-K3 P15-K4

P15-K3 and **P15-K4** were identified by ^{1}H-NMR analysis of the catalysis crude mixture in MeCN (**Figure S89-S90-S91**). ^{1}H-NMR (400 MHz, $CDCl_3$) δ, ppm: 7.81 – 7.75 (m, 5H), 7.34 – 7.29 (m, 6H), 4.64 (d, J = 7.3 Hz, 1H), 3.81 – 3.68 (m, 1H), 2.56 – 2.46 (m, 1H), 2.45 (s, 10H), 2.45 – 2.36 (m, 3H), 2.37 – 2.24 (m, 1H), 2.15 (m, 2H), 2.01 – 1.83 (m, 3H). **P15-K3**: ^{1}H NMR (400 MHz, $CDCl_3$) δ ppm: 7.81 – 7.75 (m, 5H), 7.34 – 7.30 (m, 6H), 5.20 (d, J = 7.5 Hz, 1H), 3.90 – 3.80 (m, 1H), 2.93 – 2.84 (m, 1H), 2.83 – 2.77 (m, 1H), 2.74 (dd, J = 12.5, 3.8 Hz, 2H), 2.45 (s, 10H), 2.23 (m, 3H), 2.02 (m, 3H).

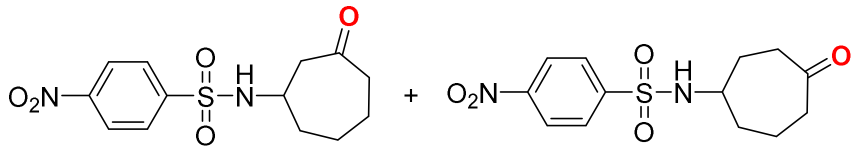

P16-K3 P16-K4

P16-K3 and **P16-K4** were identified by ^{1}H-NMR analysis of the catalysis crude mixture in MeCN (**figure S92-S93-S94**). ^{1}H-NMR (400 MHz, $CDCl_3$) δ, ppm: 8.38 (dt, J = 9.3, 2.1 Hz), 8.08 (dt, J = 9.1, 2.4 Hz), 5.54 (d, J = 7.3 Hz), 4.82 (d, J = 8.0 Hz), 3.89 – 3.82 (m), 3.51 (pd, J = 8.1, 4.0 Hz), 2.97 – 2.79 (m), 2.59 – 2.32 (m), 2.32 – 2.11 (m).

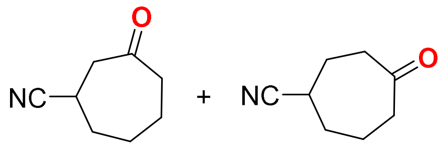

P17-K3 P17-K4

P17-K3 and **P17-K4** were identified by ^{1}H-NMR and GCanalysis of the catalysis crude mixture in HFIP (**figure S95-S96**). ^{1}H NMR (400 MHz, $CDCl_3$) δ, ppm:3.02 (tt, J = 6.8, 3.2 Hz, 1H), 2.95 – 2.77 (m, 1H), 2.75 – 2.44 (m, 3H), 2.17 – 1.90 (m, 4H), 1.90 – 1.76 (m, 2H).

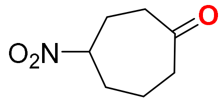

P18-K4

P18-K4 was identified by ^{1}H-NMR analysis of the catalysis crude mixture in NFTBA (**figure S97-S98**). ^{1}H-NMR (400 MHz, $CDCl_3$) δ, ppm: 4.59 (ddd, J = 13.7, 8.9, 3.7 Hz, 1H), 2.78 – 2.67 (m, 1H), 2.65 – 2.40 (m, 3H), 2.42 – 2.29 (m, 1H), 2.29 – 2.14 (m, 1H), 2.08 (tdd, J = 16.6, 9.4, 3.9 Hz, 2H), 1.85 – 1.66 (m, 2H). **P18-K3**: ^{1}H-NMR (400 MHz, $CDCl_3$) δ, ppm: 4.76 – 4.71 (m, 1H).

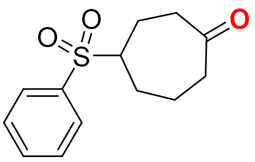

P19-K4

P19-K4 was identified by ^{1}H-NMR analysis of the catalysis crude mixture in NFTBA (**figure S99-S100**), ^{1}H-NMR (400 MHz, $CDCl_3$) δ, ppm: 7.91 (dd, J = 5.2, 3.4 Hz, 2H), 7.72 (ddd, J = 7.5, 4.0, 1.1 Hz, 1H), 7.62 (t, J = 7.6 Hz, 2H), 3.09 – 2.97 (m, 1H), 2.67 (ddd, J = 16.5, 6.5, 2.6 Hz, 1H), 2.58 – 2.43 (m, 4H), 2.18 – 2.08 (m, 1H), 1.87 – 1.72 (m, 1H), 1.67 – 1.58 (m, 4H).

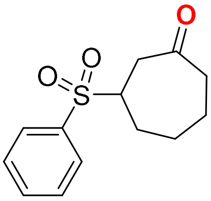

P19-K3

P19-K3 was identified by ^{1}H-NMR analysis of the catalysis crude mixture in NFTBA, (**figure S99-S101**), ^{1}H-NMR (400 MHz, $CDCl_3$) δ, ppm: 3.26 – 3.20 (m, 1H), 2.95 – 2.92 (m, 1H), 2.76 – 2.74 (m, 1H).

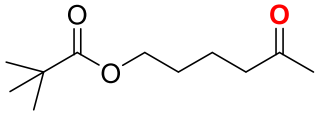

P22-K5

P22-K5 was identified by ^{1}H-NMR analysis of the catalysis crude mixture in NFTBA (**figure S105-S106**). ^{1}H-NMR (400 MHz, $CDCl_3$) δ, ppm: 4.08 (t, J = 5.9 Hz, 2H), 2.50 (tt, J = 7.0, 3.7 Hz, 2H overlapped with **P22-K4** peak), 2.17 (s, 3H), 1.73 – 1.61 (m, 4H), 1.22 (s, 9H).

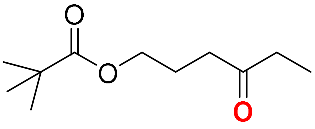

P22-K4

P22-K4: was identified by ^{1}H-NMR analysis of the catalysis crude mixture in NFTBA (**figure S105-S107**). ^{1}H NMR (400 MHz, CDCl3) δ 4.32 – 4.22 (t, 2H), 2.51 (t, 2H overlapped with **P22-K5** peak), 2.46 (q, 2H), 1.96 (dd, J = 13.7, 6.7 Hz, 2H), 1.28 (s, 11H), 1.09 (t, J = 7.4 Hz, 3H).

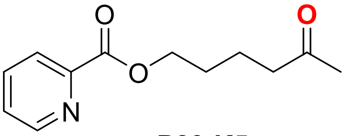

P23-K5

P23-K5 was identified by ^{1}H-NMR analysis of the catalysis crude mixture in NFTBA (**figure S108-S109**). ^{1}H-NMR (400 MHz, $CDCl_3$) δ, ppm: 8.79 (d, J = 6.4 Hz, 1H), 8.15 (d, J = 6.9 Hz, 1H), 7.94 – 7.81 (m, 1H), 7.54 – 7.47 (m, 1H), 4.45 (t, J = 6.5 Hz, 2H), 2.54 (t, J = 7.2 Hz, 2H), 2.17 (s, 3H), 1.91 – 1.82 (m, 2H), 1.81 – 1.70 (m, 2H).

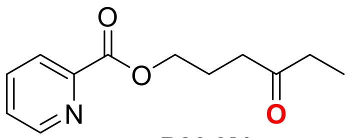

P23-K4

P23-K4 was identified by ^{1}H-NMR analysis of the catalysis crude mixture in NFTBA (**figure S108-S110**). ^{1}H NMR (400 MHz, $CDCl_3$) δ, ppm 4.47 (t, J = 4.3 Hz, 2H), 2.61 (t, J = 7.3 Hz, 2H), 2.47 (q, J = 7.2 Hz, 2H), 2.17 – 2.10 (m, 2H), 1.08 (t, J = 7.3 Hz, 3H). (aromatic peaks are overlapped with **P23-K5** ones).

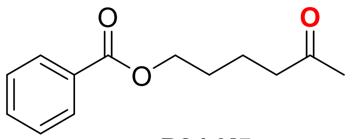

P24-K5

P24-K5 was identified by ^{1}H-NMR analysis of the catalysis crude mixture in NFTBA (**figure S111-S112**), ^{1}H-NMR (400 MHz, $CDCl_3$) δ, ppm: 8.06 (d, J = 6.1 Hz, 2H), 7.58 (t, J = 7.4 Hz, 1H), 7.46 (t, J = 7.7 Hz, 2H), 4.35 (t, J = 6.0 Hz, 2H), 2.54 (t, J = 6.8 Hz, 2H), 2.14 (s, 3H) 1.87 – 1.71 (m, 4H).

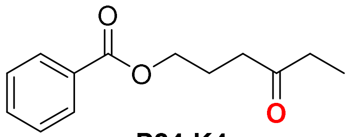

P24-K4

P24-K4: was identified by ^{1}H-NMR analysis of the catalysis crude mixture in NFTBA (**figure S111-S113**). ^{1}H-NMR (400 MHz, $CDCl_3$) δ, ppm: 4.62 (t, J = 6.5 Hz, 2H), 2.60 (t, J = 7.3 Hz, 2H), 2.48 (dd, J = 14.6, 7.3 Hz, 3H), 2.15 – 2.06 (m, 2H), 1.09 (t, J = 8.0, 3H). (aromatic peaks are overlapped with **P24-K5** ones).

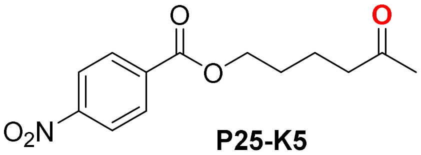

P25-K5

P25-K5 was identified by ^{1}H-NMR analysis of the catalysis crude mixture in NFTBA (**figure S114-S115**). ^{1}H-NMR (400 MHz, $CDCl_3$) δ, ppm: 8.31 (d, J = 8.9 Hz, 2H), 8.23 (d, J = 8.9 Hz, 2H), 4.44 – 4.35 (t, J = 7.0 Hz, 1H), 2.56 (t, J = 6.9 Hz, 1H), 2.19 (s, 3H), 1.87 – 1.71 (m, 4H).

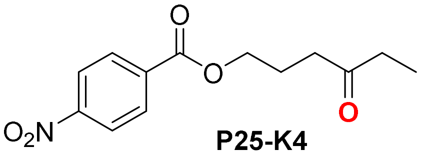

P25-K4

P25-K4 was identified by ^{1}H-NMR analysis of the catalysis crude mixture in NFTBA (**figure S114-S116**). ^{1}H-NMR (400 MHz, $CDCl_3$) δ, ppm: 4.42 (t, J = 3.2 Hz, 2H), 2.61 (t, J = 7.1 Hz, 2H), 2.48 (dd, J = 14.6, 7.3 Hz, 1H), 2.12 (dd, J = 13.7, 6.8 Hz, 2H), 1.09 (t, J = 7.3 Hz, 3H).

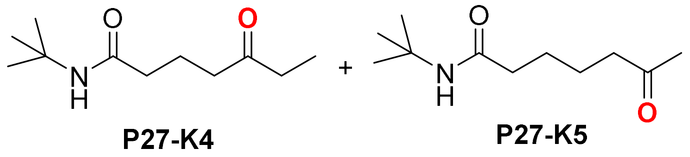

P27-K4 + P27-K5

P25-K4 and **P25-K5** were identified by ^{1}H-NMR analysis of the catalysis crude mixture in MeCN (**figure S117-S118-S119**). ^{1}H-NMR (400 MHz, $CDCl_3$) δ, ppm: 1H NMR (400 MHz, CDCl3) δ 5.49 (s), 5.35 (s), 2.75 (t, J = 6.5 Hz), 2.47 (dt, J = 23.2, 7.1 Hz), 2.37 (t, J = 6.6 Hz), 2.06 (s), 1.90 (p, J = 7.1), 1.90 (p, J = 7.1 Hz), 1.66 – 1.55 (m), 1.36 (d, J = 1.8 Hz), 1.15 – 1.04 (m).

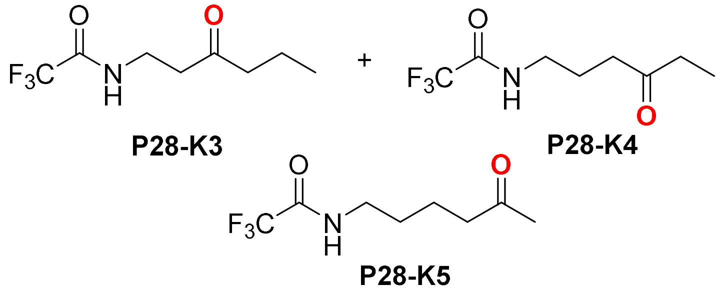

P28-K3 + P28-K4, P28-K5

P28-K3, **P28-K4** and **P28-K5** were identified by ^{1}H-NMR analysis of the catalysis crude mixture in MeCN (**figure S120-S121-S122**). ^{1}H-NMR (400 MHz, $CDCl_3$) δ, ppm: 7.02 (s), 6.74 (s), 3.61 (dd, J = 11.6, 5.8 Hz), 3.38 (p, J = 6.2 Hz), 2.78 – 2.69 (m), 2.58 (t, J = 6.5 Hz), 2.53 (t, J = 6.4 Hz), 2.51 – 2.45 (m), 2.19 (d, J = 2.9 Hz), 1.92 – 1.88 (m), 1.68 – 1.57 (m), 1.09 (t, J = 7.3 Hz).

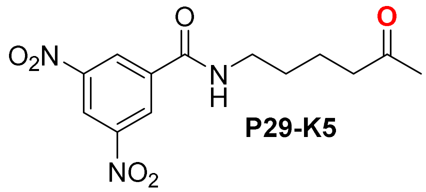

P29-K5

P29-K5 was identified by ^{1}H-NMR analysis of the catalysis crude mixture in HFIP (**figure S123-S124**). ^{1}H-NMR (400 MHz, $CDCl_3$) δ,ppm: 9.19 (t, J = 2.0 Hz, 1H), 9.10 (d, J = 2.0 Hz, 2H), 3.53 (dd, J = 11.7, 6.0 Hz, 2H), 2.62 (t, J = 6.1 Hz, 2H), 1.72 (dt, J = 11.7, 4.6 Hz, 4H).

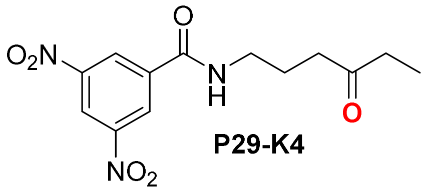

P29-K4

P29-K4 was identified by ^{1}H-NMR analysis of the catalysis crude mixture in HFIP (**figure S123-S125**). ^{1}H-NMR (400 MHz, $CDCl_3$) δ,ppm 9.05 (d, J = 2.0 Hz, 2H) (aromatic peaks are partially overlapped with **P29-K5** ones), 3.53 (dd, J = 11.7, 6.0 Hz, 10 H) (**P29-K4**+**P29-K5**), 2.76 – 2.71 (t, 2H), 2.55 (dd, J = 14.7, 7.4 Hz, 2H), 2.00 (dt, J = 12.3, 6.2 Hz, 2H), 1.13 (t, J = 7.3 Hz, 3H).

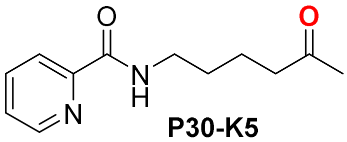

P30-K5

P30-K5 was identified by ^{1}H-NMR analysis of the catalysis crude mixture in HFIP (**figure S126-S127**). ^{1}H-NMR (400 MHz, $CDCl_3$) δ, ppm: 8.57 (ddd, J = 4.9, 1.6, 0.9 Hz, 1H), 8.23 – 8.19 (m, 1H), 7.87 (td, J = 7.7, 1.7 Hz, 1H), 7.48 – 7.41 (m, 1H), 3.53 – 3.45 (m, 2H), 2.53 (t, J = 6.9 Hz, 2H), 2.17 (s, 3H), 1.76 – 1.65 (m, 4H).

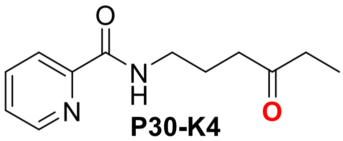

P30-K4

P30-K4 was identified by ^{1}H-NMR analysis of the catalysis crude mixture in HFIP (**figure S126-S128**). ^{1}H-NMR (400 MHz, $CDCl_3$) δ, ppm 3.53 (dd, J = 6.9, 3.1 Hz) (**P30-K4**+**P30-K5**), 2.55 (t, J = 7.3 Hz, 2H), 2.46 (dd, J = 14.4, 7.1 Hz, 2H), 1.96 (dt, J = 13.8, 6.8 Hz, 2H), 1.06 (t, J = 7.3 Hz, 3H). (aromatic peaks are partially overlapped with **P30-K5** ones).

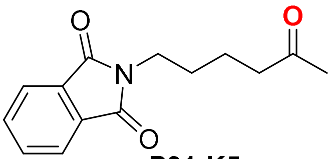

P31-K5

P31-K5 was identified by ^{1}H-NMR analysis of the catalysis crude mixture in HFIP (**figure S129-S130**). ^{1}H-NMR (400 MHz, $CDCl_3$) δ, ppm: 7.88 – 7.84 (m, 2H), 7.77 – 7.71 (m, 2H), 3.72 (t, J = 6.9 Hz, 2H), 2.51 (t, J = 7.2 Hz, 2H), 2.16 (s, 3H), 1.76 – 1.67 (m, 4H).

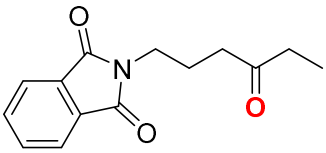

P31-K4

P31-K4: was identified by ^{1}H-NMR analysis of the catalysis crude mixture in HFIP (**figure S129-S131**). ^{1}H-NMR (400 MHz, $CDCl_3$) δ, ppm: 3.72 (t, J = 6.9 Hz) (**P31-K4**+**P31-K5**), 2.51 (t, J = 7.2 Hz) (**P31-K4**+**P31-K5**), 2.48 – 2.34 (m, 2H), 2.05 – 1.95 (m, 2H), 1.06 (t, J = 7.3 Hz, 1H).

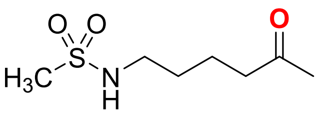

P32-K5

P32-K5 was identified by ^{1}H-NMR analysis of the catalysis crude mixture in MeCN, (**figure S132-S133**). ^{1}H-NMR (400 MHz, $CDCl_3$) δ, ppm: 4.34 (s, 1H), 3.43 – 3.32 (q, 2H), 2.97 (s, 3H), 2.52 (t, J = 6.7 Hz, 2H), 2.20 (s, 3H), 1.88 (m, 2H), 1.71 – 1.61 (m, 2H).

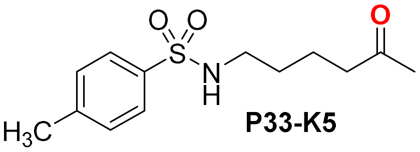

P33-K5

P33-K5 was identified by ^{1}H-NMR analysis of the catalysis crude mixture in HFIP (**figure S134-S135**). ^{1}H-NMR (400 MHz, $CDCl_3$) δ, ppm: 7.75 (t, J = 6.7 Hz, 2H), 7.37 – 7.31 (m, 2H), 4.42 (m, 1H), 2.97 – 2.91 (m, 2H), 2.48 – 2.38 (m, 5H s(Ph-**CH_3**)+t(-**CH2**-)), 2.13 (s, 3H), 1.49 (dd, J = 8.8, 6.4 Hz, 4H).

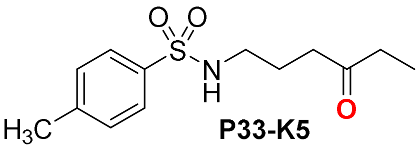

P33-K5

P33-K4 was identified by ^{1}H-NMR analysis of the catalysis crude mixture in HFIP (**figure S134-S136**). ^{1}H-NMR (400 MHz, $CDCl_3$) δ, ppm: 7.84 (d, J = 8.3 Hz, 2H), 7.33 (d, J = 8.0 Hz, **P33-K4**+**P33-K5**). 4.73 (s, 1H), 2.98 (dd, J = 8.7, 4.3 Hz, 2H), 2.51 (t, J = 6.7 Hz, 2H), 2.46 (s, 3H), 2.44 – 2.35 (m, 2H), 1.83 – 1.71 (m, 2H), 1.06 (t, J = 7.3 Hz, 3H).

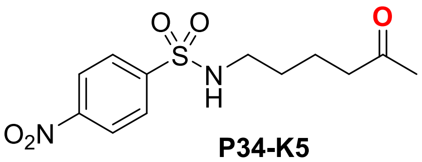

P34-K5

P34-K5, was identified by ^{1}H-NMR analysis of the catalysis crude mixture in NFTBA (**figure S137-S138**). ^{1}H-NMR (400 MHz, CD_3OD) δ, ppm: 8.47 – 8.38 (m, 2H), 8.14 – 8.04 (m, 2H), 2.96 – 2.90 (m, 2H), 2.47 (t, J = 7.1 Hz, 2H), 2.12 (s, 3H), 1.60 – 1.40 (m, 4H).

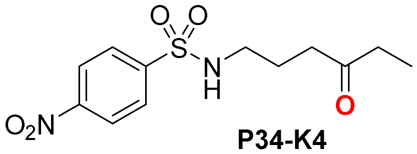

P34-K4

P34-K4, was identified by ^{1}H-NMR analysis of the catalysis crude mixture in NFTBA (**figure S137-S139**). ^{1}H-NMR (400 MHz, CD_3OD) δ, ppm: 2.97 – 2.91 (m) (**P34-K4**+**P34-K5**), 2.54-2.50 (t, J = 7.1 Hz, 2H), 2.43-2.38 (m, 2H), 1.76-1.66 (m, 2H), 1.03 – 1.00 (t, J = 7.1 Hz, 3H). (aromatic peaks are partially overlapped with **P34-K5** ones).

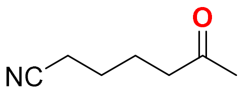

P35-K5

P35-K5 was identified by ^{1}H-NMR analysis of the catalysis crude mixture in MeCN (**figure S140-S141**). ^{1}H-NMR (400 MHz, $CDCl_3$) δ, ppm: 2.53 (t, J = 6.8 Hz, 1H), 2.38 (t, J = 6.8 Hz, 1H), 2.18 (s, 1H), 1.78 – 1.66 (m, 2H).

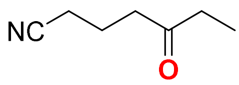

P35-K4

P35-K4 was identified by ^{1}H-NMR analysis of the catalysis crude mixture in MeCN (**figure S140-S142**). ^{1}H-NMR (400 MHz, $CDCl_3$) δ, ppm: 2.64 (t, J = 6.9 Hz, 1H), 2.46 (dt, J = 6.9, 2.4 Hz, 2H), 1.95 (p, J = 6.9 Hz, 1H), 1.10 (t, J = 7.3 Hz, 1H).

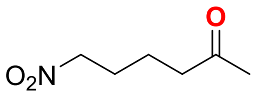

P36-K5

P36-K5 was identified by ^{1}H-NMR analysis of the catalysis crude mixture in NFTBA (**figure S143-S144**). ^{1}H-NMR (400 MHz, $CDCl_3$) δ, ppm: 4.41 (t, J = 6.9 Hz, 2H), 2.54 (t, J = 7.0 Hz, 2H), 2.18 (s, 3H), 2.04 (dt, J = 12.3, 7.1 Hz, 2H), 1.69 (ddd, J = 14.7, 10.3, 7.1 Hz, 2H).

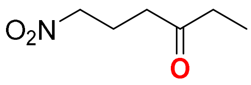

P36-K4

P36-K4 was identified by ^{1}H-NMR analysis of the catalysis crude mixture in NFTBA (**figure S143-S145**). ^{1}H-NMR (400 MHz, $CDCl_3$) δ, ppm: 4.46 (t, J = 7.0 Hz, 2H), 2.60 (t, J = 6.8 Hz, 2H), 2.47 (dd, J = 14.5, 7.3 Hz, 2H), 2.36 – 2.25 (m, 2H), 1.10 (t, J = 6.7 Hz, 3H).

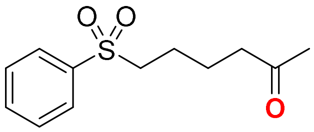

P37-K5

P37-K5 was identified by ^{1}H-NMR analysis of the catalysis crude mixture in NFTBA (**figure S146-S147**). ^{1}H-NMR (400 MHz, $CDCl_3$) δ, ppm: 7.95 – 7.91 (m, 2H), 7.74 – 7.65 (m, 1H), 7.63 – 7.57 (m, 2H), 3.14 – 3.09 (m, 2H), 2.46 (t, J = 6.9 Hz, 2H), 2.14 (s, 3H), 1.82 – 1.63 (m, 4H).

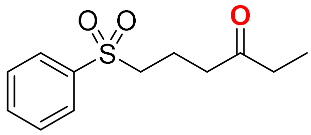

P37-K4

P37-K4 was identified by ^{1}H-NMR analysis of the catalysis crude mixture in NFTBA (**figure S146-S148**). ^{1}H-NMR (400 MHz, $CDCl_3$) δ, ppm: 3.20 – 3.14 (m, 2H), 2.64 (t, J = 6.7 Hz, 2H), 2.45 (dd, J = 16.9, 10.2 Hz, 2H), 2.01 (dt, J = 14.4, 4.1 Hz, 2H), 1.06 (t, J = 8.0 Hz, 3H).

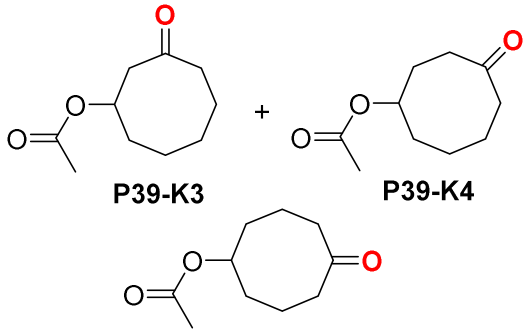

P39-K3 P39-K4 P39-K5

P39-K3, **P39-K4** and **P39-K5** were identified by ^{1}H-NMR analysis of the catalysis crude mixture in MeCN and HFIP, (**figure S149-S150-S151**), ^{1}H-NMR (400 MHz, $CDCl_3$) δ, ppm: 4.97 – 4.88 (m), 4.69 (ddd, J = 11.6, 7.6, 3.7 Hz), 2.82 – 2.71 (m), 2.57 – 2.49 (m), 2.49 – 2.34 (m), 2.31 – 2.10 (m), 2.05 (s), 2.02 (s), 1.99 – 1.92 (m), 1.91 – 1.87 (m), 1.87 – 1.80 (m), 1.76 – 1.68 (m): HFIP (**P39-K4**+**P39-K5**) ^{1}H-NMR (400 MHz, $CDCl_3$) δ, ppm: δ 2.07 (s, **P39-K3**), 2.05 (s, **P39-K4**), 2.02 (s, **P39-K5**): MeCN.

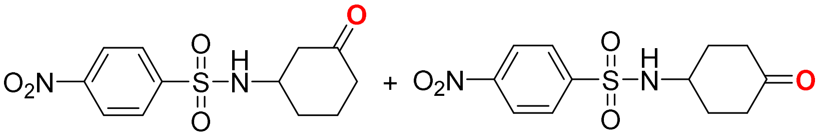

P42-K3 P42-K4

P42-K3 and **P42-K4** were identified by ^{1}H-NMR analysis of the catalysis crude mixture in MeCN (**figure S152-S153-S154**), ^{1}H-NMR (400 MHz, $CDCl_3$) δ, ppm: δ ^{1}H NMR (400 MHz, $CDCl_3$) δ ppm: 8.43 – 8.38 (m), 8.13 – 8.05 (m), (overlapped aromatic signals **P42-43**+**P42-K4**+**S42**) 4.99 (dd, J = 19.0, 6.6 Hz, N-**H P42-K3-P42-K4**), 3.90 (dd, J = 12.1, 6.0 Hz), 3.72 (m), 2.65 – 2.50 (m), 2.46 – 2.33 (m), 2.33 – 2.21 (m), 2.19 – 2.09 (m), 2.10 – 1.95 (m), 1.78 (dd, J = 14.6, 7.6 Hz).

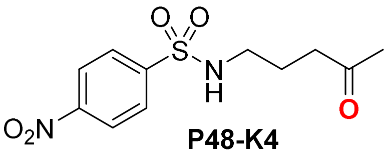

P48-K4

P48-K4 was identified by ^{1}H-NMR analysis of the catalysis crude mixture in NFTBA (**figure S155-S156**). ^{1}H-NMR (400 MHz, $CDCl_3$) δ, ppm: 8.39 (d, J = 8.8 Hz, 2H), 8.06 (d, J = 8.8 Hz, 2H), 5.03 (s, 1H), 3.05 (q, J = 6.4 Hz, 2H) (**P48-K4**+**S48**), 2.58 (t, J = 6.5 Hz, 2H), 2.18 (s, 3H), 1.84 – 1.74 (m, 2H).

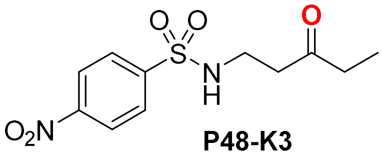

P48-K3

P48-K3 was identified by ^{1}H-NMR analysis of the catalysis crude mixture in NFTBA (**figure S155-S157**), ^{1}H-NMR (400 MHz, $CDCl_3$) δ, ppm: 8.40 (d, J = 8.6 Hz, 2H **P48-43**+**P48-K4**), 8.15 (d, J = 8.8 Hz, 2H, **P48-K3**+**P48-K4**), 5.41 (s, 1H), 3.23 (q, J = 11.7, 5.8 Hz, 2H), 2.78 – 2.72 (m, 2H), 1.07 (t, J = 6.6 Hz, 3H).

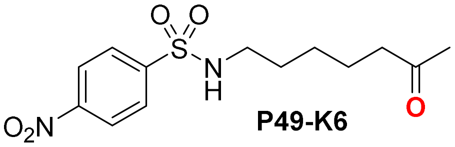

P49-K6

P49-K6 was identified by ^{1}H-NMR analysis of the catalysis crude mixture in HFIP (**figure S158-S159**), ^{1}H-NMR (400 MHz, $CDCl_3$) δ, ppm: 8.39 (d, J = 8.8 Hz, 2H), 8.10 – 8.07 (d, J = 8.8 Hz 2H), 4.83 (t, J = 5.9 Hz, 1H), 3.05 (dt, J = 9.8, 5.0 Hz, 2H), 2.46 – 2.42 (t, 2H), 2.15 (s, 3H), 1.58 – 1.49 (m, 6H).

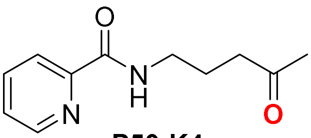

P50-K4

P50-K4 was identified by ^{1}H-NMR analysis of the catalysis crude mixture in HFIP (**figure S160-S161**), ^{1}H-NMR (400 MHz, $CDCl_3$) δ, ppm: 8.59 – 8.54 (m, 1H), 8.25 – 8.18 (m, 1H), 7.87 (tt, J = 7.7, 1.8 Hz, 1H), 7.45 (dtd, J = 5.5, 4.6, 1.1 Hz, 1H), 3.51(q, J = 14.4 Hz, 2H), 2.58 (t, J = 7.2 Hz, 2H), 2.18 (s, 3H), 1.95 (p, J = 7.1 Hz, 2H).

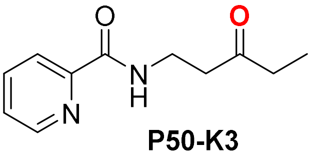

P50-K3

P50-K3 was identified by ^{1}H-NMR analysis of the catalysis crude mixture in HFIP (**figure S160-S162**), ^{1}H-NMR (400 MHz, $CDCl_3$) δ, ppm: 3.75 (dd, J = 12.3, 6.2 Hz, 2H), 2.81 (t, J = 6.1 Hz, 2H), 2.48 (dd, J = 14.6, 7.3 Hz, 2H), 1.10 (t, J = 7.3 Hz, 3H).

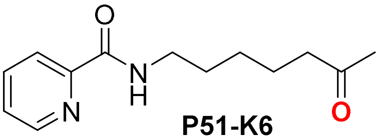

P51-K6

P51-K6 was identified by ^{1}H-NMR and GC analysis of the catalysis crude mixture in HFIP (**figure S163-S164-S165**), ^{1}H-NMR (400 MHz, $CDCl_3$) δ, ppm: 8.57 (d, J = 4.6 Hz, 1H), 8.22 (d, J = 7.8 Hz, 1H), 7.87 (td, J = 7.7, 1.6 Hz, 1H), 7.65 – 7.59 (m, 1H), 3.50 (q, J = 7.0, 2H), 2.47 (t, J = 9.4 2H), 2.15 (s, 2H), 1.68 (m, 3H).

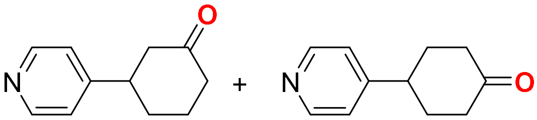

P52-K3 P52-K4

P52-K4 and **P52.K3** were identified by ^{1}H-NMR analysis of the catalysis crude mixture in HFIP, (**figure S166**), ^{1}H-NMR (400 MHz, $CDCl_3$) δ, ppm: 3.05 (m), 2.64 – 2.55 (m).

6. ^{1}H-NMR and ^{13}C-NMR spectra

6.1. NMR spectra of the substrates

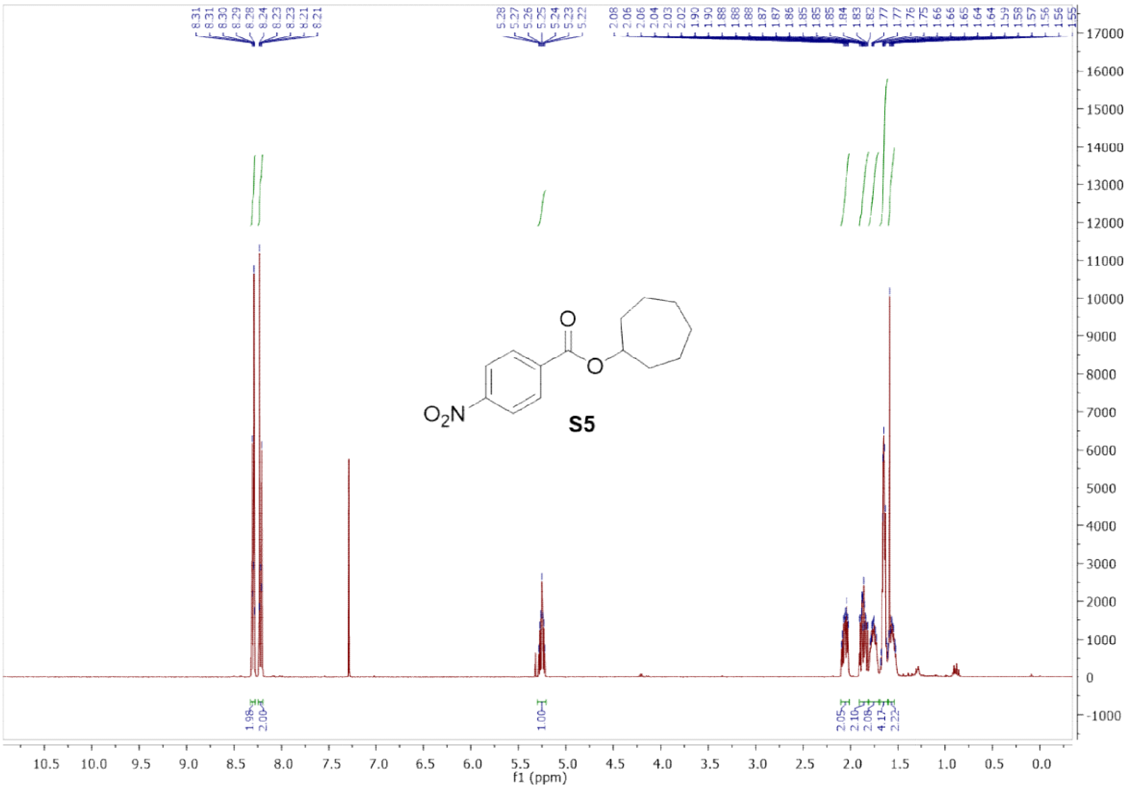

Figure S2. ^{1}H-NMR spectrum (400 MHz, $CDCl_3$) of cycloheptyl-4-nitrobenzoate (**S5**).

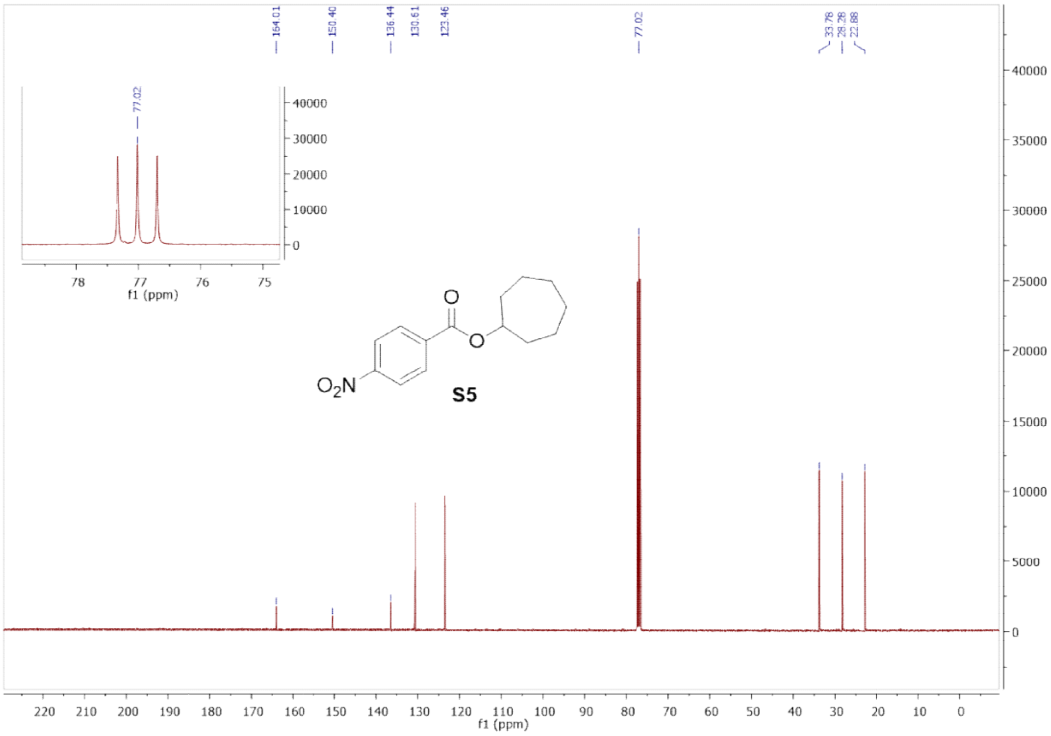

Figure S3. ^{13}C{^{1}H}-NMR spectrum (400 MHz, $CDCl_3$) of cycloheptyl-4-nitrobenzoate (**S5**).

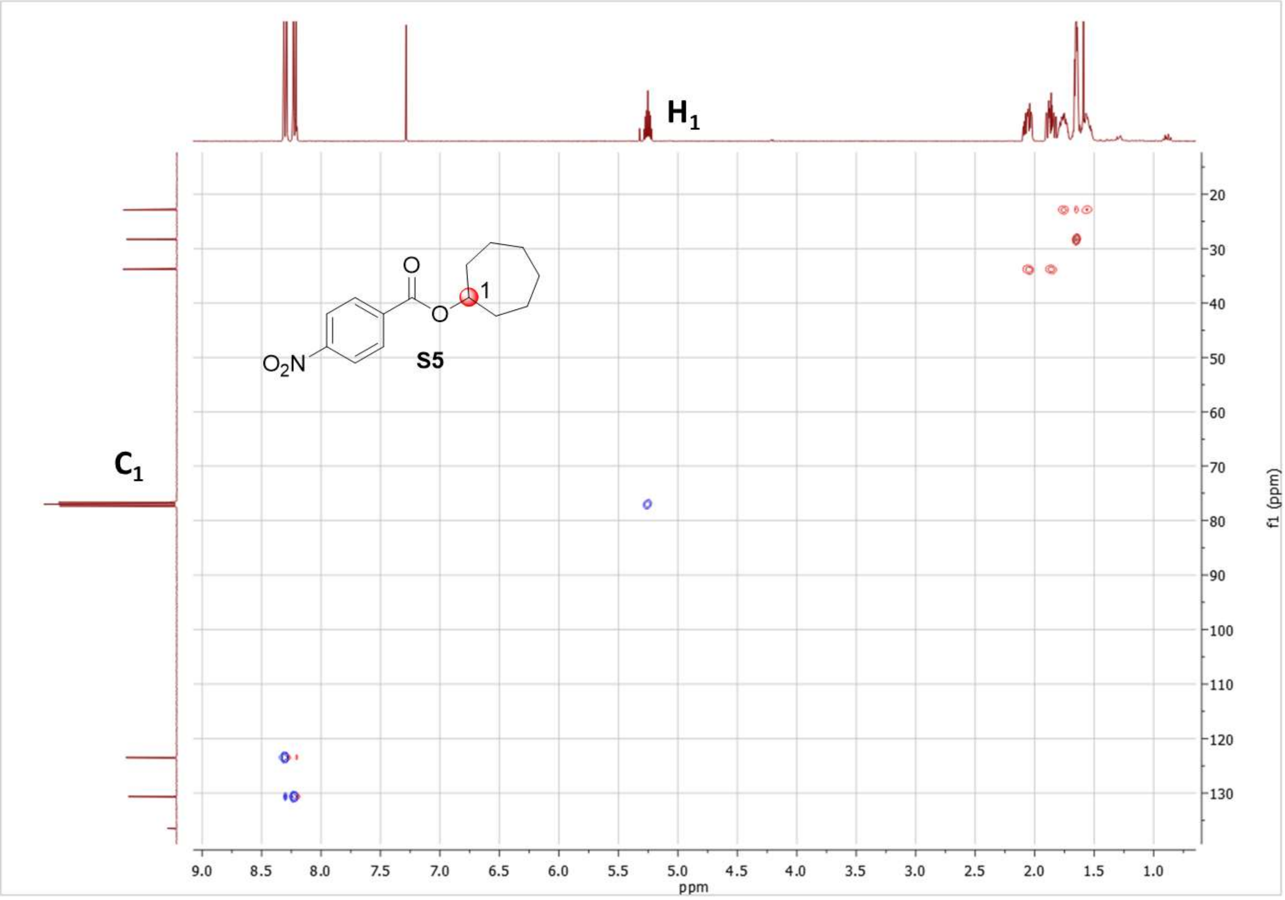

Figure S4. HSQC-NMR spectrum (400 MHz, $CDCl_3$) of cycloheptyl-4-nitrobenzoate (**S5**).

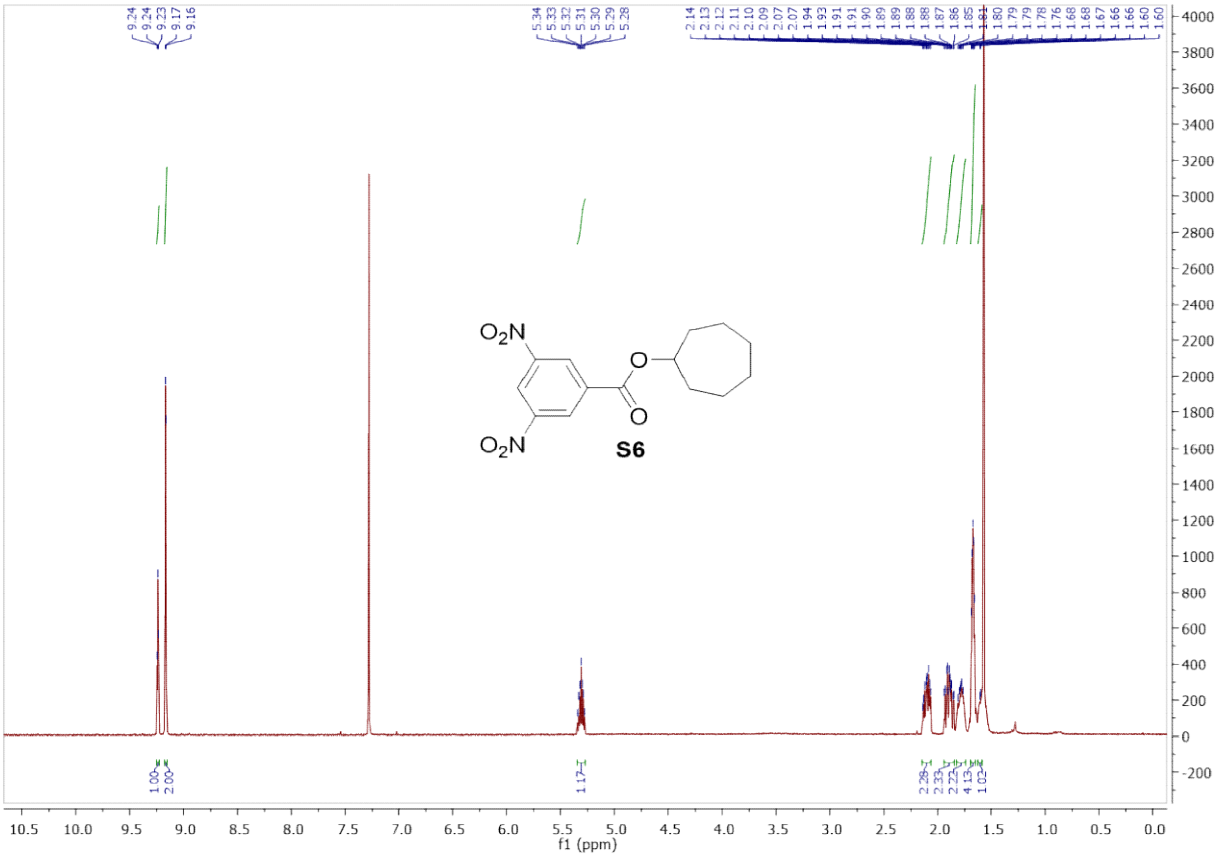

Figure S5. ^{1}H-NMR spectrum (400 MHz, $CDCl_3$) of cycloheptyl-3,5-dinitrobenzoate (**S6**).

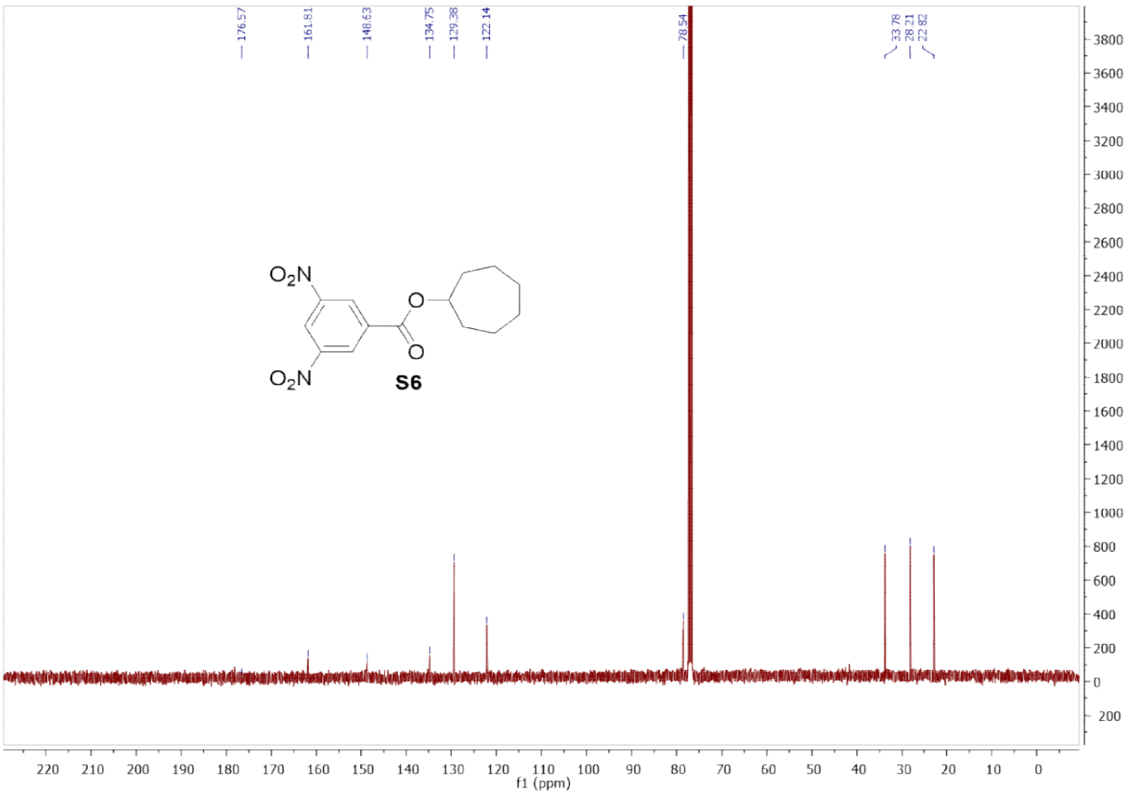

Figure S6. $^{13}C\{^1H\}$-NMR spectrum (400 MHz, $CDCl_3$) of cycloheptyl-3,5-dinitrobenzoate (**S6**).

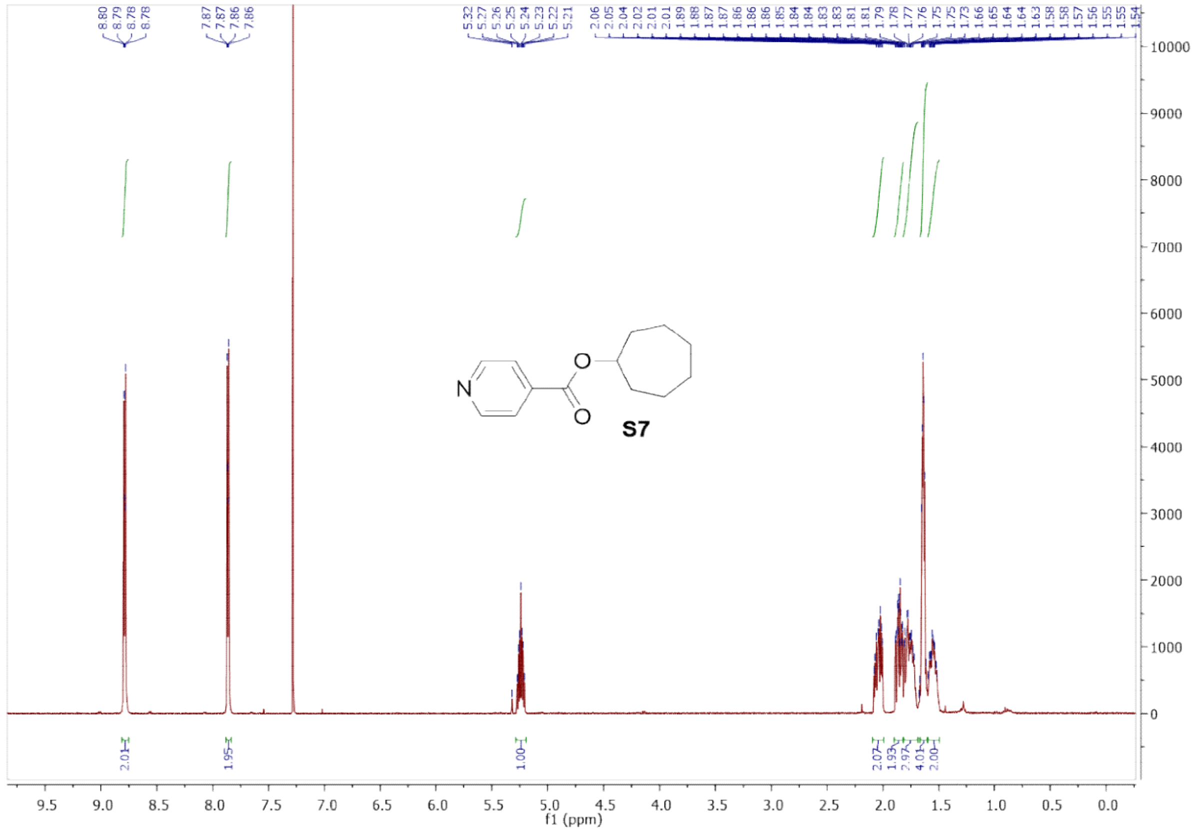

Figure S7. ^{1}H-NMR spectrum (400 MHz, $CDCl_3$) of cycloheptyl 4-pyridinecarboxylate (**S7**).

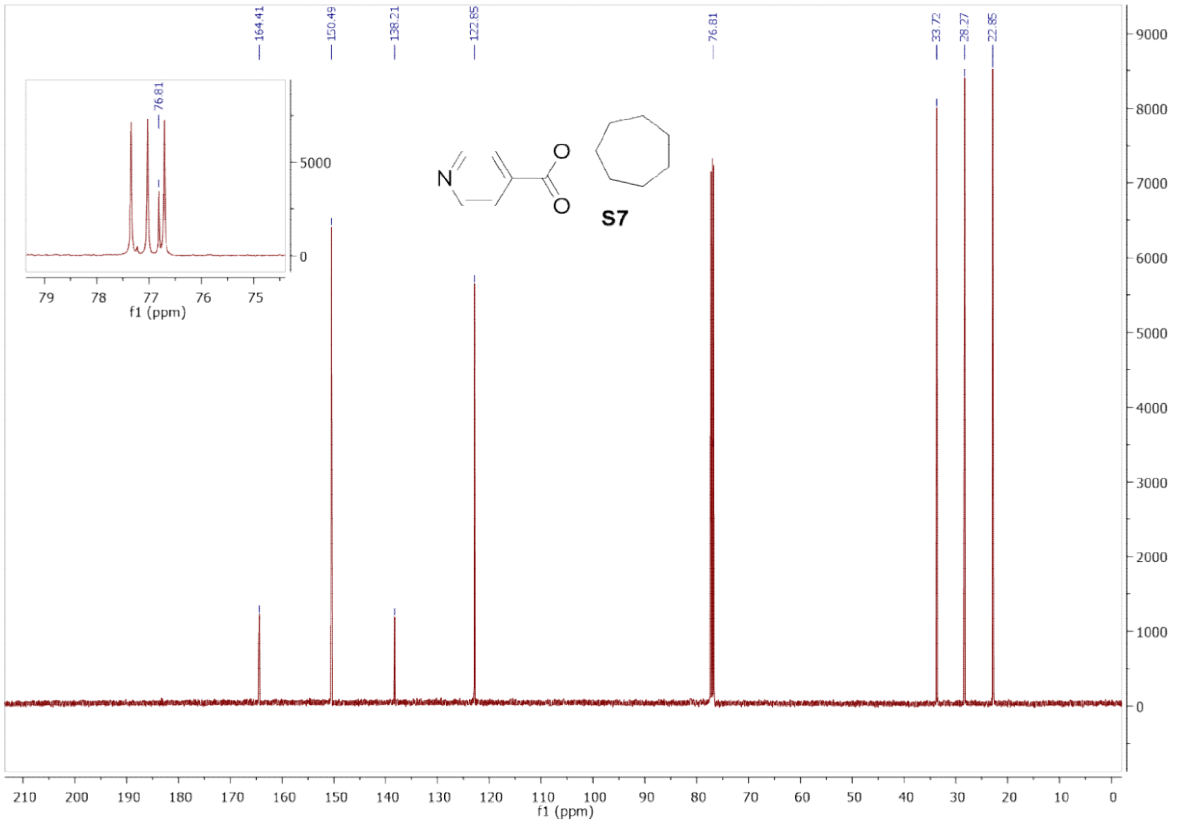

Figure S8. $^{13}C\{^1H\}$-NMR spectrum (400 MHz, $CDCl_3$) of cycloheptyl 4-pyridinecarboxylate (**S7**).

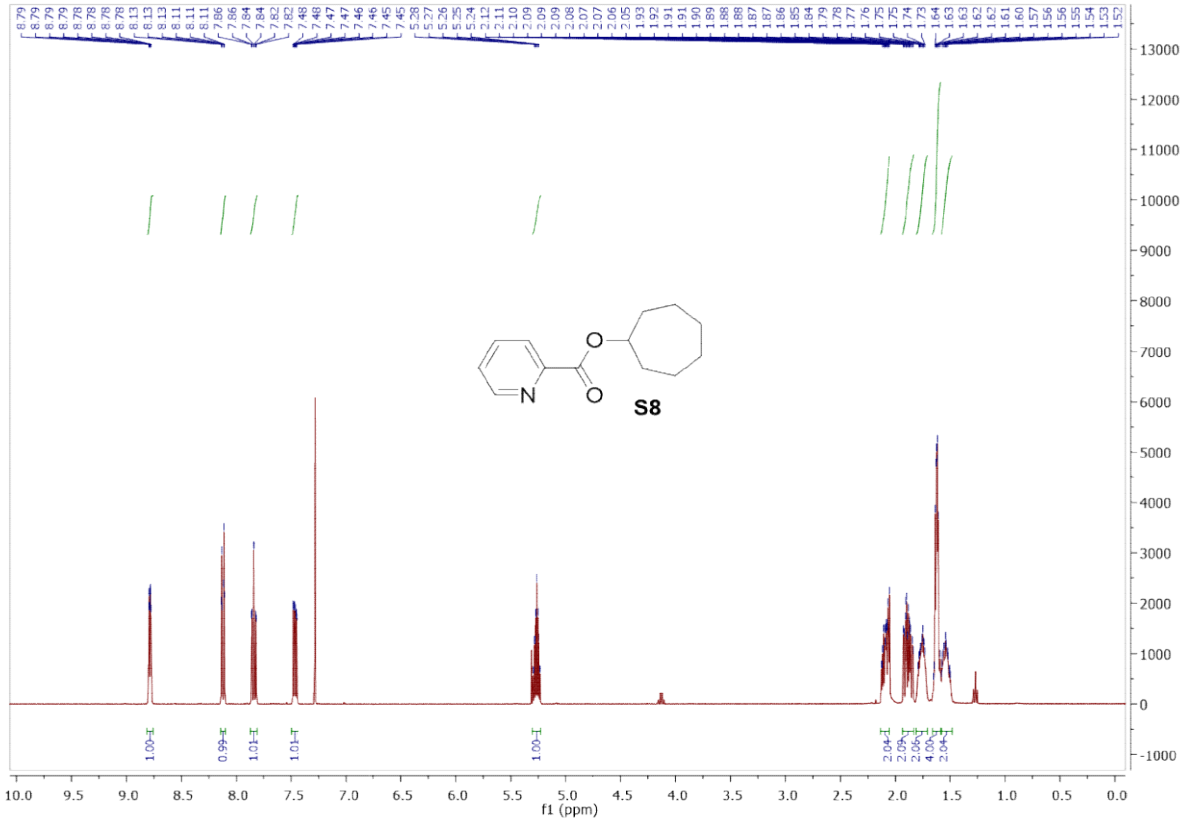

Figure S9. ^{1}H-NMR spectrum (400 MHz, $CDCl_3$) of cycloheptyl 2-pyridinecarboxylate (**S8**).

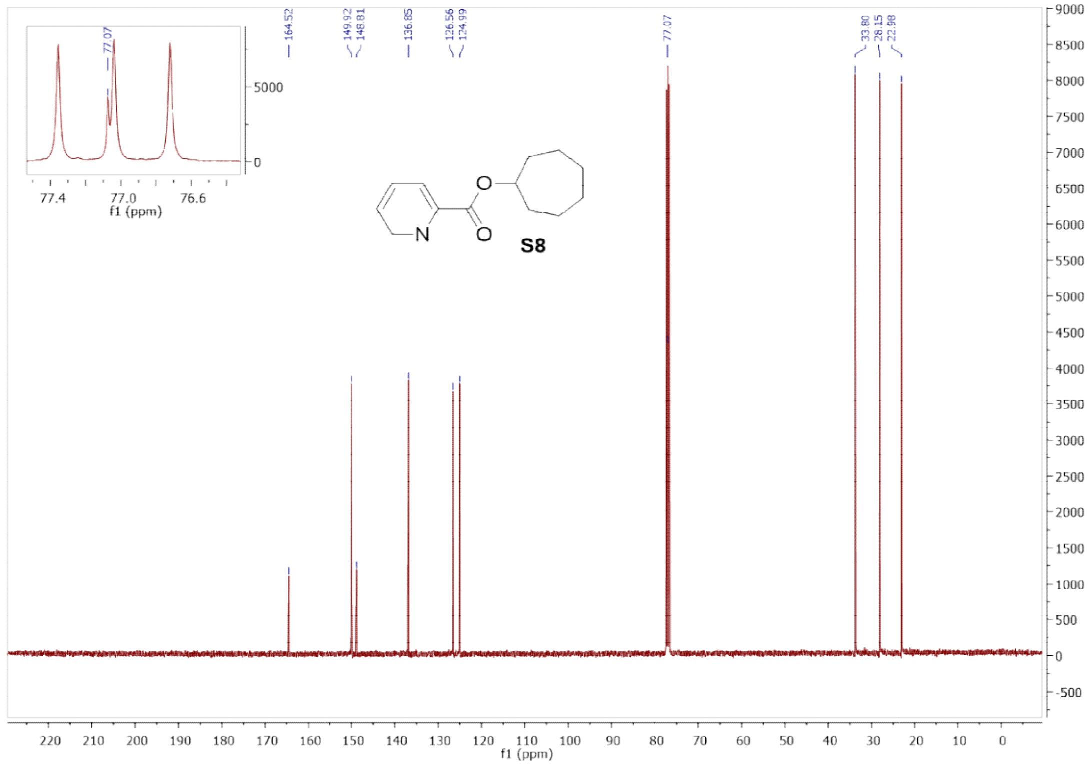

Figure S10. $^{13}C\{^{1}H\}$-NMR spectrum (400 MHz, $CDCl_3$) of cycloheptyl 2-pyridinecarboxylate (**S8**).

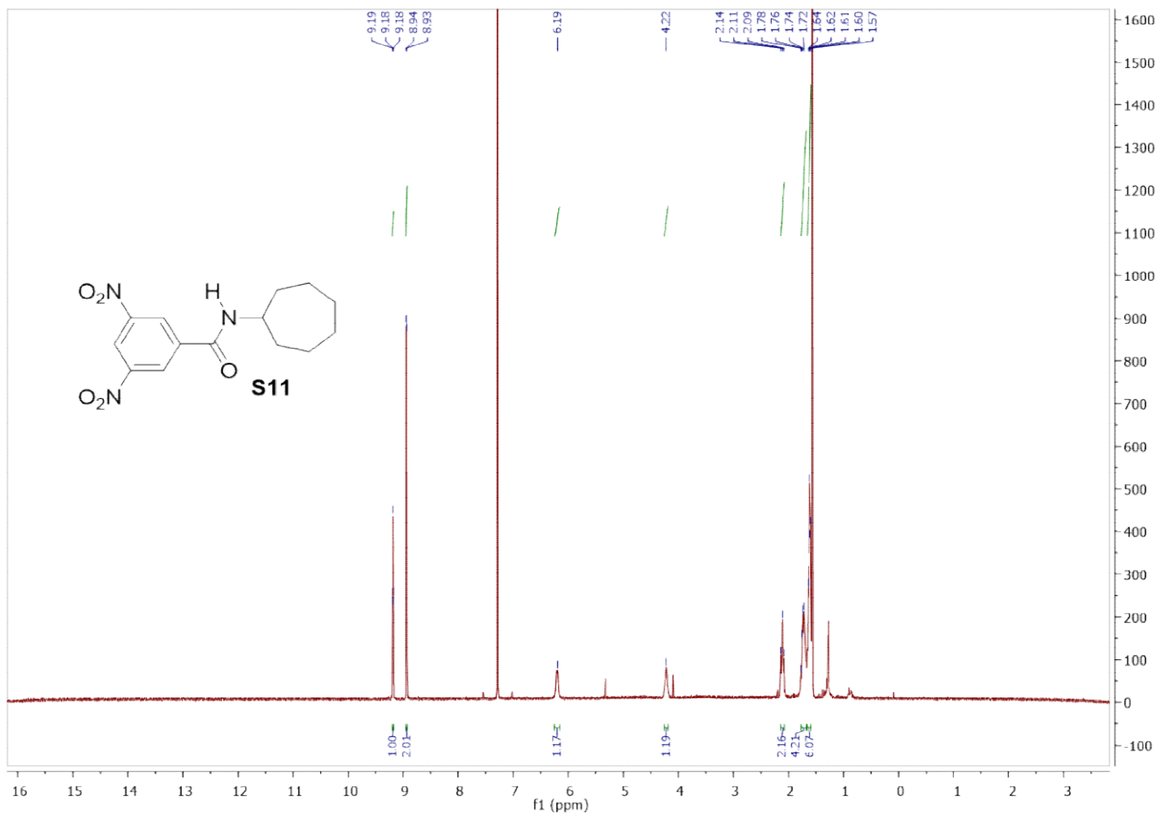

Figure S11. ^{1}H-NMR spectrum (400 MHz, $CDCl_3$) of *N*-cycloheptyl-3,5-dinitrobenzamide (**S11**).

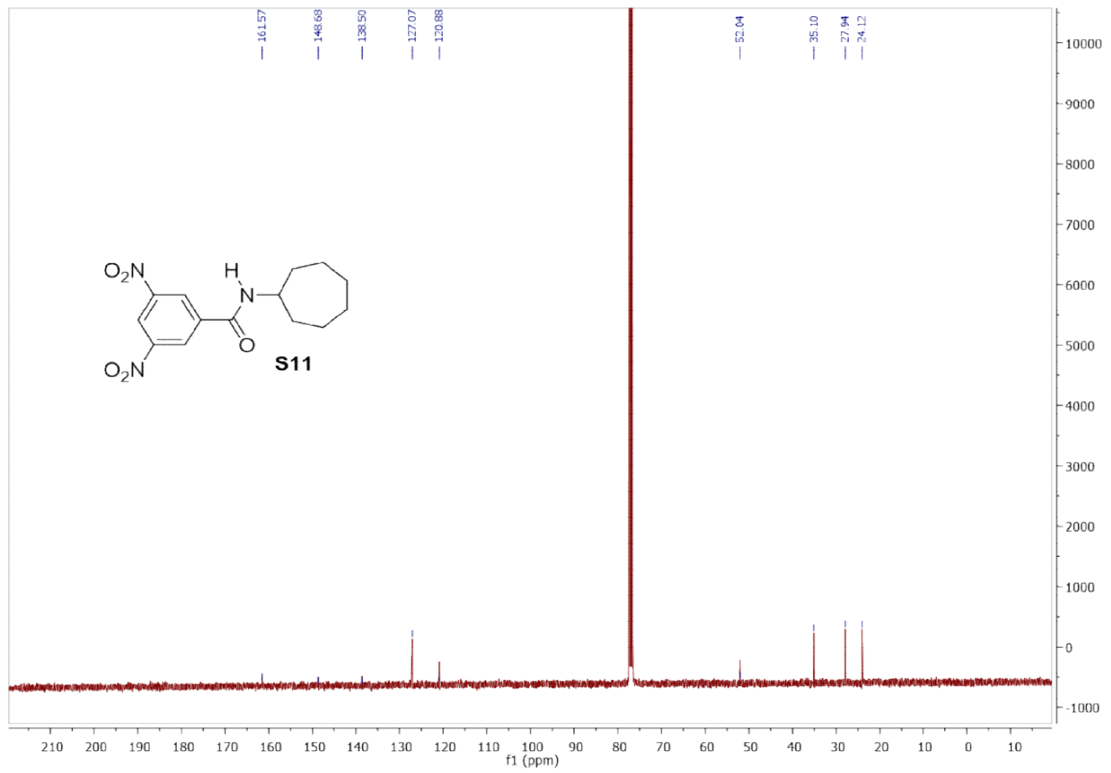

Figure S12. $^{13}C\{^{1}H\}$-NMR spectrum (400 MHz, $CDCl_3$) of *N*-cycloheptyl-3,5-dinitrobenzamide (**S11**).

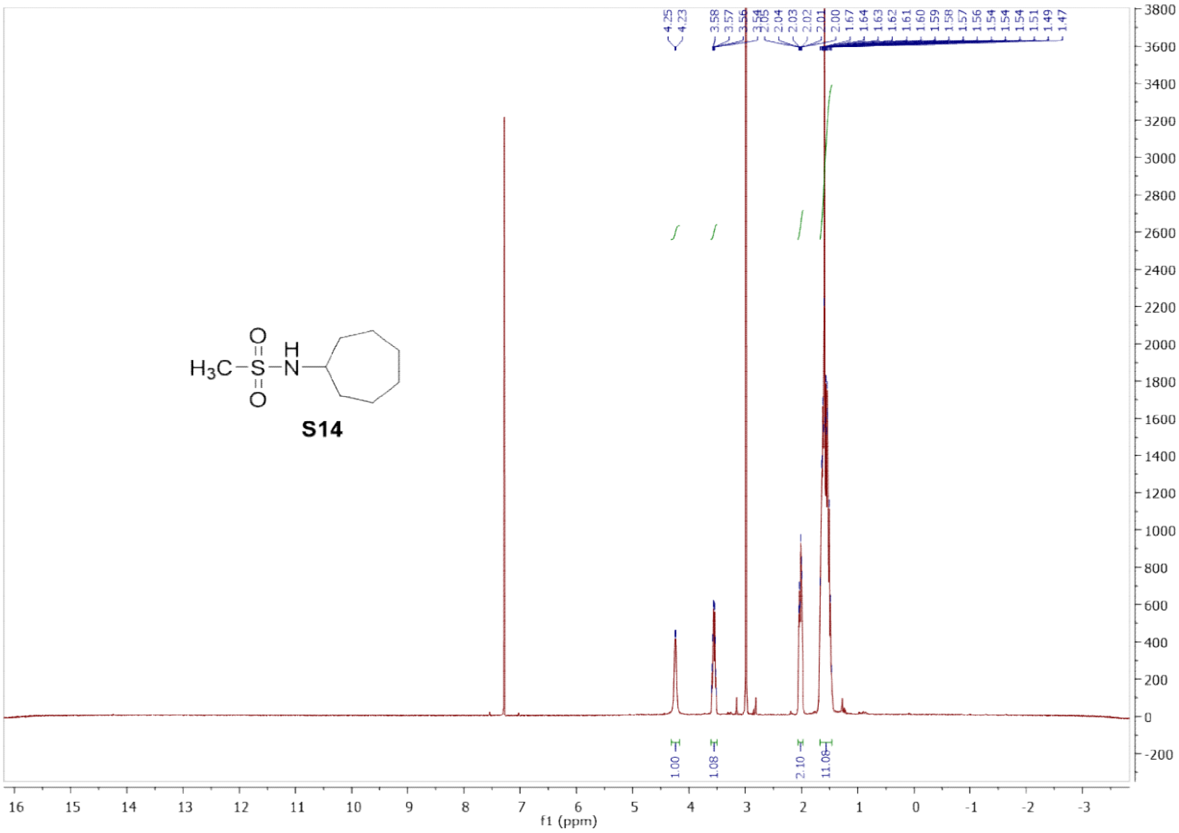

Figure S13. ^{1}H-NMR spectrum (400 MHz, $CDCl_3$) of *N*-cycloheptylmethanesulfonamide (**S14**).

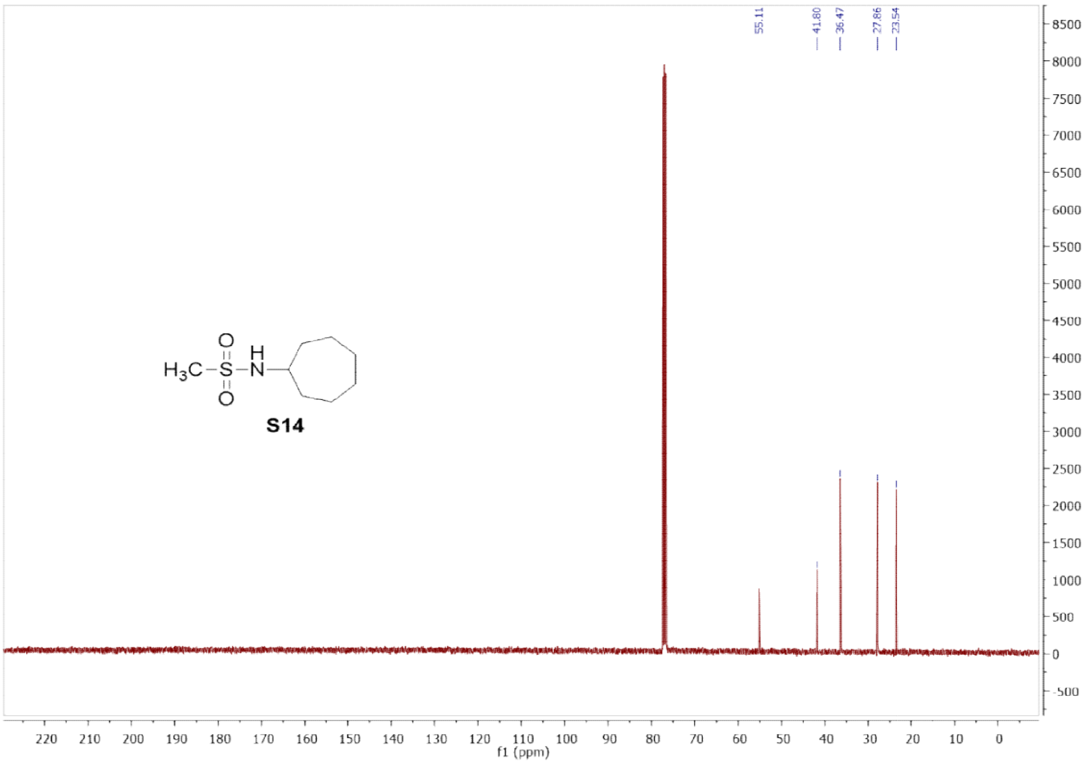

Figure S14. $^{13}C\{^{1}H\}$-NMR spectrum (400 MHz, $CDCl_3$) of *N*-cycloheptylmethanesulfonamide (**S14**).

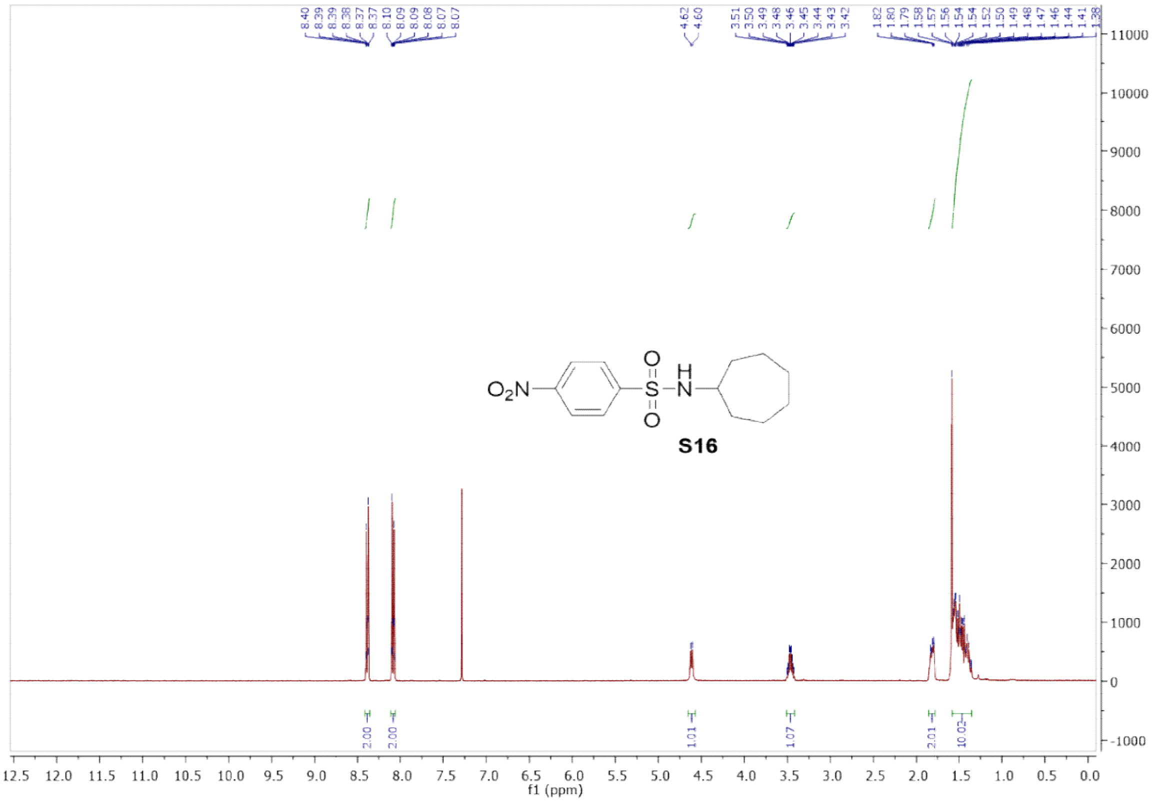

Figure S15. ^{1}H-NMR spectrum (400 MHz, $CDCl_3$) of *N*-cycloheptyl-4-nitrobenzenesulfonamide (**S16**).

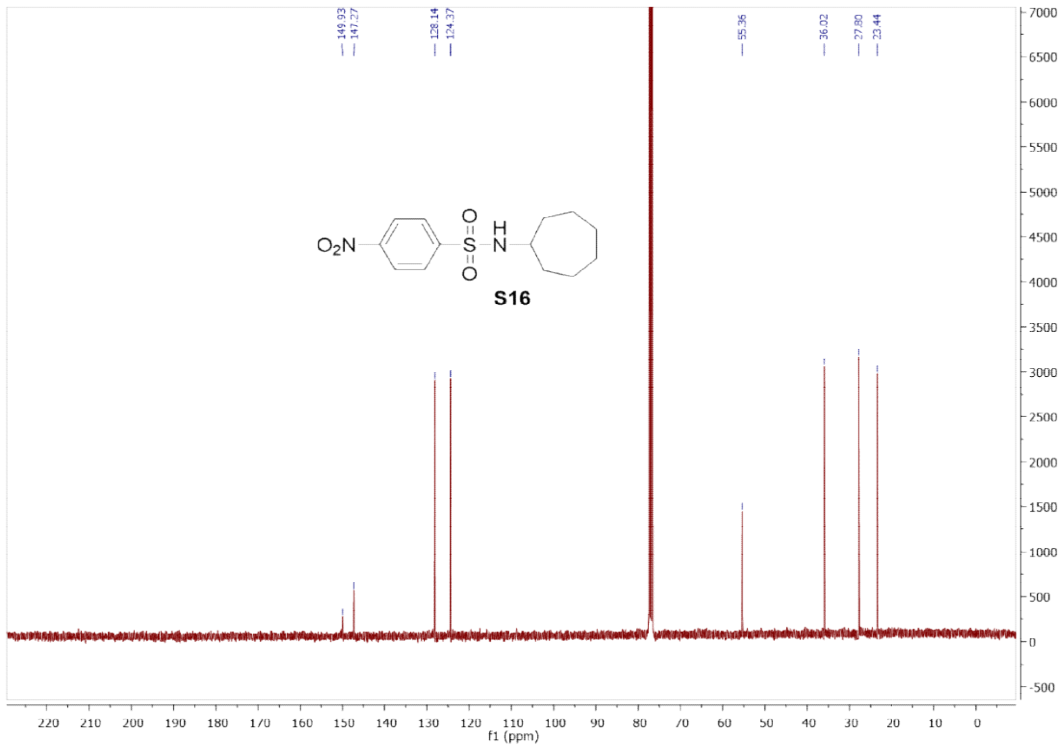

Figure S16. $^{13}C\{^{1}H\}$-NMR spectrum (400 MHz, $CDCl_3$) of *N*-cycloheptyl-4-nitrobenzenesulfonamide (**S16**).

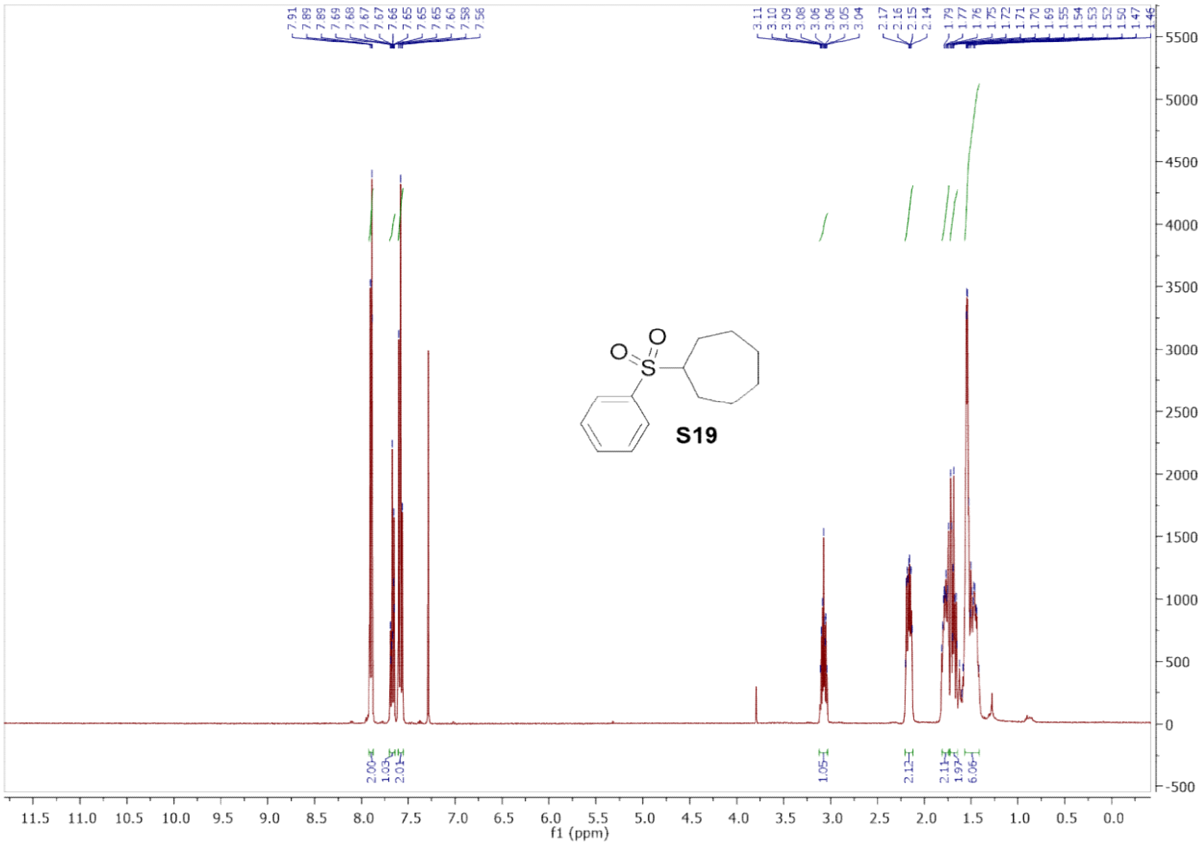

Figure S17. ^{1}H-NMR spectrum (400 MHz, $CDCl_3$) of (phenylsulfonyl)cycloheptane (**S19**).

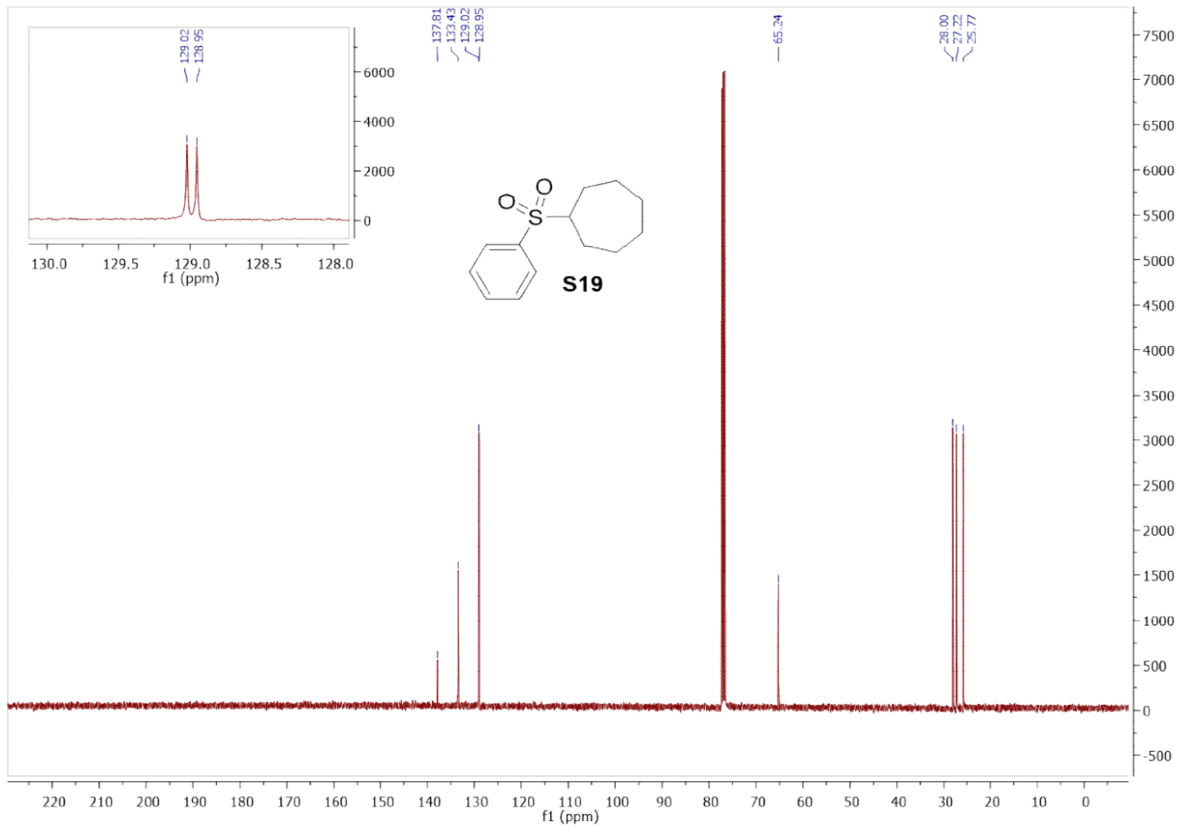

Figure S18. $^{13}C\{^{1}H\}$-NMR spectrum (400 MHz, $CDCl_3$) of (phenylsulfonyl)cyclohpetane (**S19**).

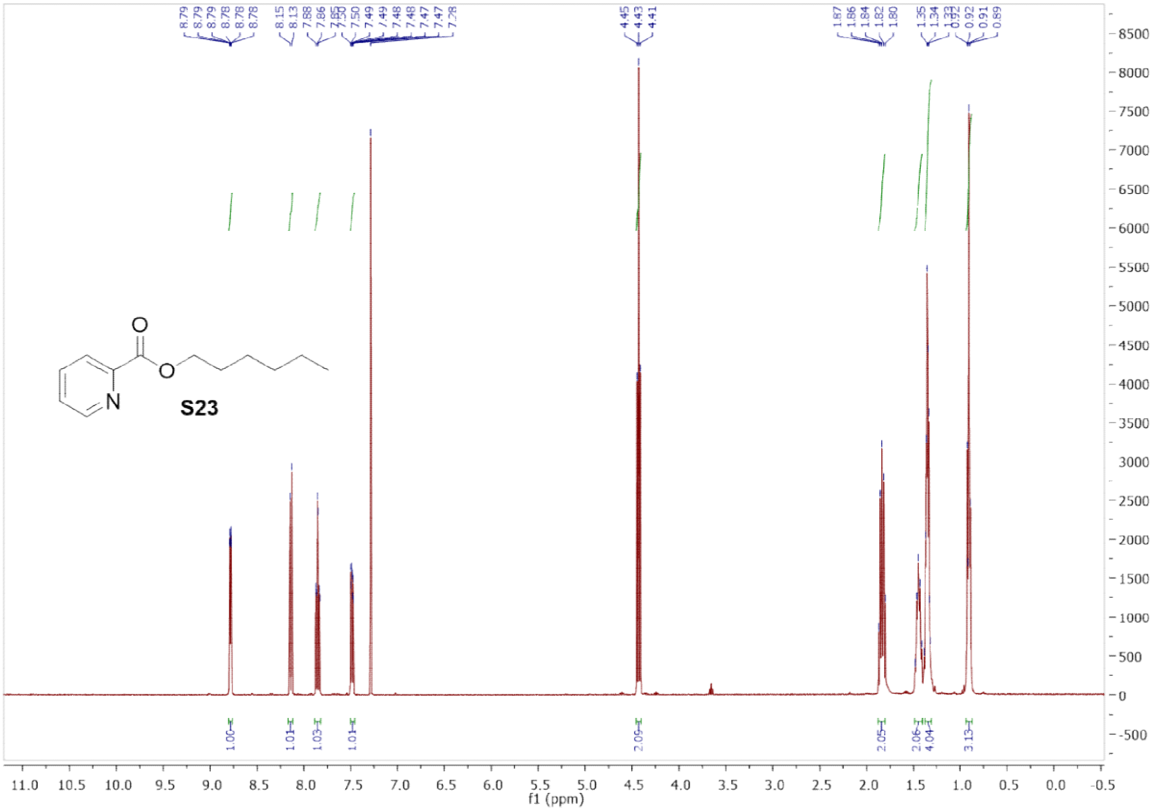

Figure S19. ^{1}H-NMR spectrum (400 MHz, $CDCl_3$) of 1-hexyl 2-pyridinecarboxylate (**S23**).

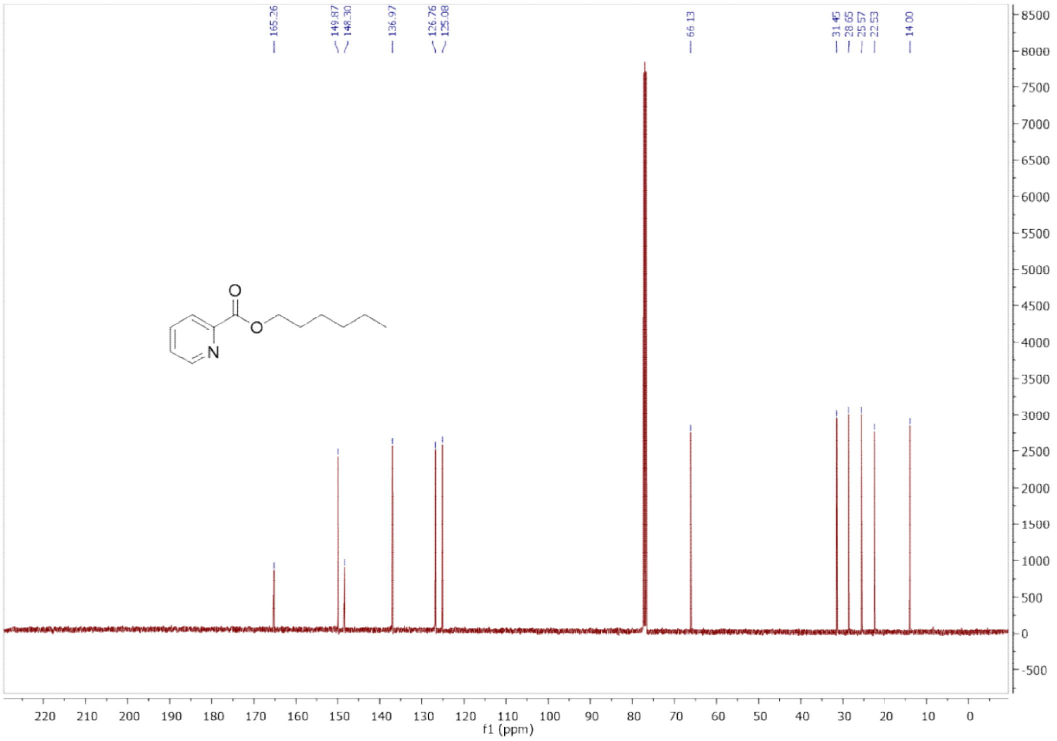

Figure S20. $^{13}C\{^{1}H\}$-NMR spectrum (400 MHz, $CDCl_3$) of 1-hexyl 2-pyridinecarboxylate (**S23**).

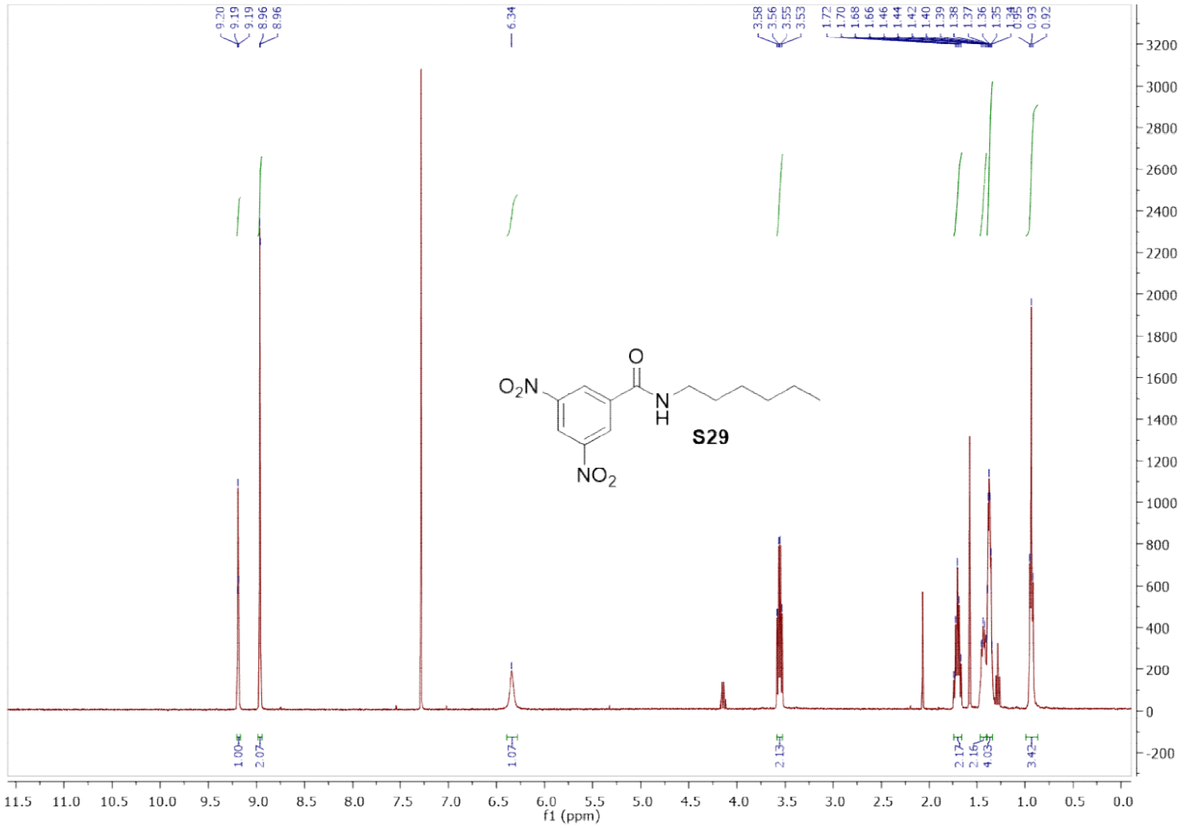

Figure S21. ^{1}H-NMR spectrum (400 MHz, $CDCl_3$) of *N*-hexyl-3,5-dinitrobenzamide (**S29**).

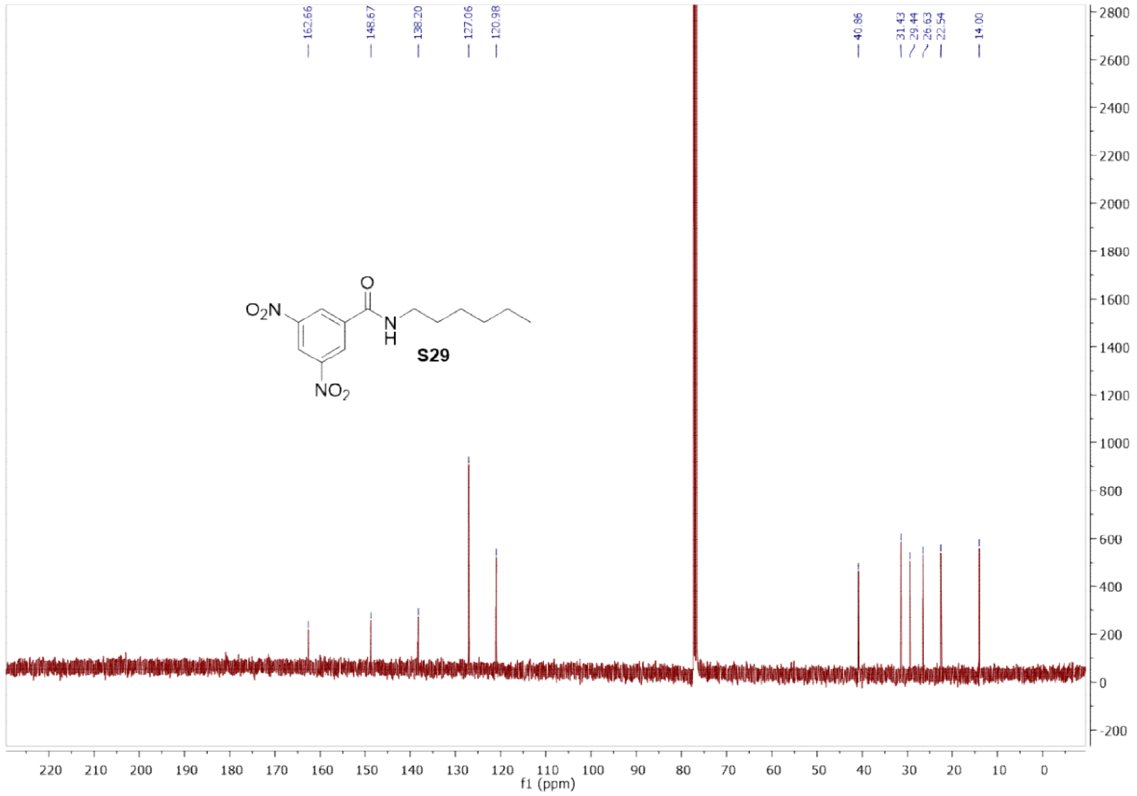

Figure S22. $^{13}C\{^{1}H\}$-NMR spectrum (400 MHz, $CDCl_3$) of *N*-hexyl-3,5-dinitrobenzamide (**S29**).

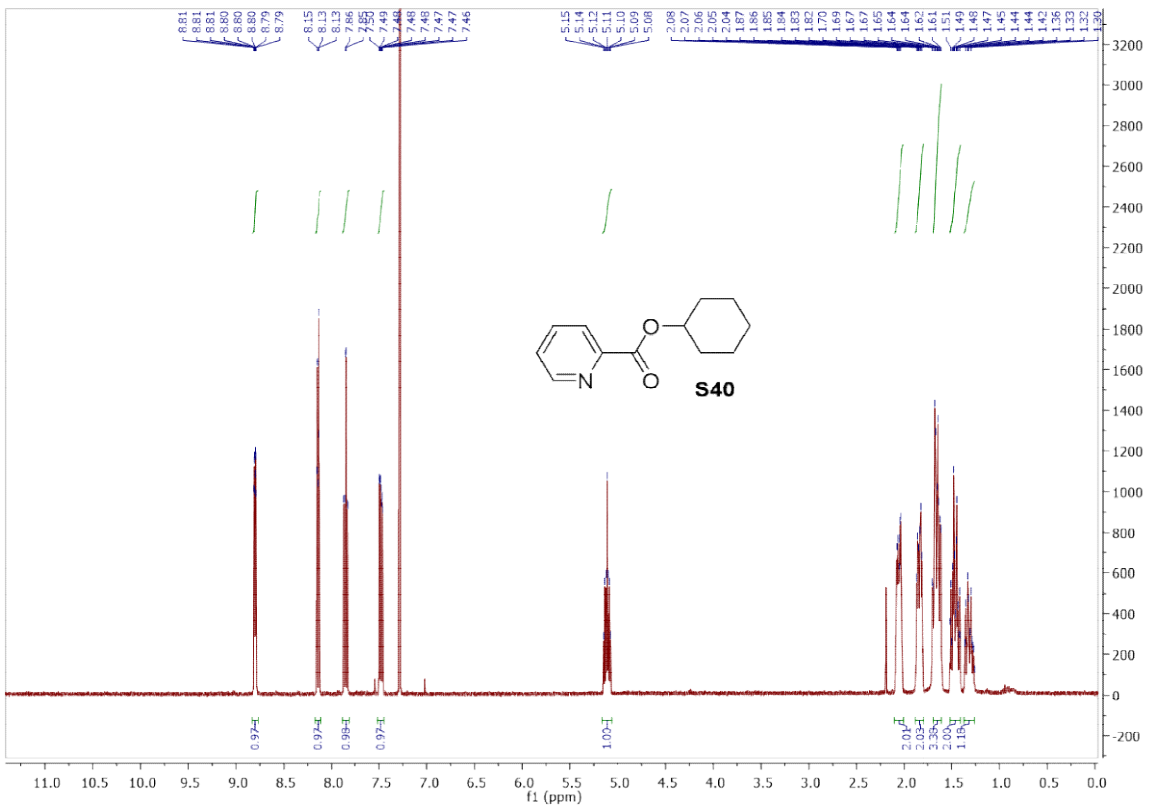

Figure S23. ^{1}H-NMR spectrum (400 MHz, $CDCl_3$) of cyclohexyl 2-pyridinecarboxylate (**S40**).

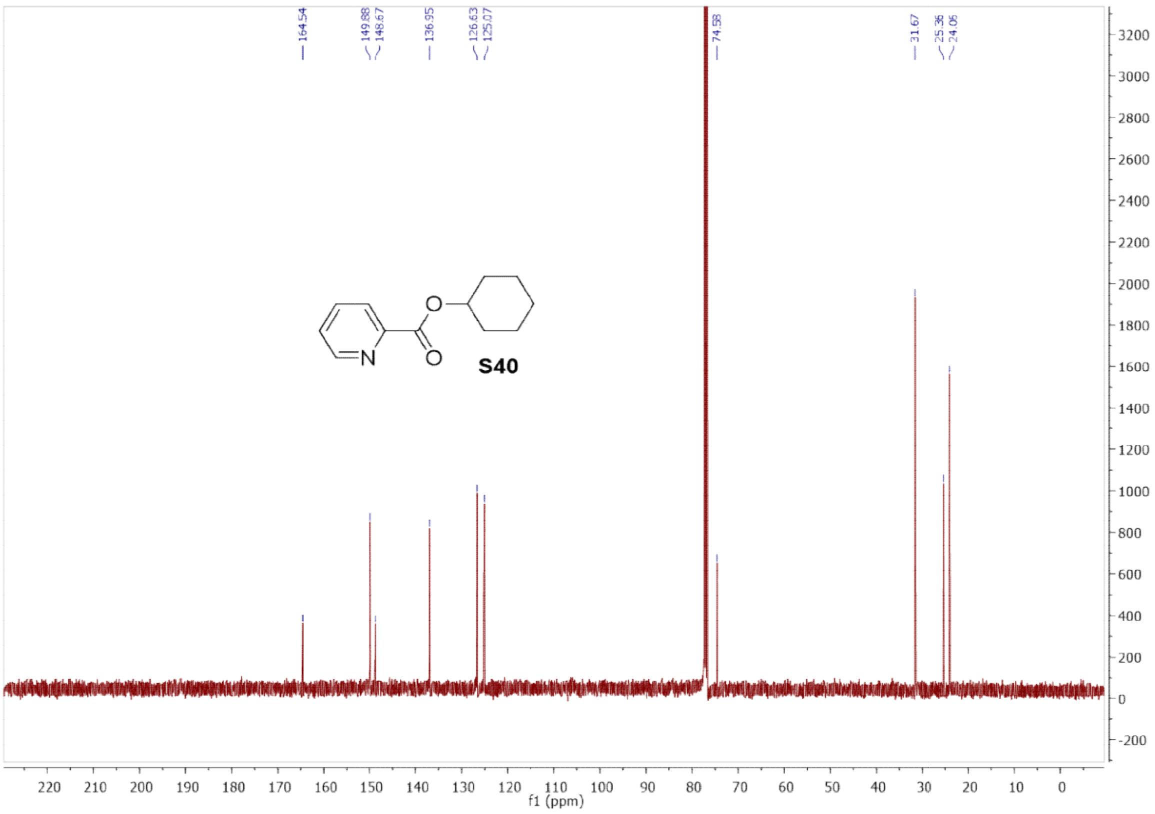

Figure S24. $^{13}C\{^{1}H\}$-NMR spectrum (400 MHz, $CDCl_3$) of cyclohexyl 2-pyridinecarboxylate (**S40**).

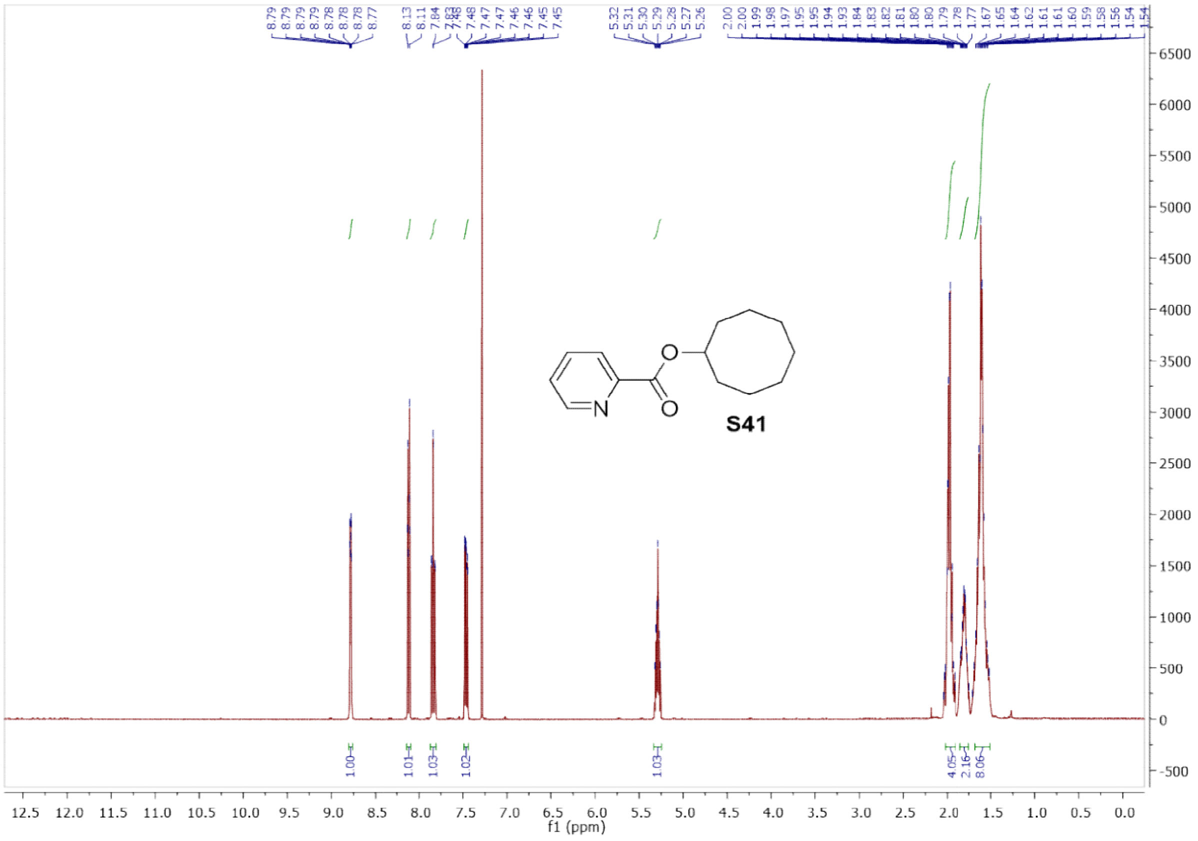

Figure S25. ^{1}H-NMR spectrum (400 MHz, $CDCl_3$) of cyclooctyl 2-pyridinecarboxylate (**S41**).

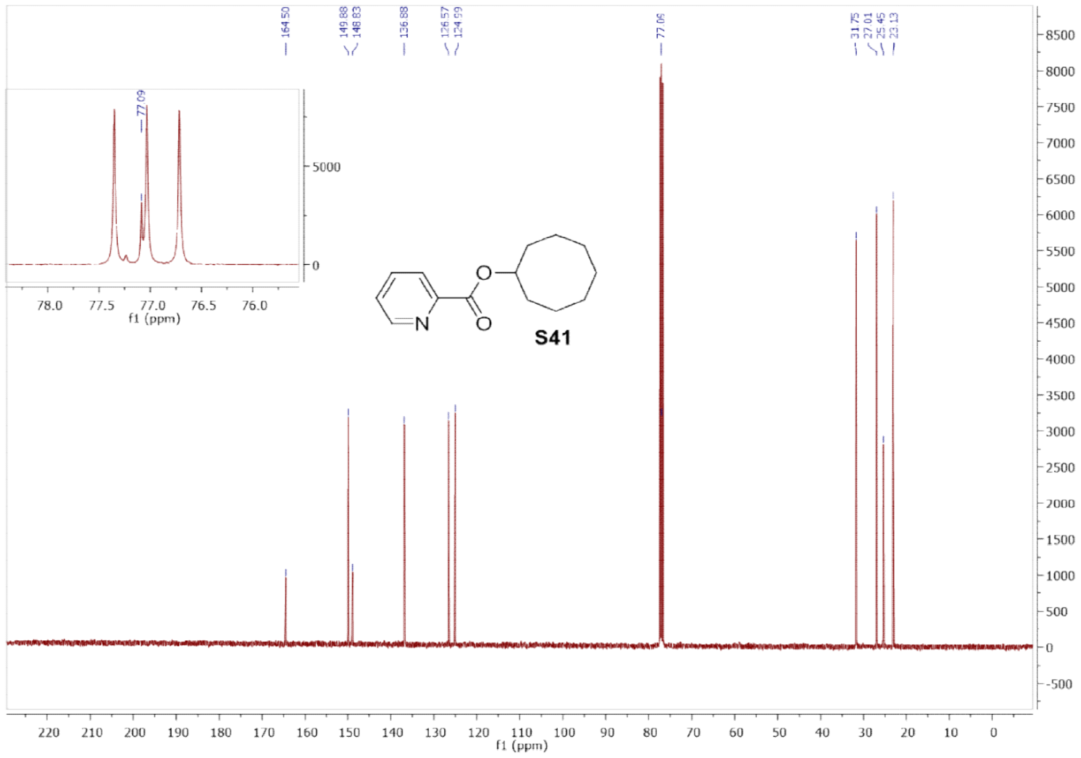

Figure S26. $^{13}C\{^{1}H\}$-NMR spectrum (400 MHz, $CDCl_3$) of cyclooctyl 2-pyridinecarboxylate (**S41**).

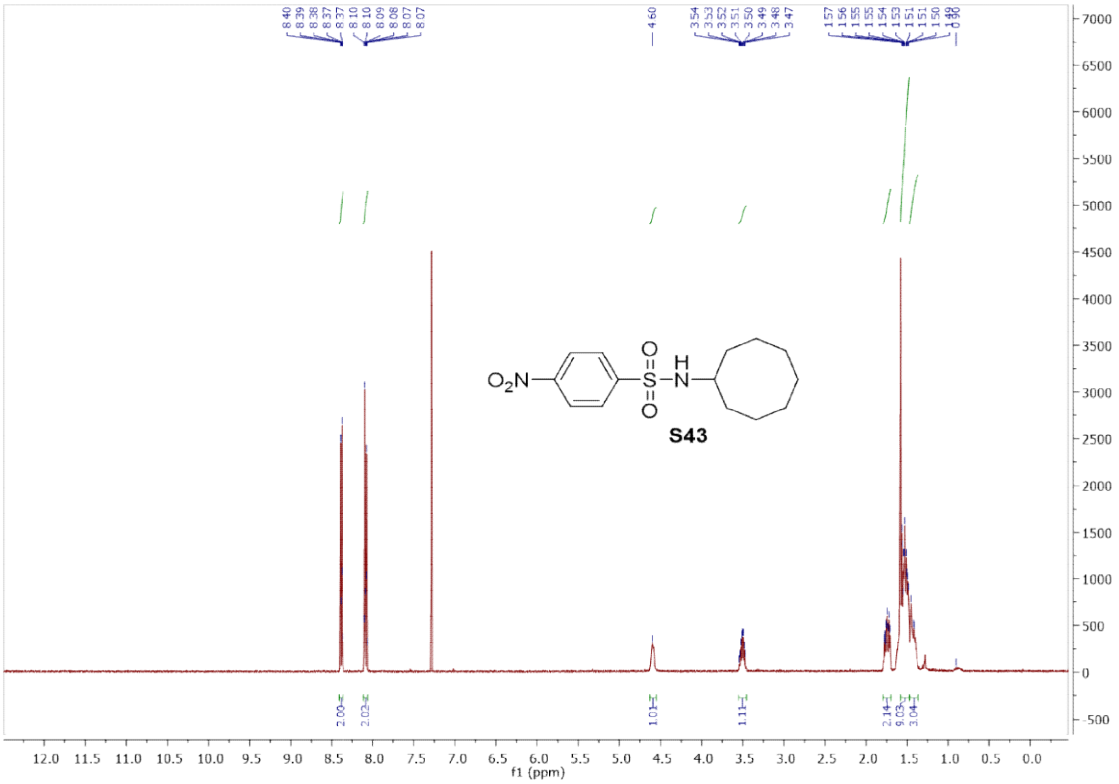

Figure S27. ^{1}H-NMR spectrum (400 MHz, $CDCl_3$) of *N*-cyclooctyl-4-nitrobenzenesulfonamide (**S43**).

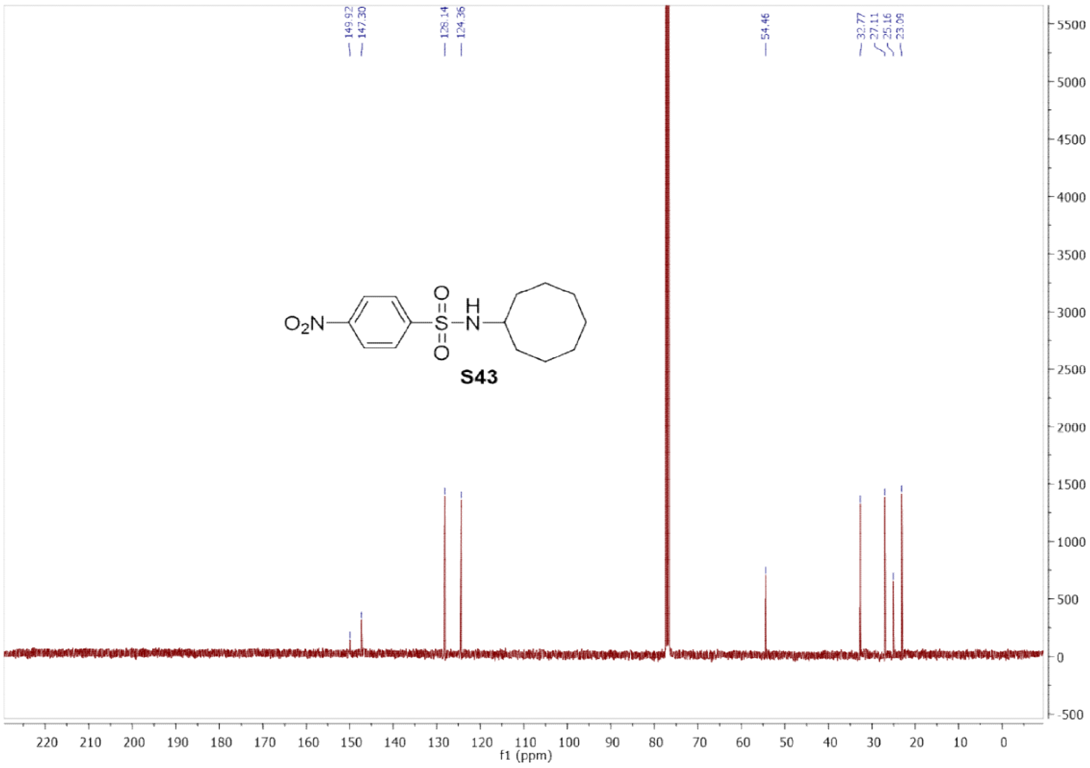

Figure S28. $^{13}C\{^1H\}$-NMR spectrum (400 MHz, $CDCl_3$) of *N*-cyclooctyl-4-nitrobenzenesulfonamide (**S43**).

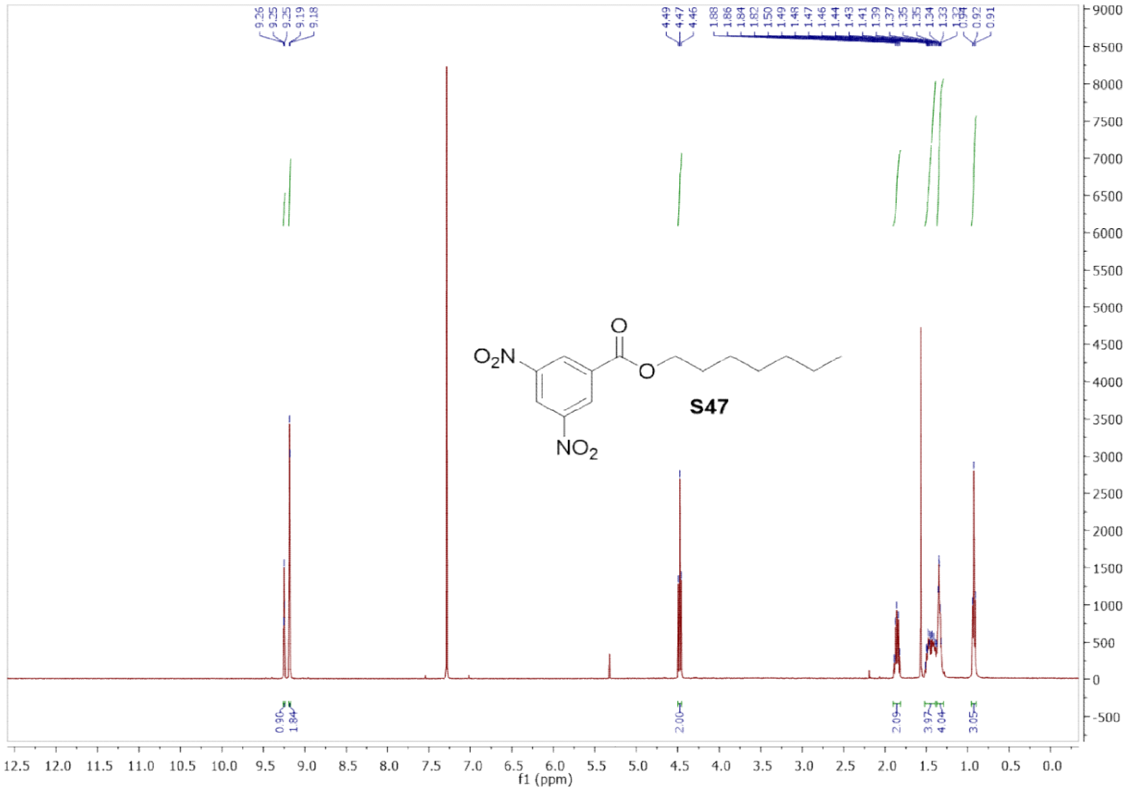

Figure S29. ^{1}H-NMR spectrum (400 MHz, $CDCl_3$) of 1-heptyl-3,5-dinitrobenzoate (**S47**).

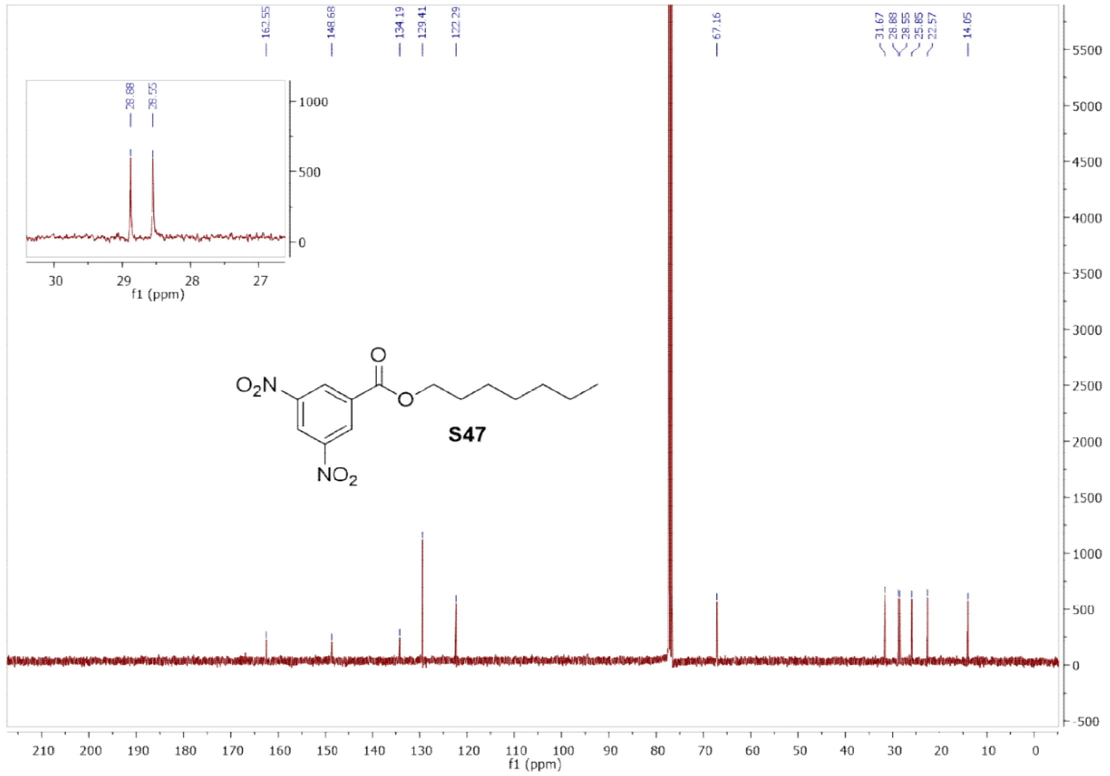

Figure S30. ^{13}C{^{1}H}-NMR spectrum (400 MHz, $CDCl_3$) of 1-heptyl-3,5-dinitrobenzoate (**S47**).

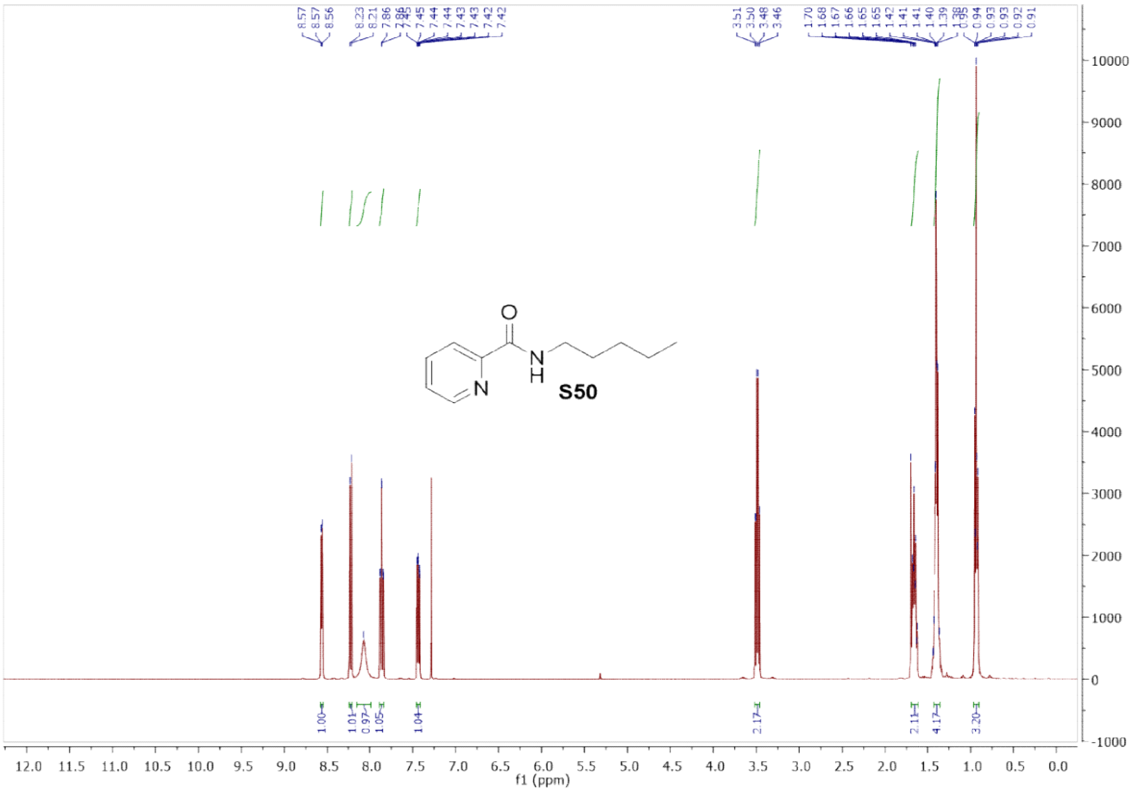

Figure S31. ^{1}H-NMR spectrum (400 MHz, $CDCl_3$) of *N*-pentyl 2-pyridinecarboxamide (**S50**).

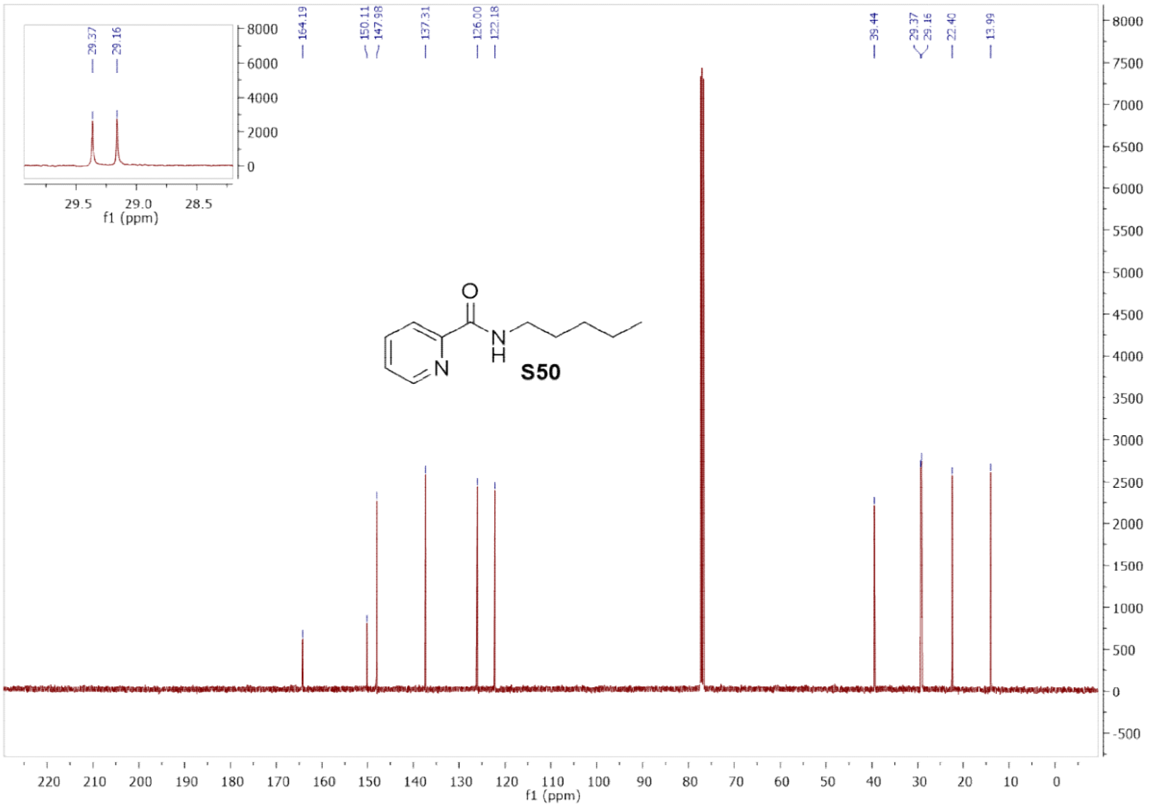

Figure S32. $^{13}C\{^{1}H\}$-NMR spectrum (400 MHz, $CDCl_3$) of *N*-pentyl 2-pyridinecarboxamide (**S50**).

6.2. NMR spectra of the isolated oxidation products

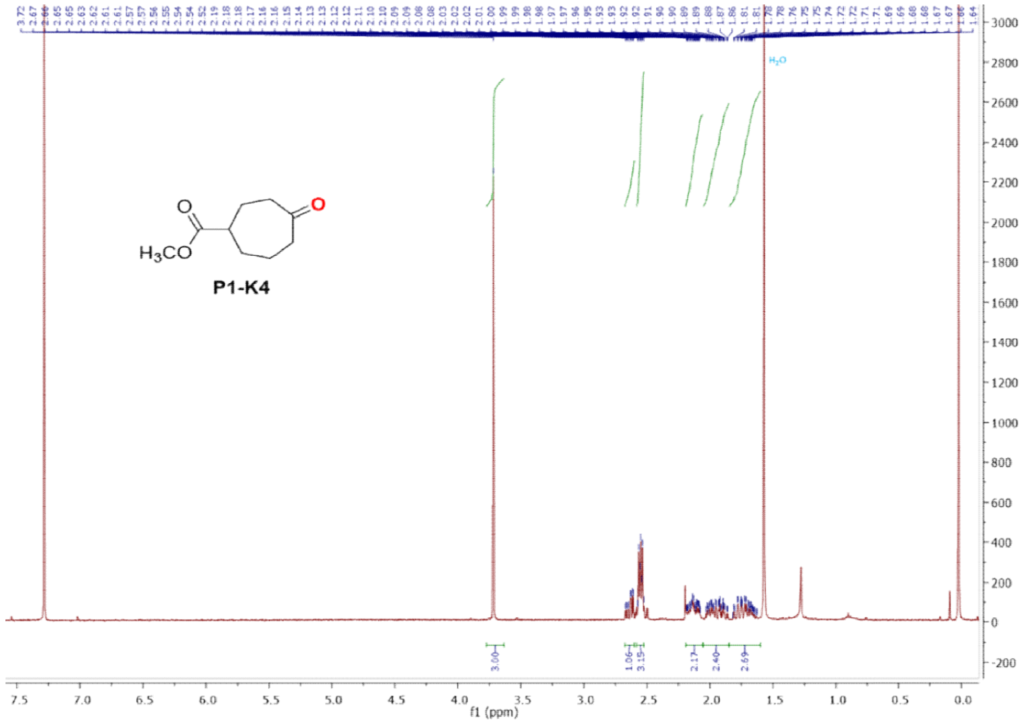

Figure S33. ^{1}H-NMR spectrum (400 MHz, $CDCl_3$) of methyl 4-oxocycloheptane-1-carboxylate (**P1-K4**).

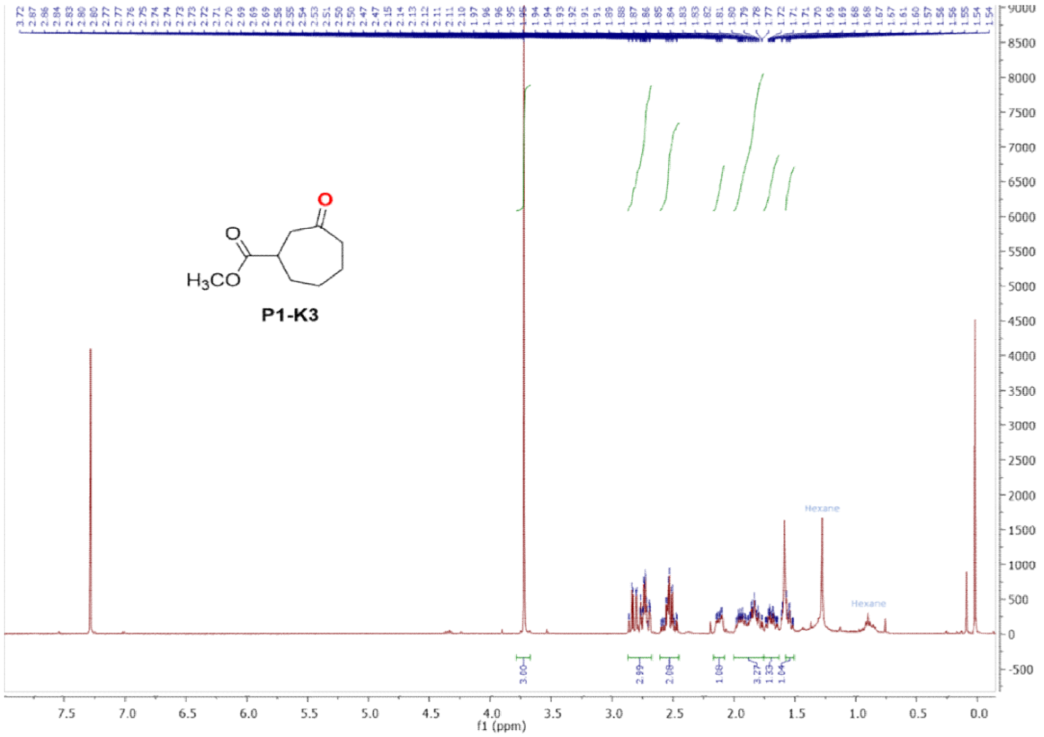

Figure S34. ^{1}H-NMR spectrum (400 MHz, $CDCl_3$) of methyl 3-oxocycloheptane-1-carboxylate (**P1-K3**).

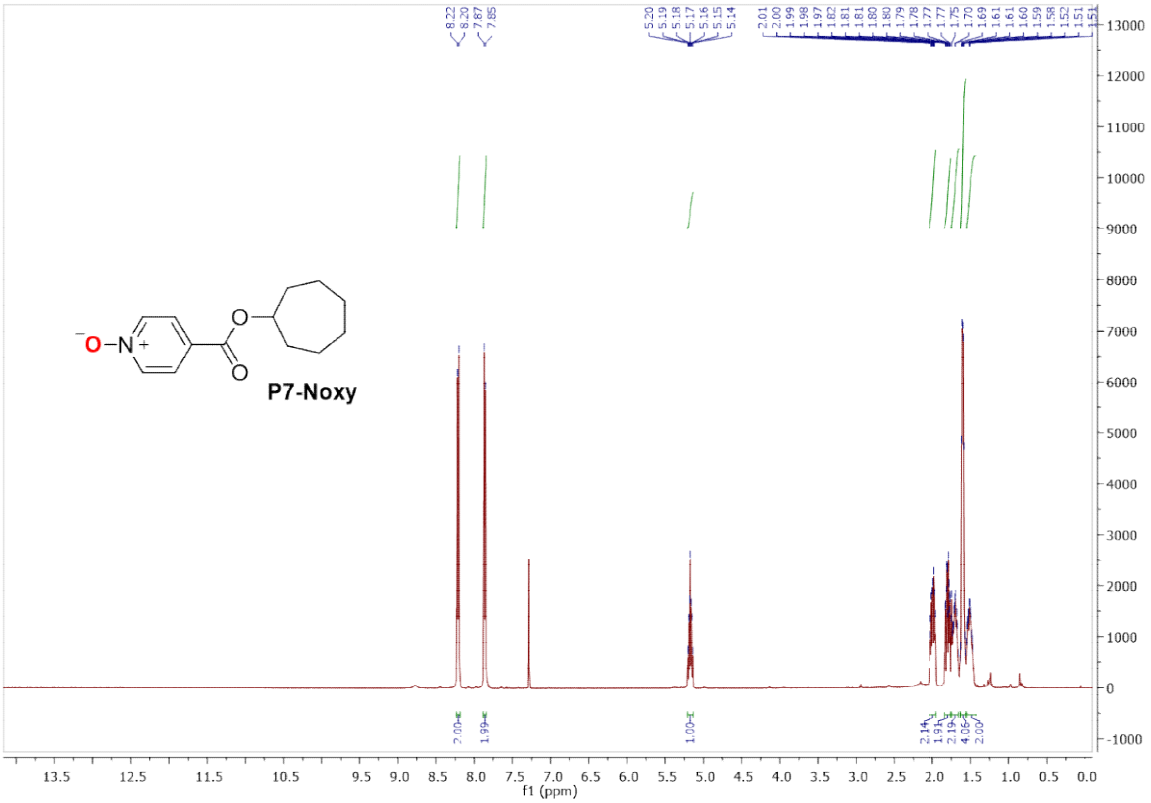

Figure S35. ^{1}H-NMR spectrum (400 MHz, $CDCl_3$) of 4-((cycloheptyloxy)carbonyl)pyridine 1-oxide (**P7-Noxy**).

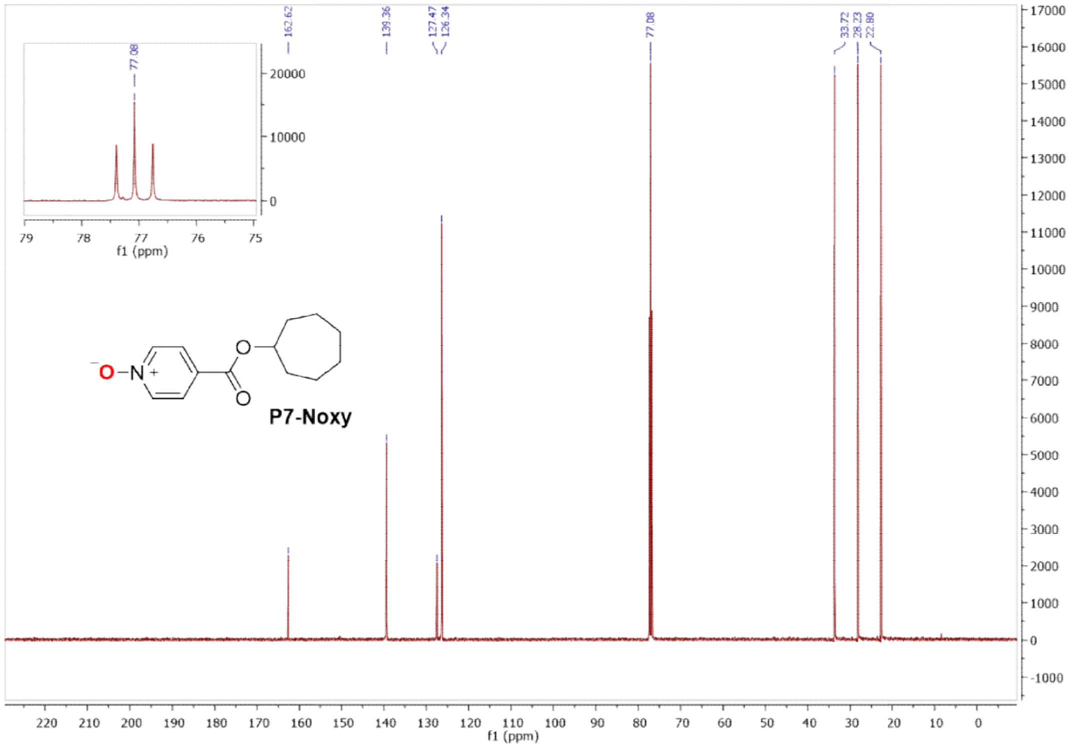

Figure S36. $^{13}C\{^{1}H\}$-NMR spectrum (400 MHz, $CDCl_3$) of 4-((cycloheptyloxy)carbonyl)pyridine 1-oxide (**P7-Noxy**).

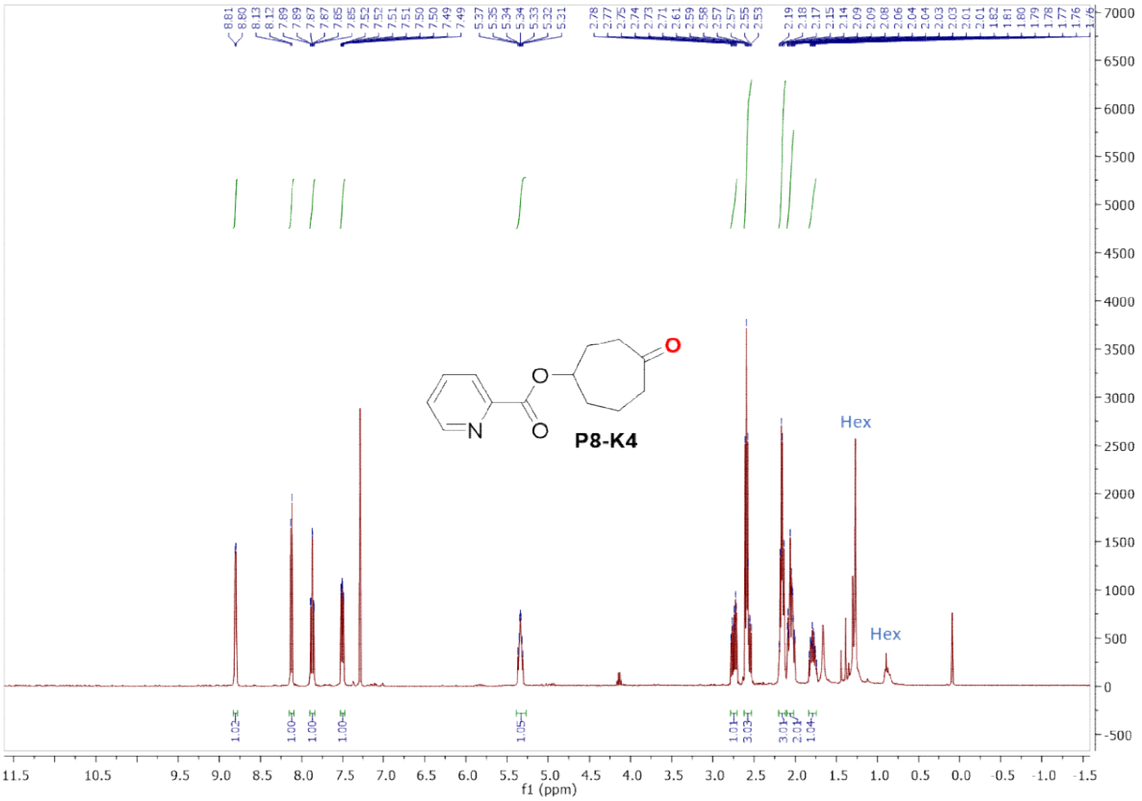

Figure S37. ^{1}H-NMR spectrum (400 MHz, $CDCl_3$) of 4-oxocycloheptyl 2-pyridinecarboxylate (**P8-K4**).

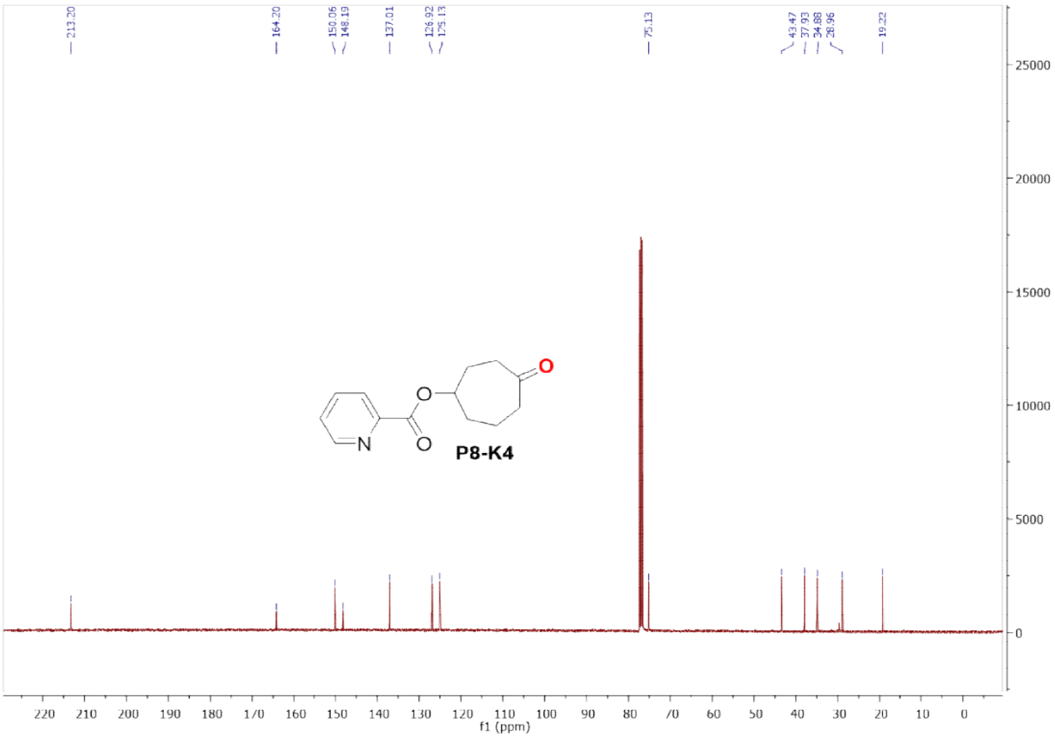

Figure S38. $^{13}C\{^{1}H\}$-NMR spectrum (400 MHz, $CDCl_3$) of 4-oxocycloheptyl 2-pyridinecarboxylate (**P8-K4**).

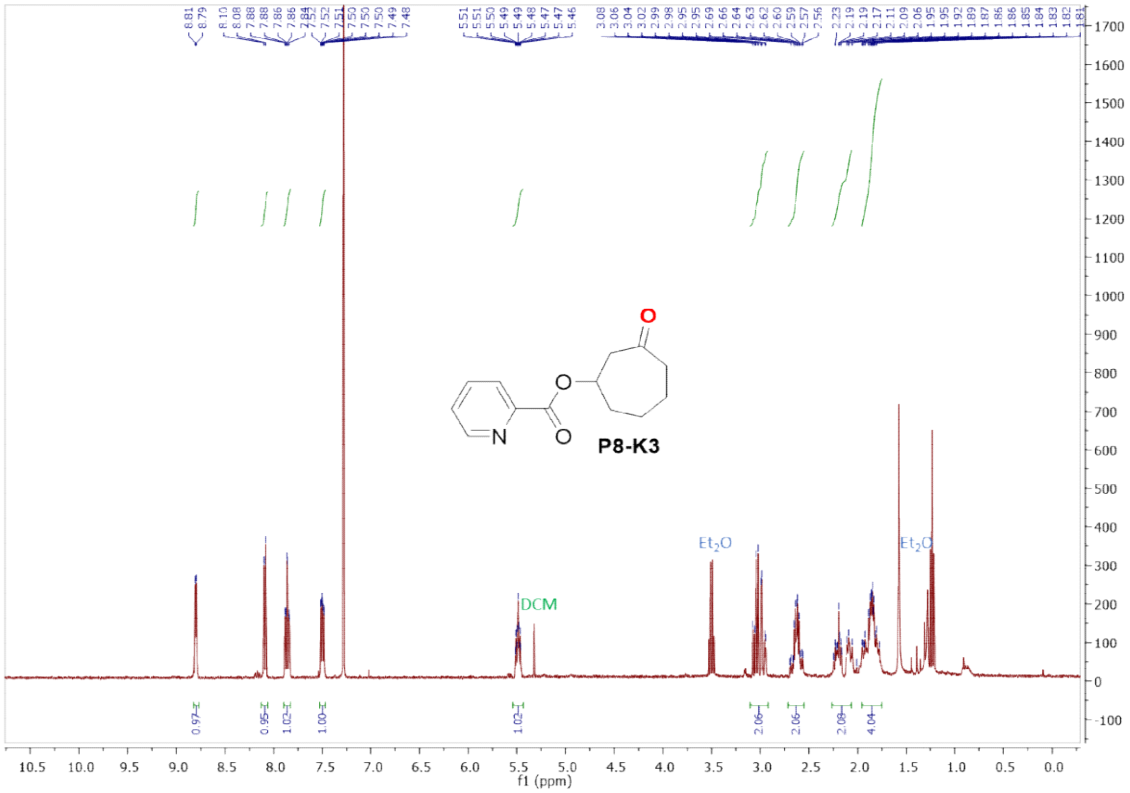

Figure S39. ^{1}H-NMR spectrum (400 MHz, $CDCl_3$) of 3-oxocycloheptyl 2-pyridinecarboxylate (**P8-K3**).

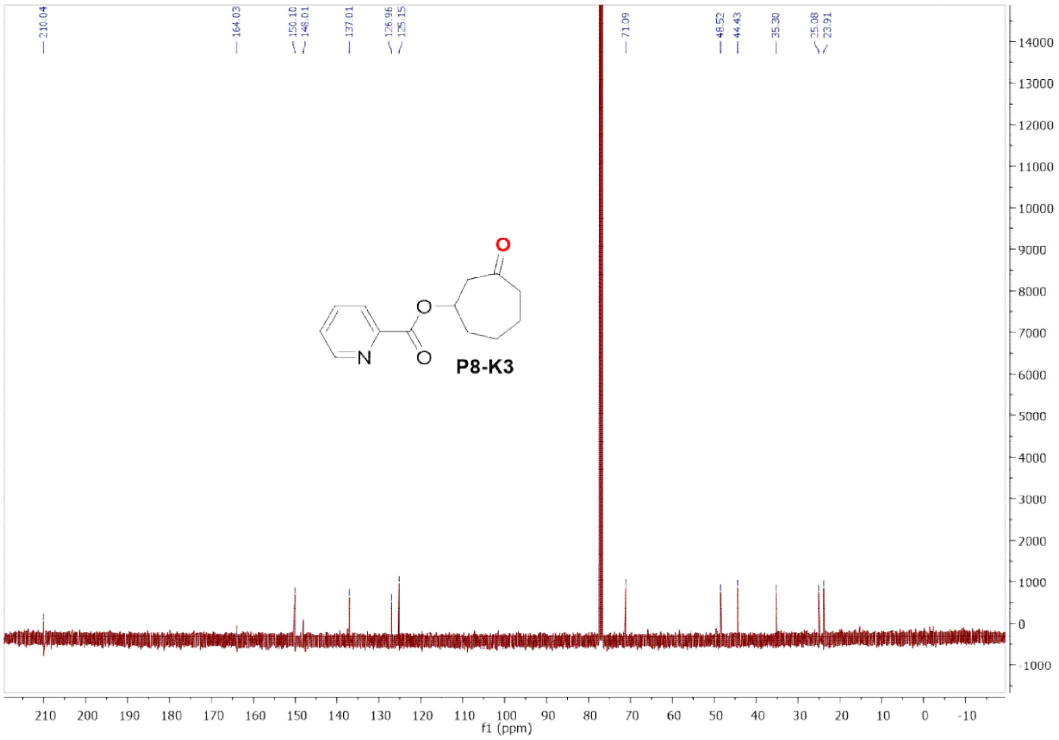

Figure S40. $^{13}C\{^{1}H\}$-NMR spectrum (400 MHz, $CDCl_3$) of 3-oxocycloheptyl 2-pyridinecarboxylate (**P8-K3**).

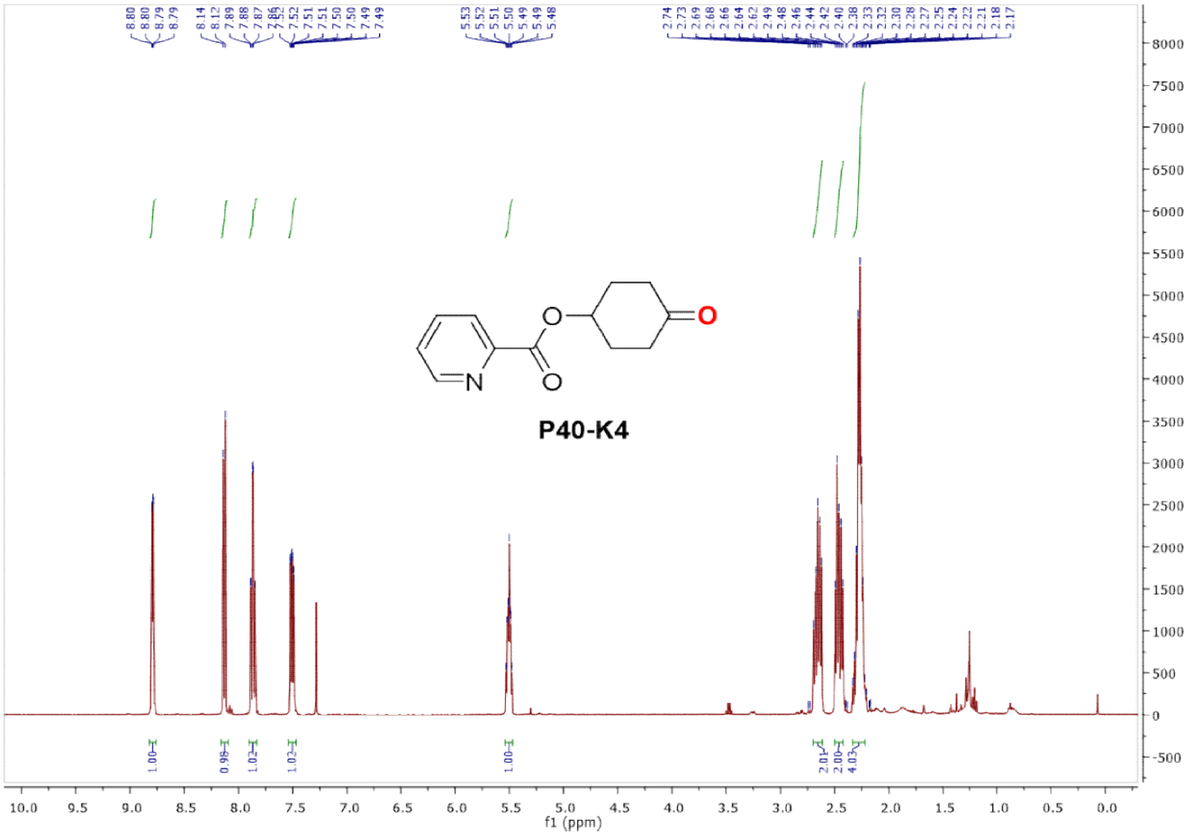

Figure S41. ^{1}H-NMR spectrum (400 MHz, $CDCl_3$) of 4-oxocyclohexyl 2-pyridinecarboxylate (**P40-K4**).

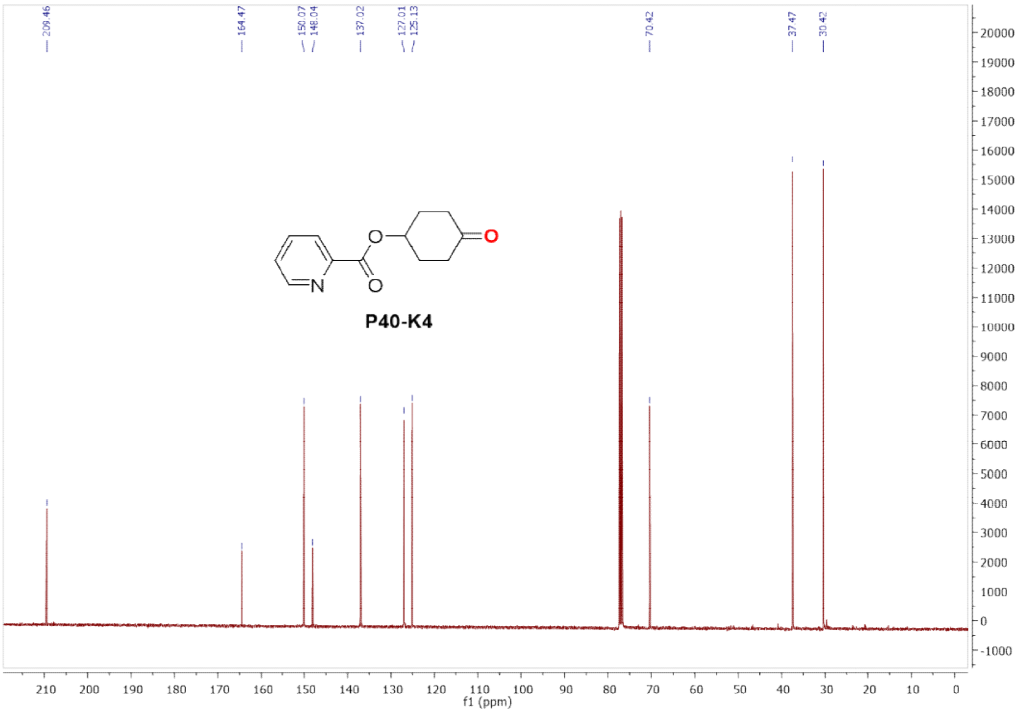

Figure S42. $^{13}C\{^{1}H\}$-NMR spectrum (400 MHz, $CDCl_3$) of 4-oxocyclohexyl 2-pyridinecarboxylate (**P40-K4**).

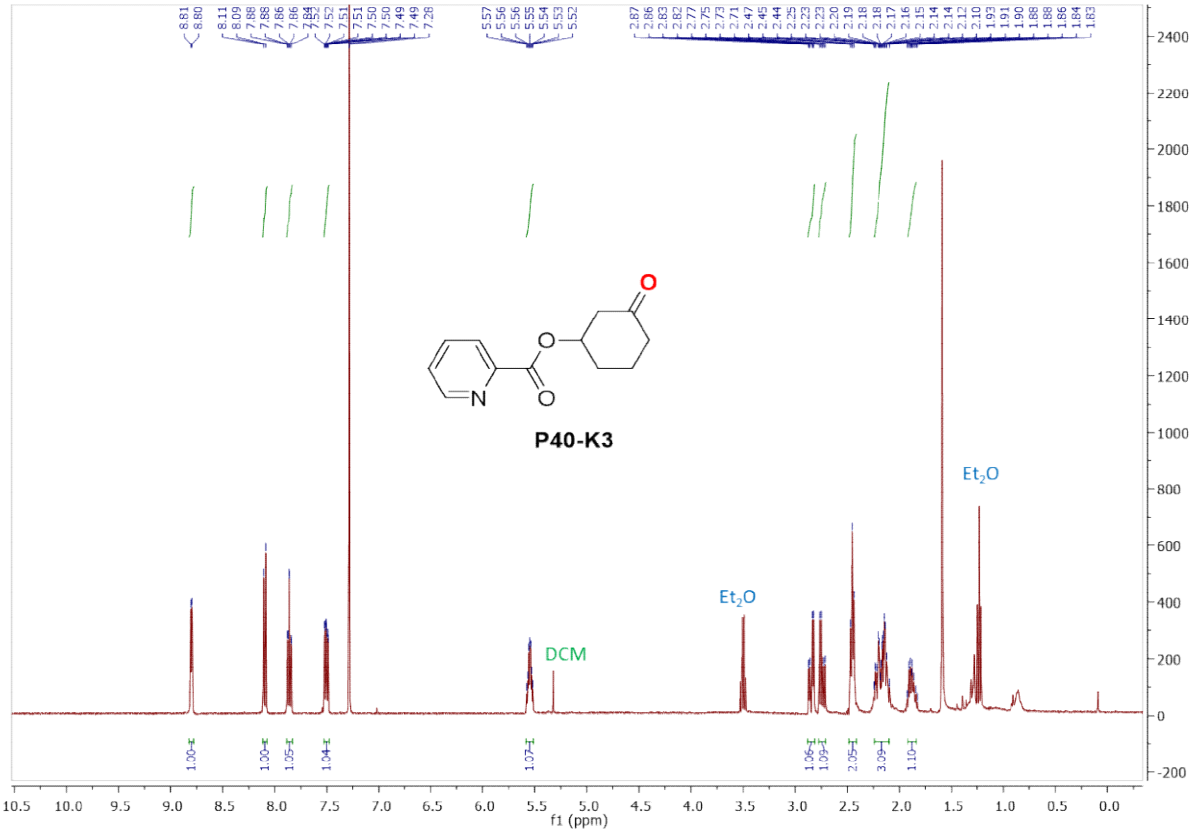

Figure S43. ^{1}H-NMR spectrum (400 MHz, $CDCl_3$) of 3-oxocyclohexyl 2-pyridinecarboxylate (**P40-K3**).

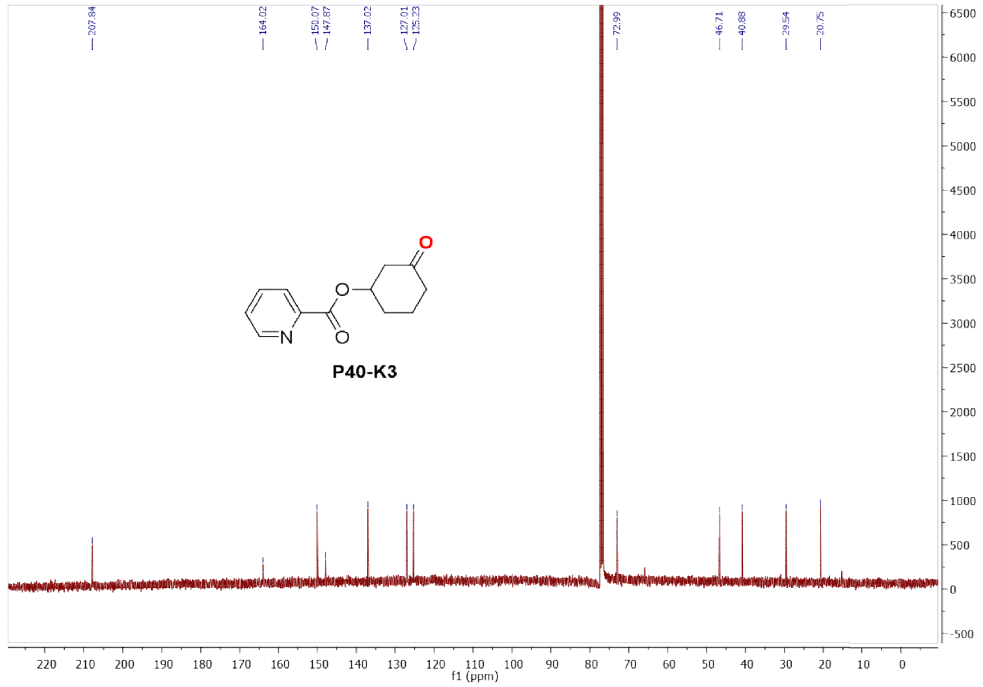

Figure S44. $^{13}C\{^{1}H\}$-NMR spectrum (400 MHz, $CDCl_3$) of 3-oxocyclohexyl 2-pyridinecarboxylate (**P40-K3**).

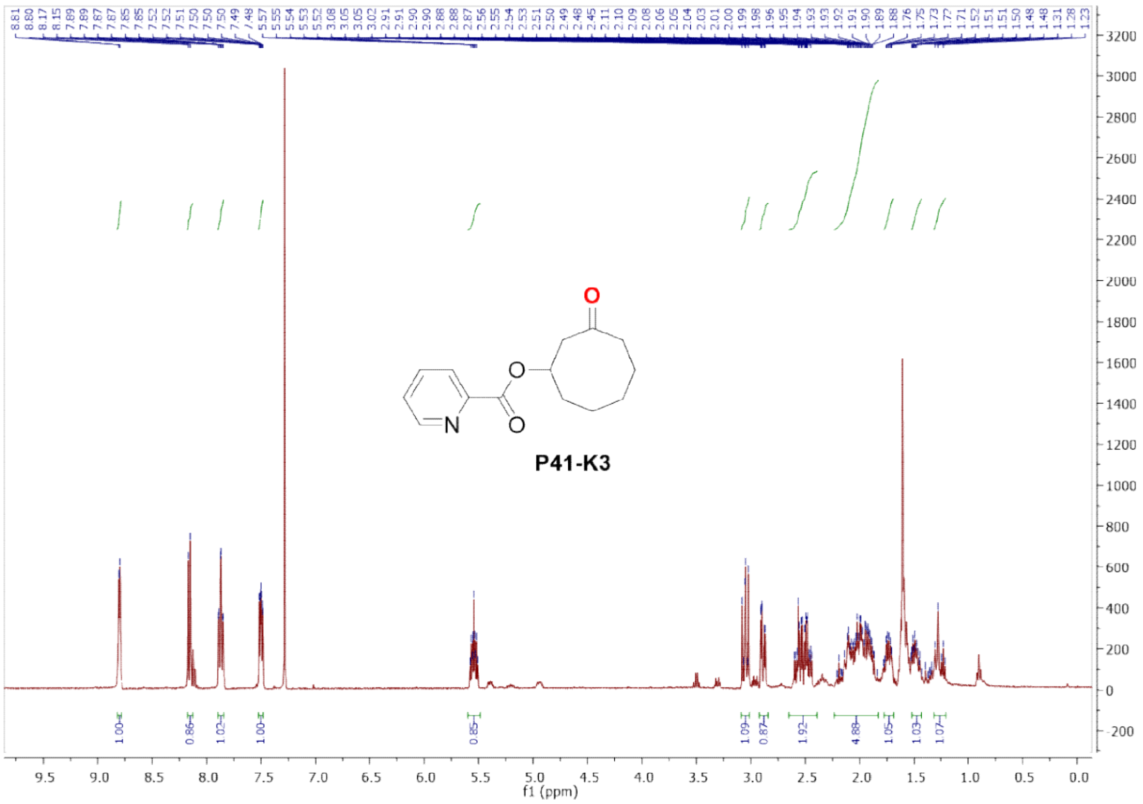

Figure S45. ^{1}H-NMR spectrum (400 MHz, $CDCl_3$) of 3-oxocyclooctyl 2-pyridinecarboxylate (**P41-K3**).

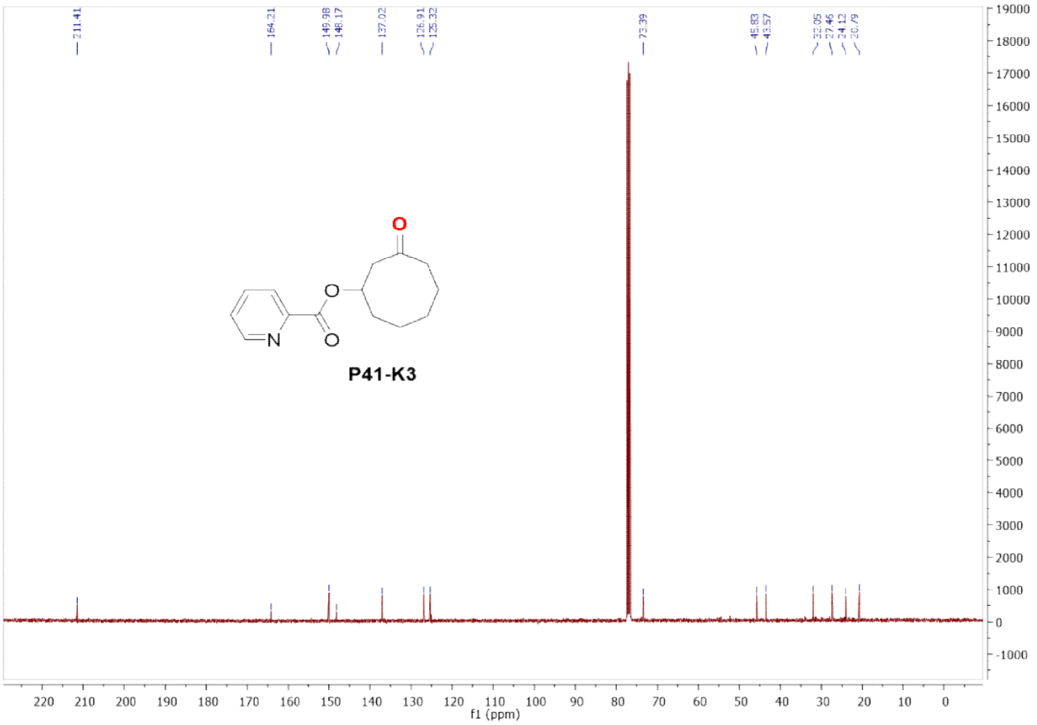

Figure S46. $^{13}C\{^{1}H\}$-NMR spectrum (400 MHz, $CDCl_3$) of 3-oxocyclooctyl 2-pyridinecarboxylate (**P41-K3**).

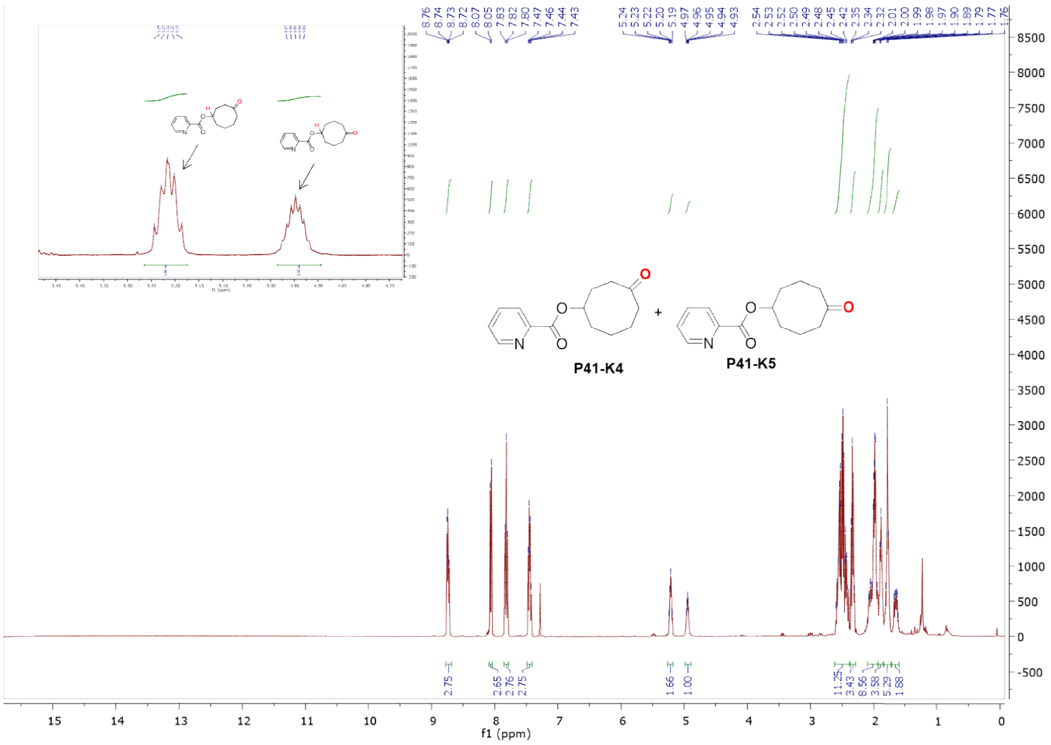

Figure S47. ^{1}H-NMR spectrum (400 MHz, $CDCl_3$) of 4-oxocyclooctyl 2-pyridinecarboxylate+5-oxocyclooctyl 2-pyridinecarboxylate (**P41-K4+P41-K5**).

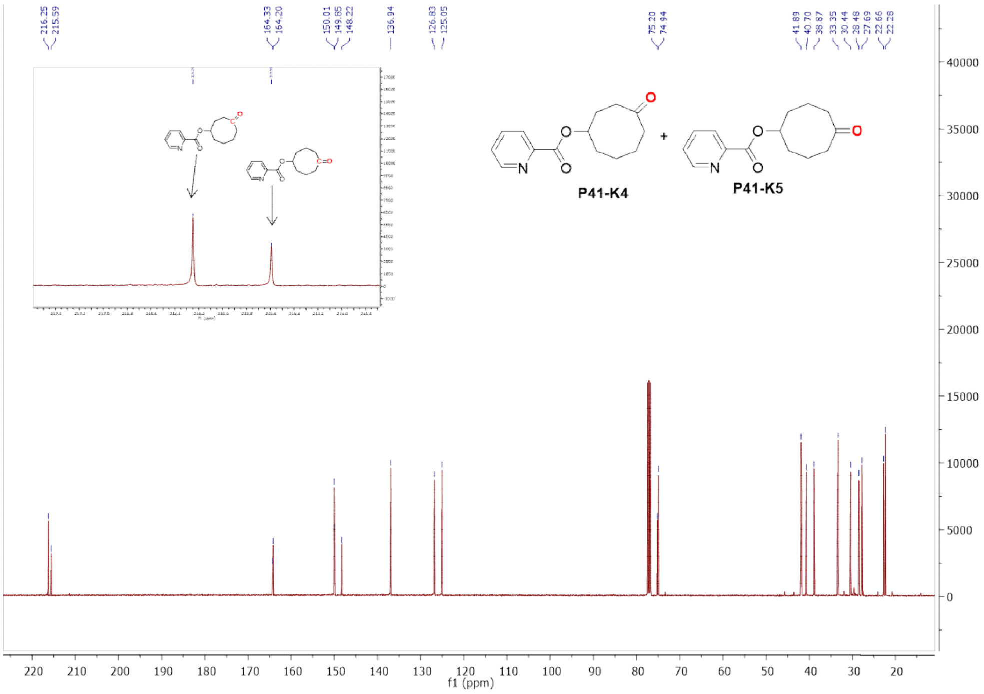

Figure S48. $^{13}C\{^{1}H\}$-NMR spectrum (400 MHz, $CDCl_3$) of 4-oxocyclooctyl 2-pyridinecarboxylate+5-oxocyclooctyl 2-pyridinecarboxylate (**P41-K4+P41-K5**).

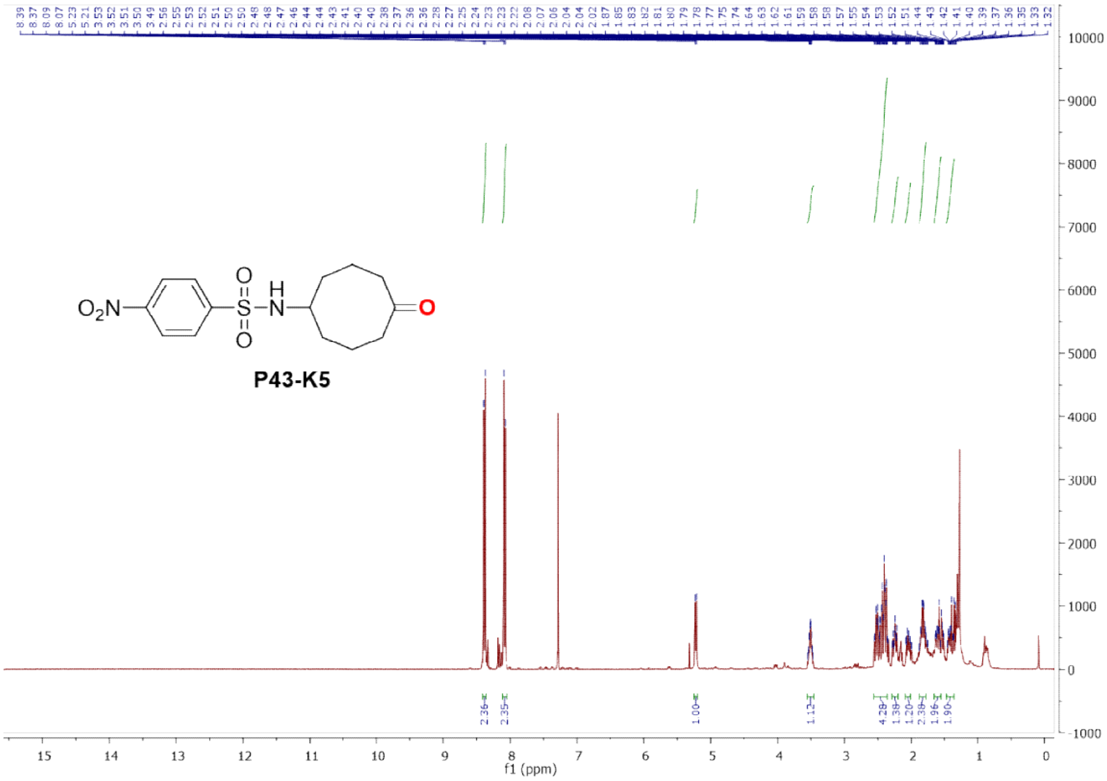

Figure S49. ^{1}H-NMR spectrum (400 MHz, $CDCl_3$) of 4-nitro-N-(5-oxocyclooctyl)benzenesulfonamide (**P43-K5**).

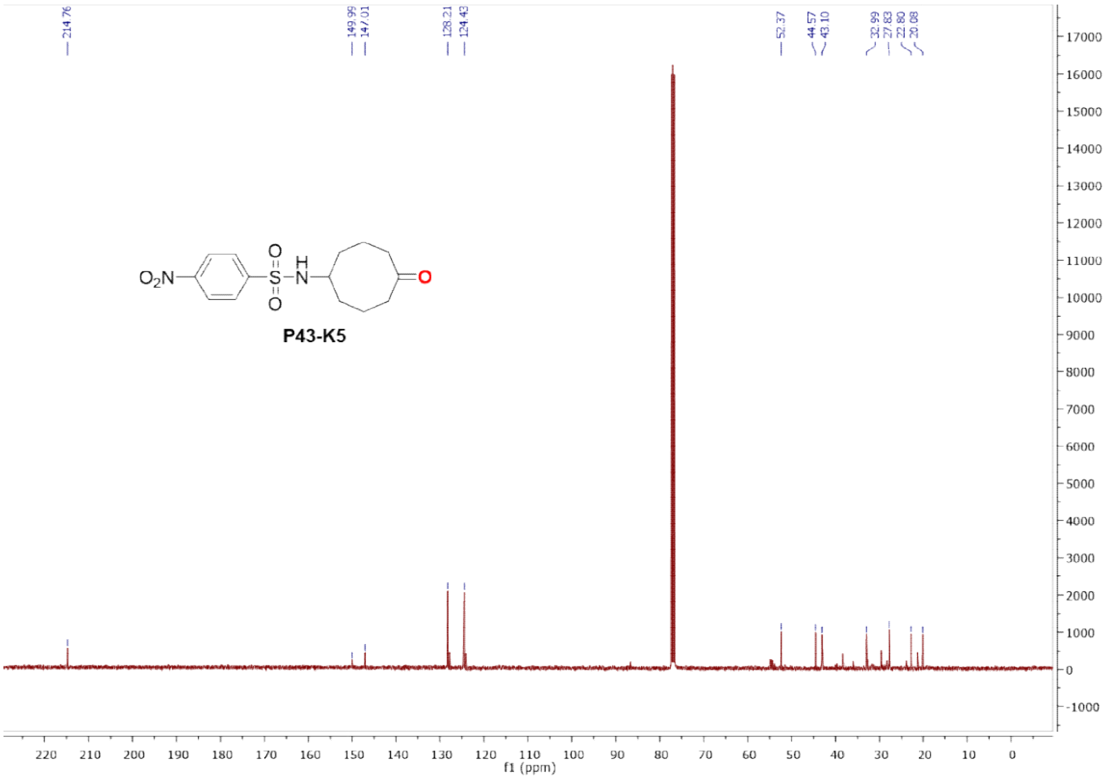

Figure S50. $^{13}C\{^{1}H\}$-NMR spectrum (400 MHz, $CDCl_3$) of 4-nitro-N-(5-oxocyclooctyl)benzenesulfonamide (**P43-K5**).

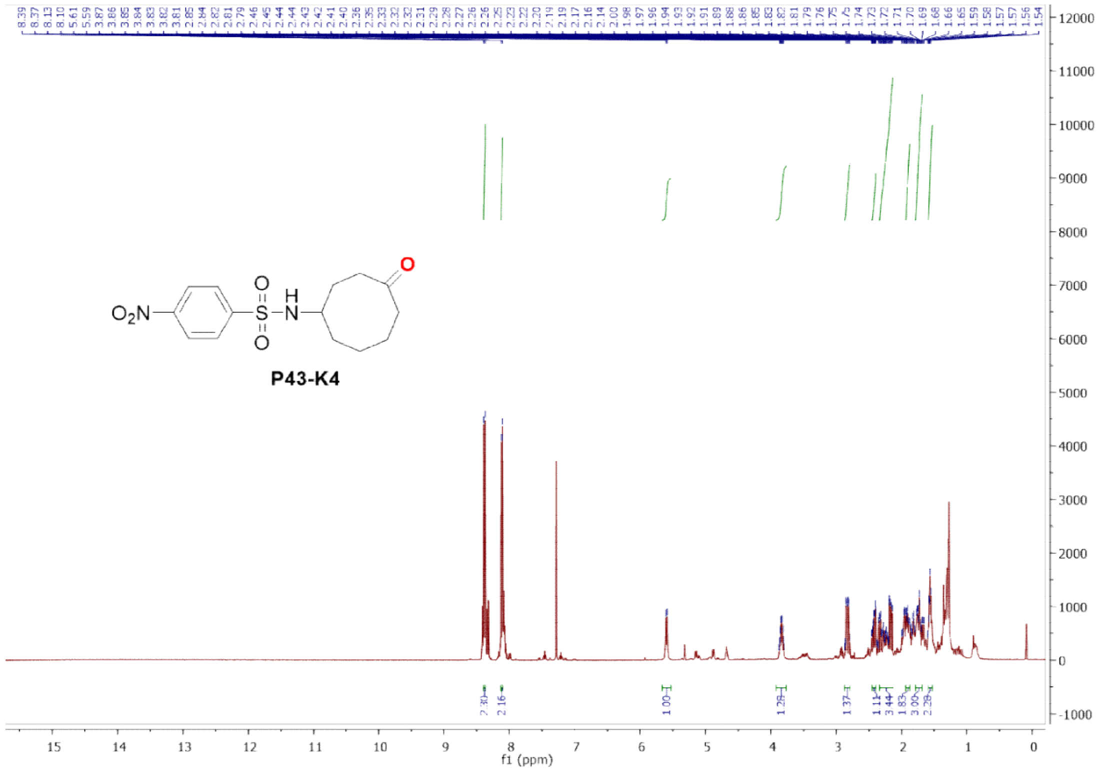

Figure S51. ^{1}H-NMR spectrum (400 MHz, $CDCl_3$) of 4-nitro-N-(4-oxocyclooctyl)benzenesulfonamide (**P43-K4**).

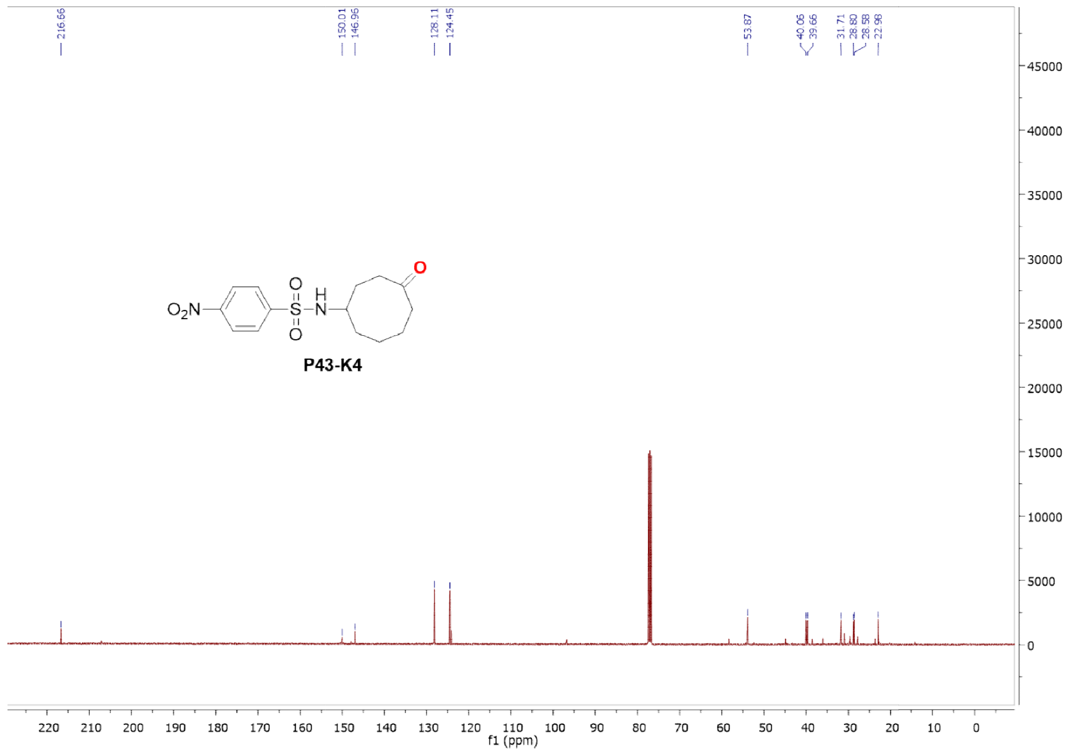

Figure S52. $^{13}C\{^{1}H\}$-NMR spectrum (400 MHz, $CDCl_3$) of 4-nitro-N-(4-oxocyclooctyl)benzenesulfonamide (**P43-K4**).

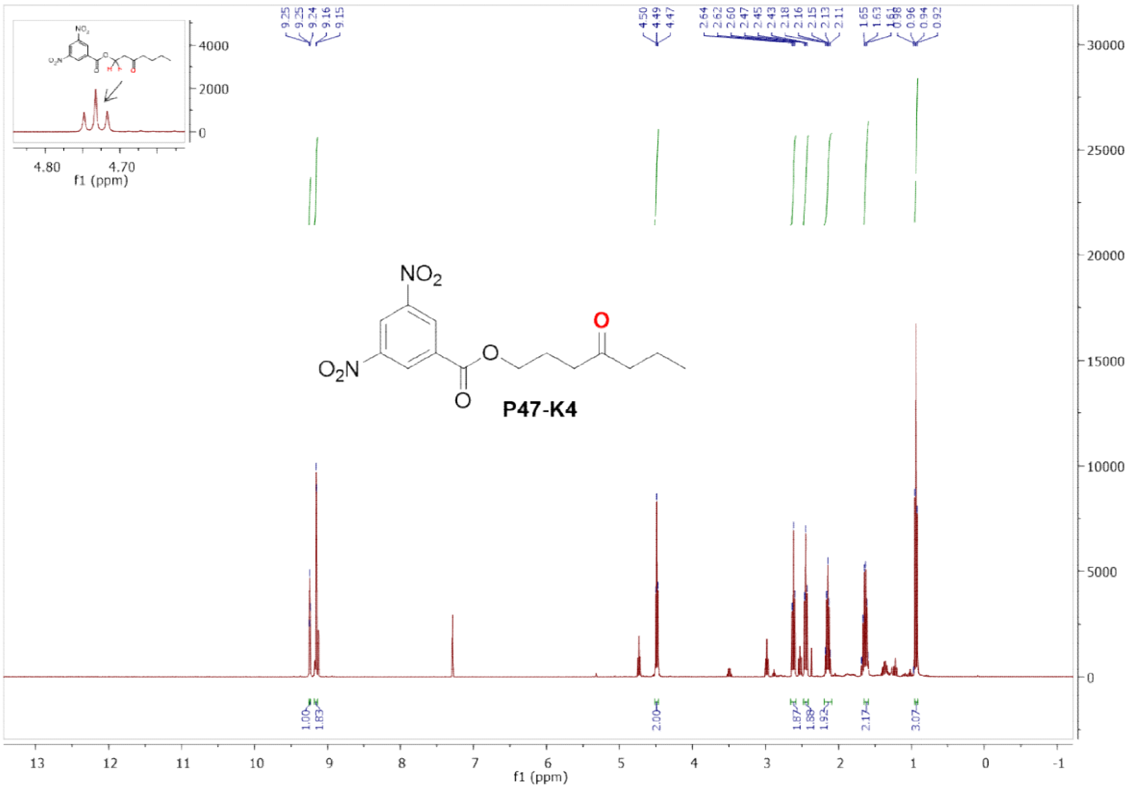

Figure S53. ^{1}H-NMR spectrum (400 MHz, $CDCl_3$) of 4-oxoheptyl 3,5-dinitrobenzoate (**P47-K4**).

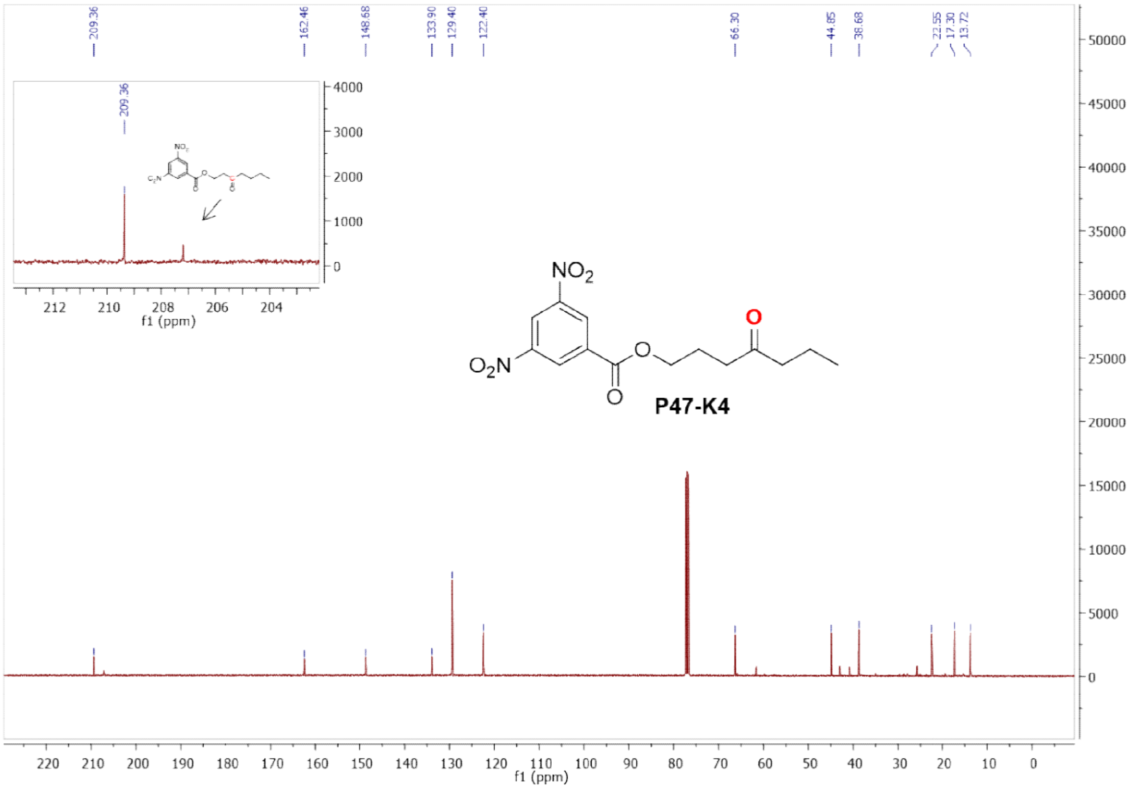

Figure S54. $^{13}C\{^{1}H\}$-NMR spectrum (400 MHz, $CDCl_3$) of 4-oxoheptyl 3,5-dinitrobenzoate (**P47-K4**).

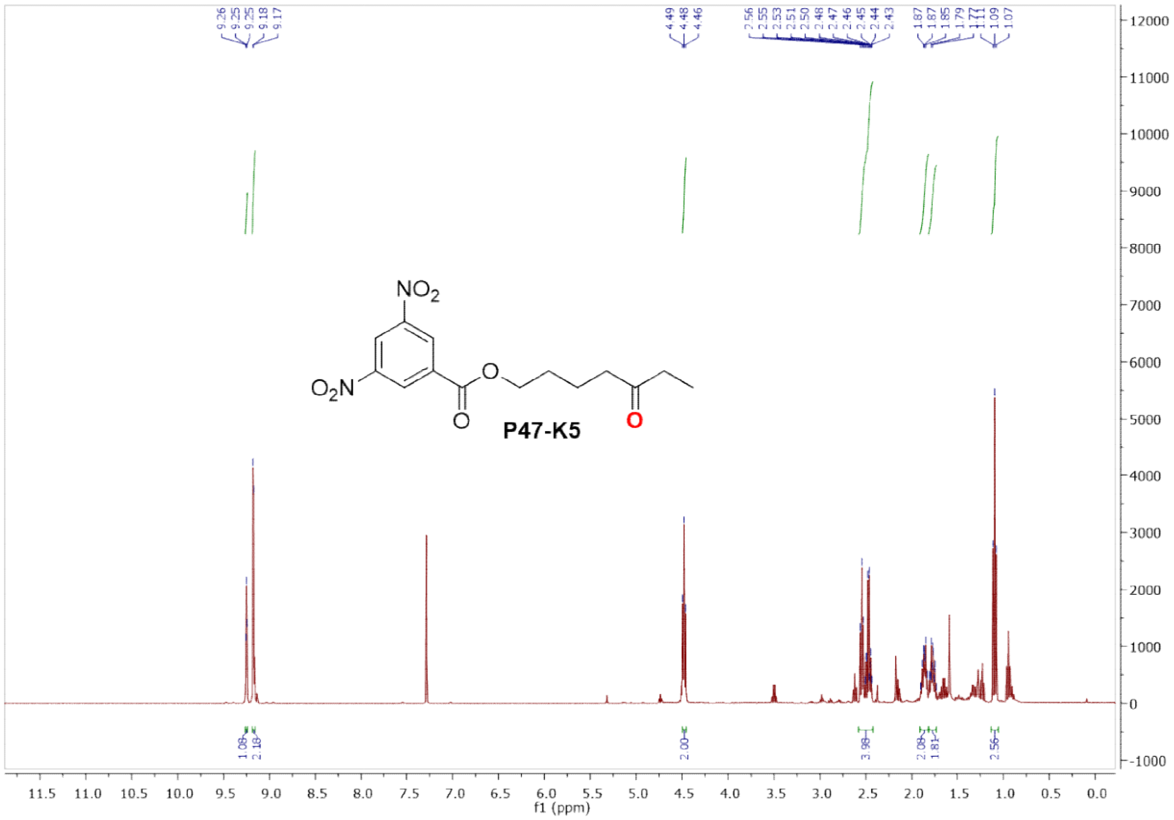

Figure S55. ^{1}H-NMR spectrum (400 MHz, $CDCl_3$) of 5-oxoheptyl 3,5-dinitrobenzoate (**P47-K5**).

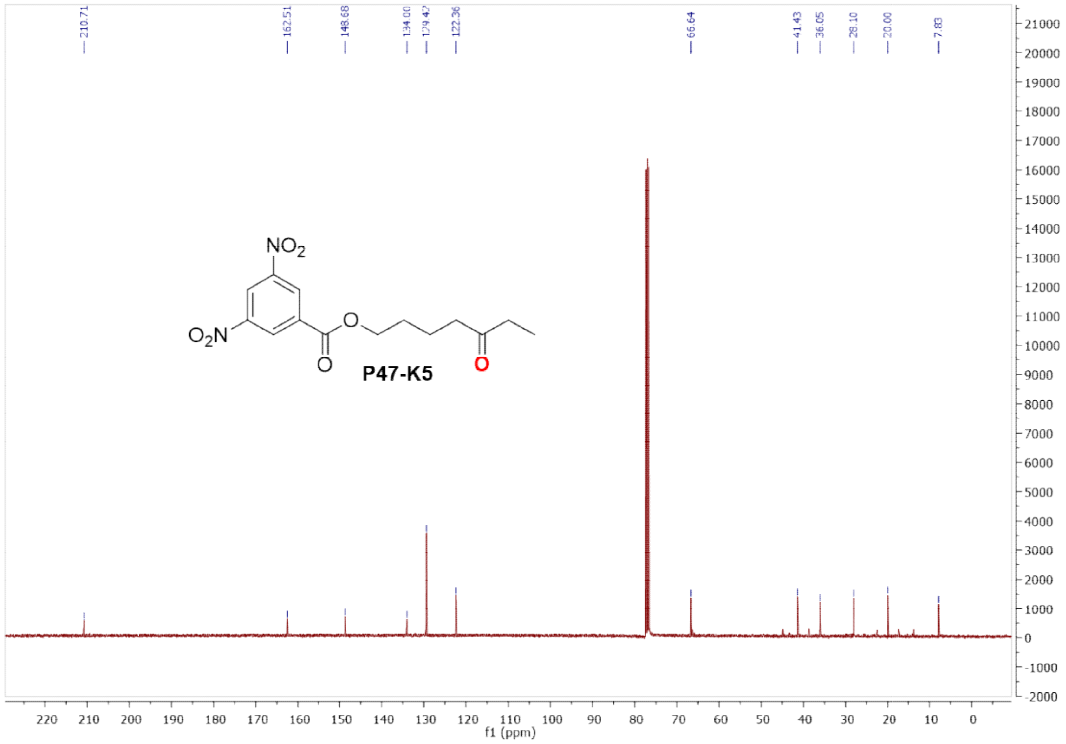

Figure S56. $^{13}C\{^{1}H\}$-NMR spectrum (400 MHz, $CDCl_3$) of 5-oxoheptyl 3,5-dinitrobenzoate (**P47-K5**).

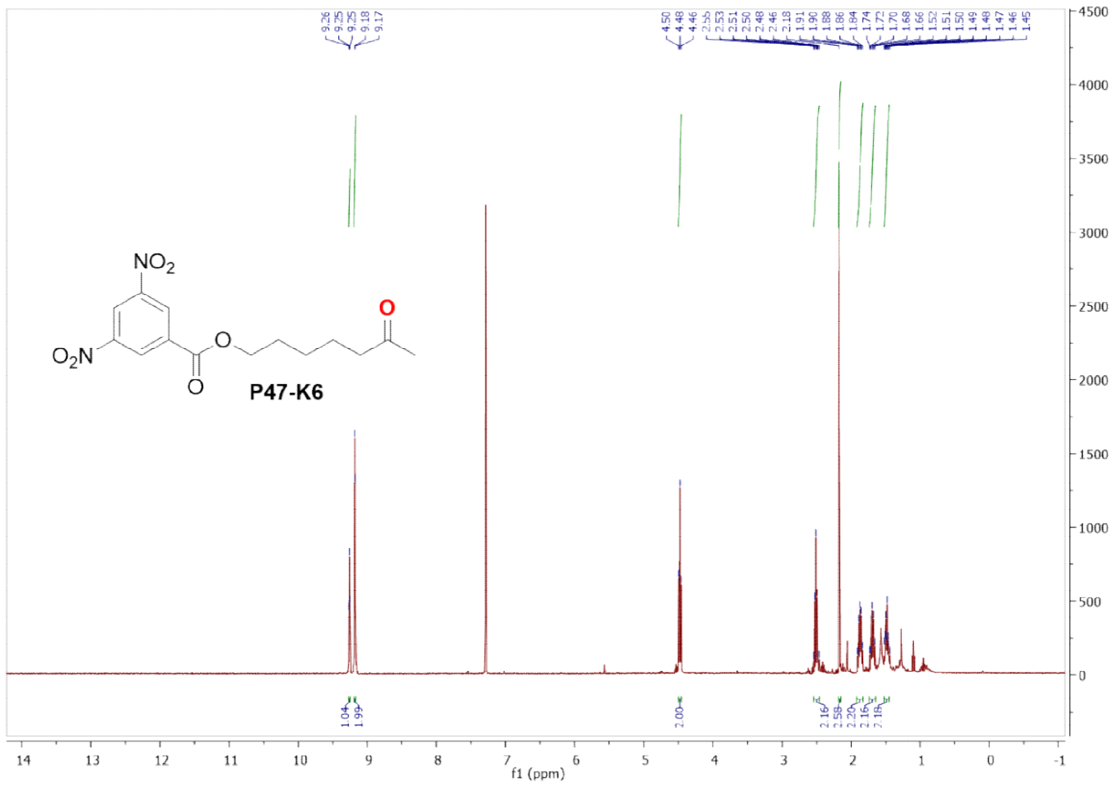

Figure S57. ^{1}H-NMR spectrum (400 MHz, $CDCl_3$) of 6-oxoheptyl 3,5-dinitrobenzoate (**P47-K6**).

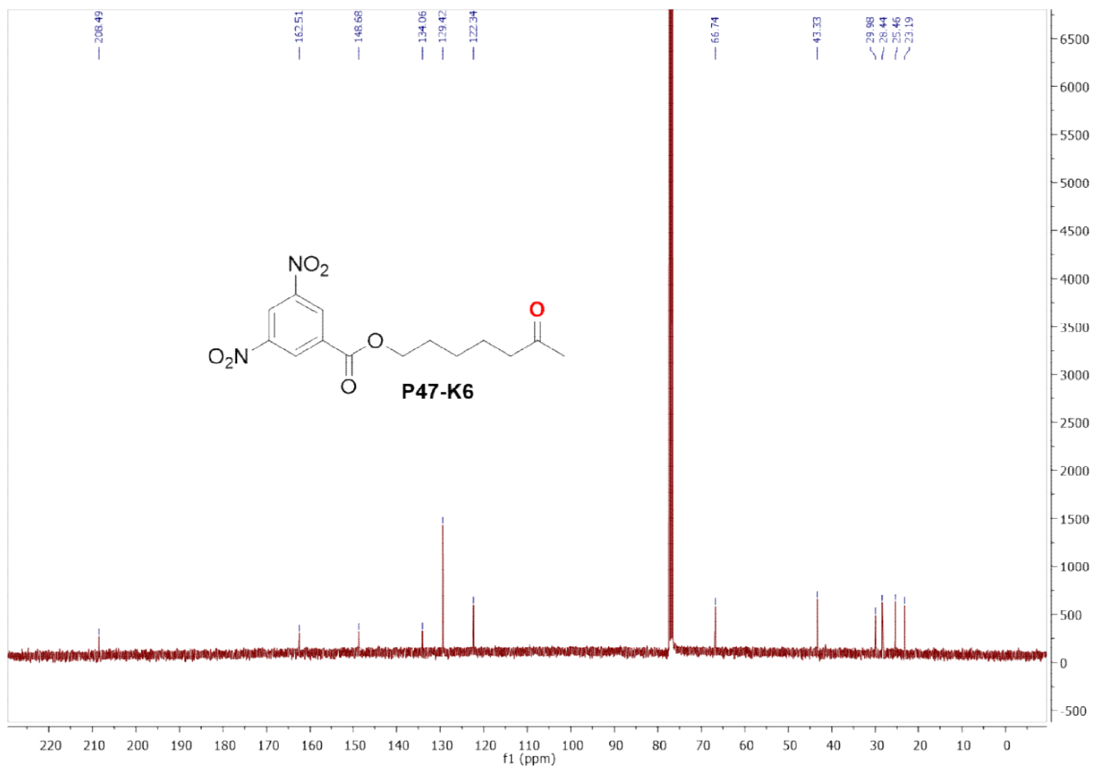

Figure S58. $^{13}C\{^{1}H\}$-NMR spectrum (400 MHz, $CDCl_3$) of 6-oxoheptyl 3,5-dinitrobenzoate (**P47-K6**).

6.3. NMR spectra and GC chromatograms of product mixtures

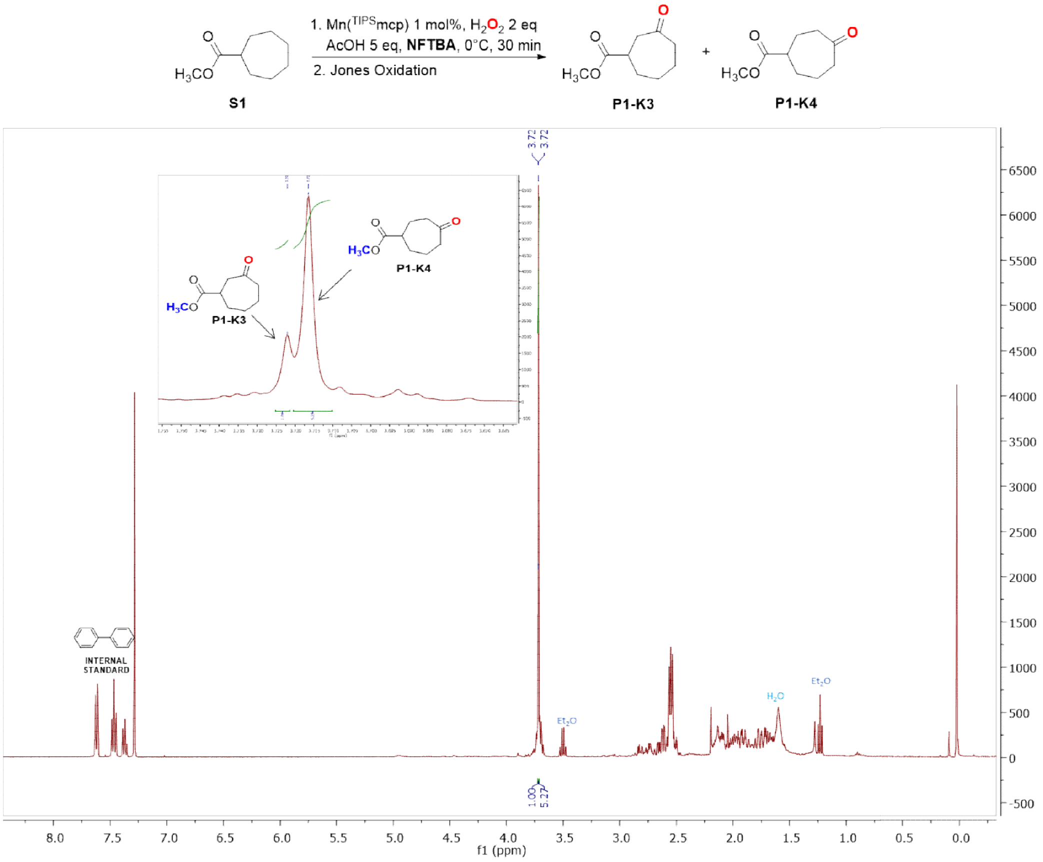

Figure S59. Crude mixture ^{1}H-NMR spectrum (400 MHz, $CDCl_3$) of methylcycloheptane carboxylate (**S1**) oxidation in NFTBA.

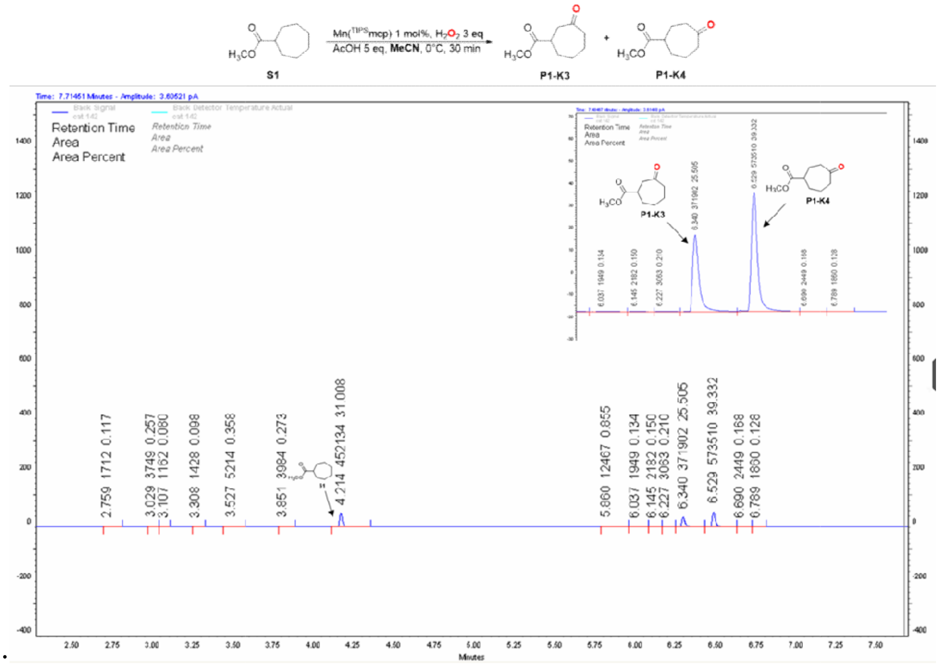

Figure S60. Crude mixture chromatogram of methylcycloheptane carboxylate (**S1**) oxidation in MeCN.

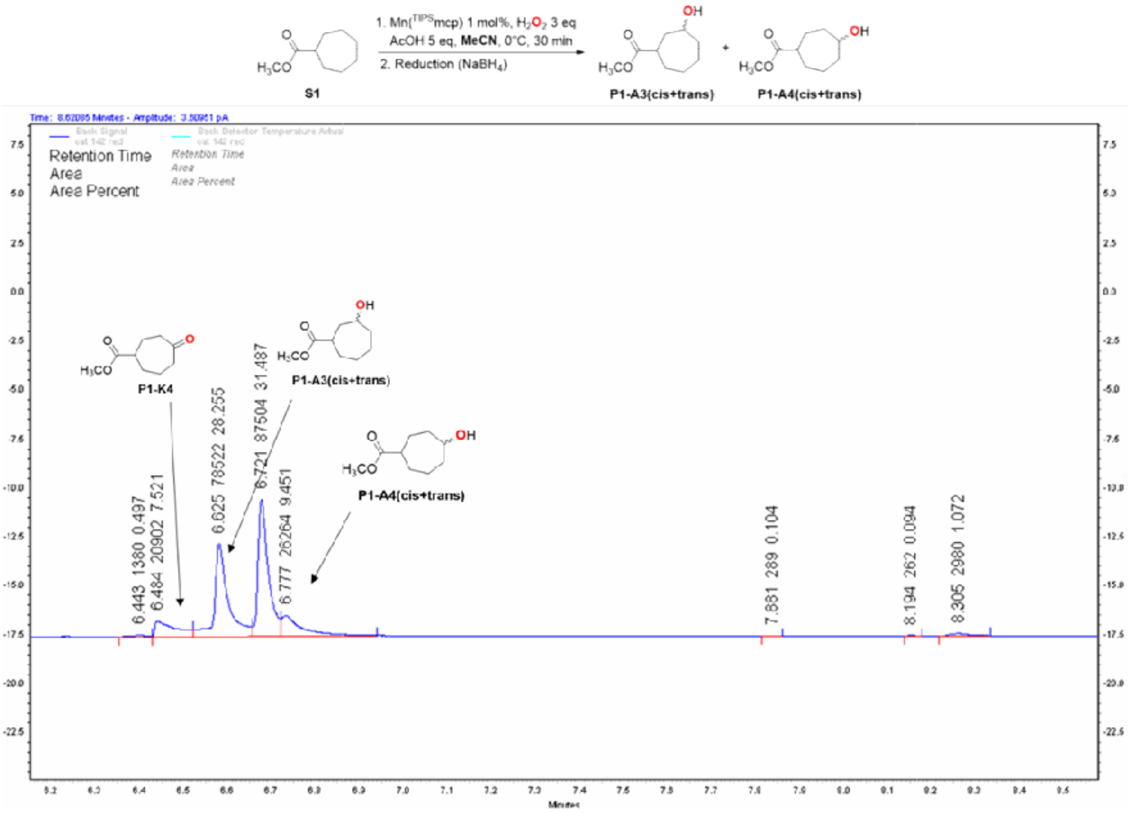

Figure S61. $NaBH_4$ reduced crude mixture chromatogram of methylcycloheptane carboxylate (**S1**) oxidation in MeCN.

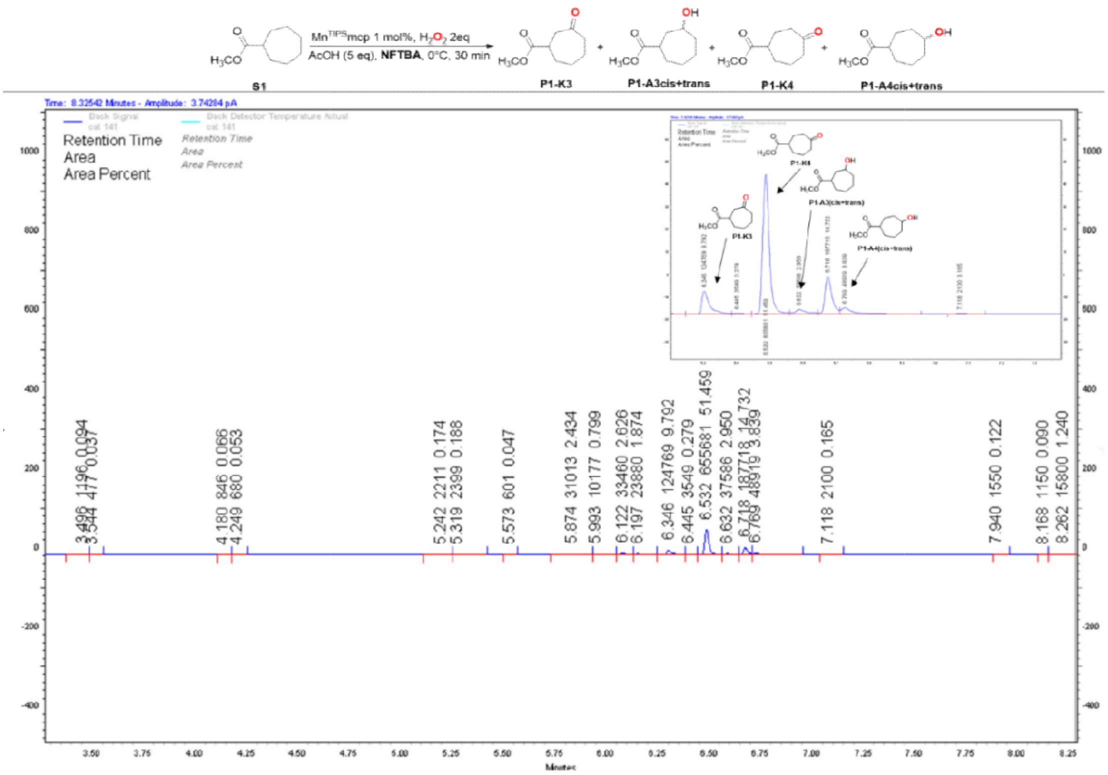

Figure S62. Crude mixture chromatogram of methylcycloheptane carboxylate (**S1**) oxidation in NFTBA.

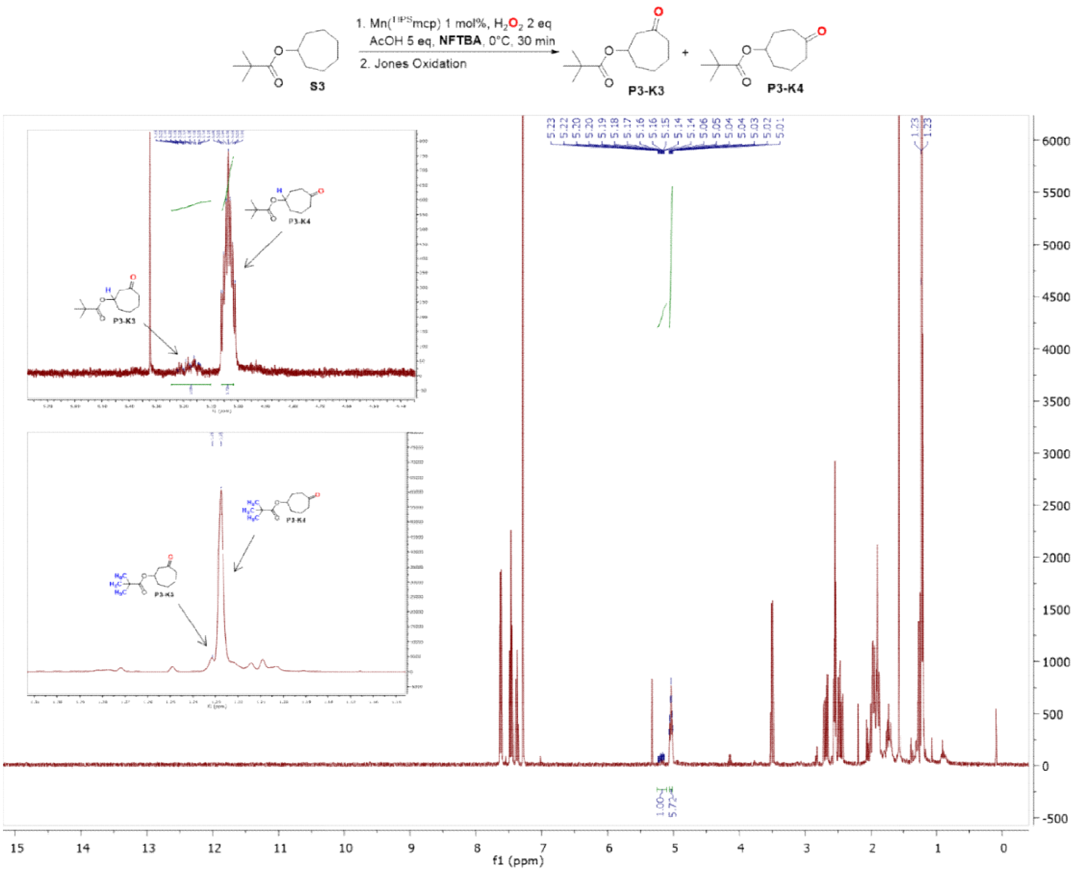

Figure S63. Crude mixture ^{1}H-NMR spectrum (400 MHz, $CDCl_3$) of cycloheptyl pivalate (**S3**) oxidation in NFTBA.

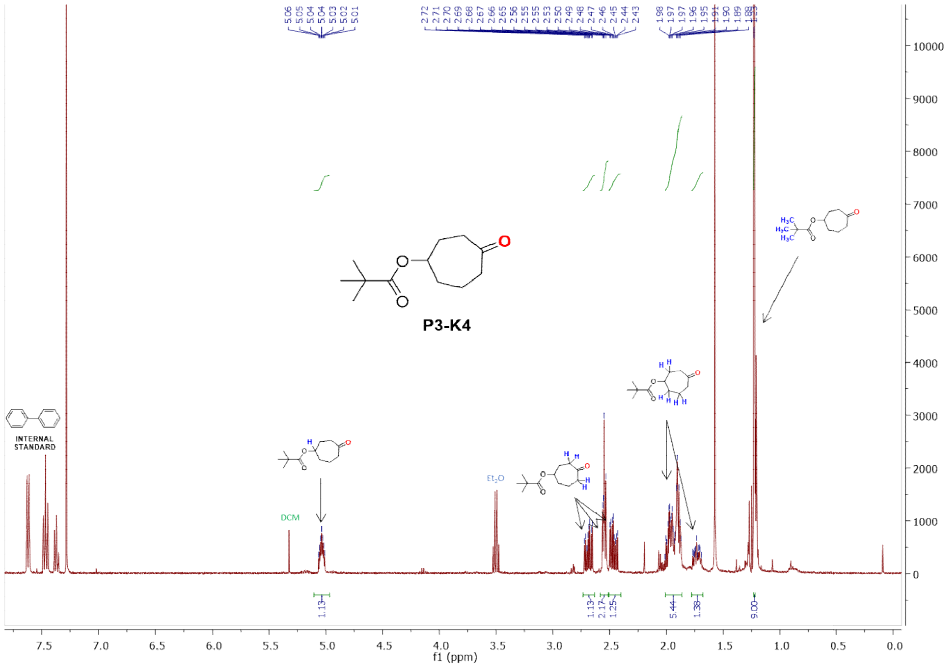

Figure S64. Crude mixture ^{1}H-NMR spectrum (400 MHz, $CDCl_3$) of cycloheptyl pivalate (**S3**) oxidation in NFTBA: Identification of **P3-K4**.

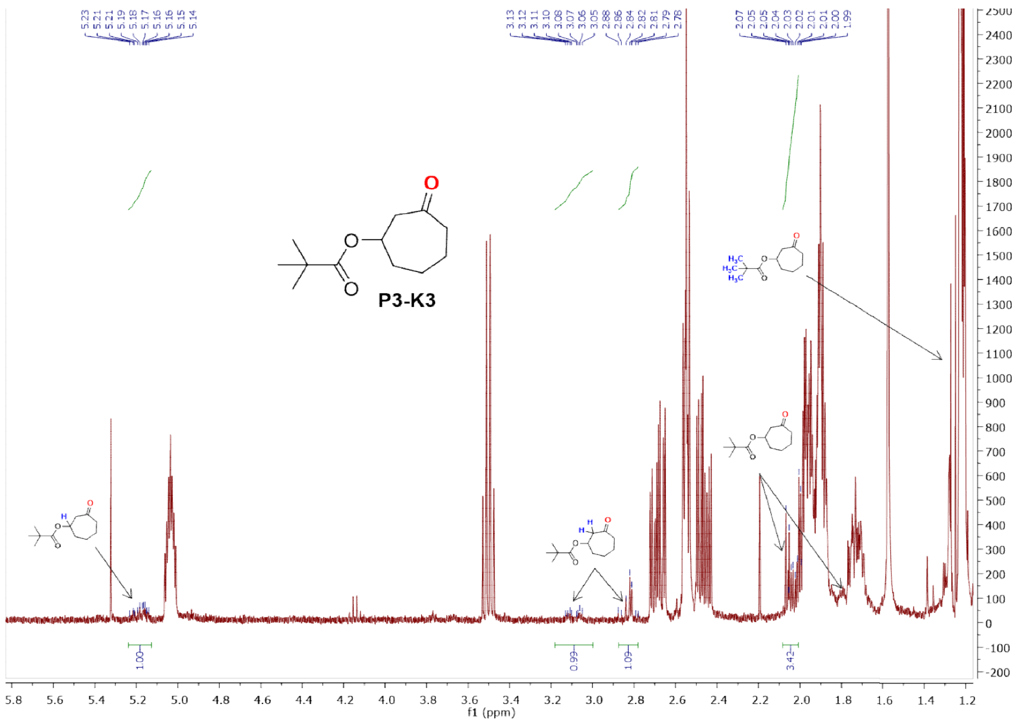

Figure S65. Crude mixture ^{1}H-NMR spectrum (400 MHz, $CDCl_3$) of cycloheptyl pivalate (**S3**) oxidation in NFTBA: Identification of **P3-K3**.

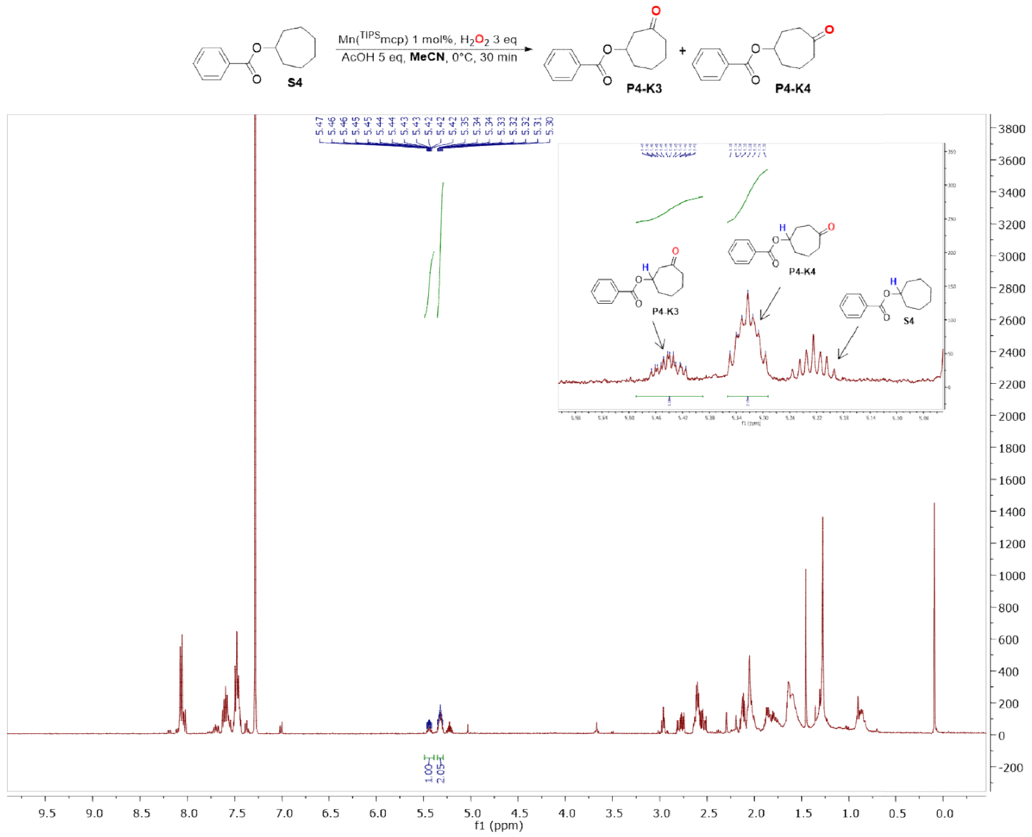

Figure S66. Crude mixture ^{1}H-NMR spectrum (400 MHz, $CDCl_3$) of cycloheptyl benzoate (**S4**) oxidation in MeCN.

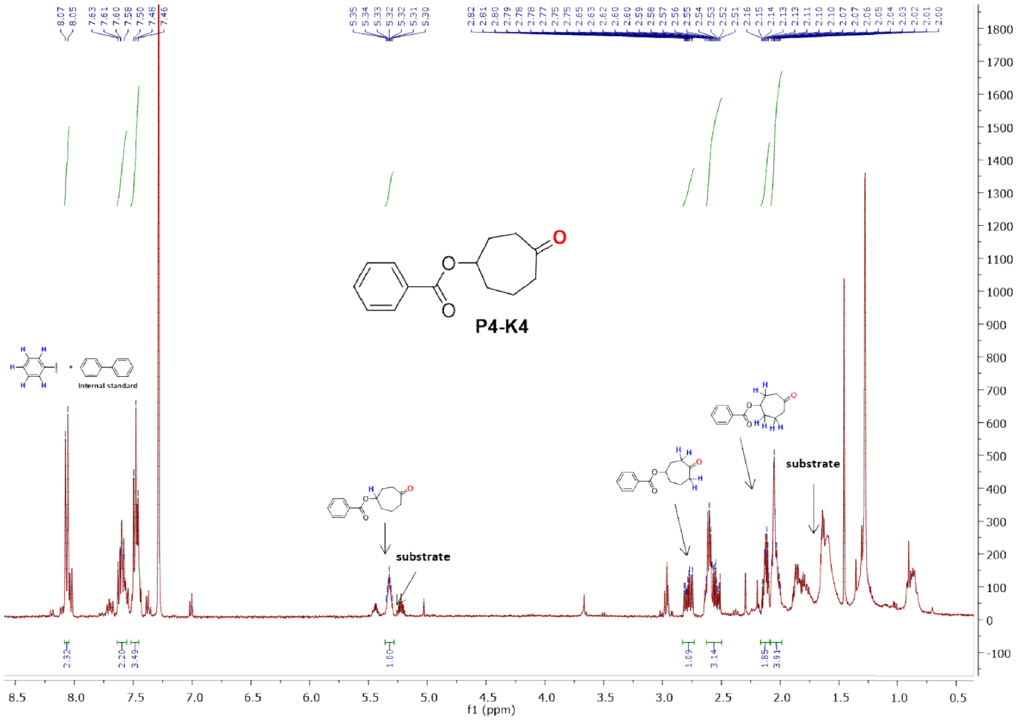

Figure S67. Crude mixture ^{1}H-NMR spectrum (400 MHz, $CDCl_3$) of cycloheptyl benzoate (**S4**) oxidation in MeCN: identification of **P4-K4**.

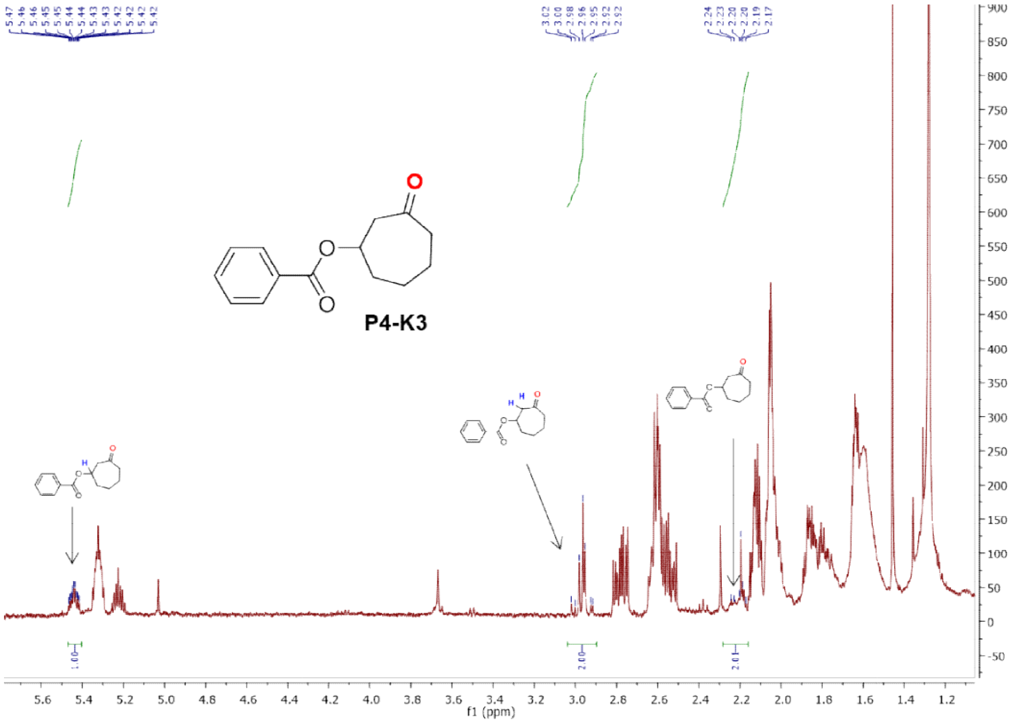

Figure S68. Crude mixture ^{1}H-NMR spectrum (400 MHz, $CDCl_3$) of cycloheptyl benzoate (**S4**) oxidation in MeCN: identification of **P4-K3**.

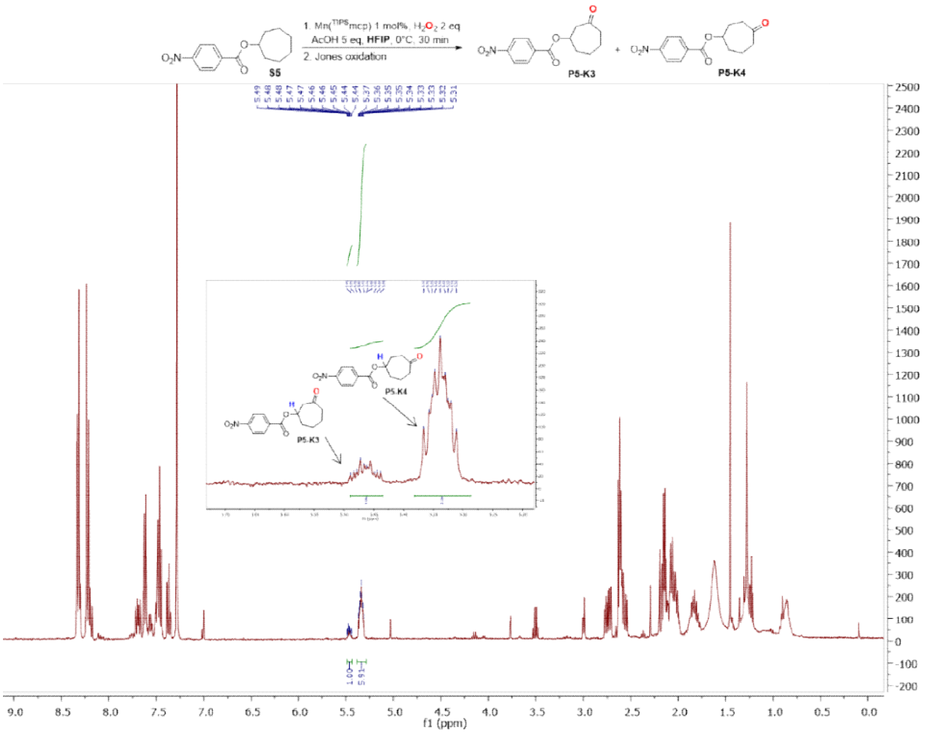

Figure S69. Crude mixture ^{1}H-NMR spectrum (400 MHz, $CDCl_3$) of cycloheptyl 4-nitrobenzoate (**S5**) oxidation in NFTBA.

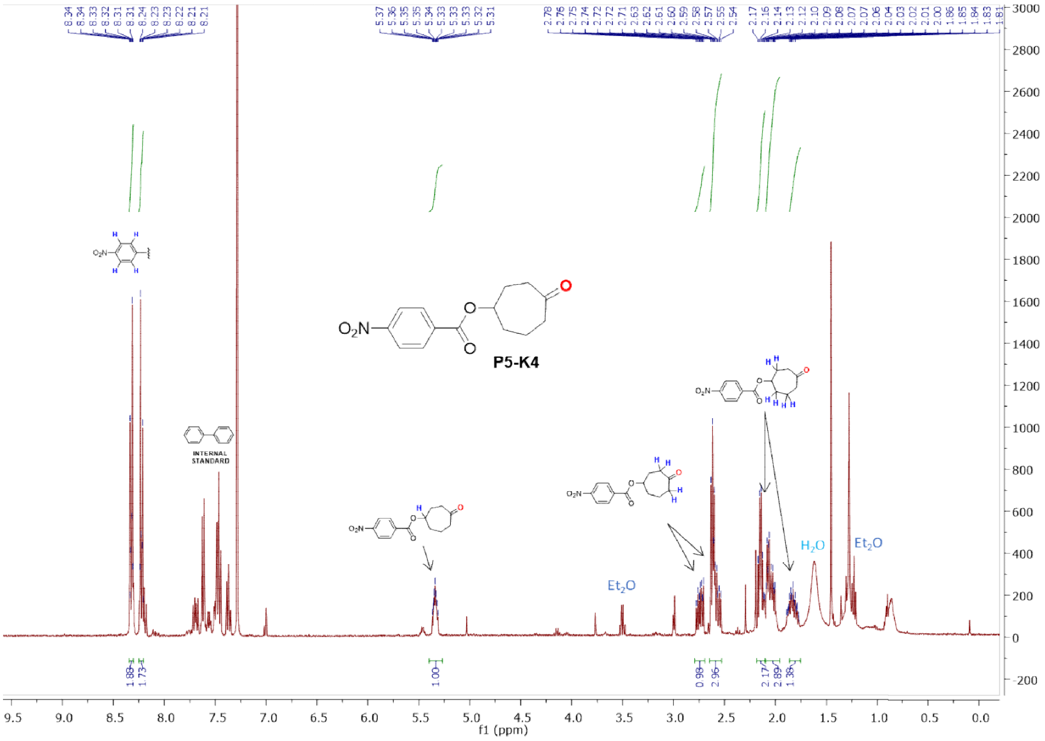

Figure S70. Crude mixture ^{1}H-NMR spectrum (400 MHz, $CDCl_3$) of cycloheptyl 4-nitrobenzoate (**S5**) oxidation in NFTBA: Identification of **P5-K4**.

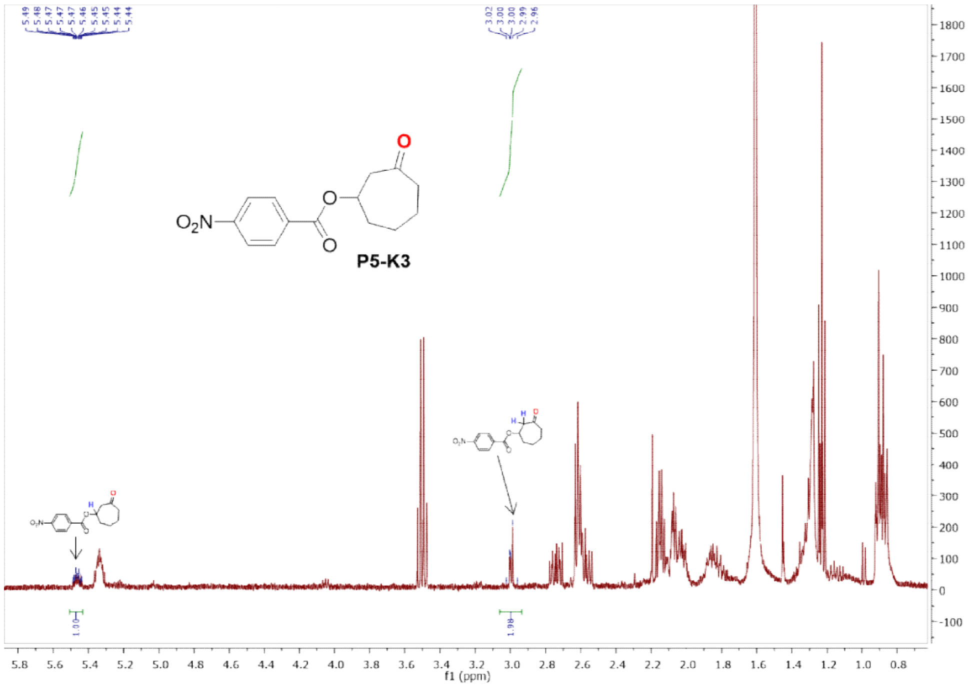

Figure S71. Crude mixture ^{1}H-NMR spectrum (400 MHz, $CDCl_3$) of cycloheptyl 4-nitrobenzoate (**S5**) oxidation in NFTBA:Identification of **P5-K3**.

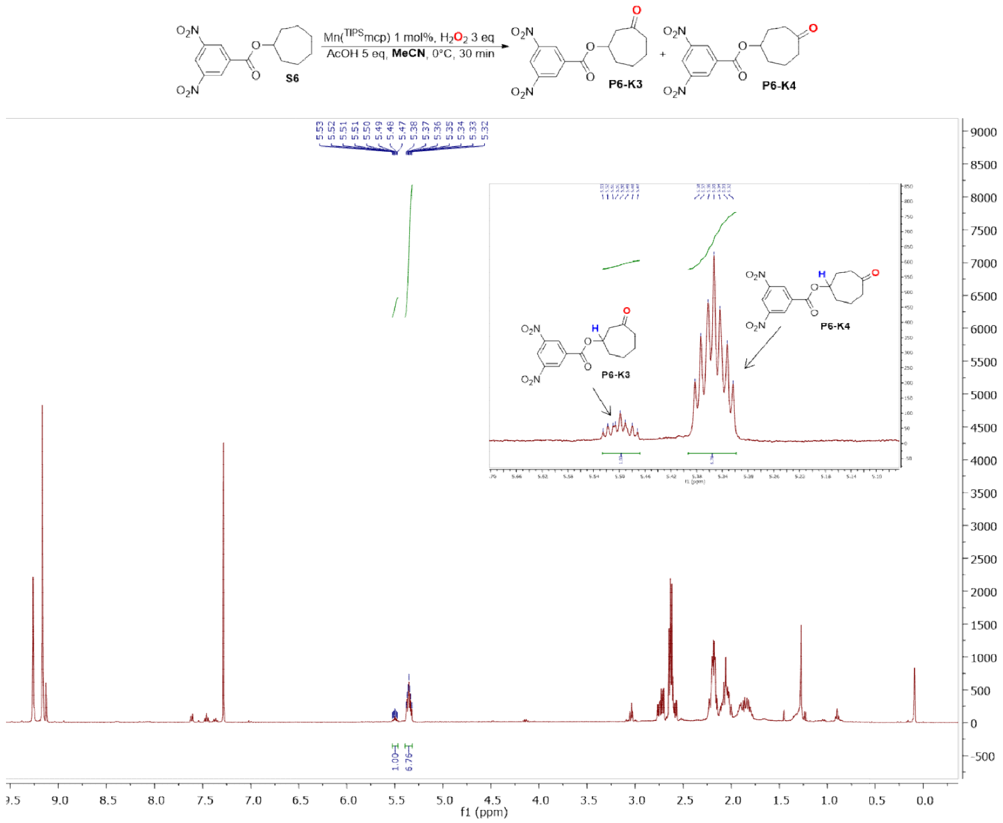

Figure S72. Crude mixture ^{1}H-NMR spectrum (400 MHz, $CDCl_3$) of cycloheptyl 3,5-dinitrobenzoate (**S6**) oxidation in MeCN.

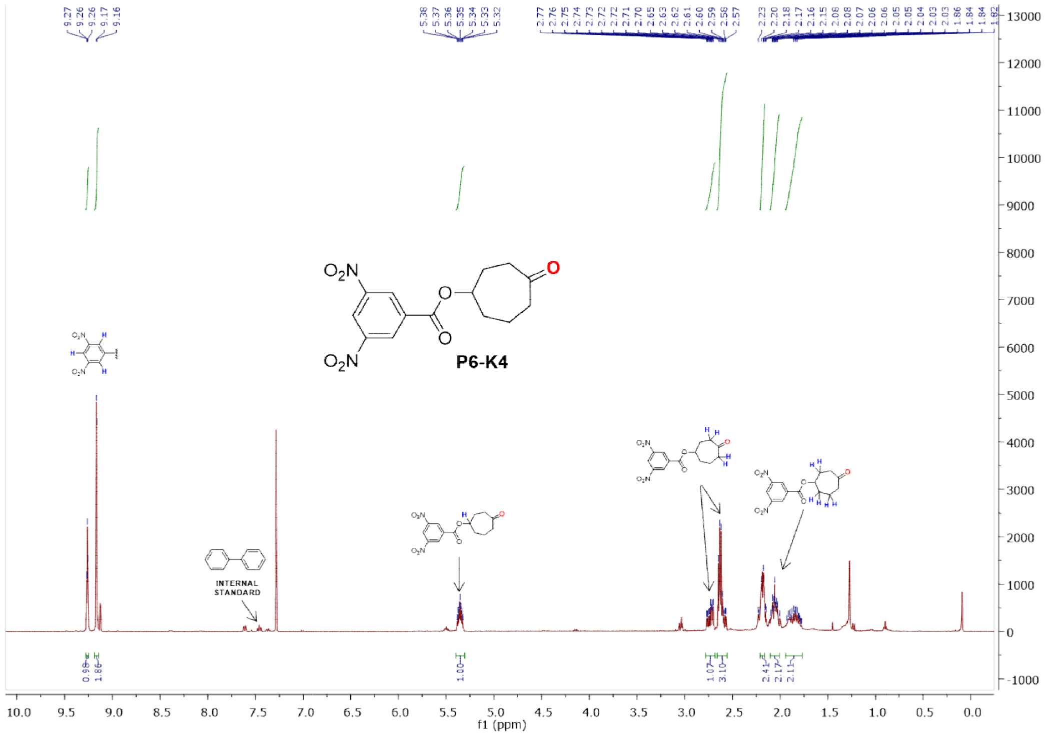

Figure S73. Crude mixture ^{1}H-NMR spectrum (400 MHz, $CDCl_3$) of cycloheptyl 3,5-dinitrobenzoate (**S6**) oxidation in MeCN: Identification of **P6-K4**.

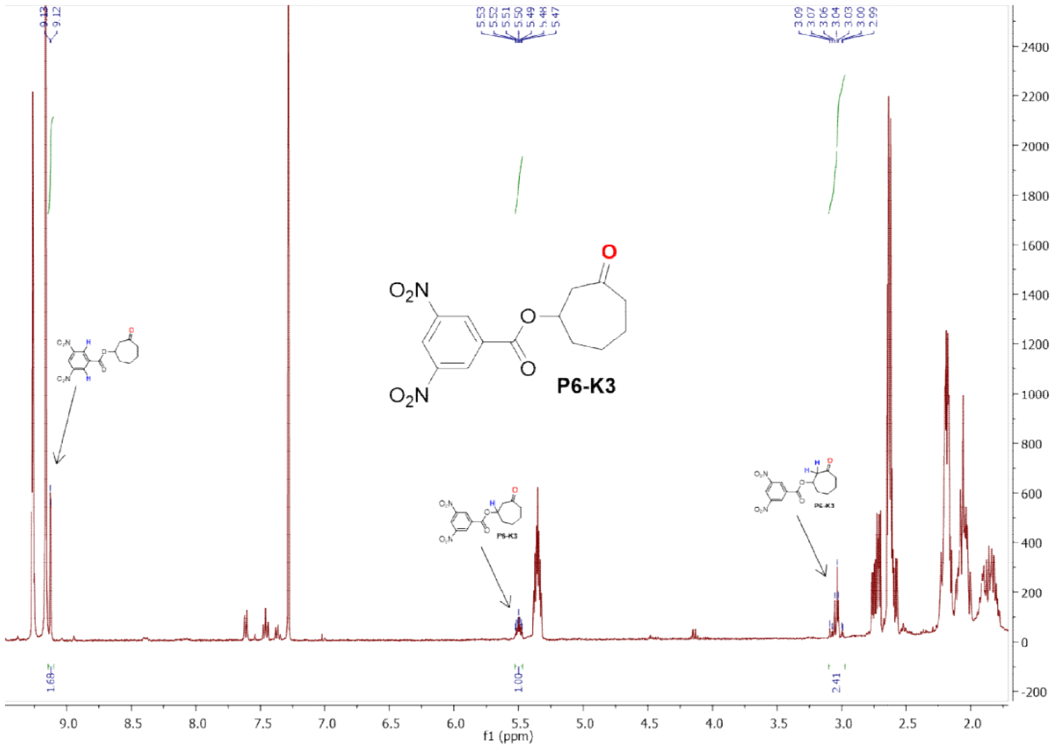

Figure S74. Crude mixture ^{1}H-NMR spectrum (400 MHz, $CDCl_3$) of cycloheptyl 3,5-dinitrobenzoate (**S6**) oxidation in MeCN: Identification of **P6-K3**.

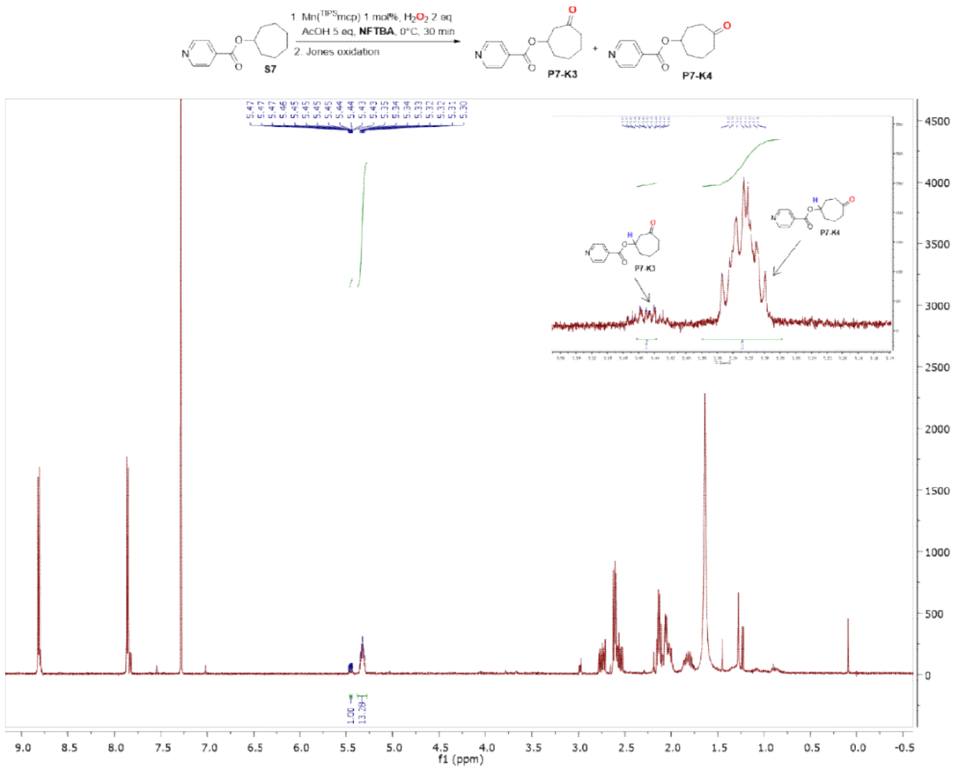

Figure S75. Crude mixture ^{1}H-NMR spectrum (400 MHz, $CDCl_3$) of cycloheptyl 4-pyridinecarboxylate (**S7**) oxidation in NFTBA.

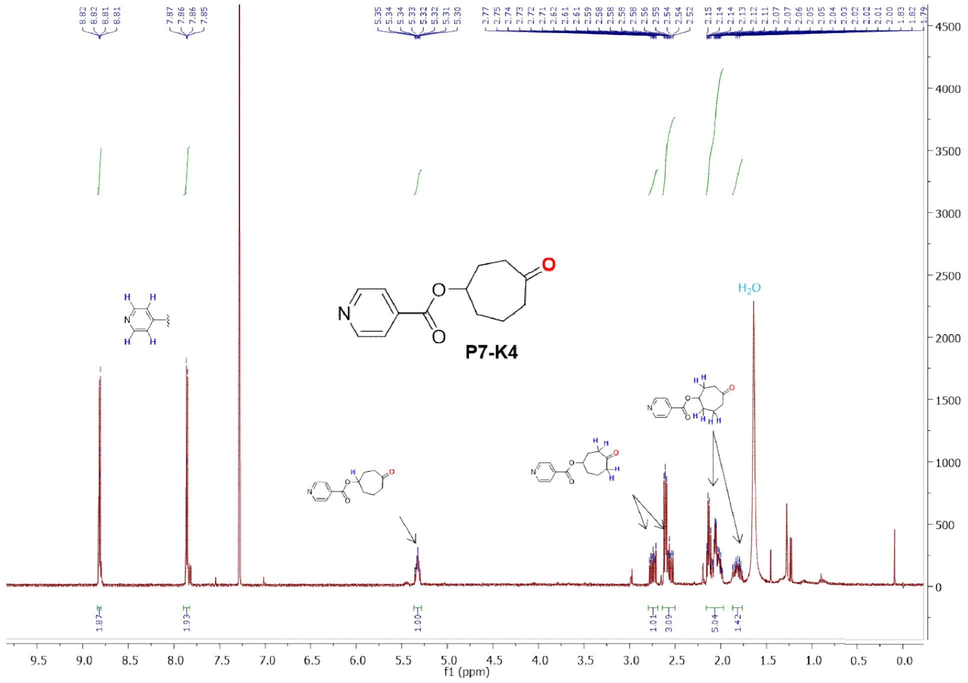

Figure S76. Crude mixture ^{1}H-NMR spectrum (400 MHz, $CDCl_3$) of cycloheptyl 4-pyridinecarboxylate (**S7**) oxidation in NFTBA: Identification of **P7-K4**.

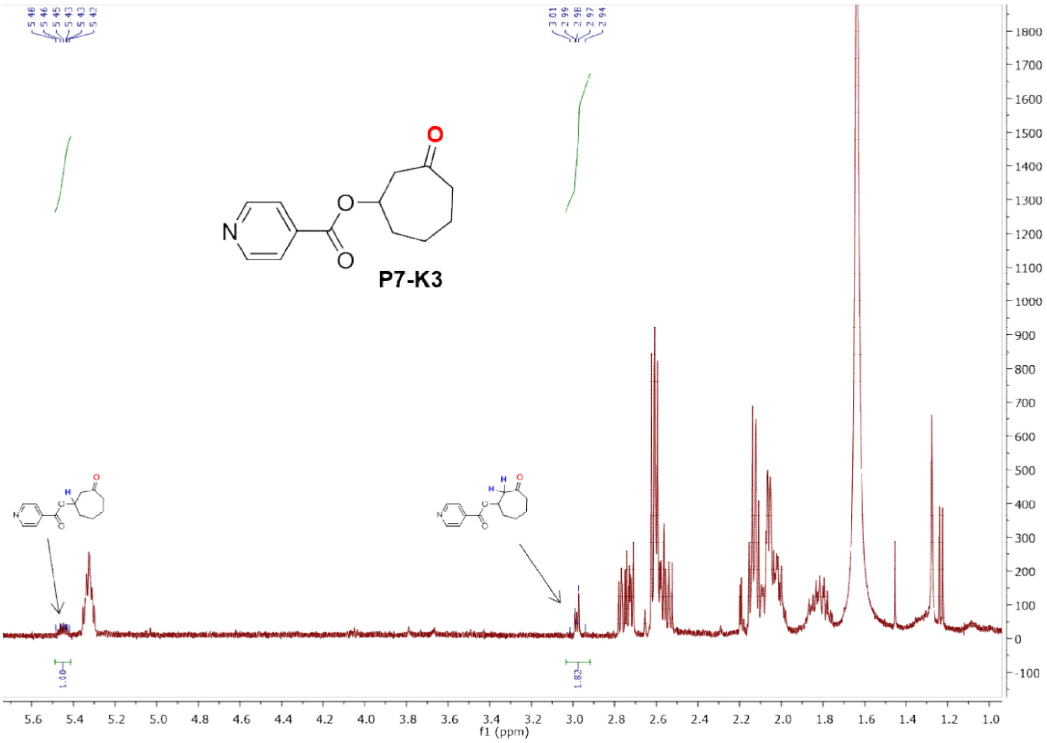

Figure S77. Crude mixture ^{1}H-NMR spectrum (400 MHz, $CDCl_3$) of cycloheptyl 4-pyridinecarboxylate (**S7**) oxidation in NFTBA: Identification of **P7-K3**.

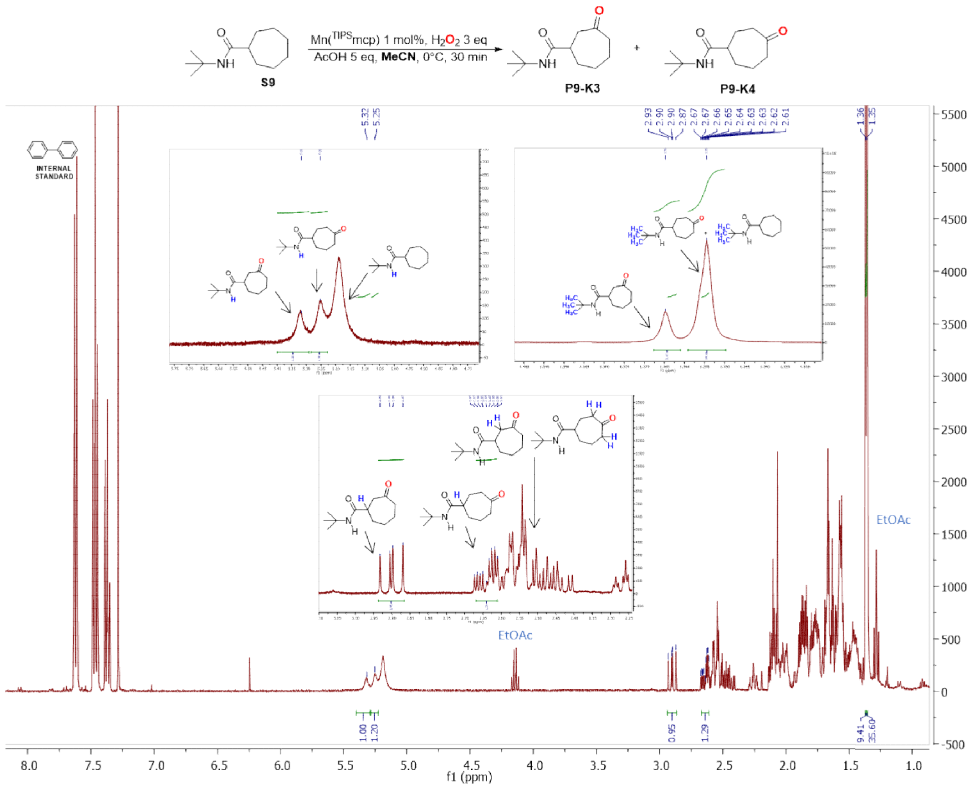

Figure S78. Crude mixture ^{1}H-NMR spectrum (400 MHz, $CDCl_3$) of *N*-(tert-butyl)cycloheptanecarboxamide (**S9**) oxidation in MeCN.

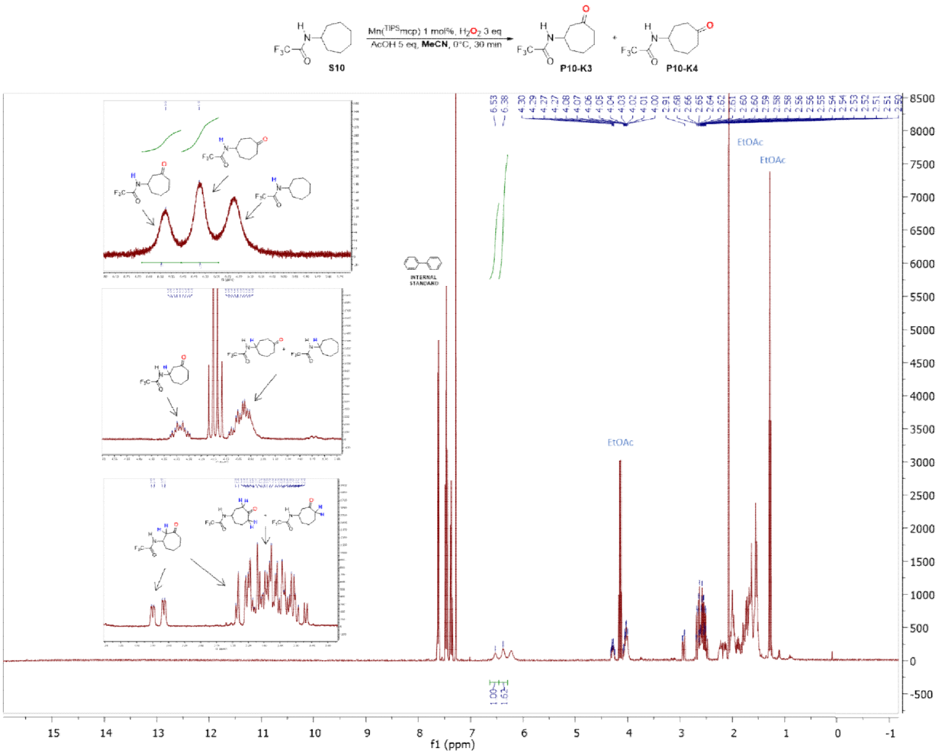

Figure S79. Crude mixture ^{1}H-NMR spectrum (400 MHz, $CDCl_3$) of *N*-cycloheptyl-2,2,2-trifluoroacetamide (**S10**) oxidation in MeCN.

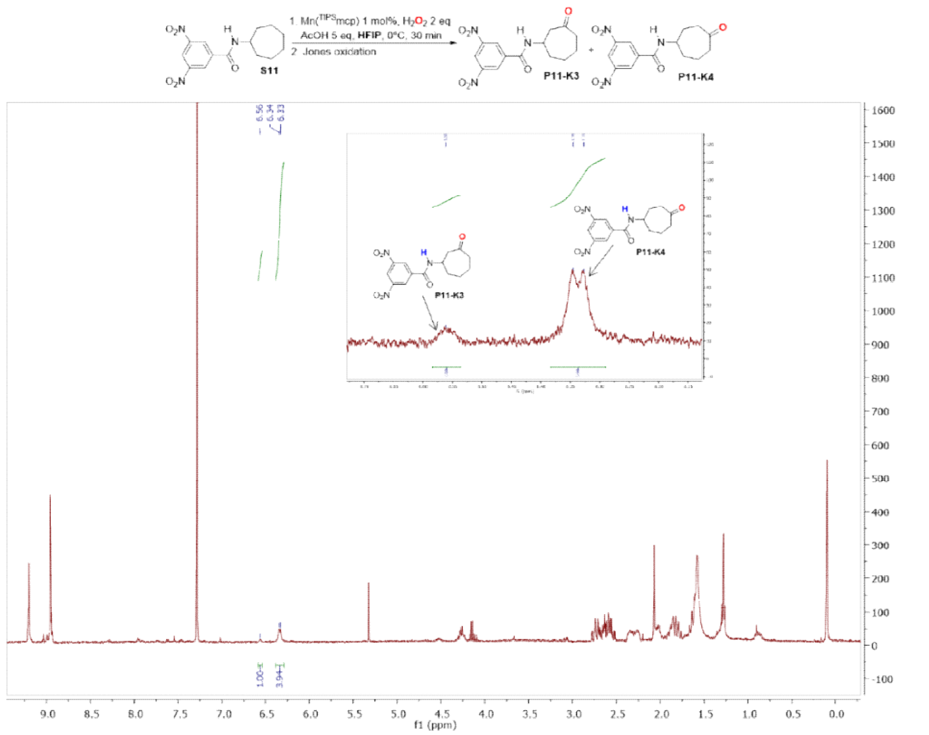

Figure S80. Crude mixture ^{1}H-NMR spectrum (400 MHz, $CDCl_3$) of *N*-cycloheptyl-3,5-dinitrobenzamide (**S11**) oxidation in HFIP.

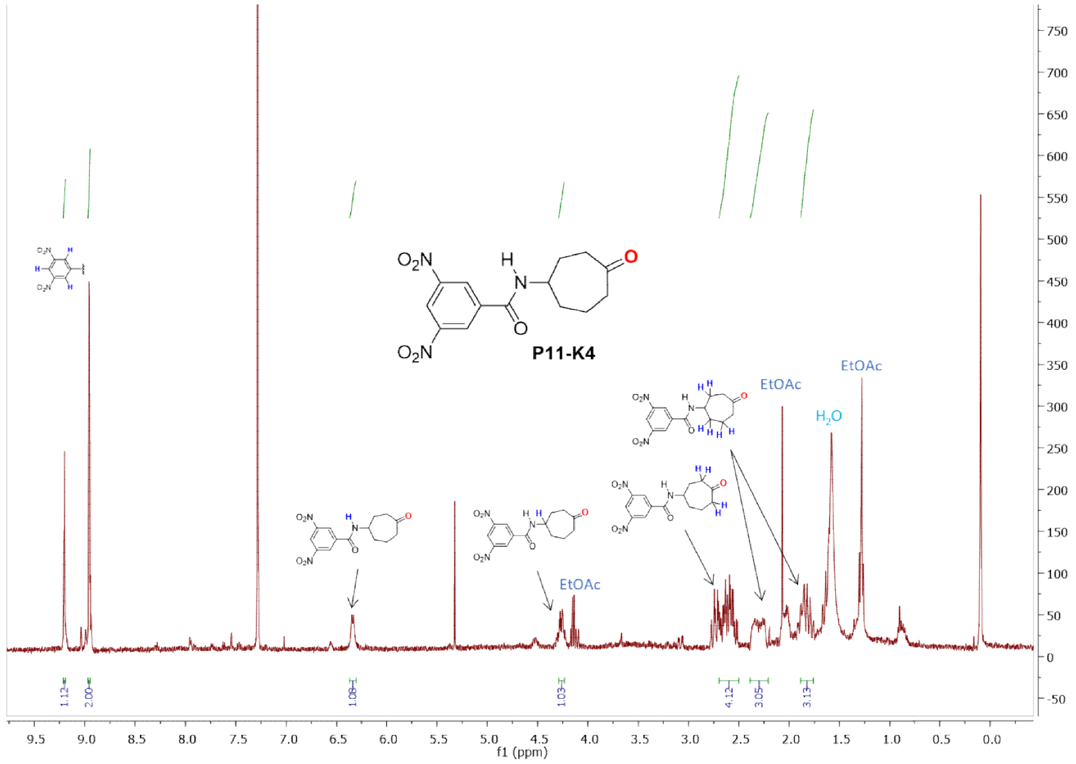

Figure S81. Crude mixture ^{1}H-NMR spectrum (400 MHz, $CDCl_3$) of *N*-cycloheptyl-3,5-dinitrobenzamide (**S11**) oxidation in HFIP: Identification of **P11-K4**.

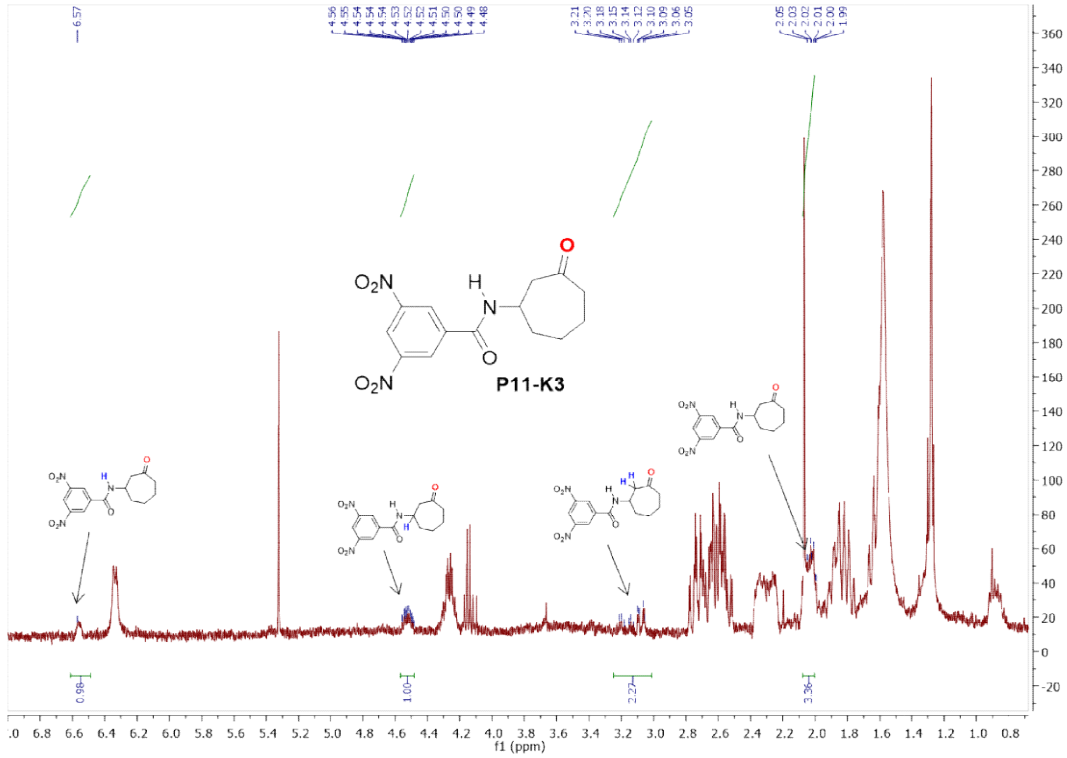

Figure S82. Crude mixture ^{1}H-NMR spectrum (400 MHz, $CDCl_3$) of *N*-cycloheptyl-3,5-dinitrobenzamide (**S11**) oxidation in HFIP: Identification of **P11-K3**.

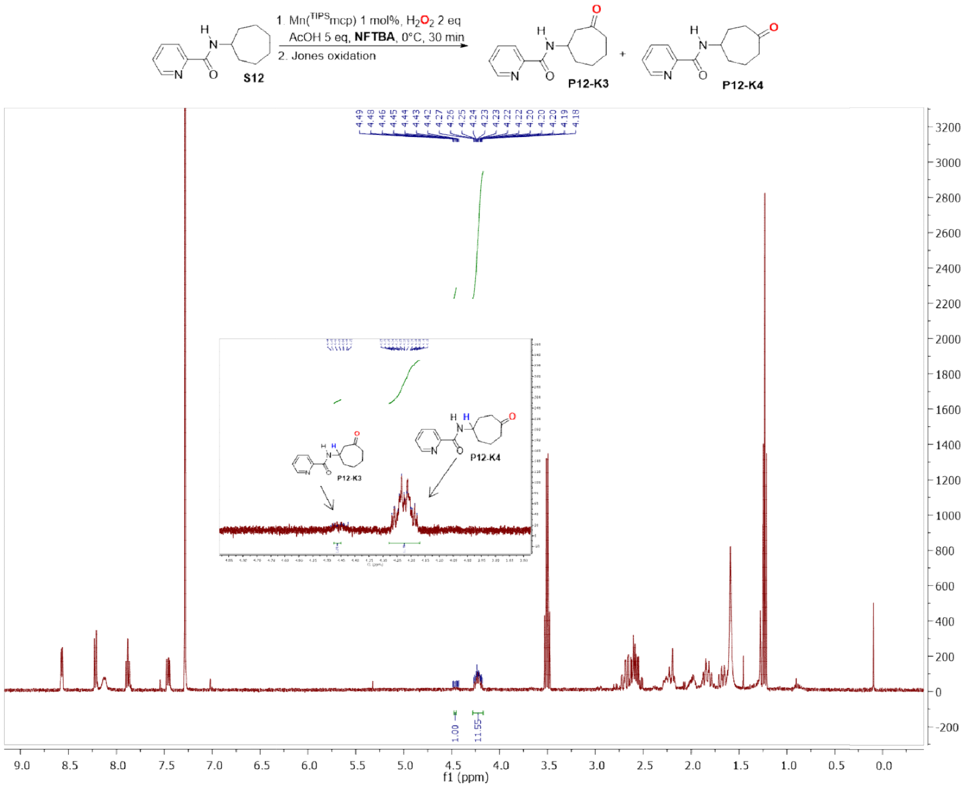

Figure S83. Crude mixture ^{1}H-NMR spectrum (400 MHz, $CDCl_3$) of *N*-cycloheptyl 2-pyridinecarboxamide (**S12**) oxidation in NFTBA.

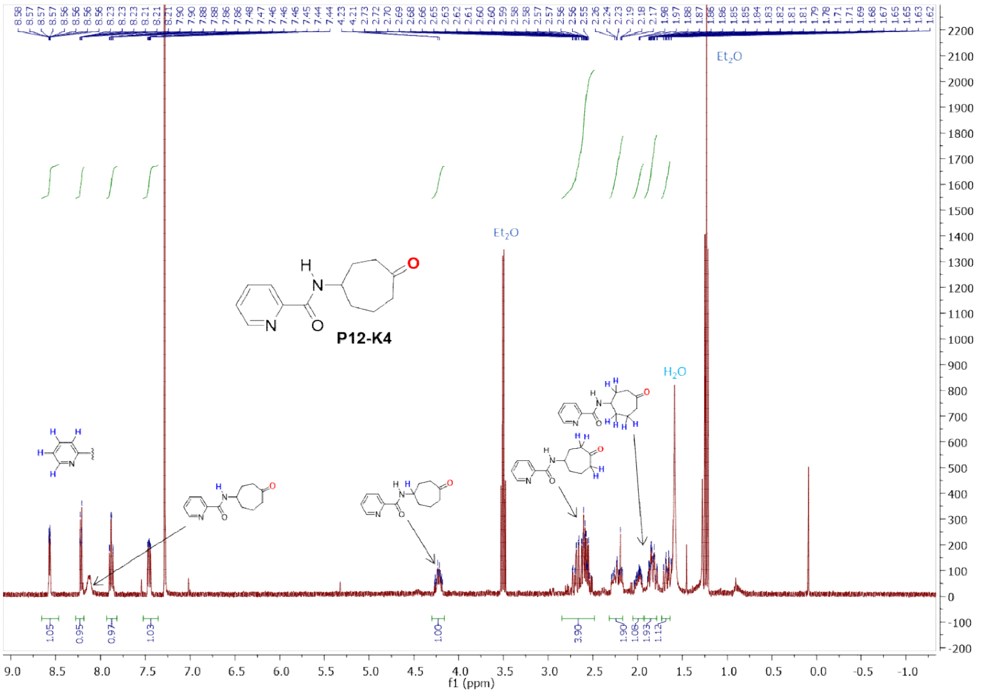

Figure S84. Crude mixture ^{1}H-NMR spectrum (400 MHz, $CDCl_3$) of *N*-cycloheptyl 2-pyridinecarboxamide (**S12**) oxidation in NFTBA: Identification of **P12-K4**.

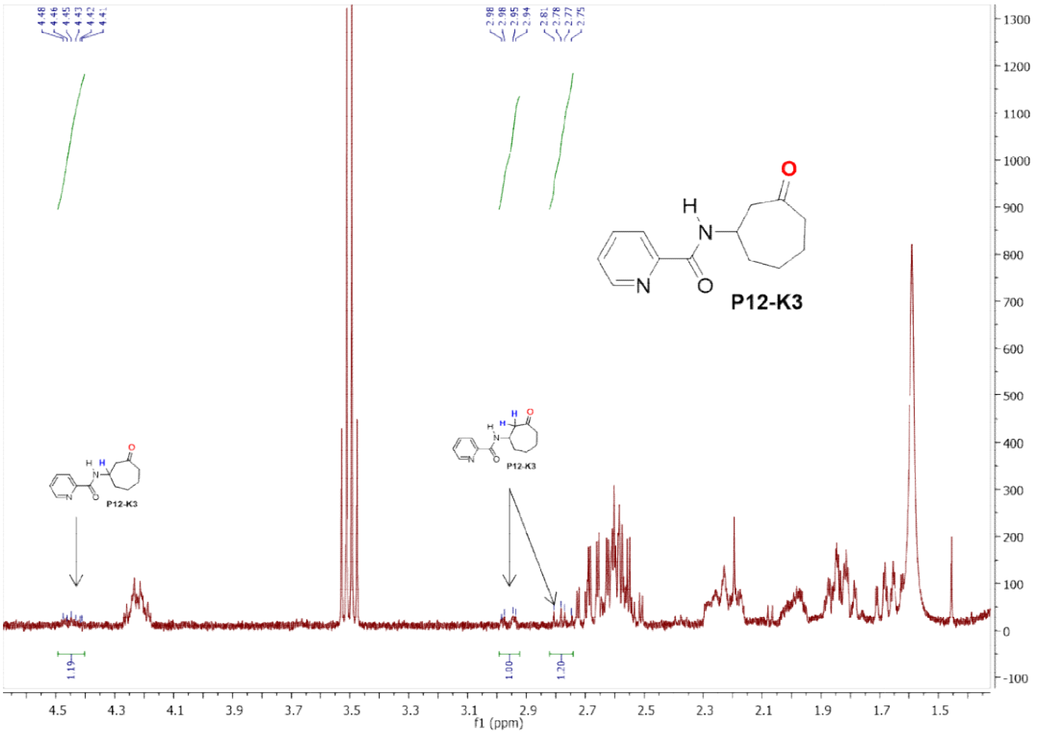

Figure S85. Crude mixture ^{1}H-NMR spectrum (400 MHz, $CDCl_3$) of *N*-cycloheptyl 2-pyridinecarboxamide (**S12**) oxidation in NFTBA: Identification of **P12-K3**.

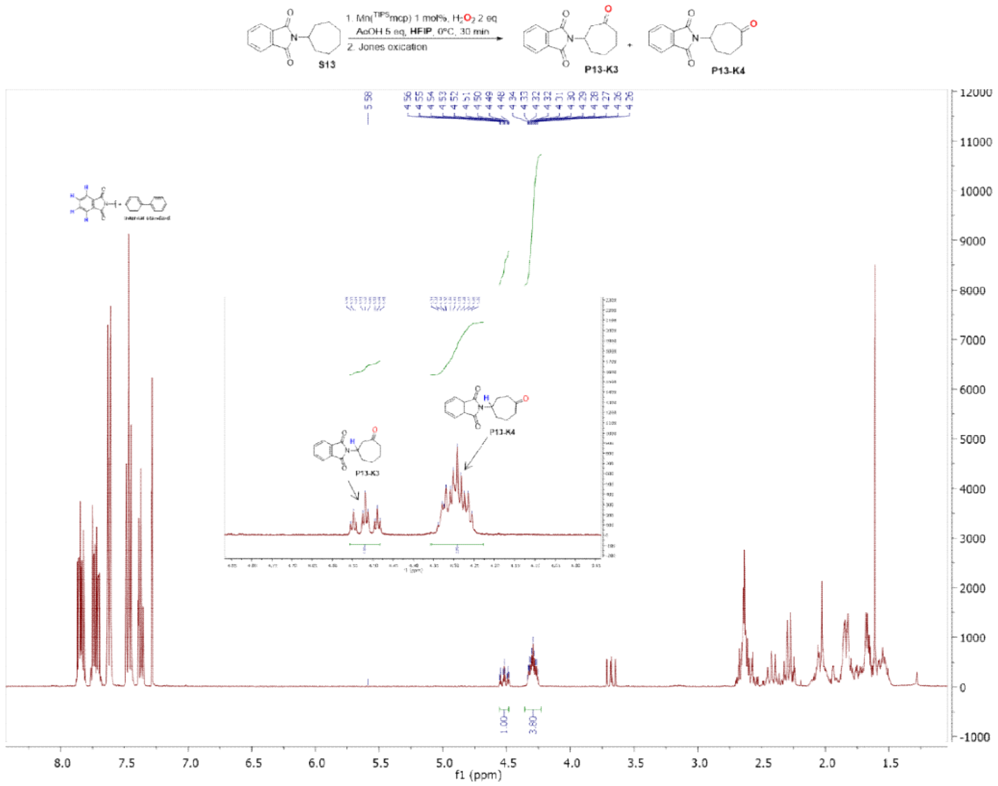

Figure S86. Crude mixture ^{1}H-NMR spectrum (400 MHz, $CDCl_3$) of *N*-cycloheptylphtalimide (**S13**) oxidation in HFIP.

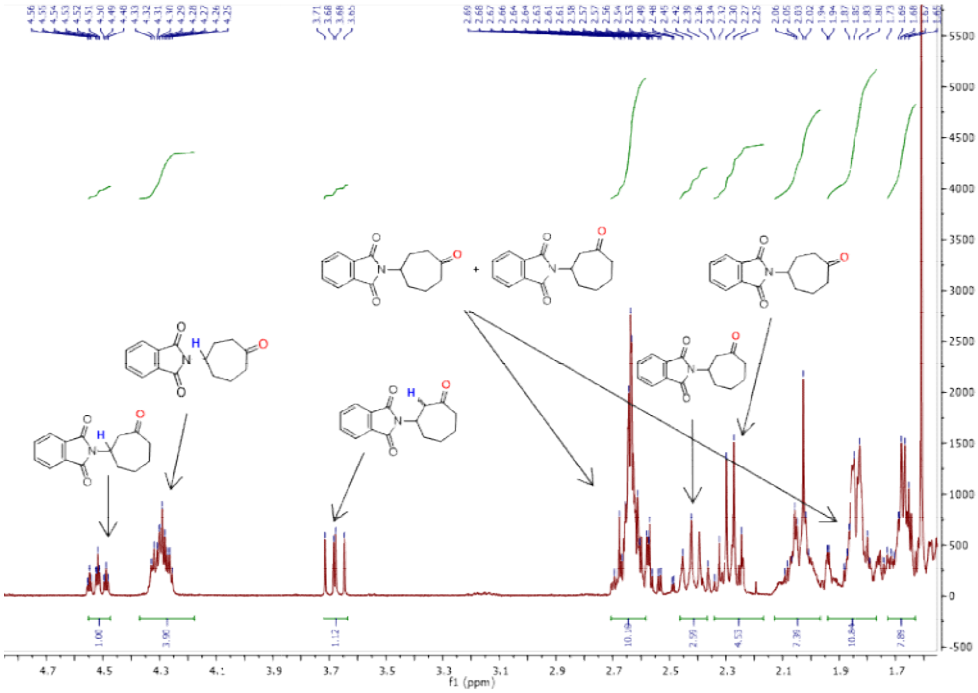

Figure S87. Crude mixture ^{1}H-NMR spectrum (400 MHz, $CDCl_3$) of of *N*-cycloheptylphtalimide (**S13**) oxidation in HFIP: Identification of **P13-K4** and **P13-K3**.

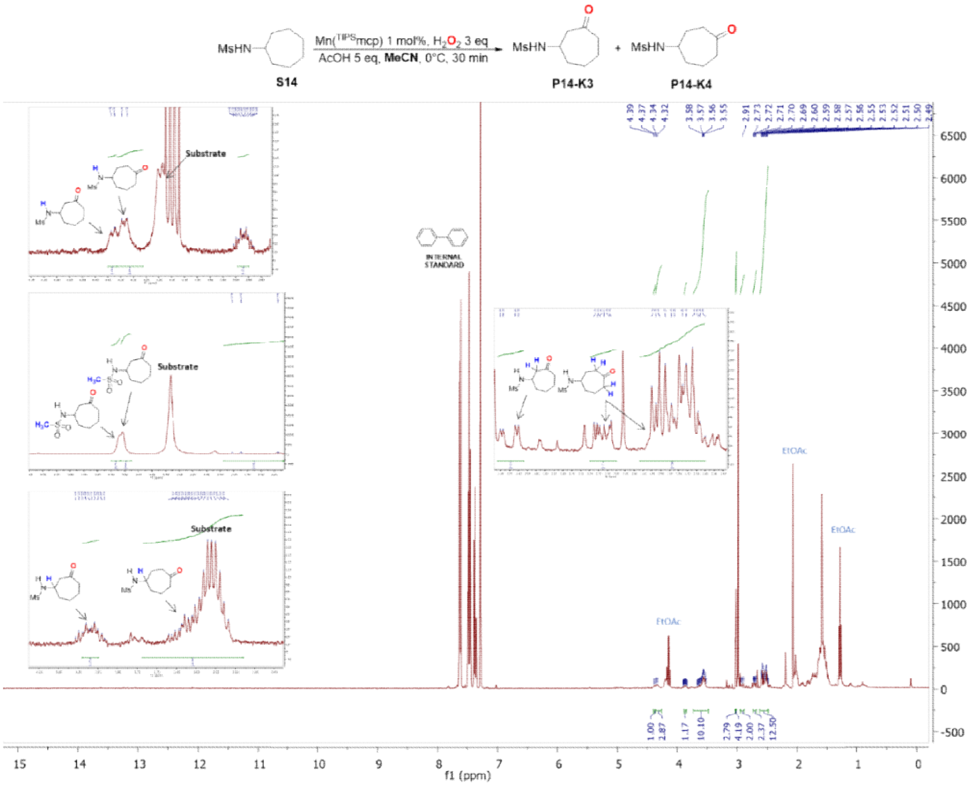

Figure S88. Crude mixture ^{1}H-NMR spectrum (400 MHz, $CDCl_3$) of *N*-cycloheptylmethanesulfonamide (**S14**) oxidation in MeCN.

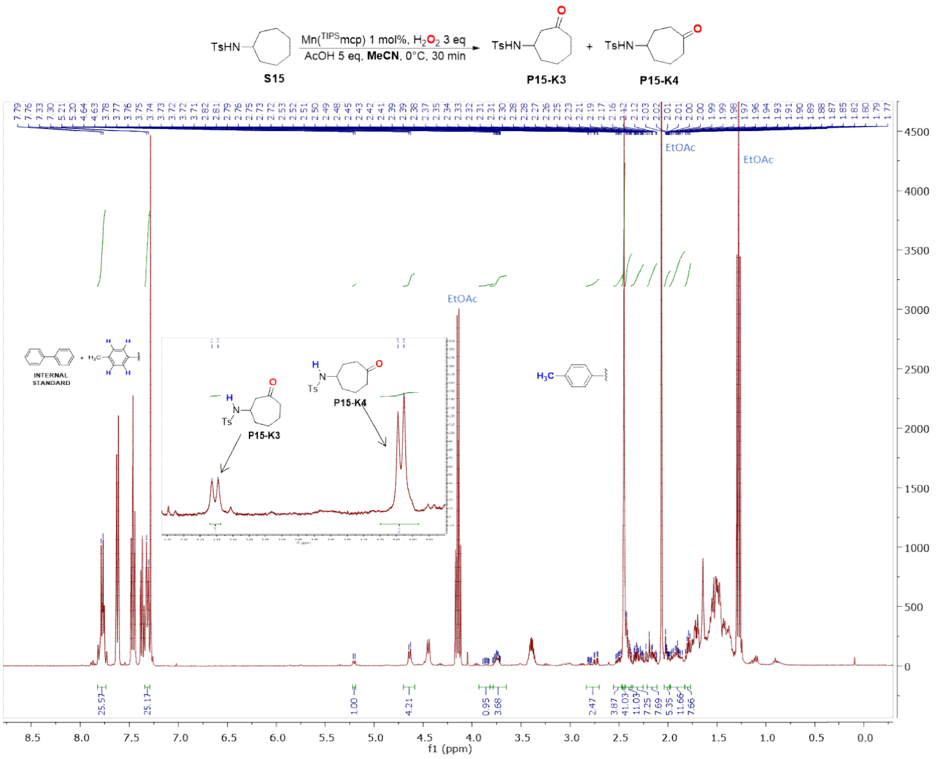

Figure S89. Crude mixture ^{1}H-NMR spectrum (400 MHz, $CDCl_3$) of *N*-cycloheptyl-4-methylbenzenesulfonamide (**S15**) oxidation in MeCN.

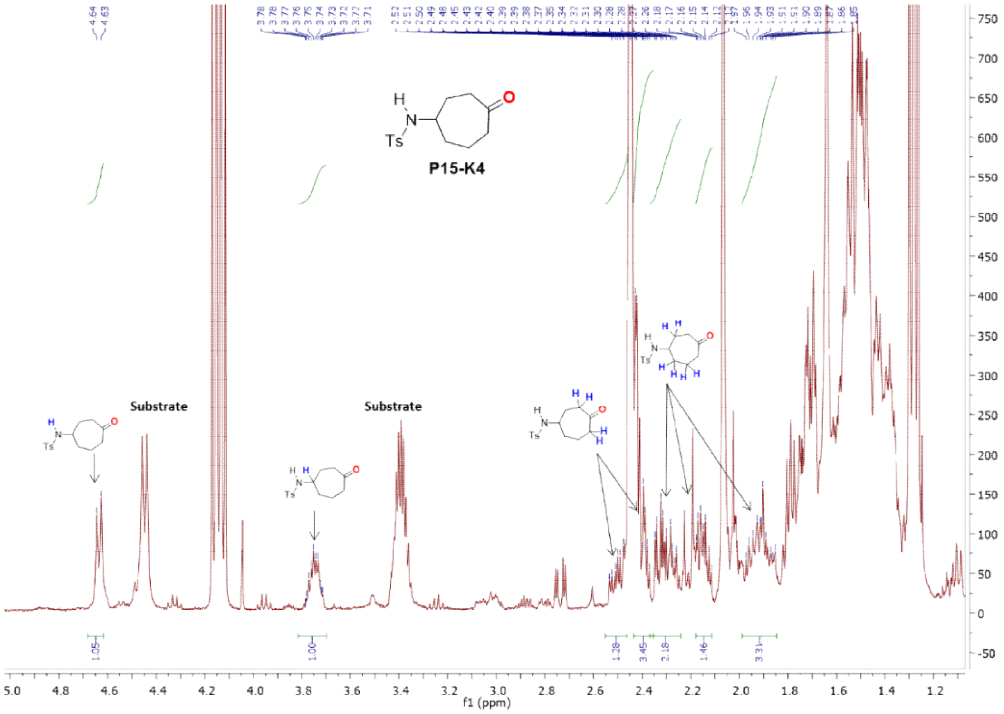

Figure S90. Crude mixture ^{1}H-NMR spectrum (400 MHz, $CDCl_3$) of *N*-cycloheptyl-4-methylbenzenesulfonamide (**S15**) oxidation in MeCN: Identification of **P15-K4**.

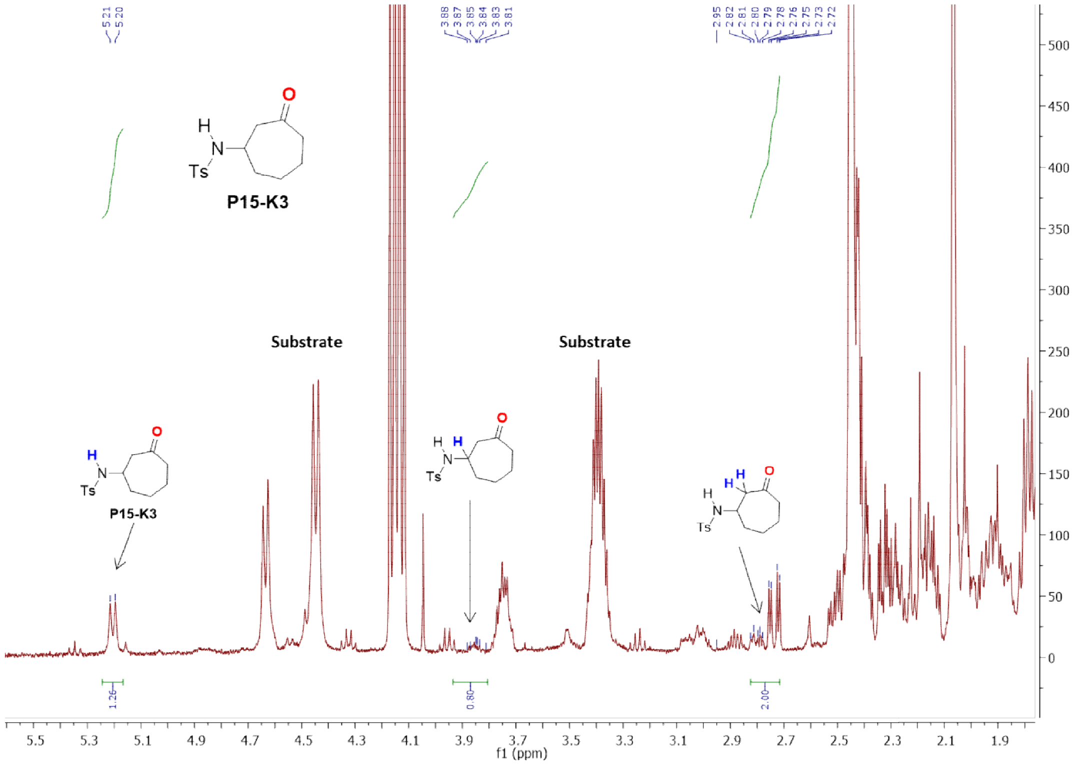

Figure S91. Crude mixture ^{1}H-NMR spectrum (400 MHz, $CDCl_3$) of *N*-cycloheptyl-4-methylbenzenesulfonamide (**S15**) oxidation in MeCN: Identification of **P15-K3**.

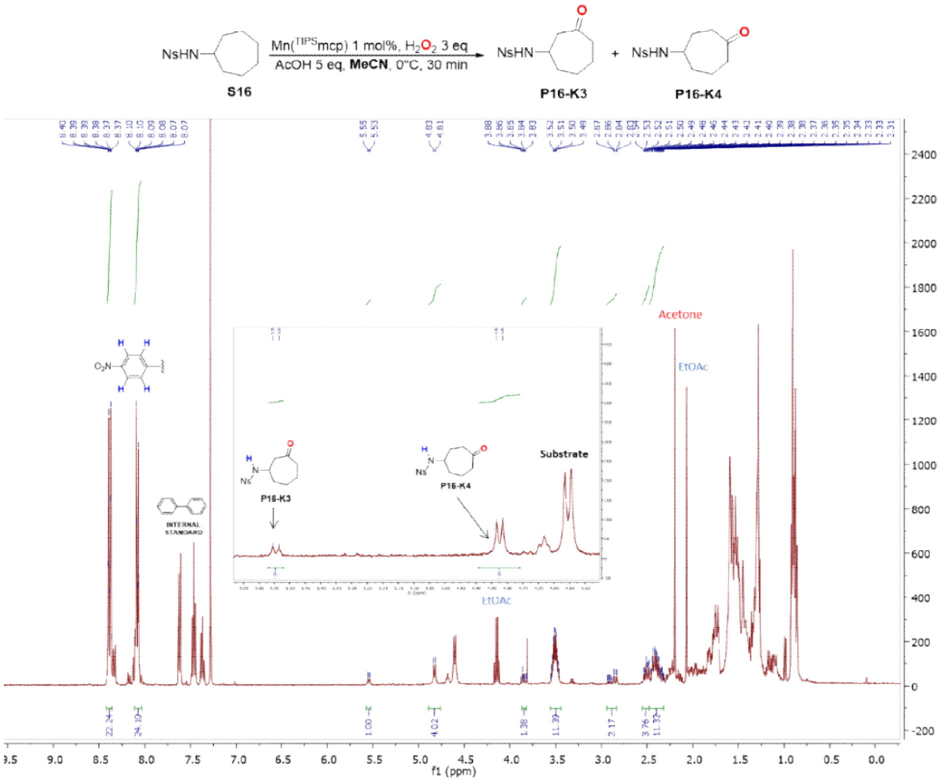

Figure S92. Crude mixture ^{1}H-NMR spectrum (400 MHz, $CDCl_3$) of *N*-cycloheptyl-4-nitrobenzenesulfonamide (**S16**) oxidation in MeCN.

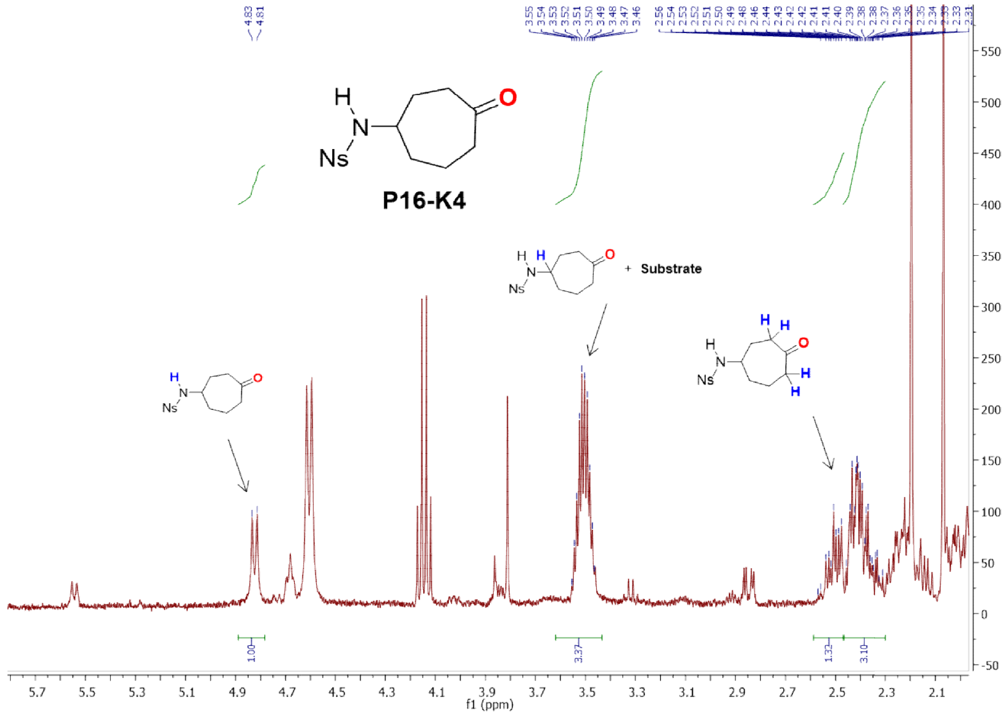

Figure S93. Crude mixture ¹H-NMR spectrum (400 MHz, $CDCl_3$) of *N*-cycloheptyl-4-nitrobenzenesulfonamide (**S16**) oxidation in MeCN: Identification of **P16-K4**.

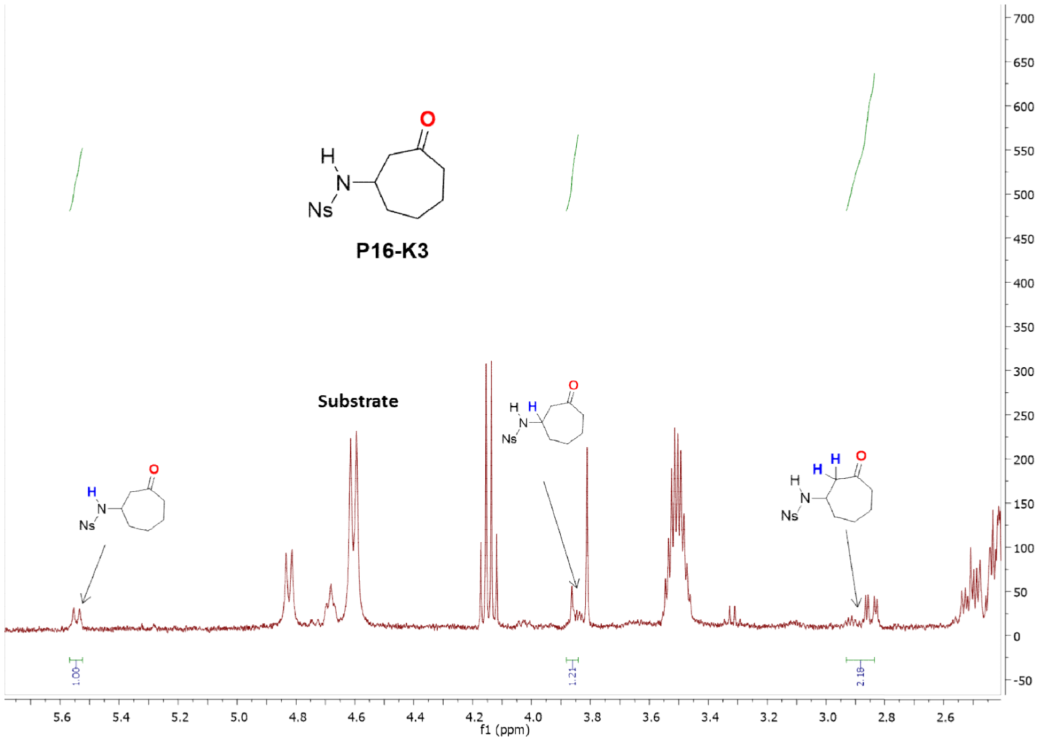

Figure S94. Crude mixture ¹H-NMR spectrum (400 MHz, $CDCl_3$) of *N*-cycloheptyl-4-nitrobenzenesulfonamide (**S16**) oxidation in MeCN: Identification of **P16-K3**.

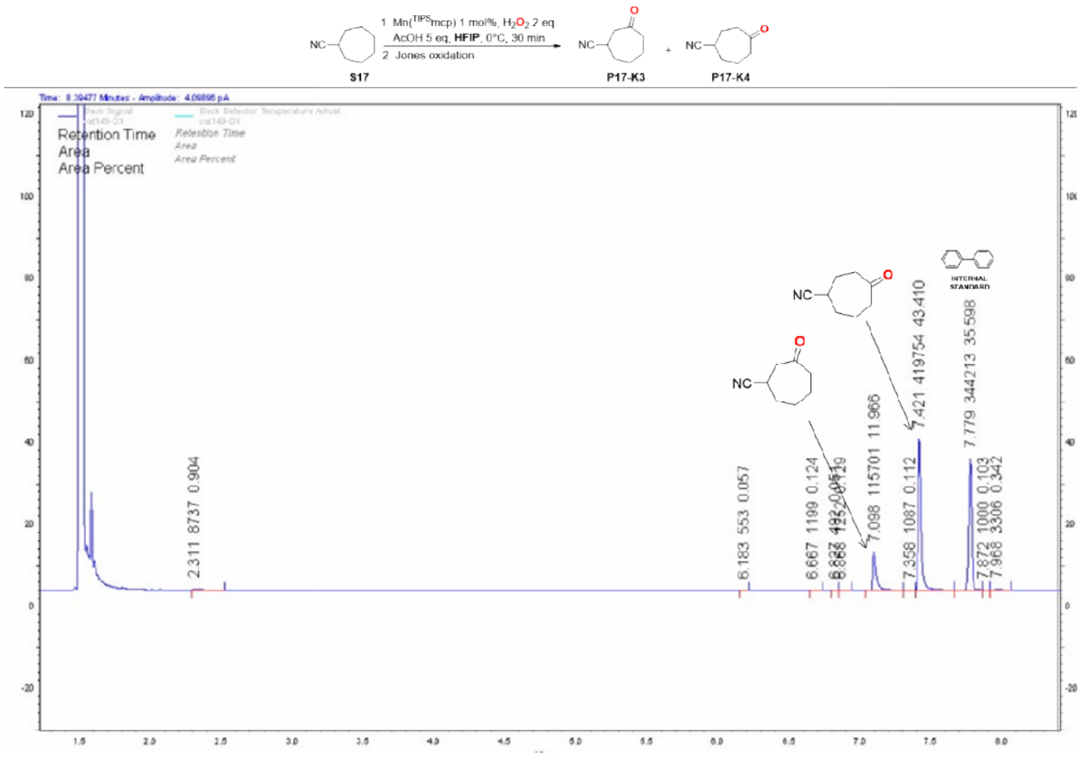

Figure S95. Crude mixture chromatogram of cycloheptanecarbonitrile (**S17**) oxidation in HFIP.

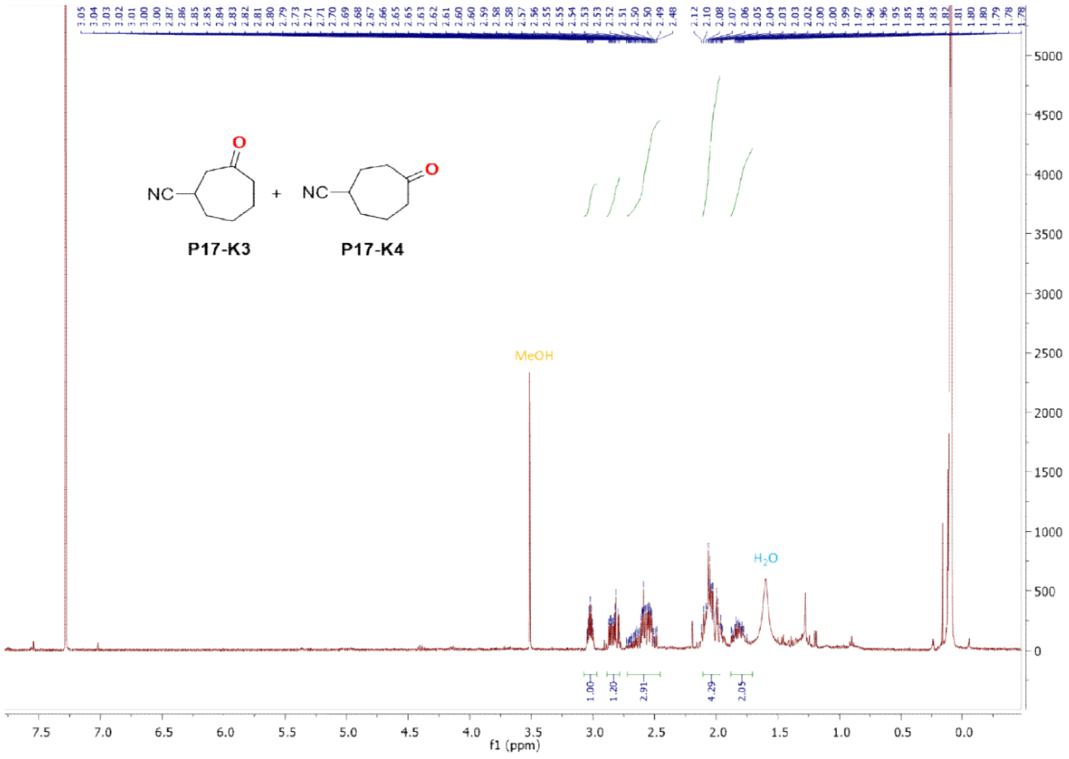

Figure S96. Crude mixture ^{1}H-NMR spectrum (400 MHz, $CDCl_3$) of cycloheptanecarbonitrile (**S17**) oxidation in HFIP.

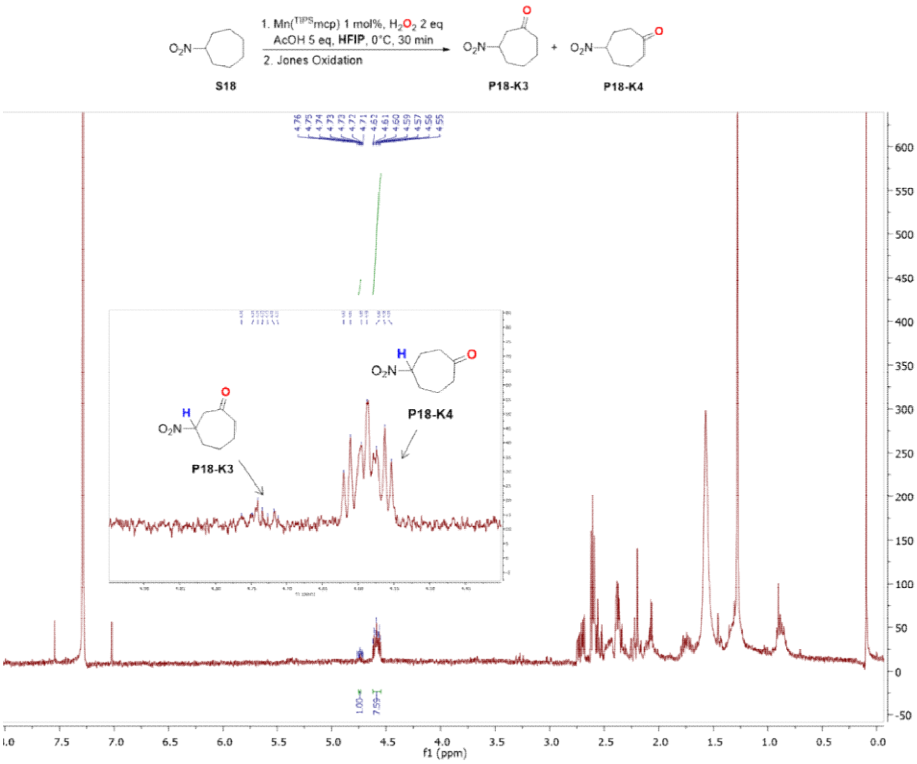

Figure S97. Crude mixture ^{1}H-NMR spectrum (400 MHz, $CDCl_3$) of nitrocycloheptane (**S18**) oxidation in NFTBA.

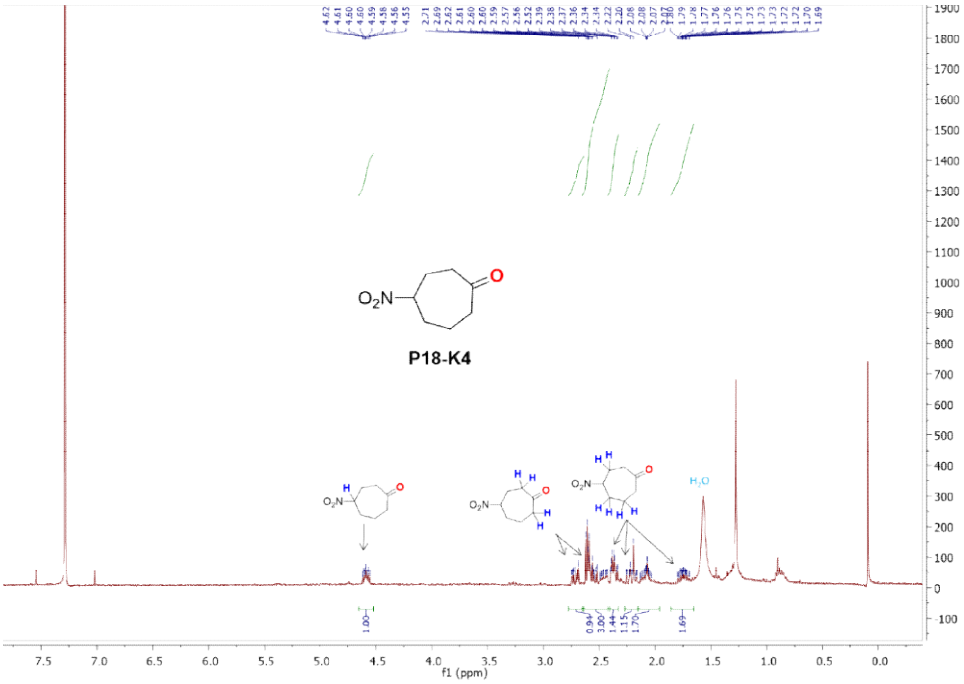

Figure S98. Crude mixture ^{1}H-NMR spectrum (400 MHz, $CDCl_3$) of nitrocycloheptane (**S18**) oxidation in NFTBA: Identification of **P18-K4**.

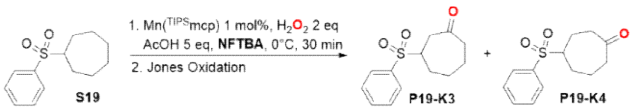

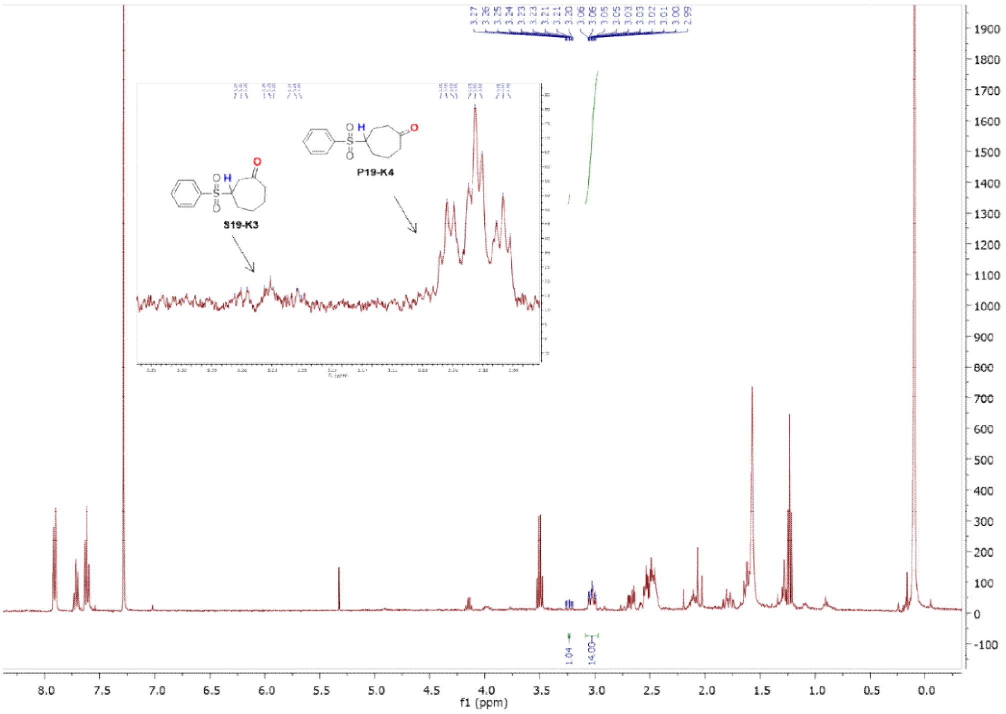

Figure S99. Crude mixture ^{1}H-NMR spectrum (400 MHz, $CDCl_3$) of (phenylsulfonyl)cycloheptane (**S19**) oxidation in NFTBA.

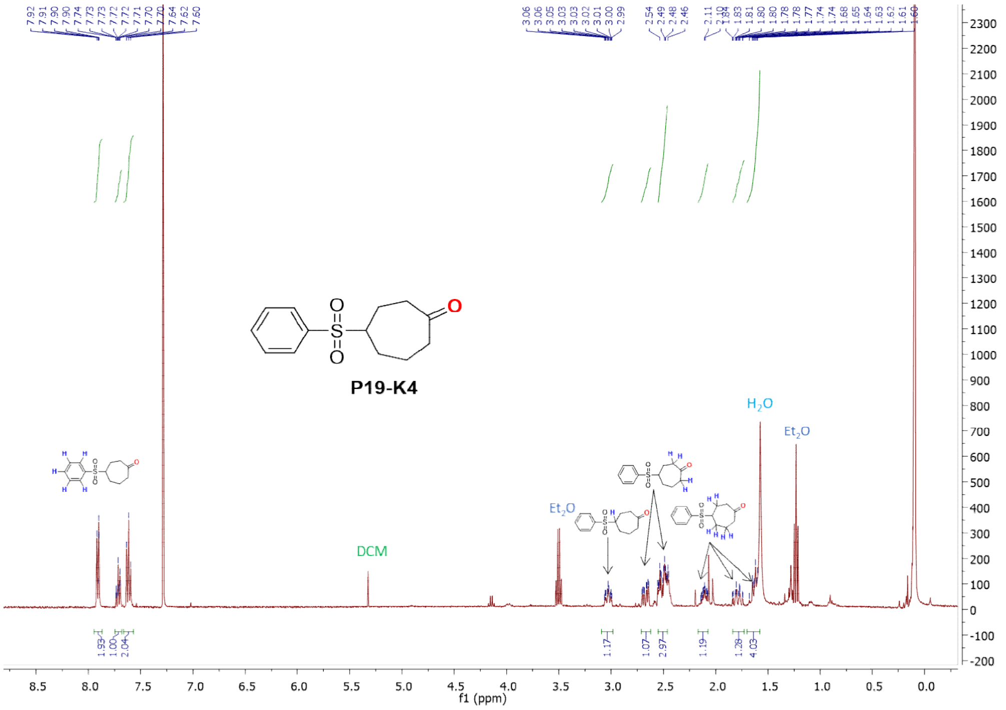

Figure S100. Crude mixture ^{1}H-NMR spectrum (400 MHz, $CDCl_3$) of (phenylsulfonyl)cycloheptane (**S19**) oxidation in NFTBA: Identification of **P19-K4**.

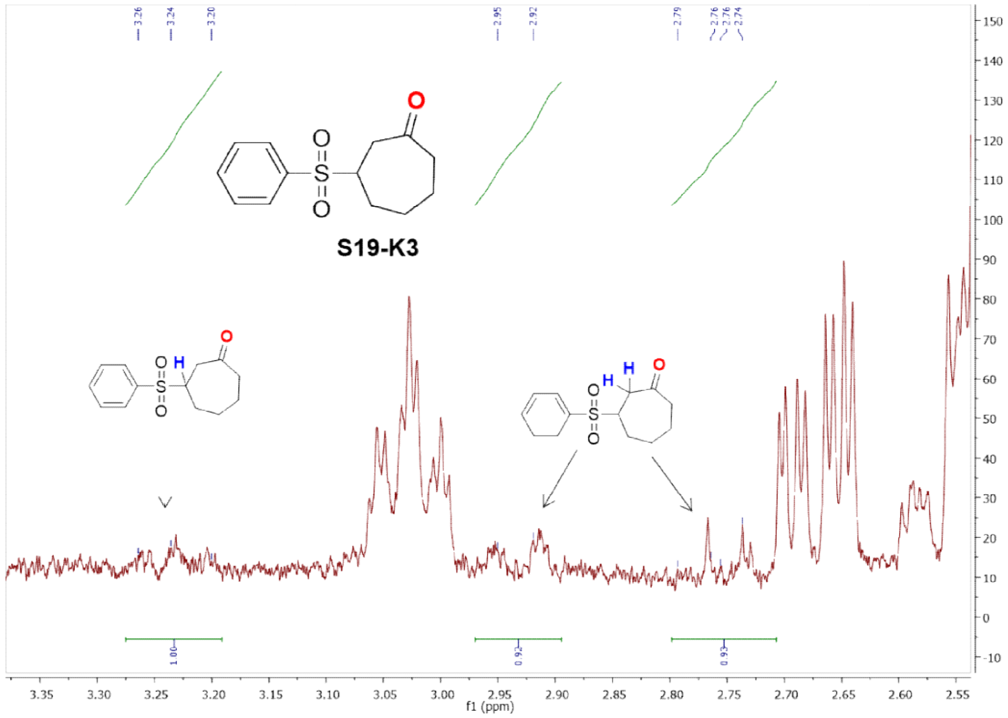

Figure S101. Crude mixture ^{1}H-NMR spectrum (400 MHz, $CDCl_3$) of (phenylsulfonyl)cycloheptane (**S19**) oxidation in NFTBA: Identification of **P19-K3**.

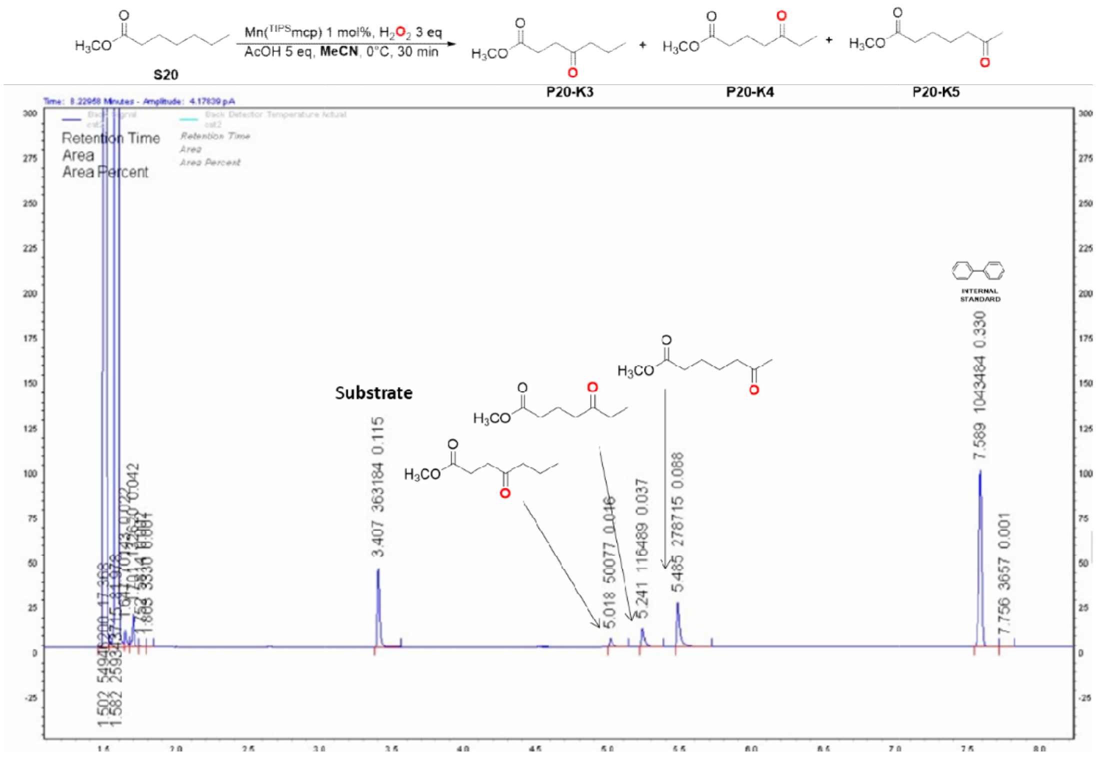

Figure S102. Crude mixture chromatogram of methylheptanoate (**S20**) oxidation in MeCN

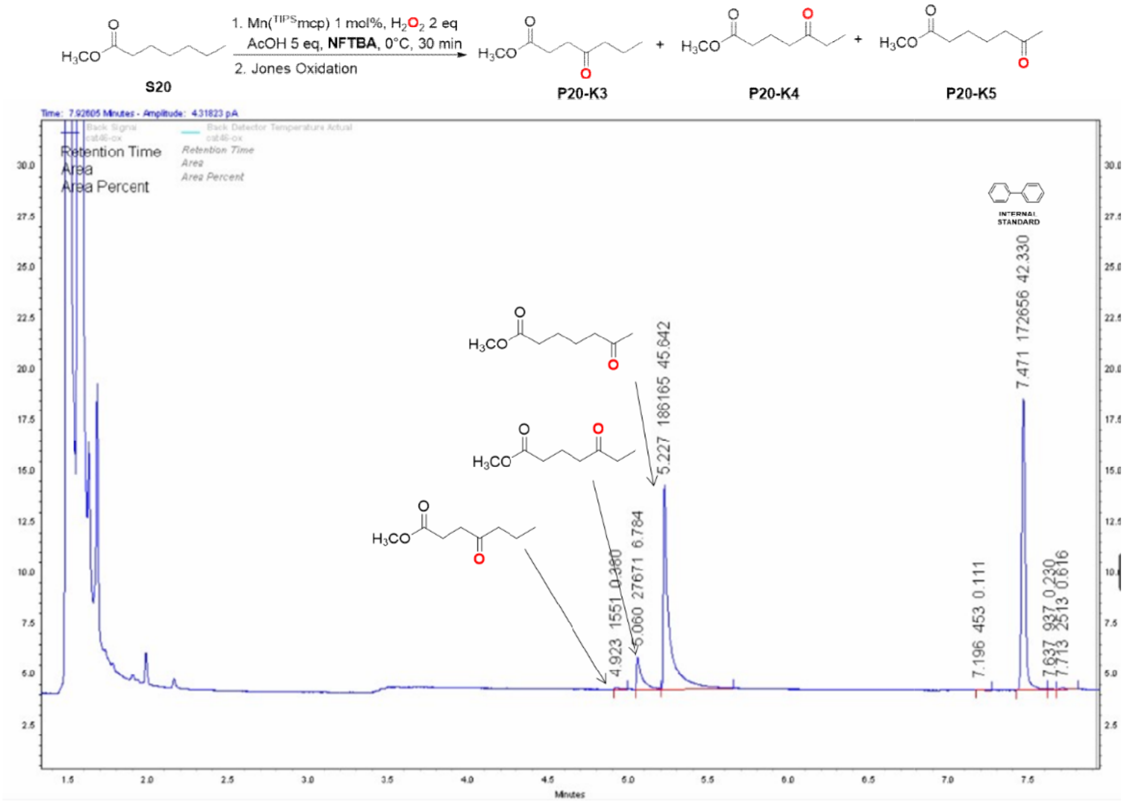

Figure S103. Crude mixture chromatogram of methylheptanoate (**S20**) oxidation in MeCN

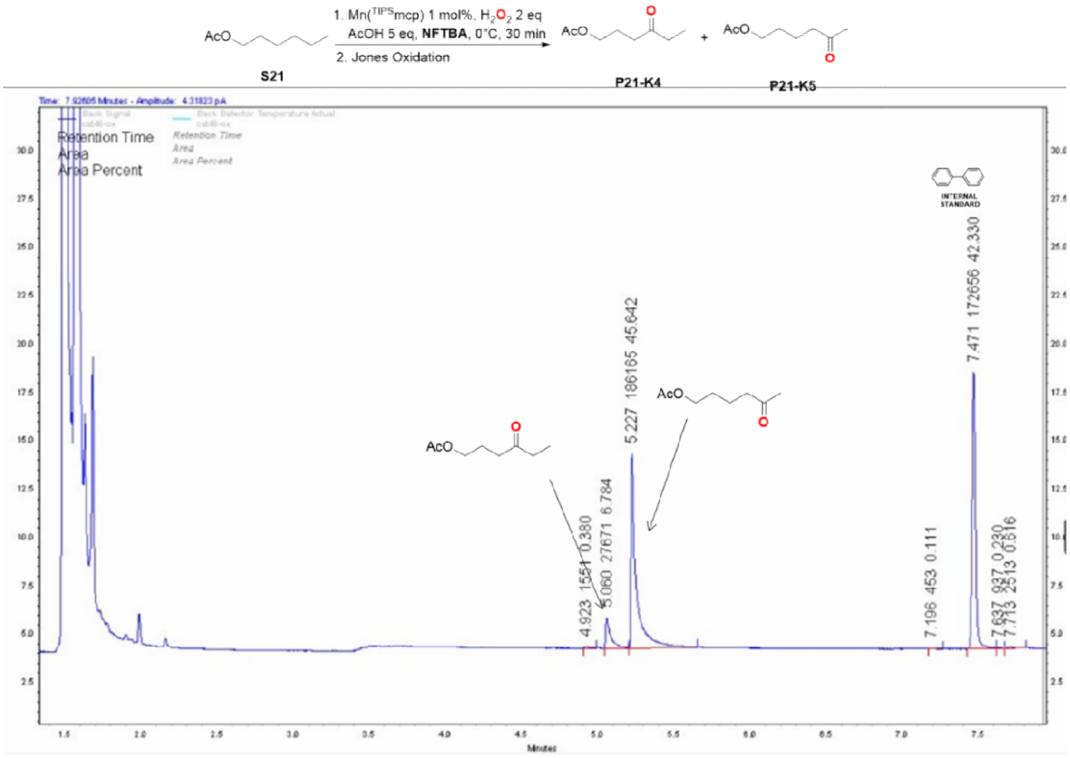

Figure S104. Crude mixture chromatogram of 1-hexy lacetate (**S21**) oxidation in NFTBA

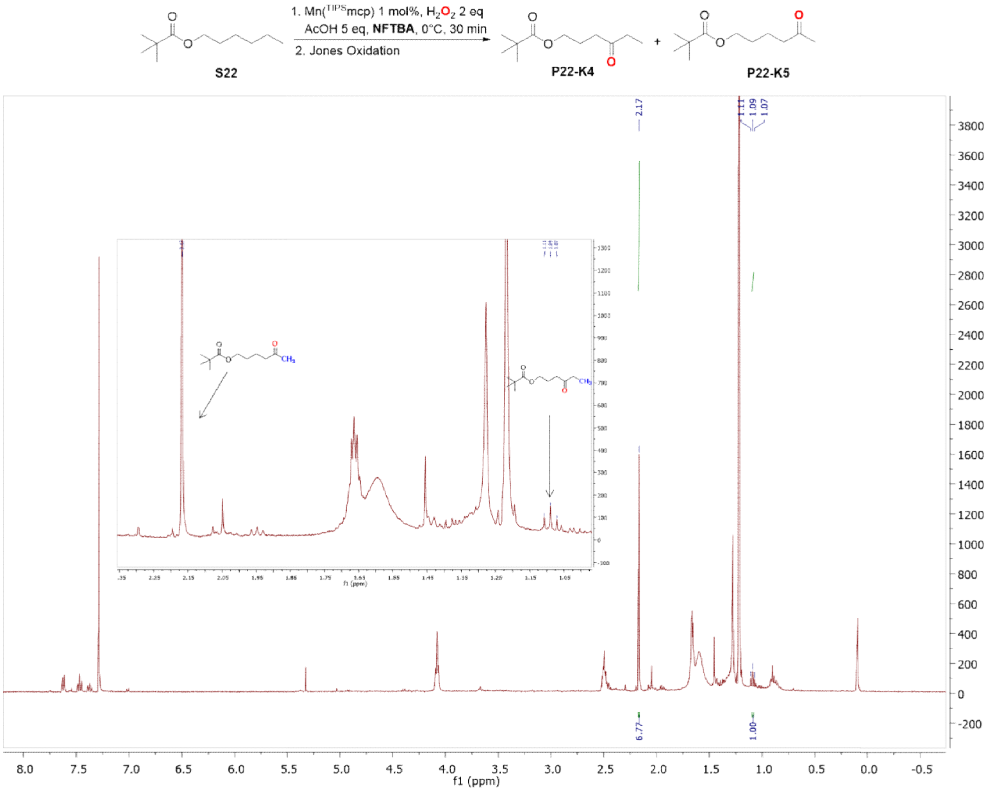

Figure S105. Crude mixture ^{1}H-NMR spectrum (400 MHz, $CDCl_3$) of 1-hexyl pivalate (**S22**) oxidation in NFTBA.

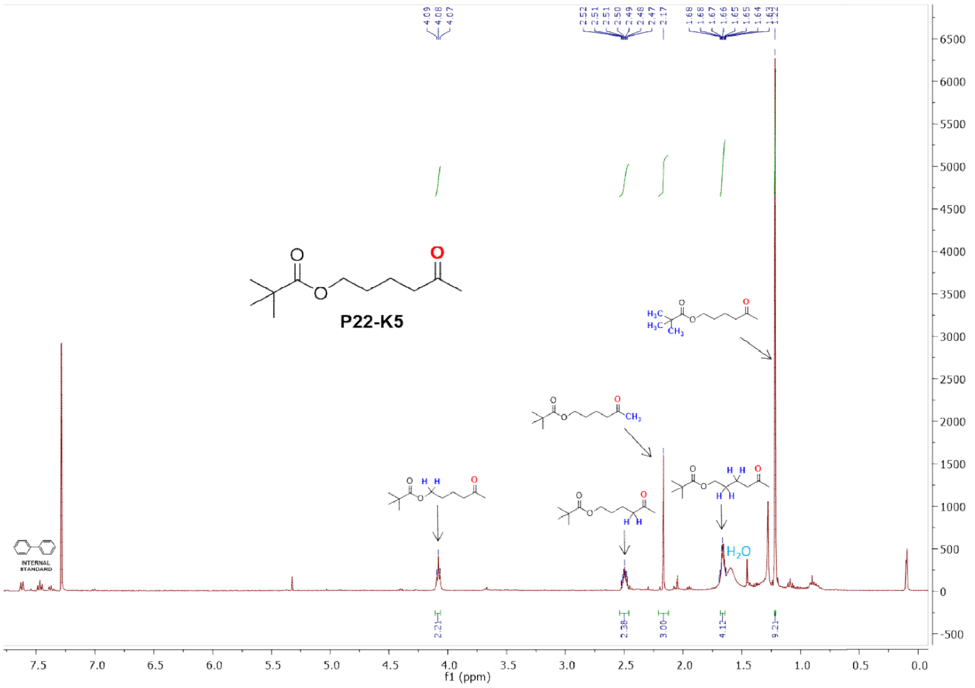

Figure S106. Crude mixture ^{1}H-NMR spectrum (400 MHz, $CDCl_3$) of 1-hexyl pivalate (**S22**) oxidation in NFTBA: Identification of **P22-K5**.

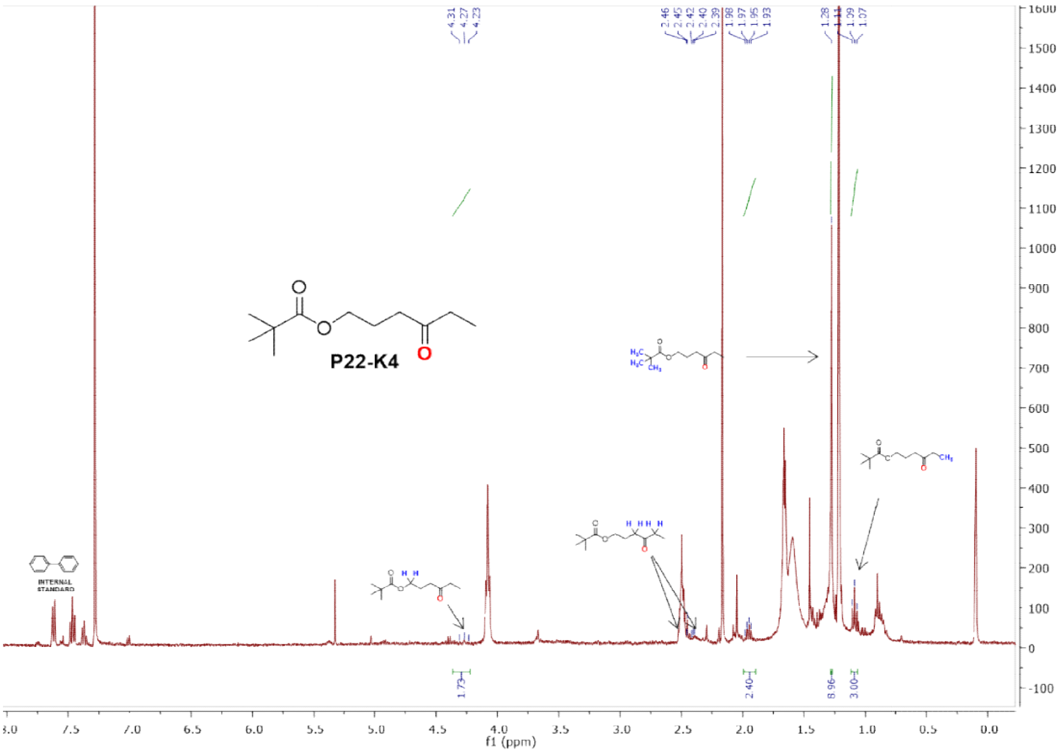

Figure S107. Crude mixture ^{1}H-NMR spectrum (400 MHz, $CDCl_3$) of 1-hexyl pivalate (**S22**) oxidation in NFTBA: Identification of **P22-K4**

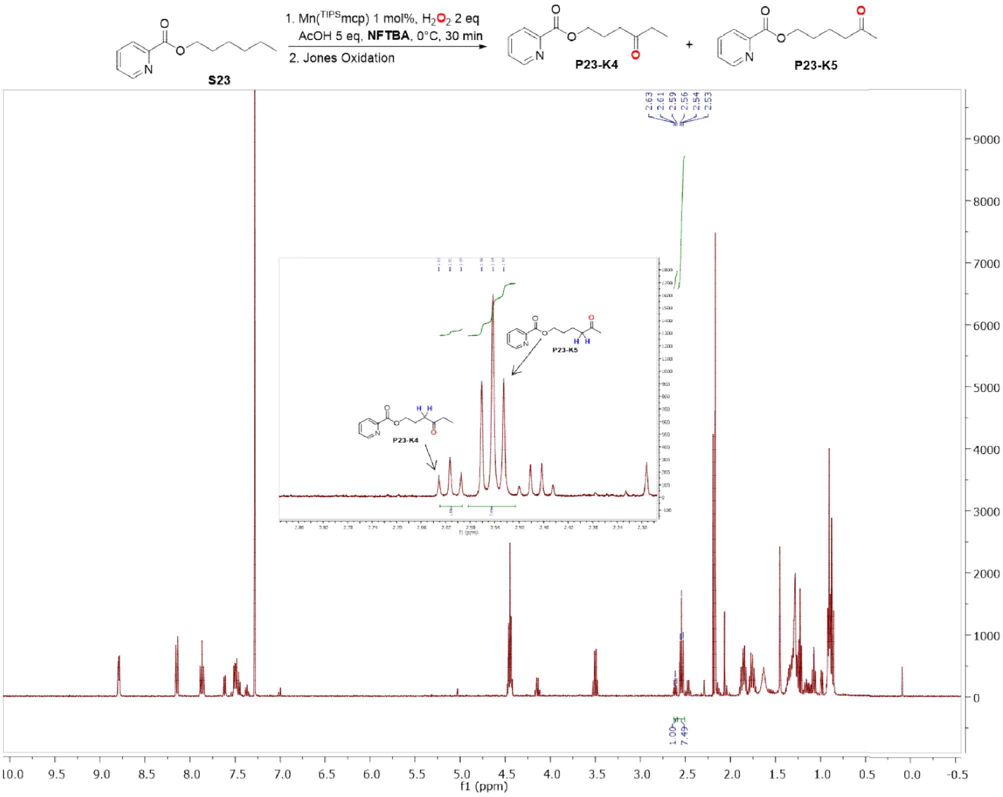

Figure S108. Crude mixture ^{1}H-NMR spectrum (400 MHz, $CDCl_3$) of 1-hexyl 2-pyridinecarboxylate (**S23**) oxidation in NFTBA.

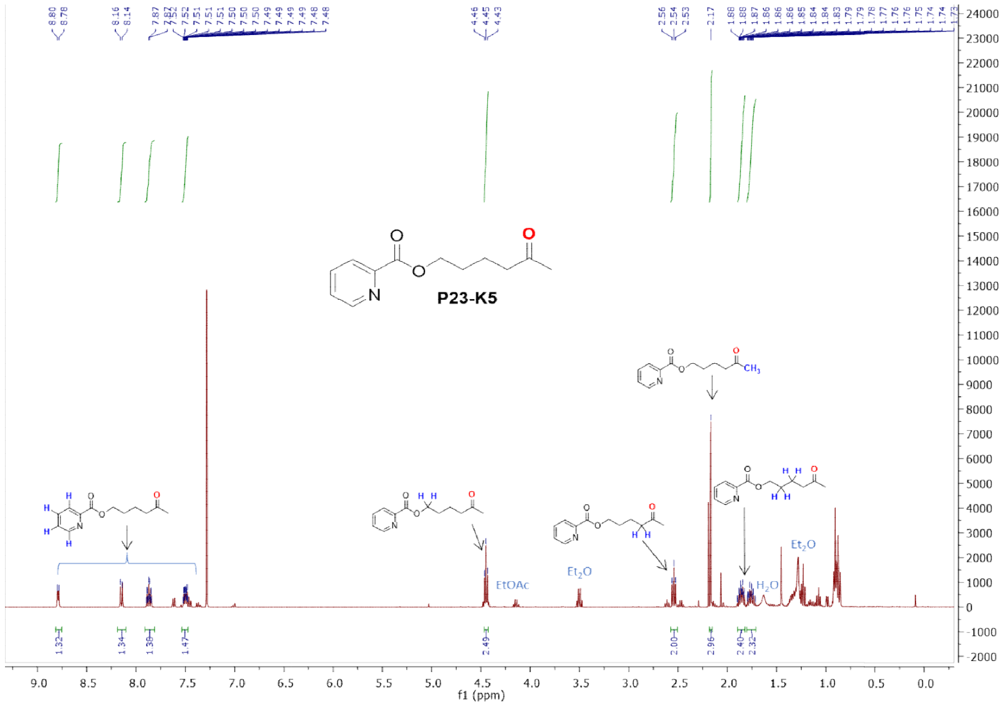

Figure S109. Crude mixture ^{1}H-NMR spectrum (400 MHz, $CDCl_3$) of 1-hexyl 2-pyridinecarboxylate (**S23**) oxidation in NFTBA: Identification of **P23-K5**.

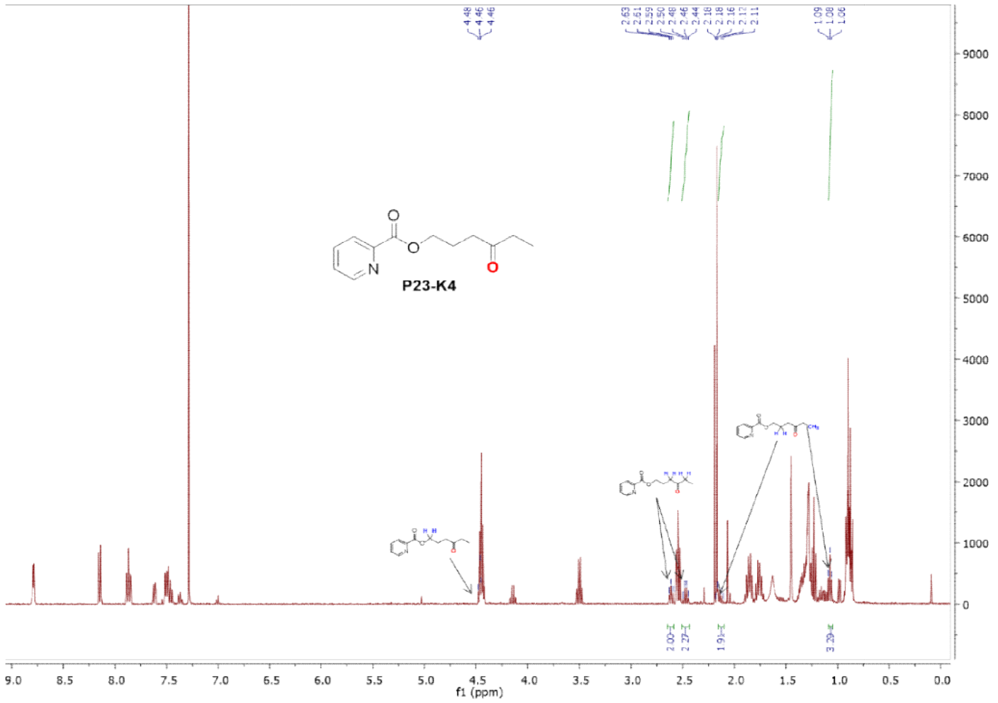

Figure S110. Crude mixture ^{1}H-NMR spectrum (400 MHz, $CDCl_3$) of 1-hexyl 2-pyridinecarboxylate (**S23**) oxidation in NFTBA: Identification of **P23-K4**.

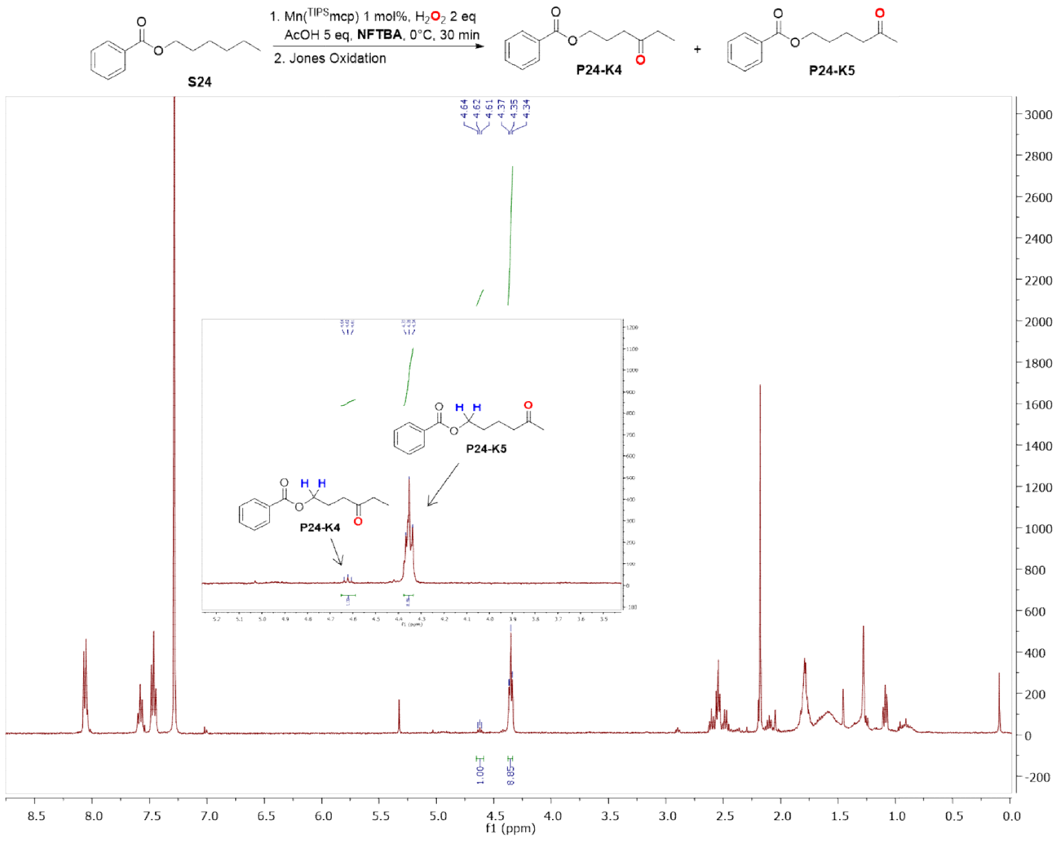

Figure S111. Crude mixture ^{1}H-NMR spectrum (400 MHz, $CDCl_3$) of 1-hexyl benzoate (**S24**) oxidation in NFTBA.

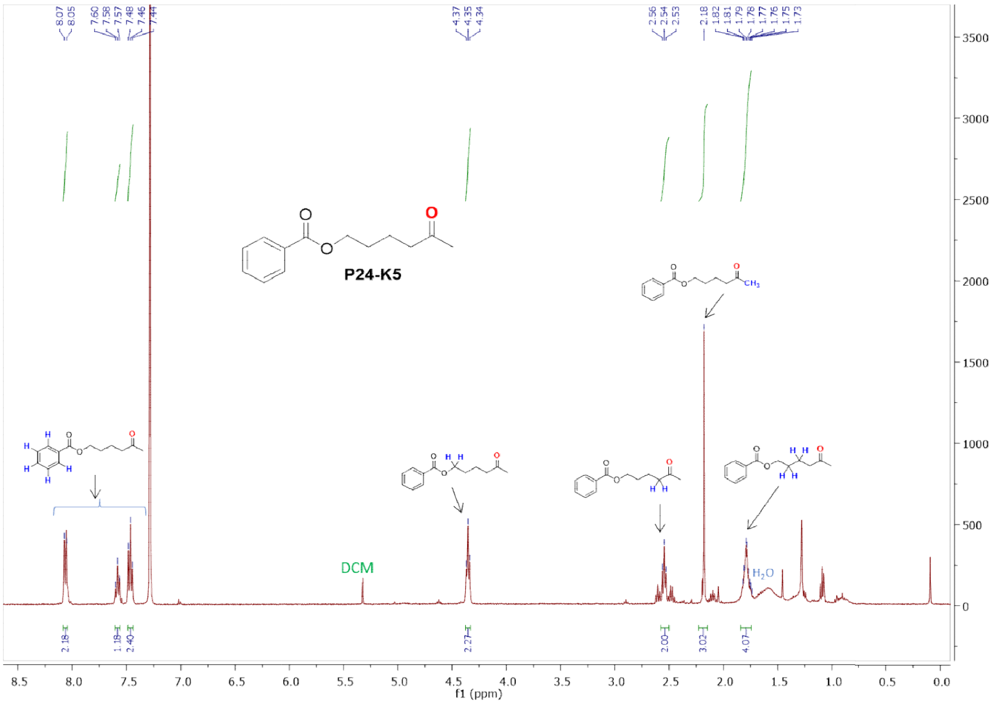

Figure S112. Crude mixture ^{1}H-NMR spectrum (400 MHz, $CDCl_3$) of 1-hexyl benzoate (**S24**) oxidation in NFTBA: Identification of **P24-K5**.

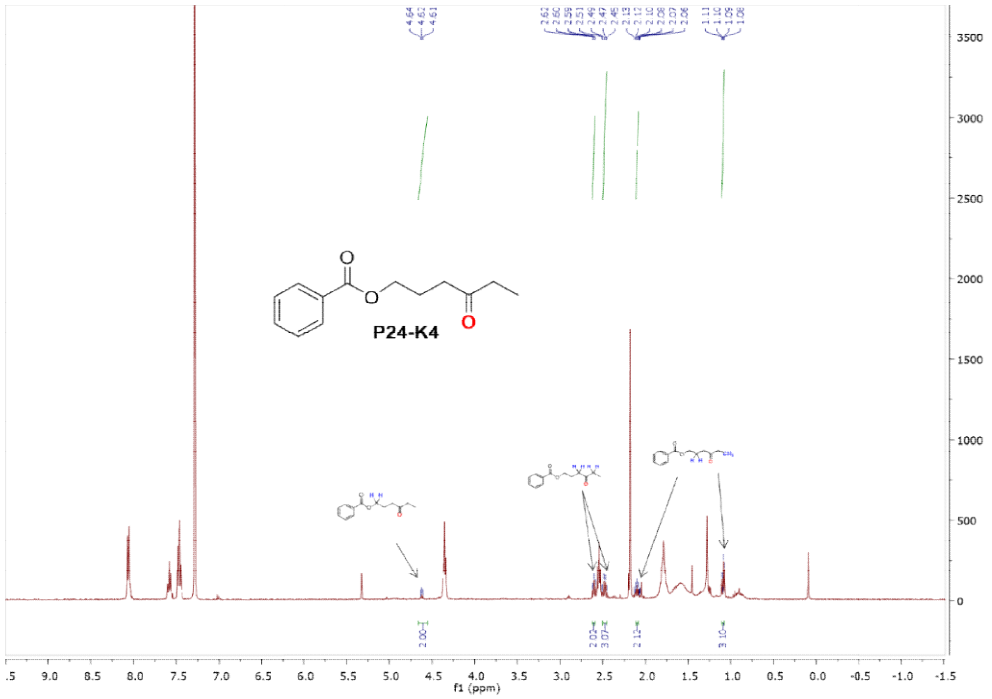

Figure S113. Crude mixture ^{1}H-NMR spectrum (400 MHz, $CDCl_3$) of 1-hexyl benzoate (**S24**) oxidation in NFTBA: Identification of **P24-K4**.

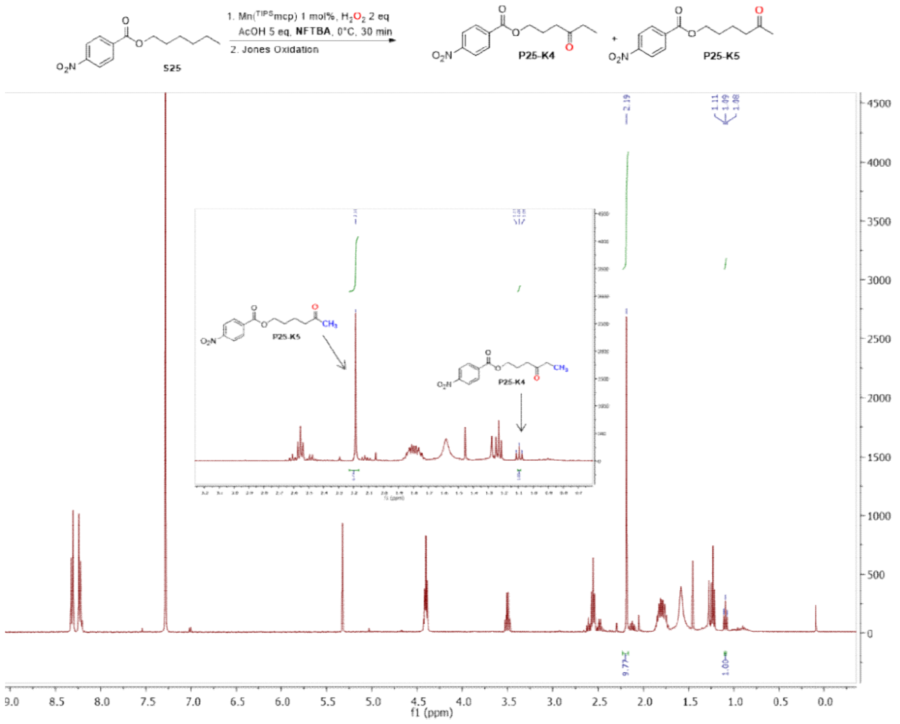

Figure S114. Crude mixture ^{1}H-NMR spectrum (400 MHz, $CDCl_3$) of 1-hexyl 4-nitrobenzoate (**S25**) oxidation in NFTBA.

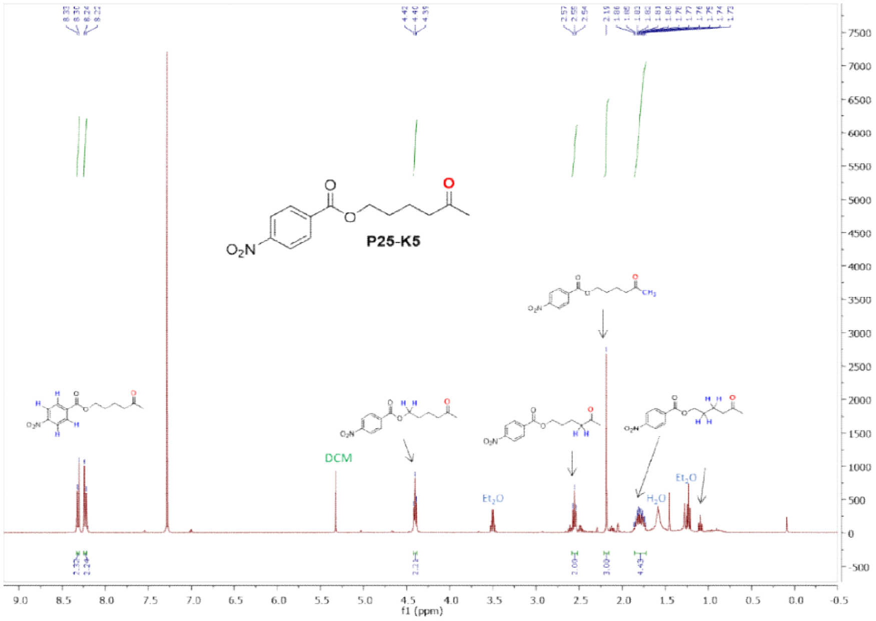

Figure S115. Crude mixture ^{1}H-NMR spectrum (400 MHz, $CDCl_3$) of 1-hexyl 4-nitrobenzoate (**S25**) oxidation in NFTBA: Identification of **P25-K5**.

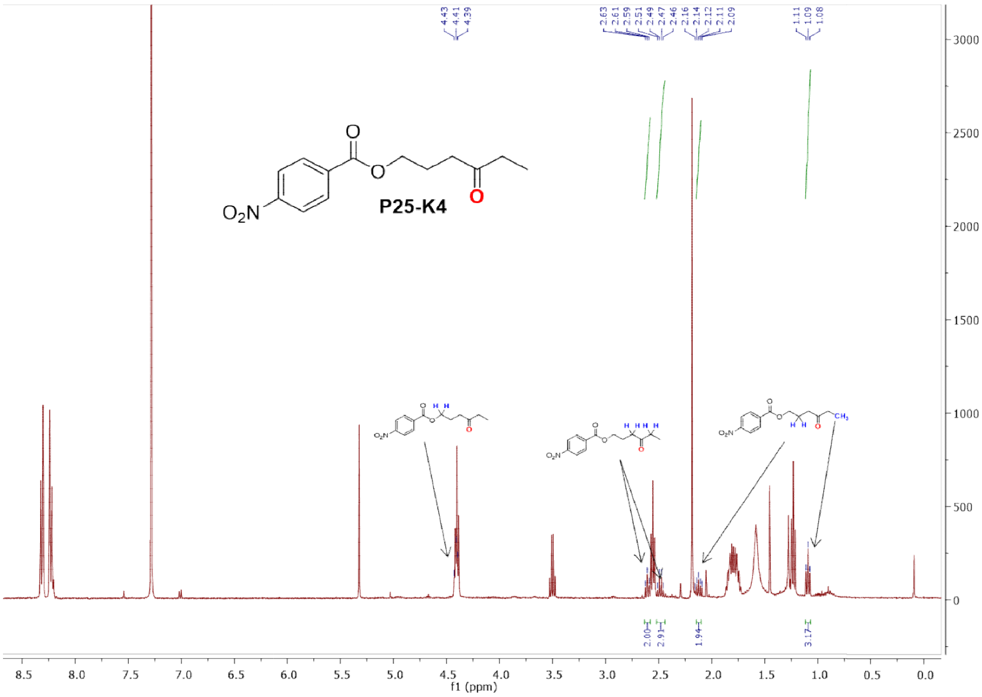

Figure S116. Crude mixture ^{1}H-NMR spectrum (400 MHz, $CDCl_3$) of 1-hexyl 4-nitrobenzoate (**S25**) oxidation in NFTBA: Identification of **P25-K4**.

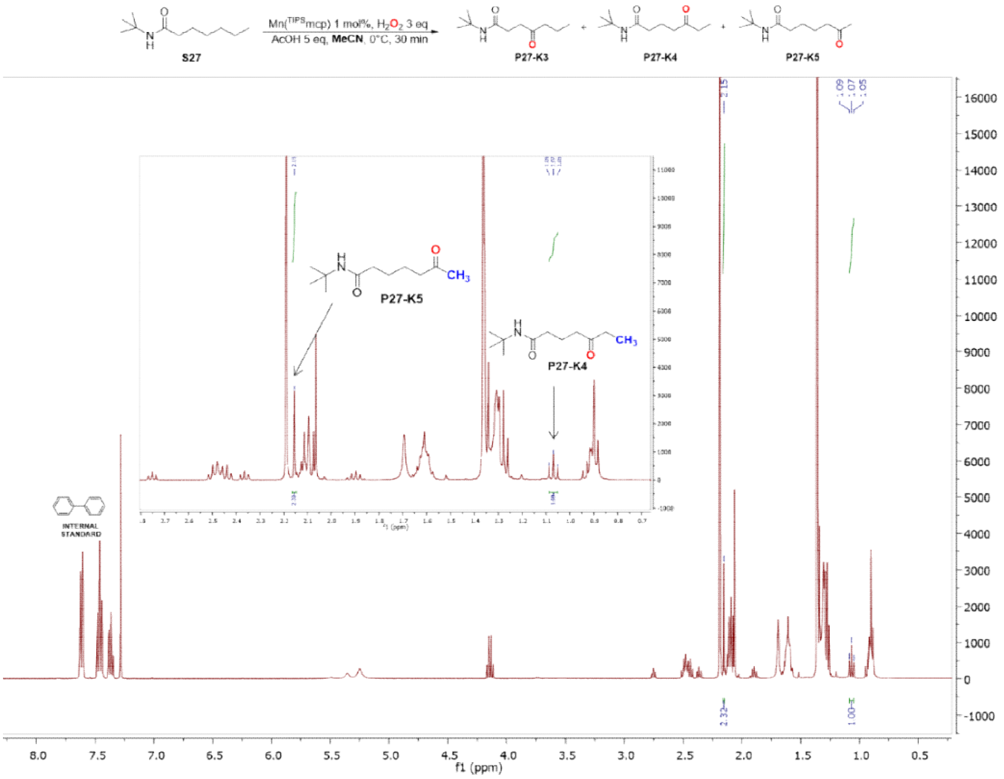

Figure S117. Crude mixture ¹H-NMR spectrum (400 MHz, $CDCl_3$) of *N*-(tert-butyl)heptanamide (**S27**) oxidation in MeCN.

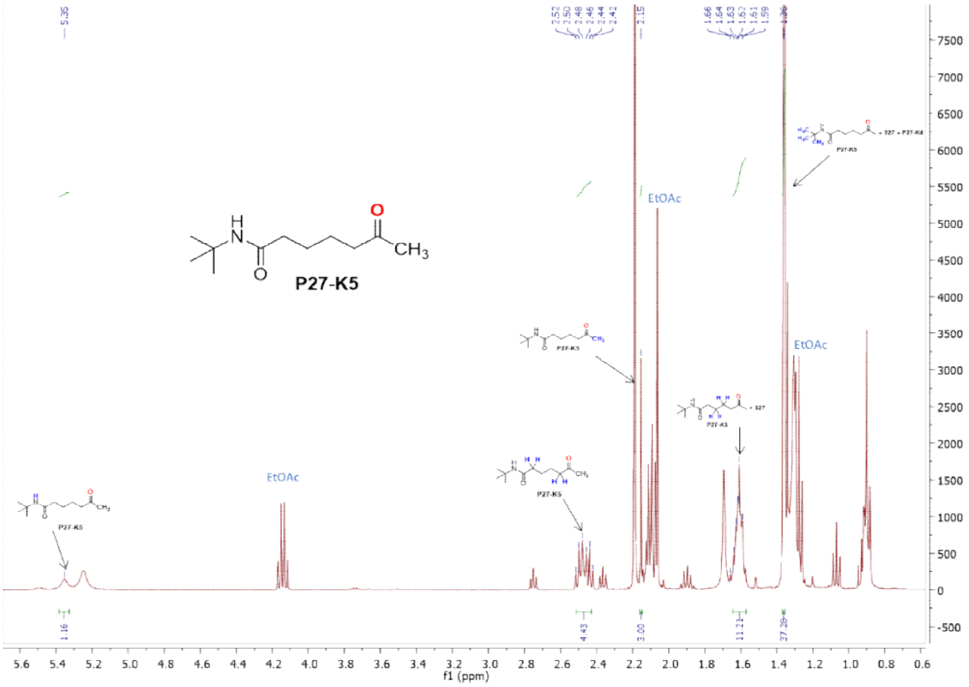

Figure S118. Crude mixture ¹H-NMR spectrum (400 MHz, $CDCl_3$) of *N*-(tert-butyl)heptanamide (**S27**) oxidation in MeCN: Identification of **P27-K5**.

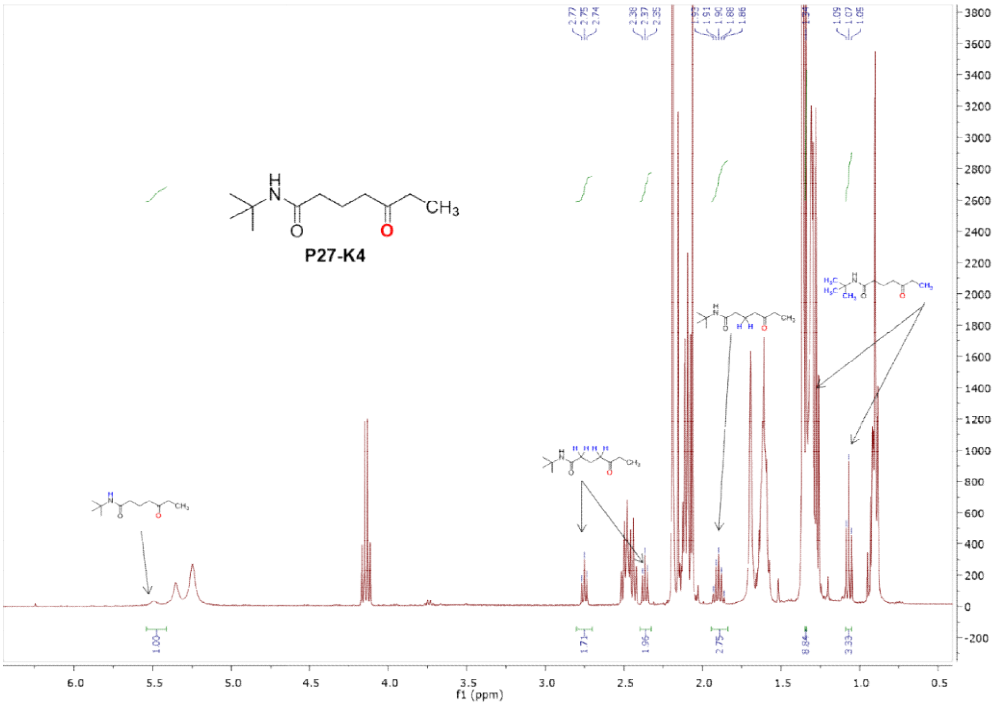

Figure S119. Crude mixture ^{1}H-NMR spectrum (400 MHz, $CDCl_3$) of *N*-(tert-butyl)heptanamide (**S27**) oxidation in MeCN: Identification of **P27-K4**.

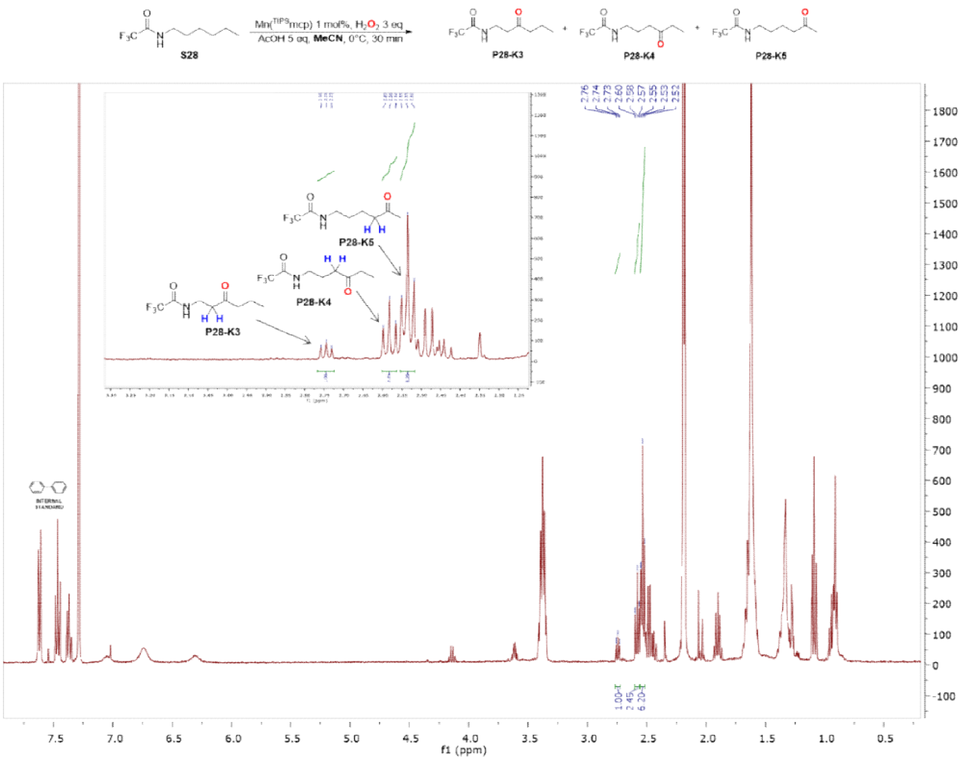

Figure S120. Crude mixture ^{1}H-NMR spectrum (400 MHz, $CDCl_3$) of *N*-hexyl-2,2,2-trifluoro acetamide (**S28**) oxidation in MeCN.

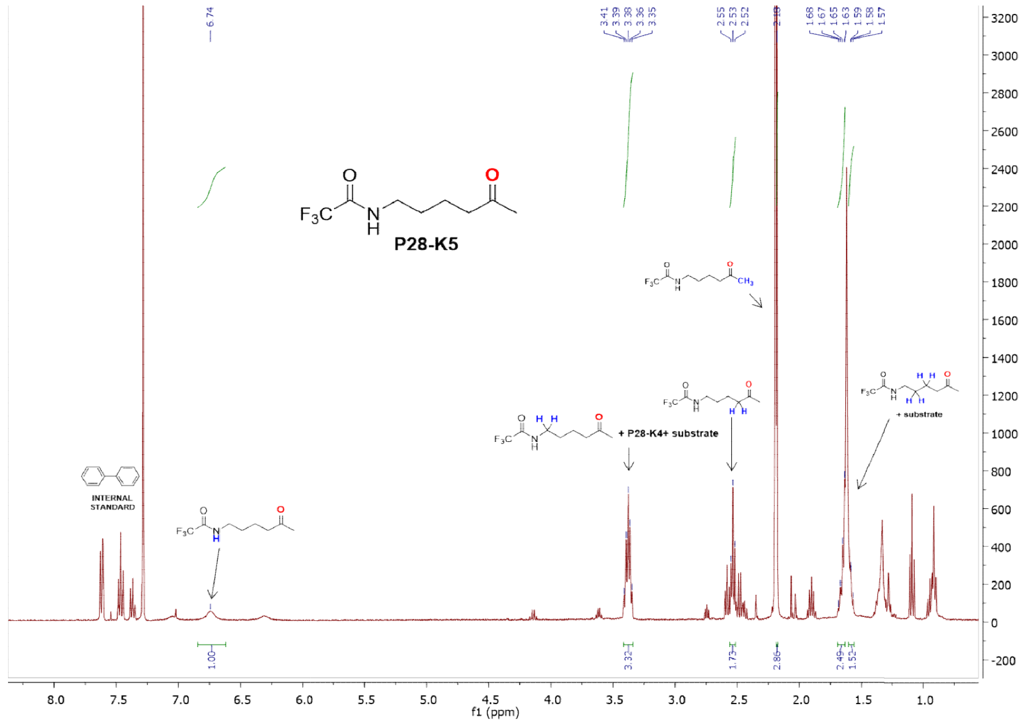

Figure S121. Crude mixture ^{1}H-NMR spectrum (400 MHz, $CDCl_3$) of *N*-hexyl-2,2,2-trifluoro acetamide (**S28**) oxidation in MeCN: Identification of **P28-K5**.

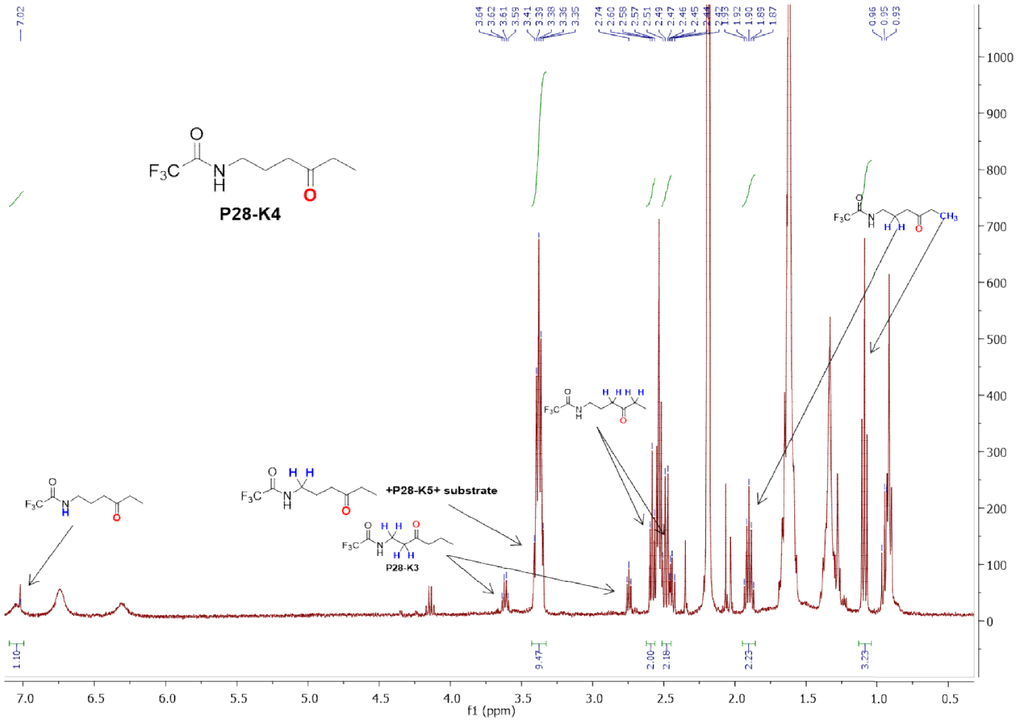

Figure S122. Crude mixture ^{1}H-NMR spectrum (400 MHz, $CDCl_3$) of *N*-hexyl-2,2,2-trifluoro acetamide (**S28**) oxidation in MeCN: Identification of **P28-K4**.

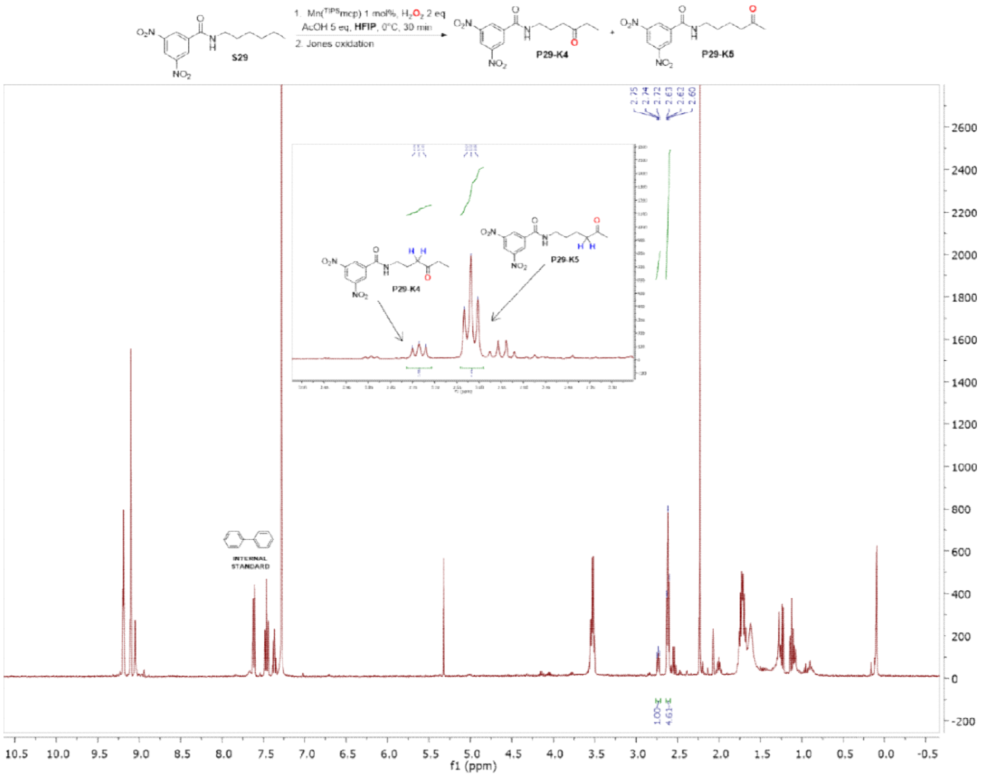

Figure S123. Crude mixture ^{1}H-NMR spectrum (400 MHz, $CDCl_3$) of *N*-hexyl-3,5-dinitrobenzamide (**S29**) oxidation in HFIP.

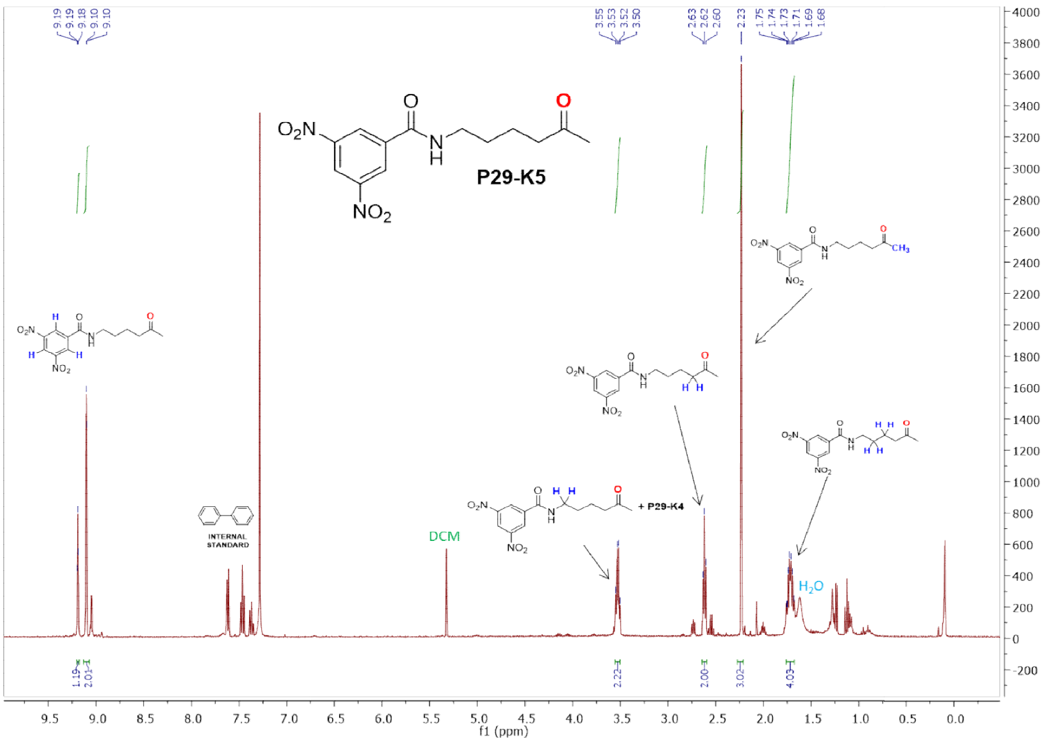

Figure S124. Crude mixture ^{1}H-NMR spectrum (400 MHz, $CDCl_3$) of *N*-hexyl-3,5-dinitrobenzamide (**S29**) oxidation in HFIP: Identification of **P29-K5**.

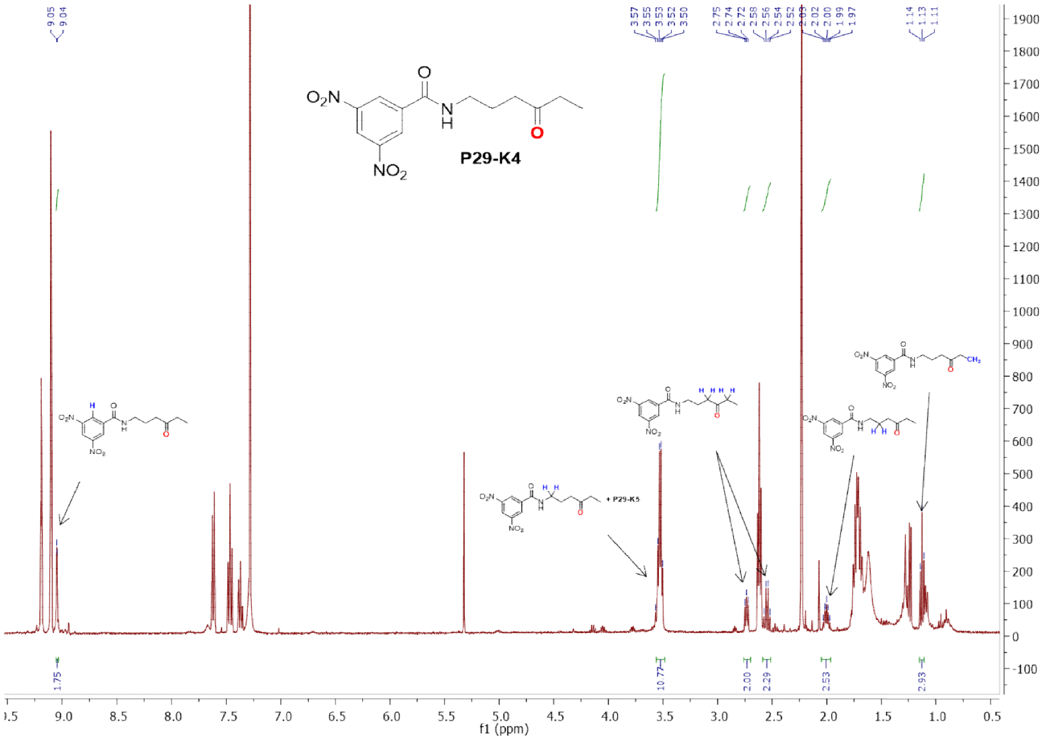

Figure S125. Crude mixture ^{1}H-NMR spectrum (400 MHz, $CDCl_3$) of *N*-hexyl-3,5-dinitrobenzamide (**S29**) oxidation in HFIP: Identification of **P29-K4**.

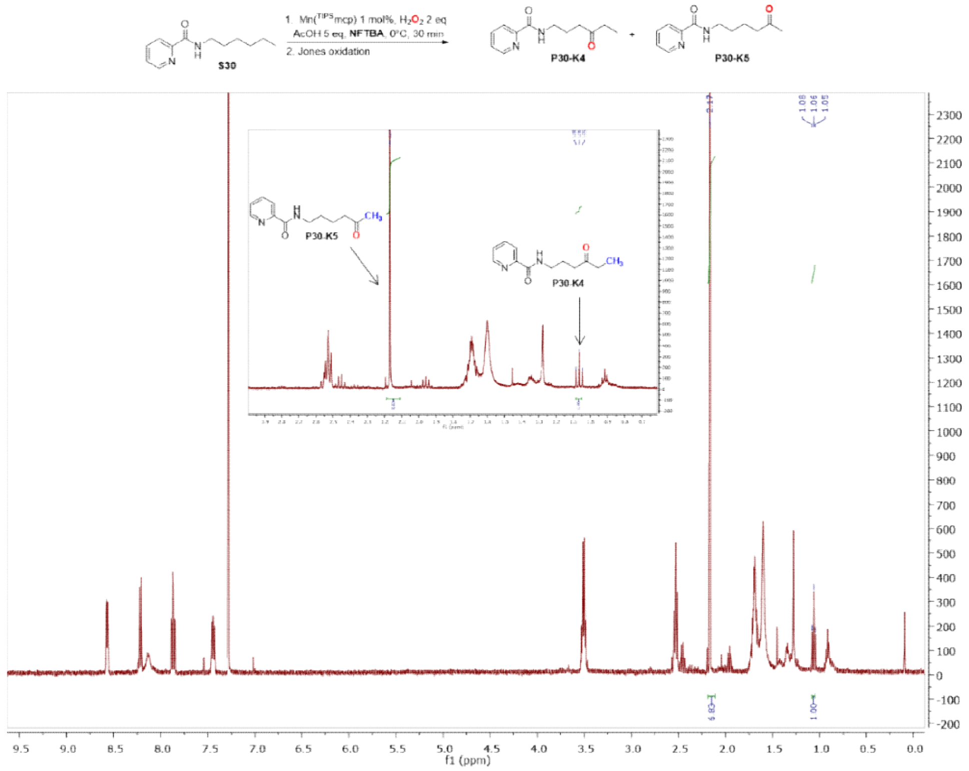

Figure S126. Crude mixture ^{1}H-NMR spectrum (400 MHz, $CDCl_3$) of *N*-hexyl 2-pyridinecarboxamide (**S30**) oxidation in NFTBA.

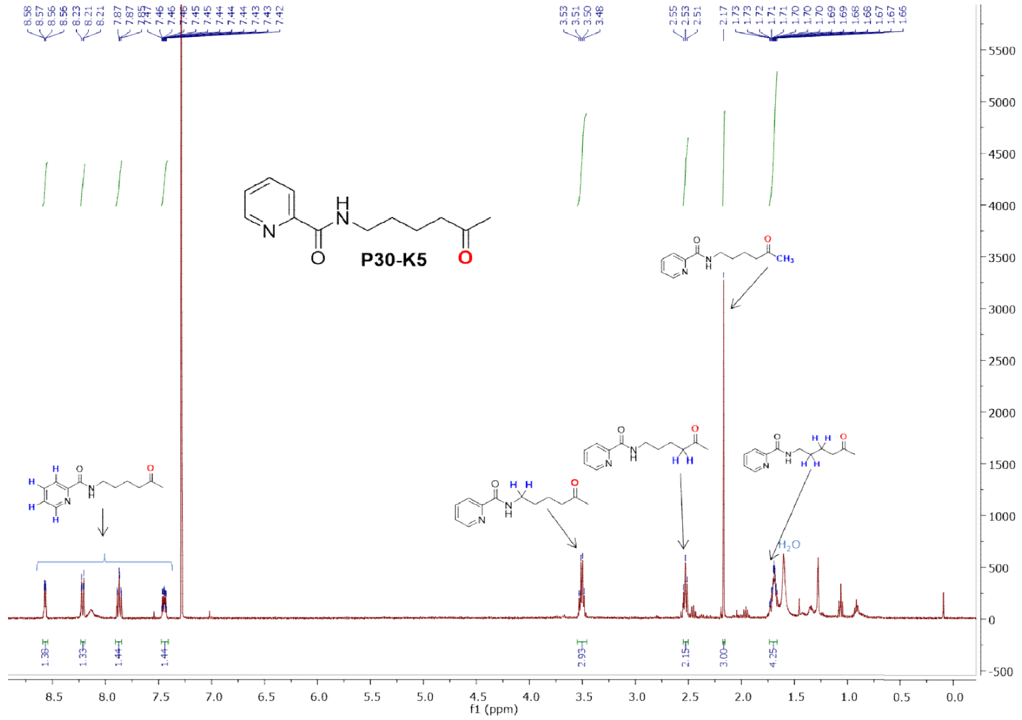

Figure S127. Crude mixture ^{1}H-NMR spectrum (400 MHz, $CDCl_3$) of *N*-hexyl 2-pyridinecarboxamide (**S30**) oxidation in NFTBA: Identification of **P30-K5**.

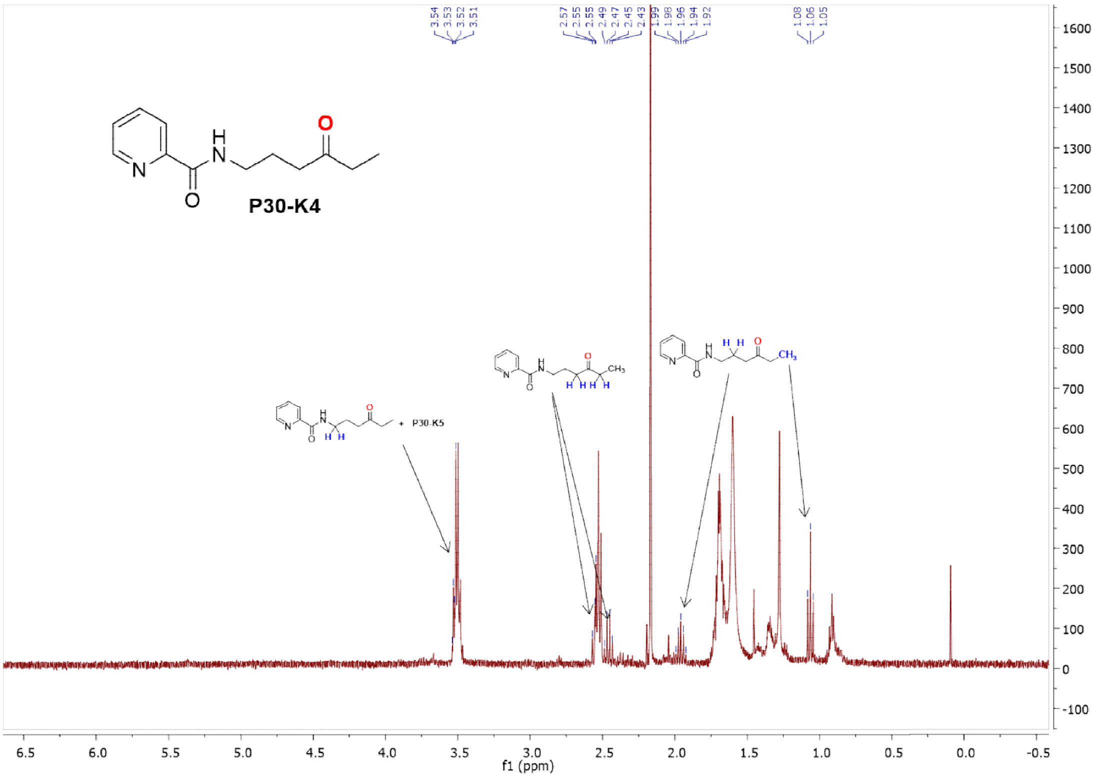

Figure S128. Crude mixture ^{1}H-NMR spectrum (400 MHz, $CDCl_3$) of *N*-hexyl 2-pyridinecarboxamide (**S30**) oxidation in NFTBA: Identification of **P30-K4**.

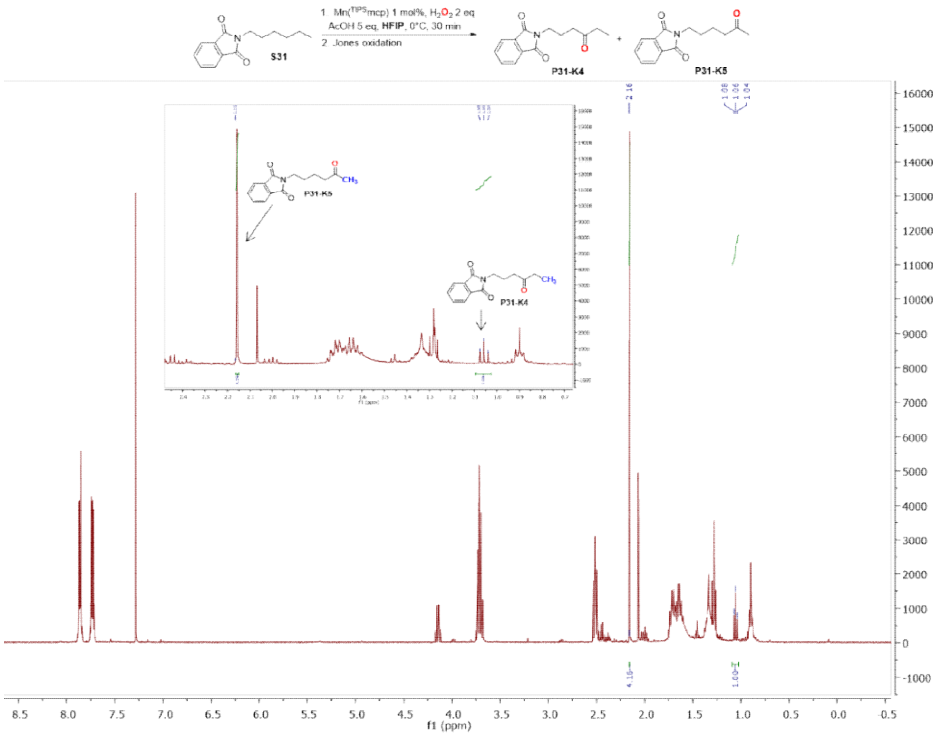

Figure S129. Crude mixture ^{1}H-NMR spectrum (400 MHz, $CDCl_3$) of *N*-hexylphtalimide (**S31**) oxidation in HFIP.

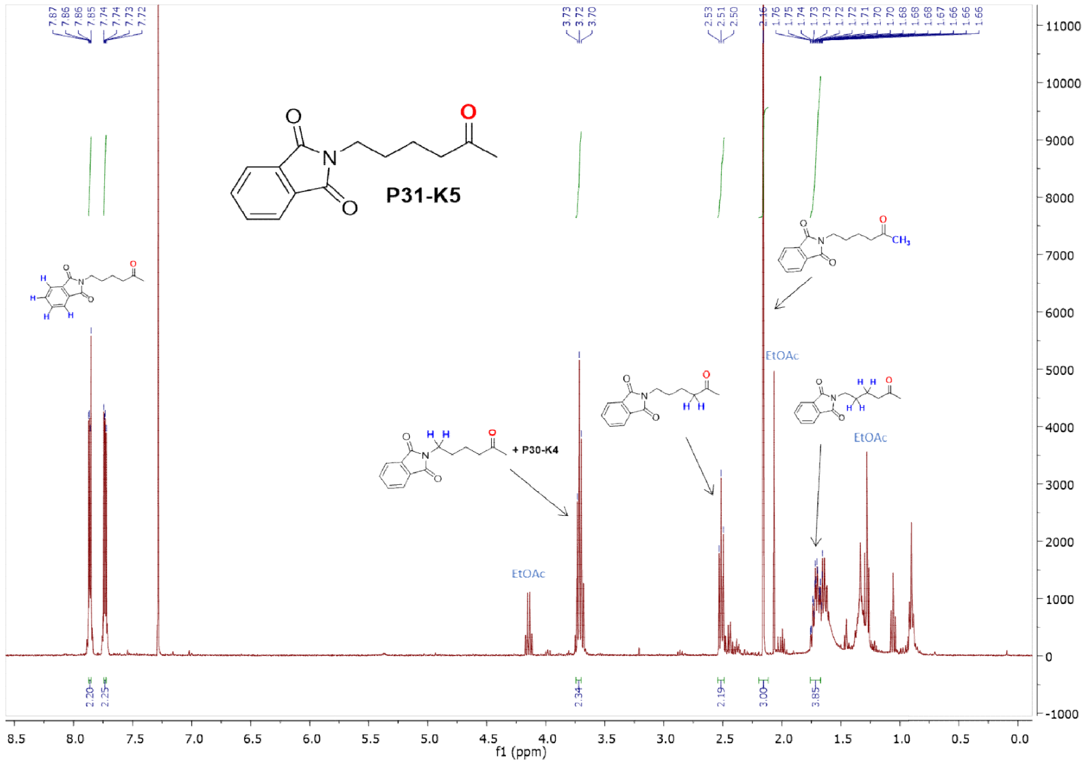

Figure S130. Crude mixture ^{1}H-NMR spectrum (400 MHz, $CDCl_3$) of *N*-hexylphtalimide (**S31**) oxidation in HFIP: Identification of **P31-K5**.

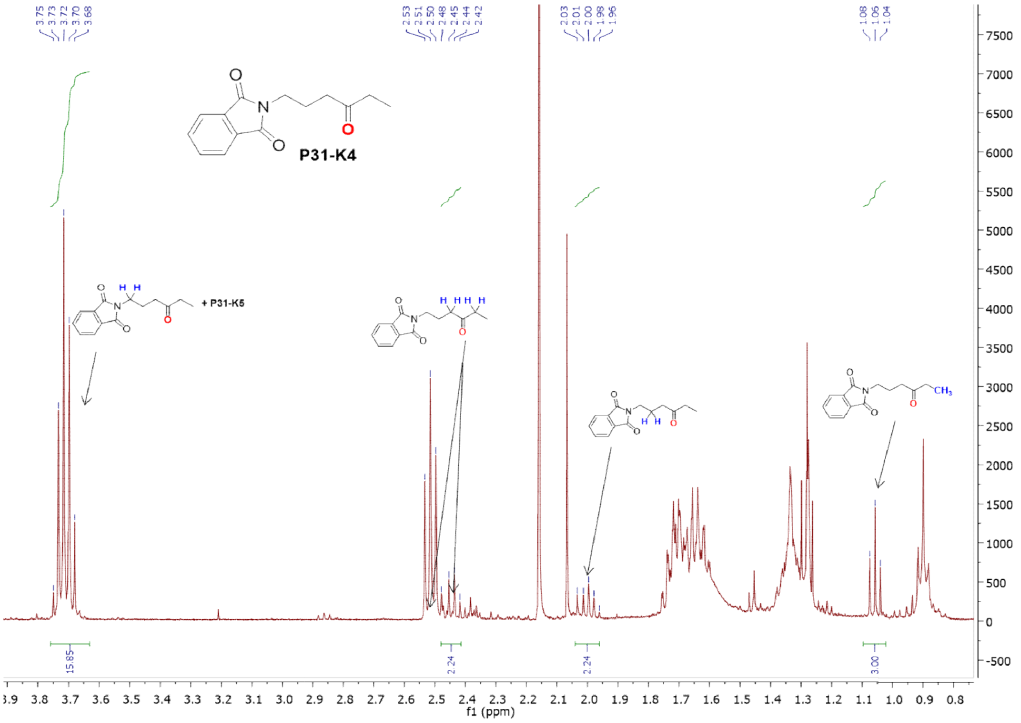

Figure S131. Crude mixture ^{1}H-NMR spectrum (400 MHz, $CDCl_3$) of *N*-hexylphtalimide (**S31**) oxidation in HFIP: Identification of **P31-K4**.

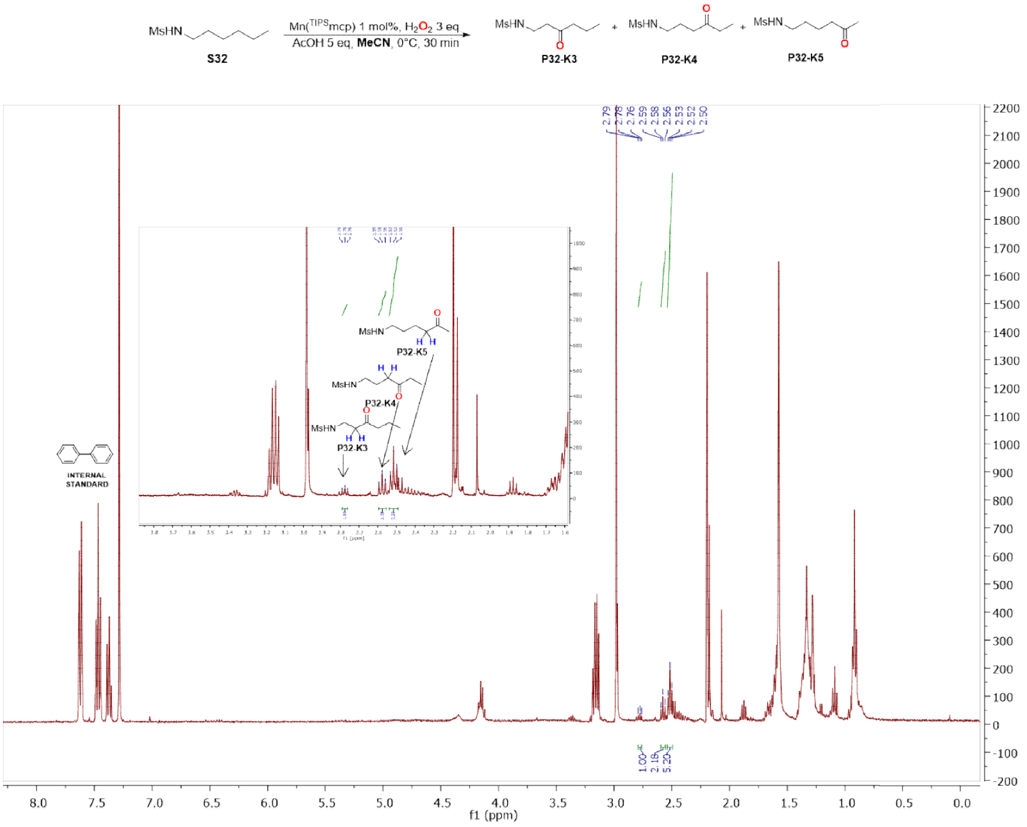

Figure S132. Crude mixture ^{1}H-NMR spectrum (400 MHz, $CDCl_3$) of *N*-hexylmethanesulfonamide (**S32**) oxidation in MeCN.

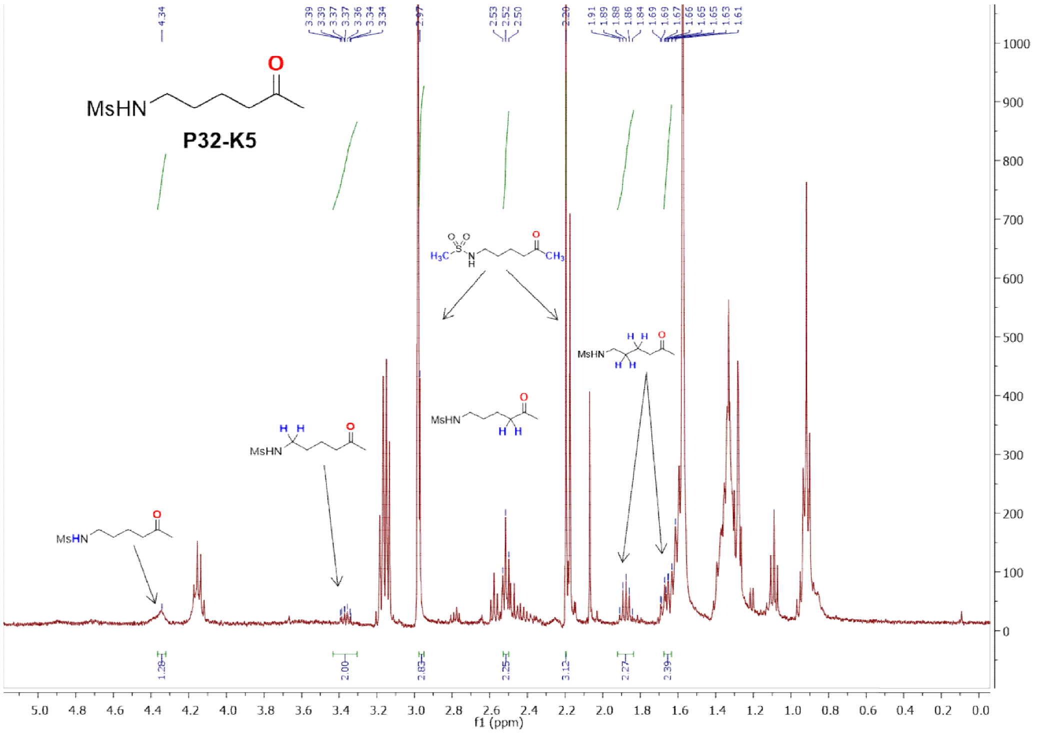

Figure S133. Crude mixture ^{1}H-NMR spectrum (400 MHz, $CDCl_3$) of *N*-hexylmethanesulfonamide (**S32**) oxidation in MeCN: Identification of **P32-K5**.

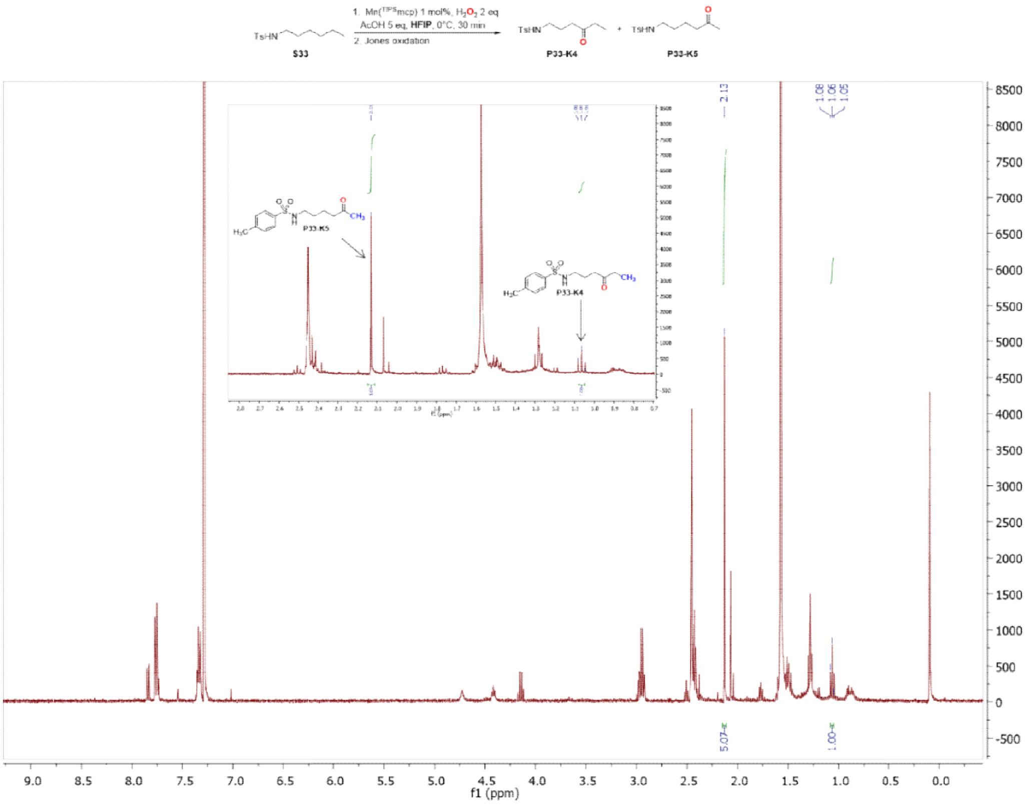

Figure S134. Crude mixture ^{1}H-NMR spectrum (400 MHz, $CDCl_3$) of *N*-hexyl-4-methylbenzenesulfonamide (**S33**) oxidation in HFIP.

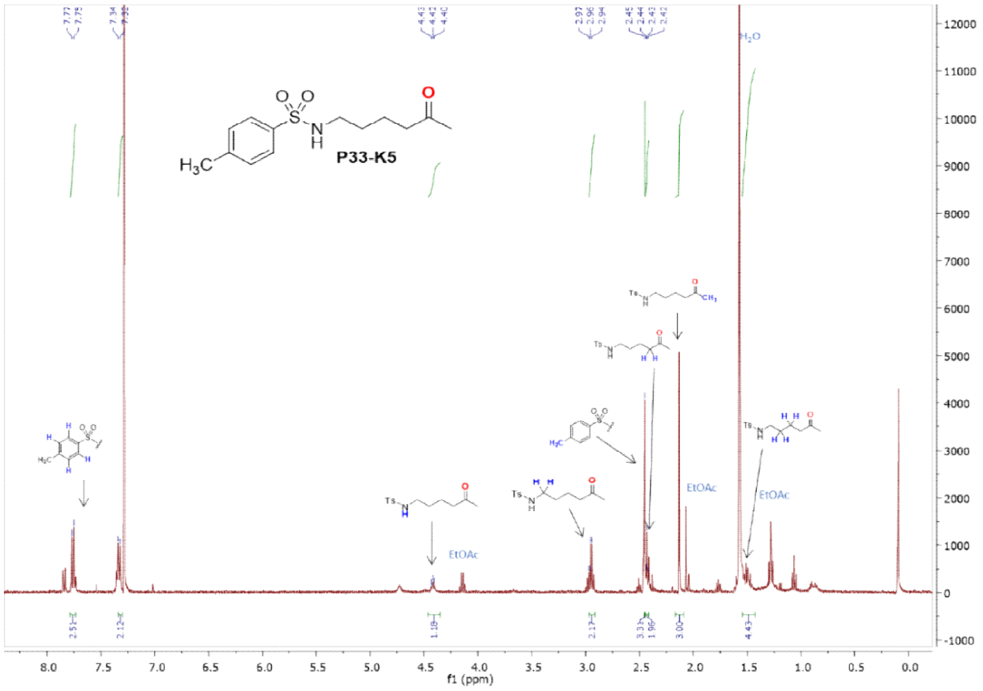

Figure S135. Crude mixture ^{1}H-NMR spectrum (400 MHz, $CDCl_3$) of *N*-hexyl-4-methylbenzenesulfonamide (**S33**) oxidation in HFIP: Identification of **P33.K5**.

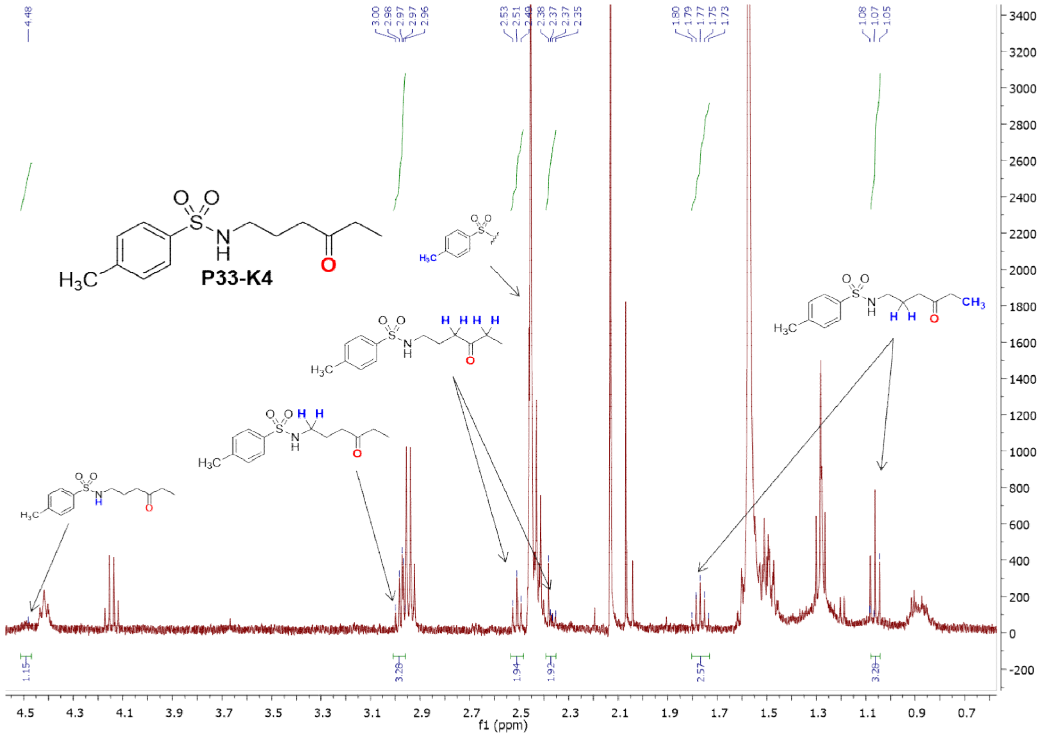

Figure S136. Crude mixture ^{1}H-NMR spectrum (400 MHz, $CDCl_3$) of *N*-hexyl-4-methylbenzenesulfonamide (**S33**) oxidation in HFIP: Identification of **P33.K4**.

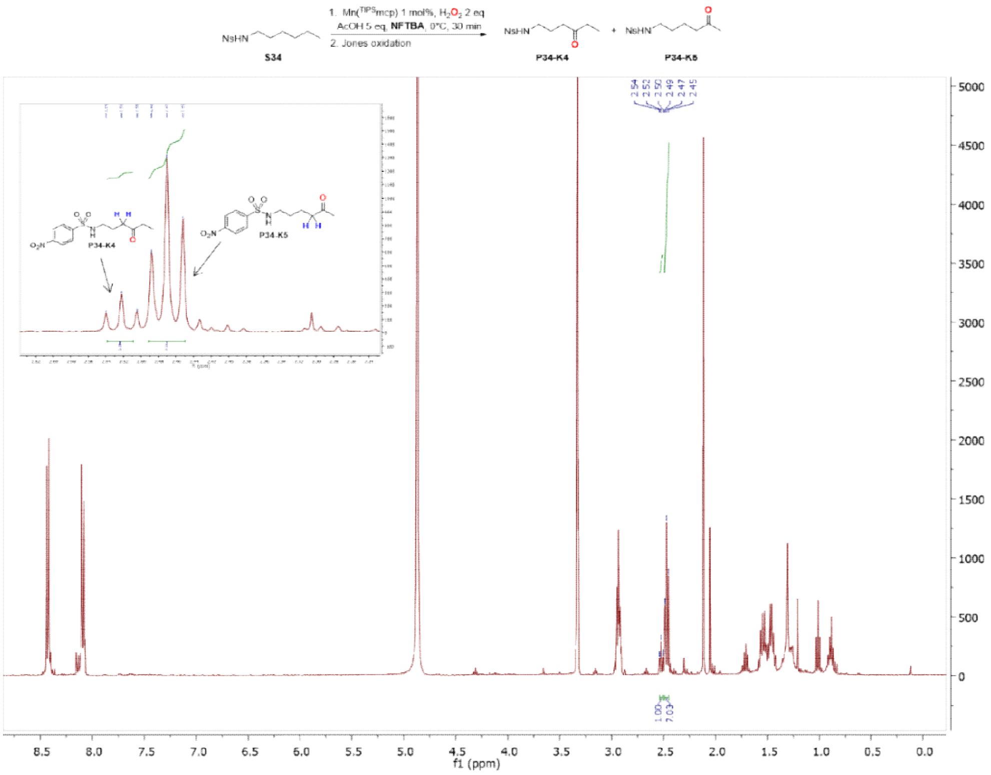

Figure S137. Crude mixture ^{1}H-NMR spectrum (400 MHz, CD_3OD) of *N*-hexyl-4-nitrobenzenesulfonamide (**S34**) oxidation in NFTBA.

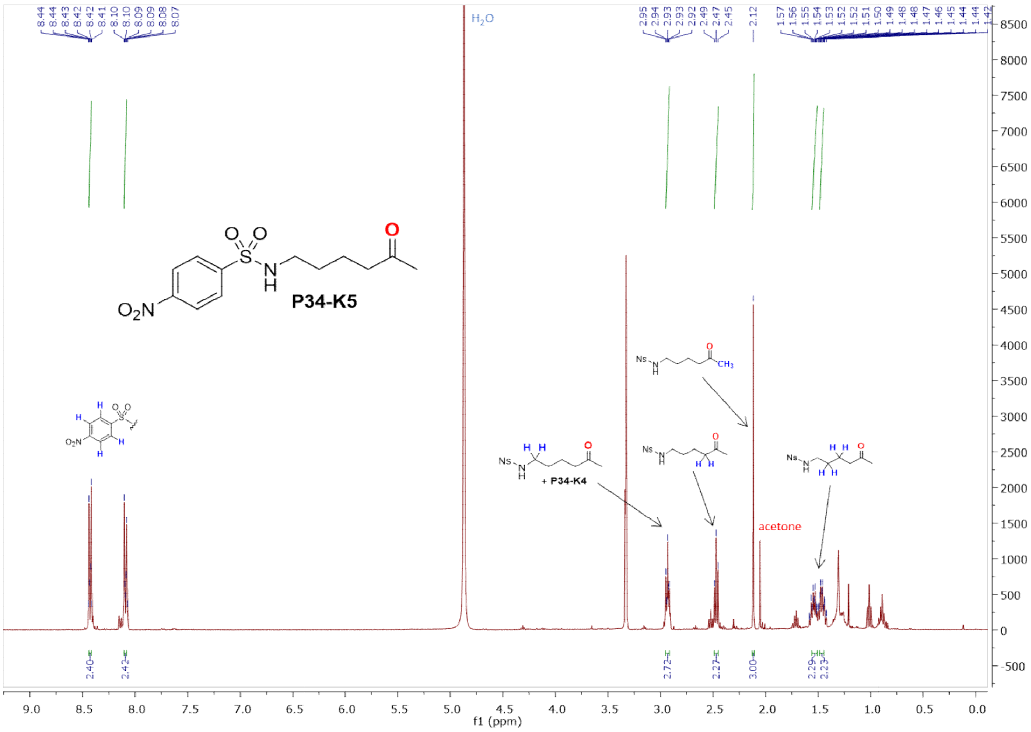

Figure S138. Crude mixture ^{1}H-NMR spectrum (400 MHz, CD_3OD) of *N*-hexyl-4-nitrobenzenesulfonamide (**S34**) oxidation in NFTBA: Identfication of **P34-K5**.

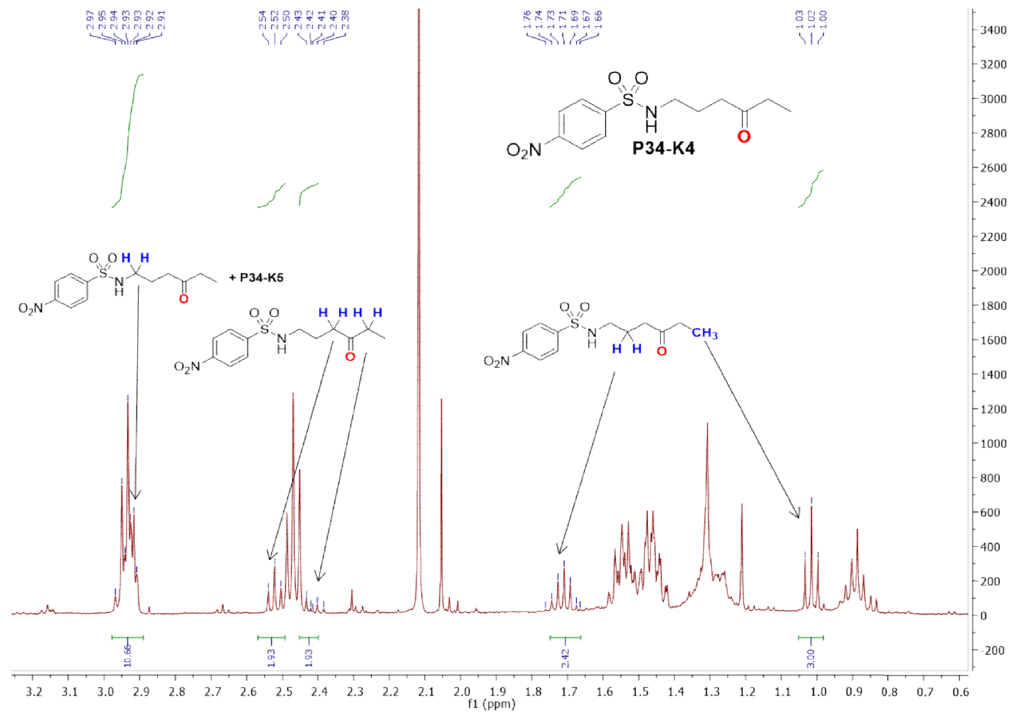

Figure S139. Crude mixture ^{1}H-NMR spectrum (400 MHz, CD_3OD) of *N*-hexyl-4-nitrobenzenesulfonamide (**S34**) oxidation in NFTBA: Identfication of **P34-K4**.

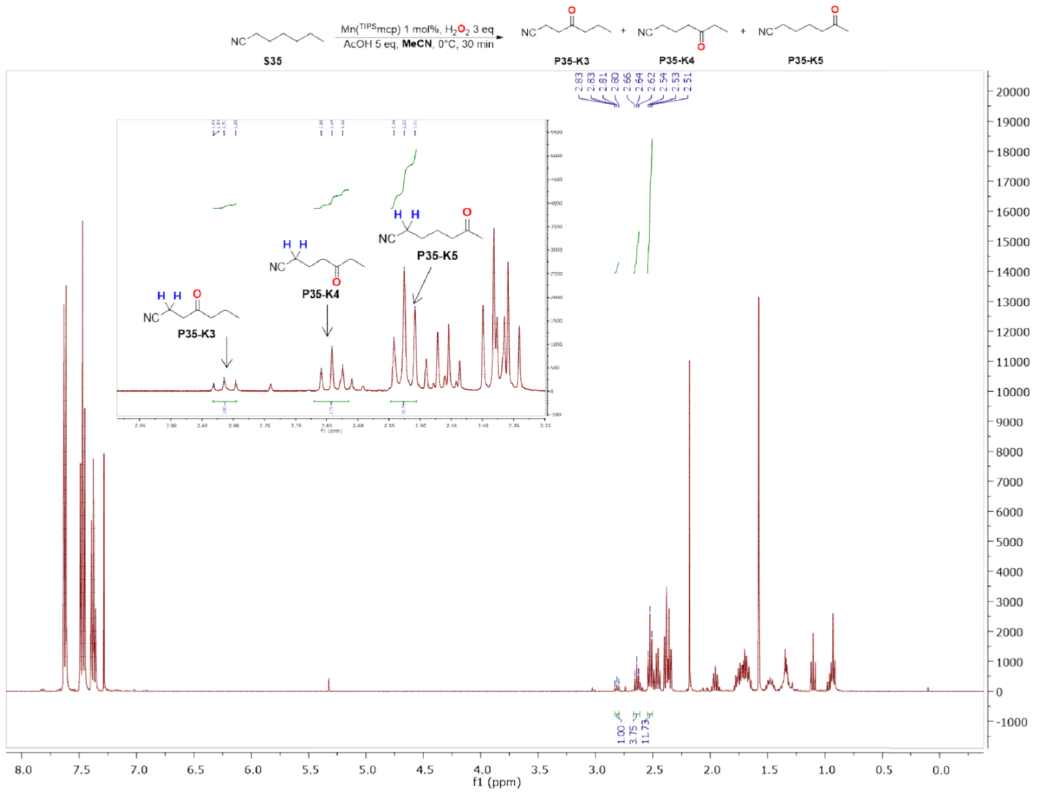

Figure S140. Crude mixture ^{1}H-NMR spectrum (400 MHz, $CDCl_3$) of heptanenitrile (**S35**) oxidation in MeCN.

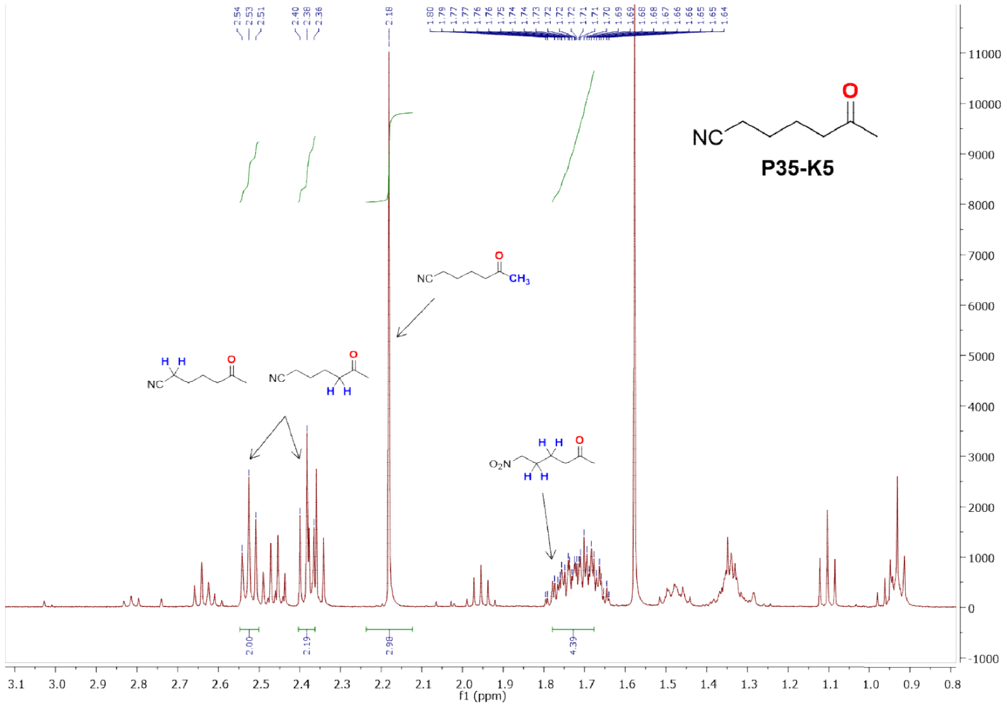

Figure S141. Crude mixture ¹H-NMR spectrum (400 MHz, $CDCl_3$) of 1-heptanenitrile (**S35**) oxidation in MeCN: Identification of **P35-K5**.

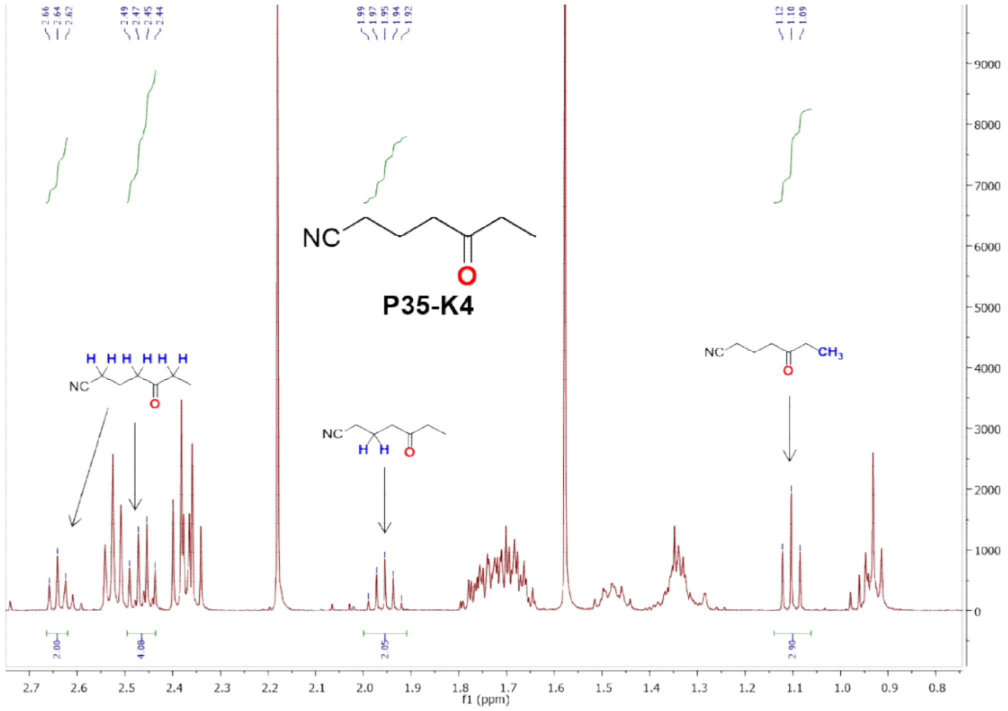

Figure S142. Crude mixture ¹H-NMR spectrum (400 MHz, $CDCl_3$) of 1-heptanenitrile (**S35**) oxidation in MeCN: Identification of **P35-K4**.

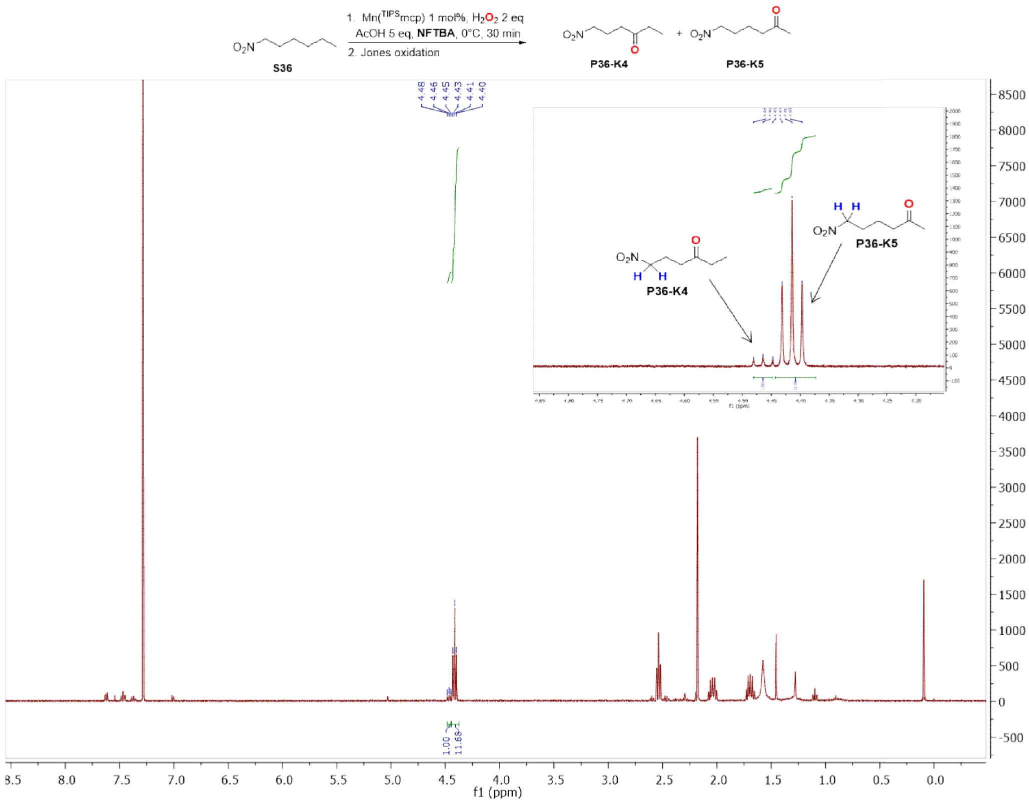

Figure S143. Crude mixture ^{1}H-NMR spectrum (400 MHz, $CDCl_3$) of 1-nitrohexane (**S36**) oxidation in NFTBA.

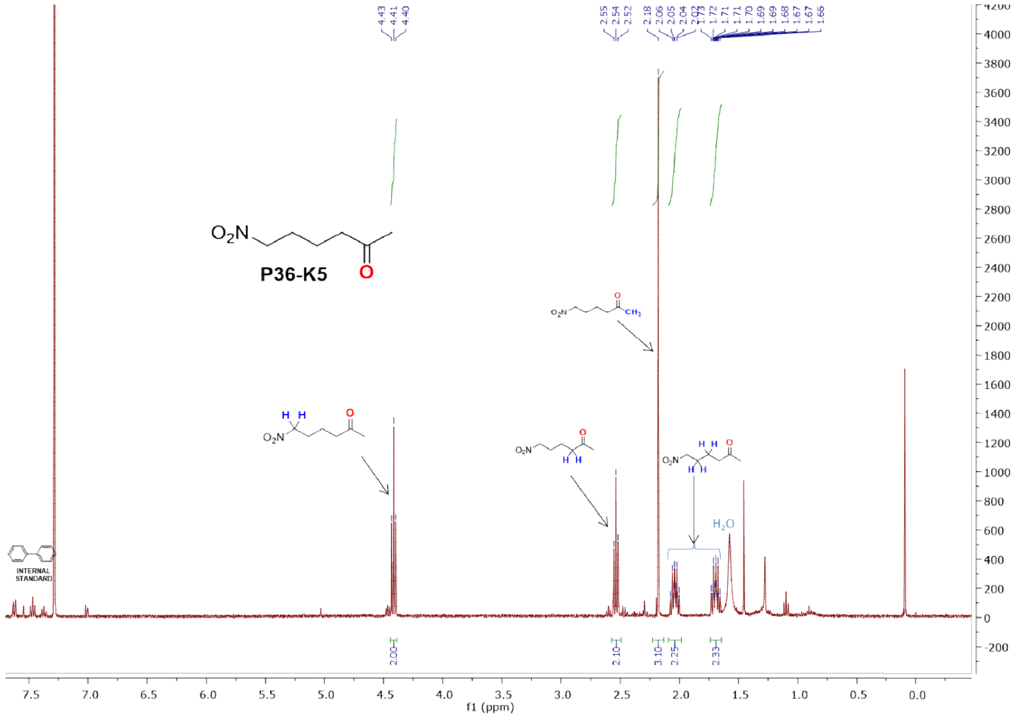

Figure S144. Crude mixture ^{1}H-NMR spectrum (400 MHz, $CDCl_3$) of 1-nitrohexane (**S36**) oxidation in NFTBA: Identification of **P36-K5**.

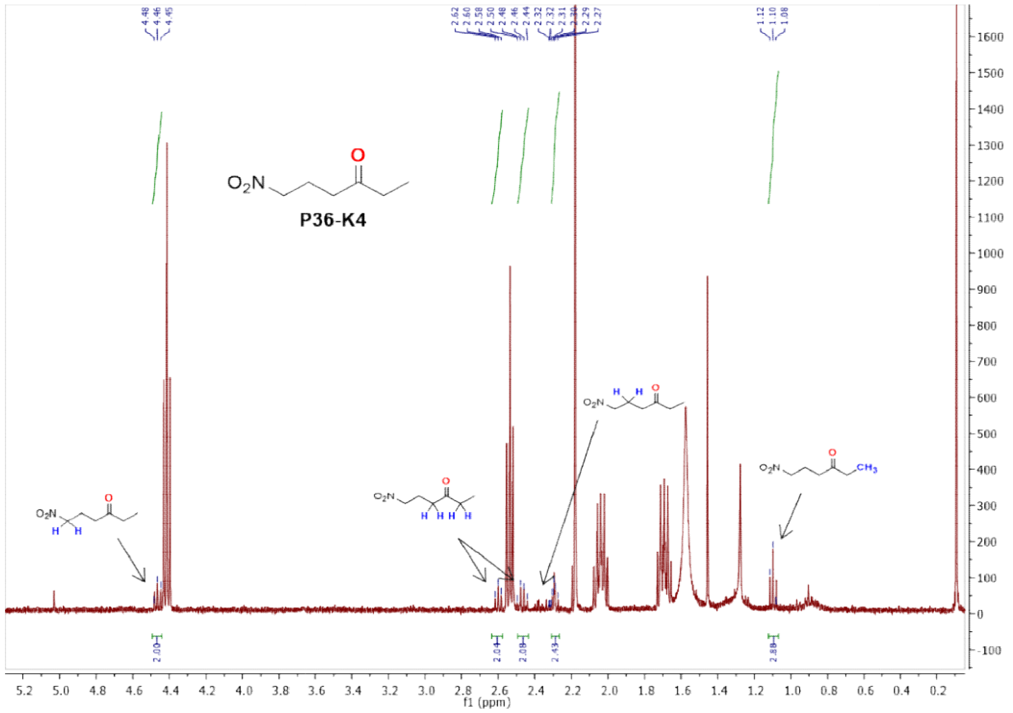

Figure S145. Crude mixture ^{1}H-NMR spectrum (400 MHz, $CDCl_3$) of 1-nitrohexane (**S36**) oxidation in NFTBA: Identification of **P36-K4**.

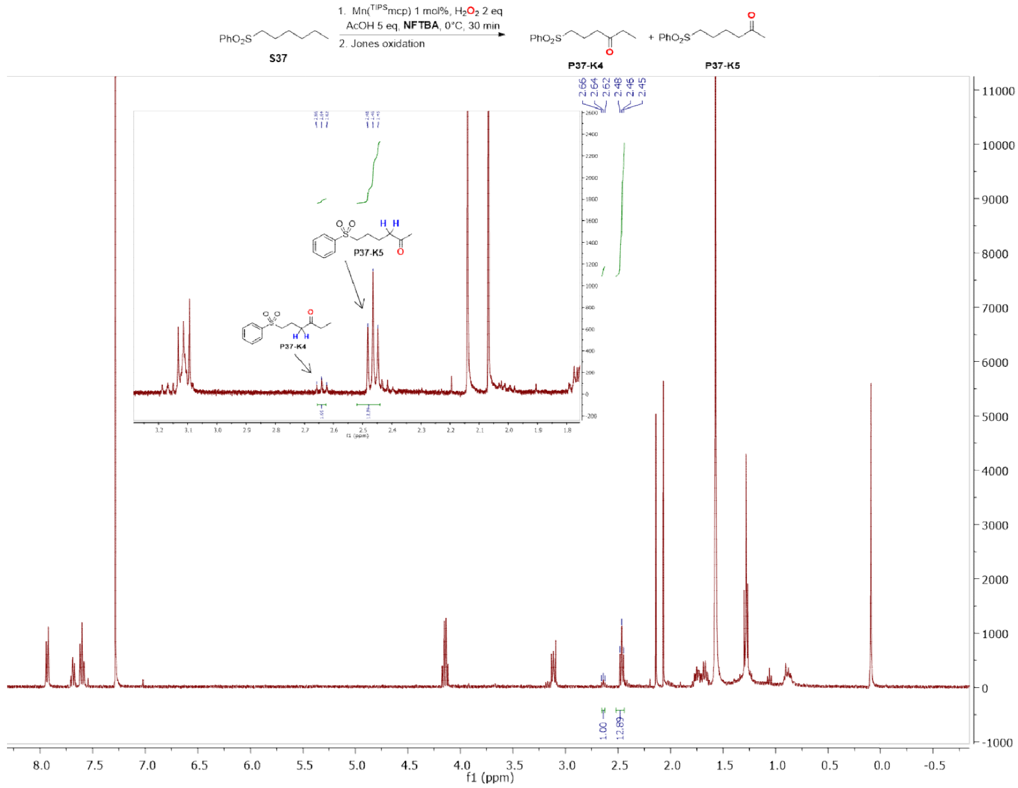

Figure S146. Crude mixture ^{1}H-NMR spectrum (400 MHz, $CDCl_3$) of (hexylsulfonyl)benzene (**S37**) oxidation in NFTBA.

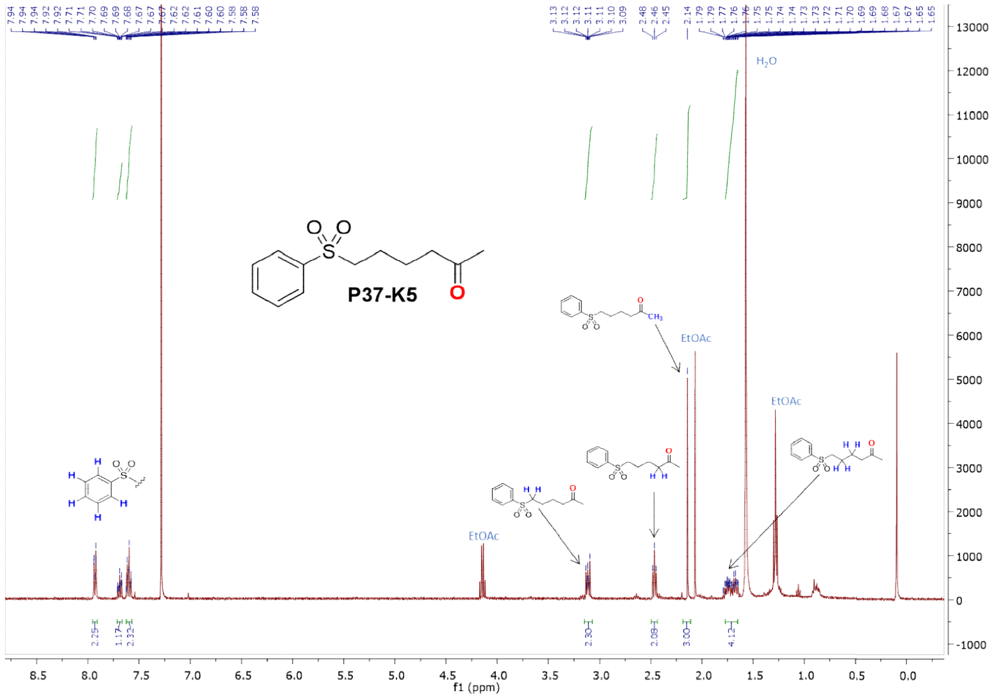

Figure S147. Crude mixture ^{1}H-NMR spectrum (400 MHz, $CDCl_3$) of (hexylsulfonyl)benzene (**S37**) oxidation in NFTBA: Identification of **P37-K5**.

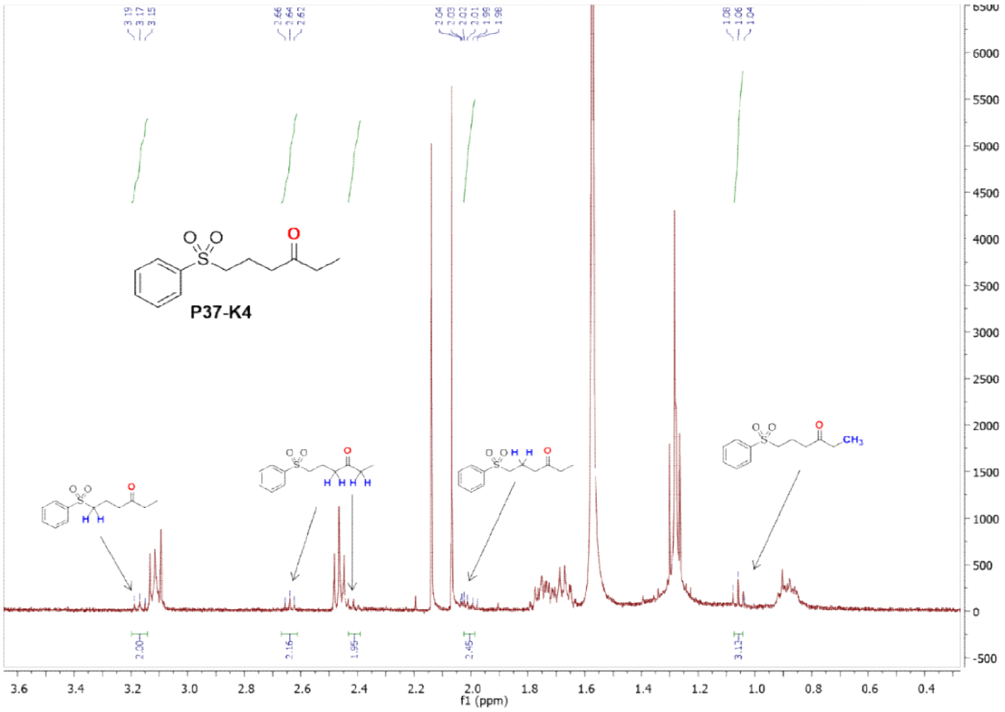

Figure S148. Crude mixture ^{1}H-NMR spectrum (400 MHz, $CDCl_3$) of (hexylsulfonyl)benzene (**S37**) oxidation in NFTBA: Identification of **P37-K4**.

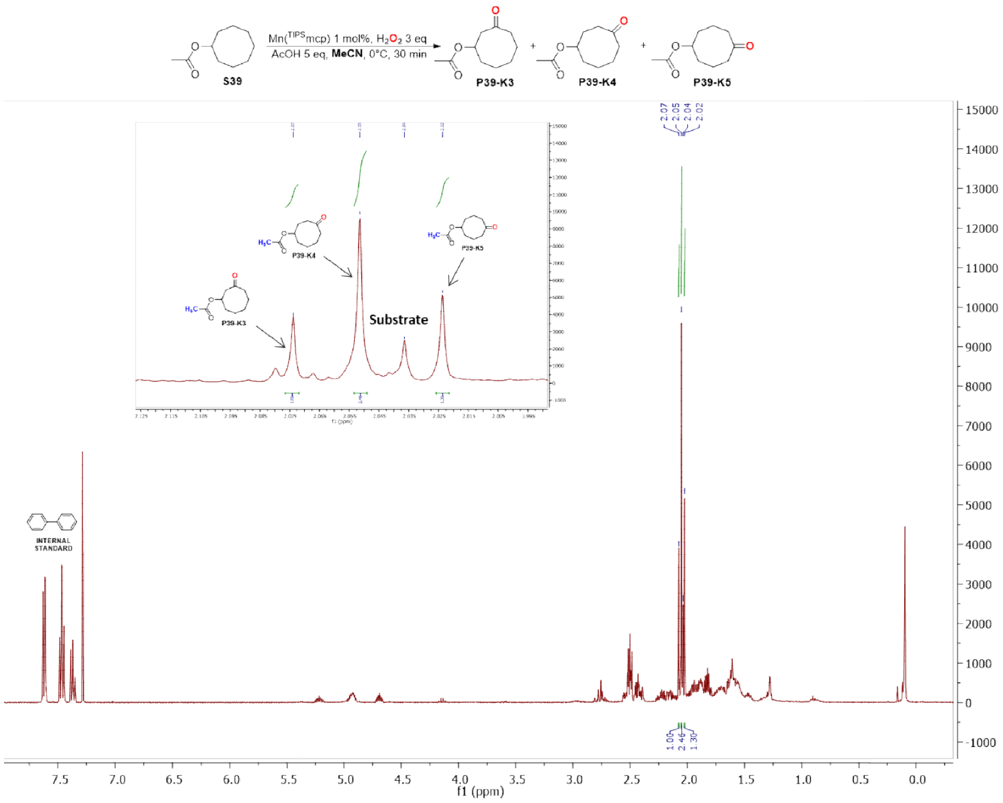

Figure S149. Crude mixture ^{1}H-NMR spectrum (400 MHz, $CDCl_3$) of cyclooctyl acetate (**S39**) oxidation in MeCN.

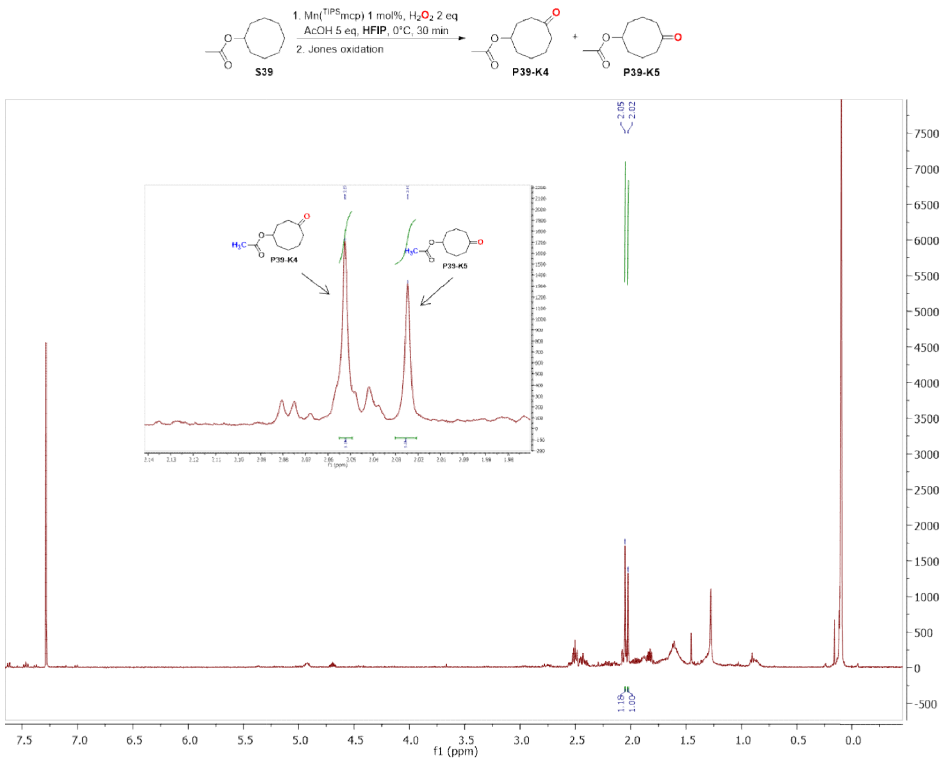

Figure S150. Crude mixture ^{1}H-NMR spectrum (400 MHz, $CDCl_3$) of cyclooctyl acetate (**S39**) oxidation in HFIP.

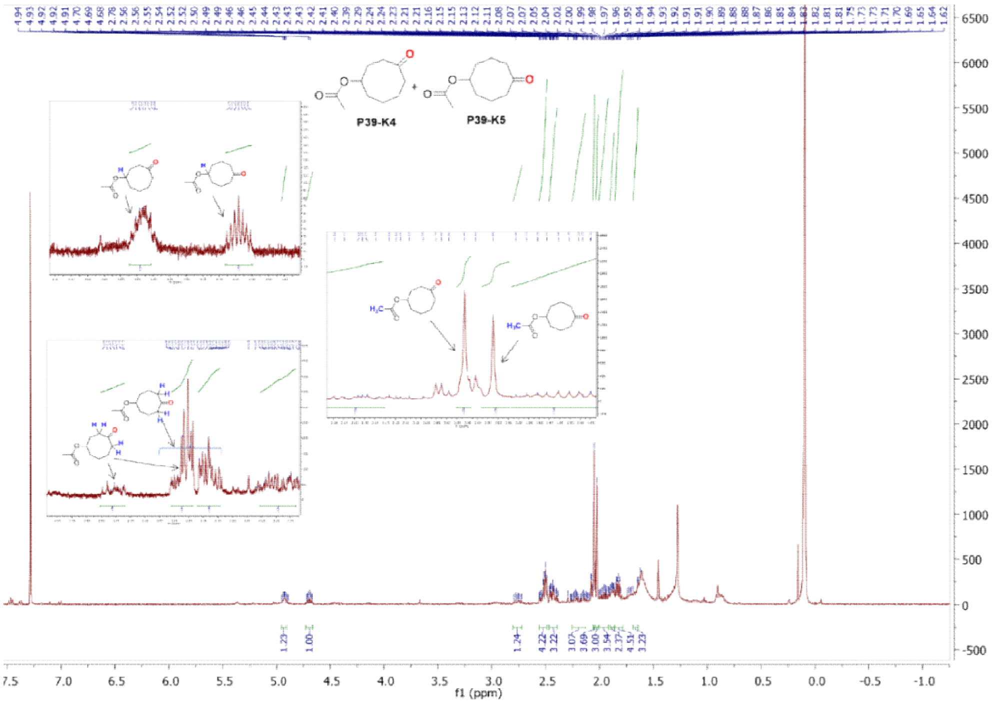

Figure S151. Crude mixture ^{1}H-NMR spectrum (400 MHz, $CDCl_3$) of cyclooctyl acetate (**S39**) oxidation in HFIP: Identification of **P39-K4** and **P39-K5**.

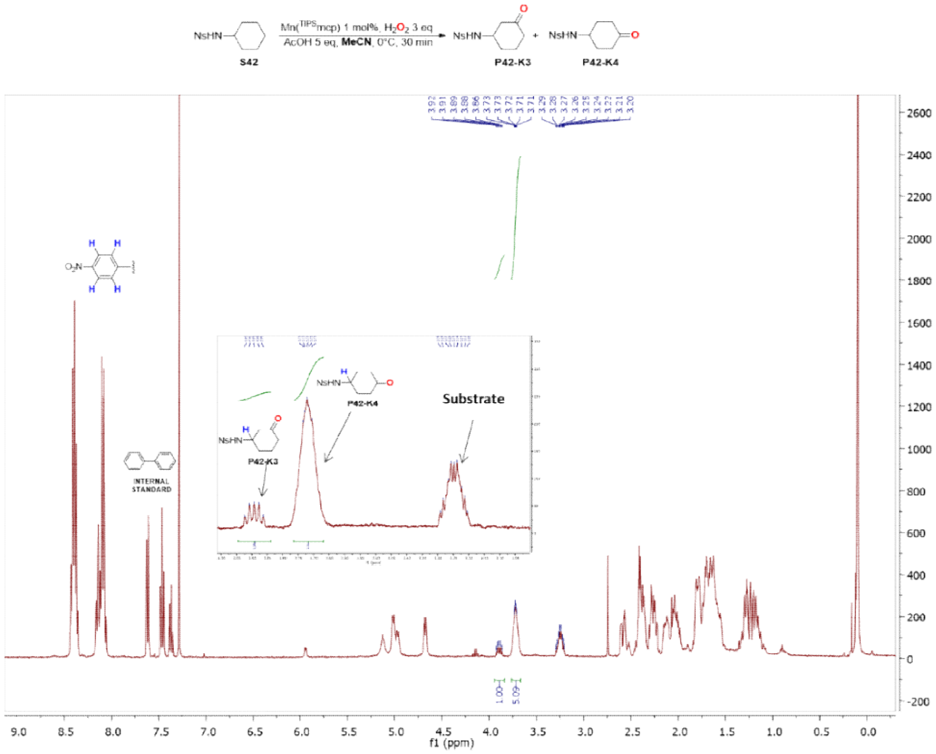

Figure S152. Crude mixture ^{1}H-NMR spectrum (400 MHz, $CDCl_3$) of *N*-cyclohexyl-4-nitrobenzenesulfonamide (**S42**) oxidation in MeCN.

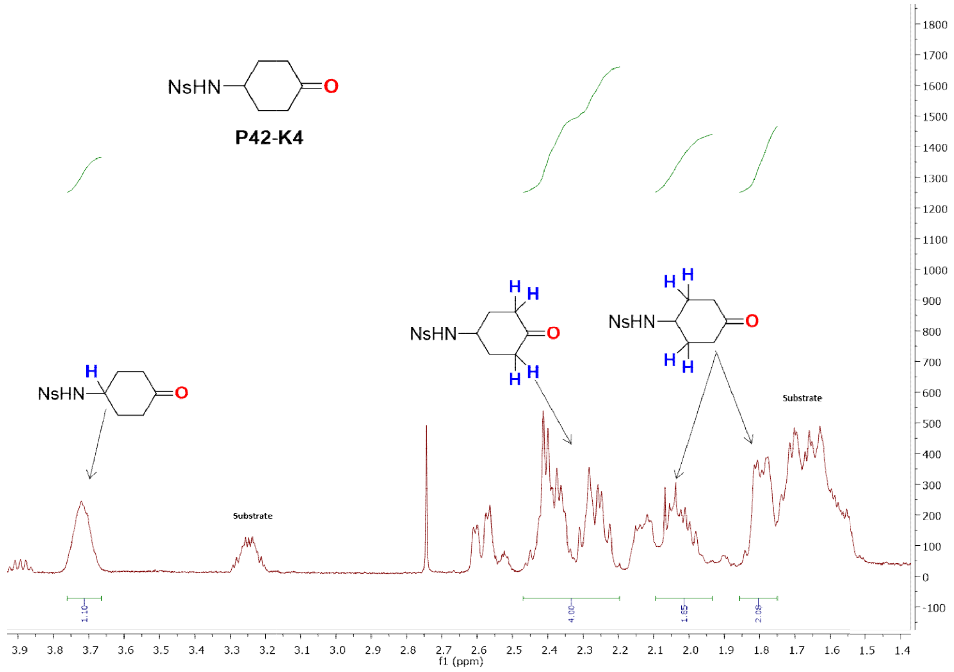

Figure S153. Crude mixture ^{1}H-NMR spectrum (400 MHz, $CDCl_3$) of *N*-cyclohexyl-4-nitrobenzenesulfonamide (**S42**) oxidation in MeCN: Identification of **P42-K4**.

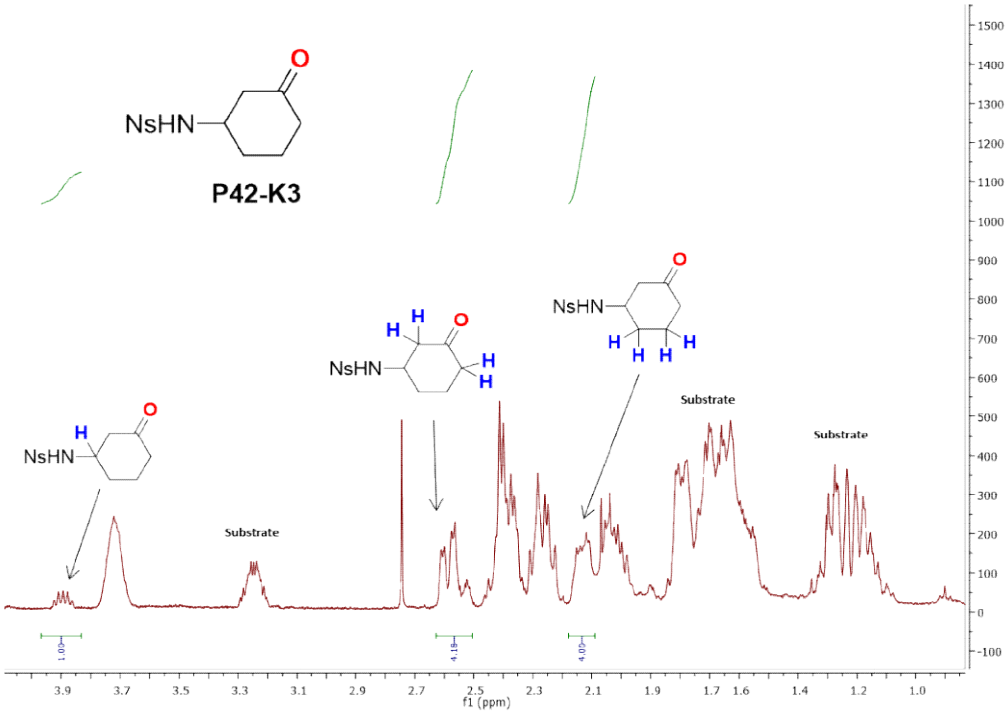

Figure S154. Crude mixture ^{1}H-NMR spectrum (400 MHz, $CDCl_3$) of *N*-cyclohexyl-4-nitrobenzenesulfonamide (**S42**) oxidation in MeCN: Identification of **P42-K3**.

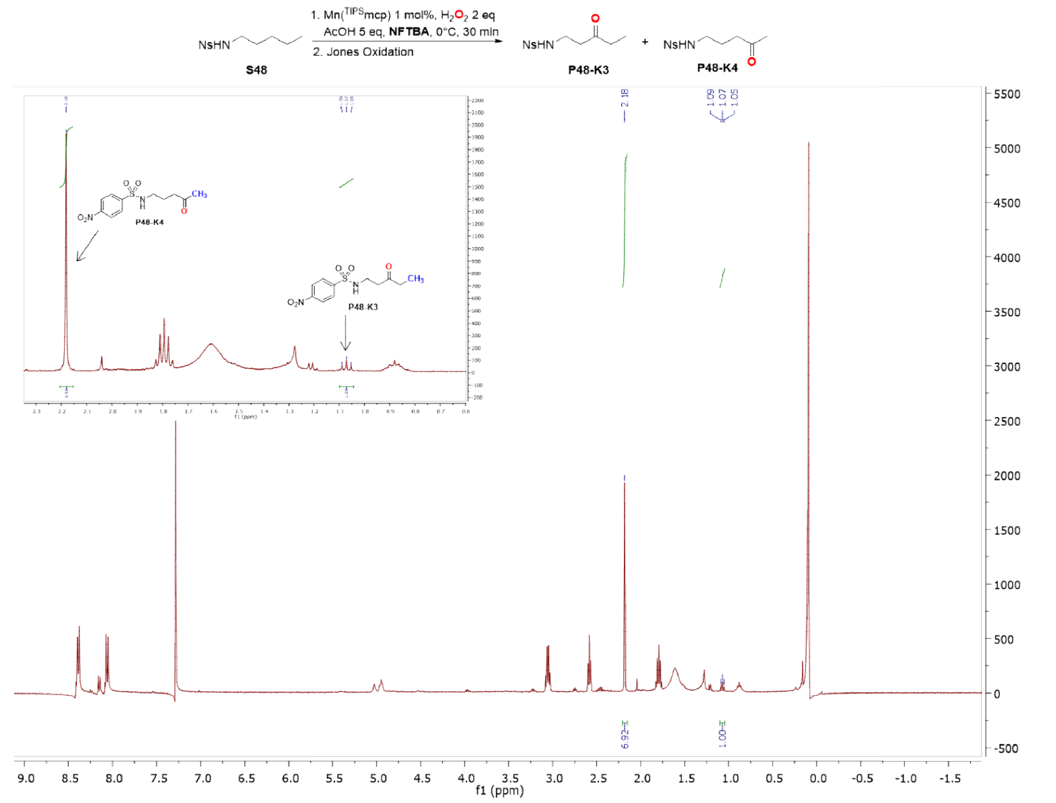

Figure S155. Crude mixture ^{1}H-NMR spectrum (400 MHz, $CDCl_3$) of 4-nitro-*N*-pentylbenzenesulfonamide (**S48**) oxidation in NFTBA.

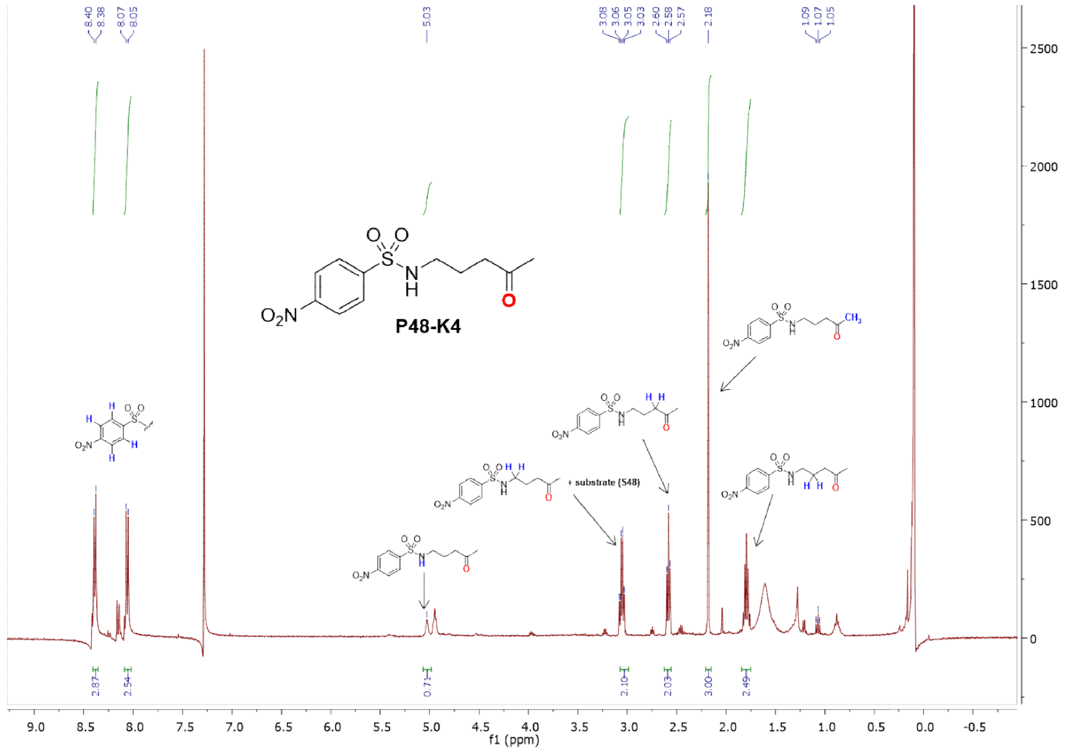

Figure S156. Crude mixture ^{1}H-NMR spectrum (400 MHz, $CDCl_3$) of 4-nitro-*N*-pentylbenzenesulfonamide (**S48**) oxidation in NFTBA: Identification of **P48-K4**.

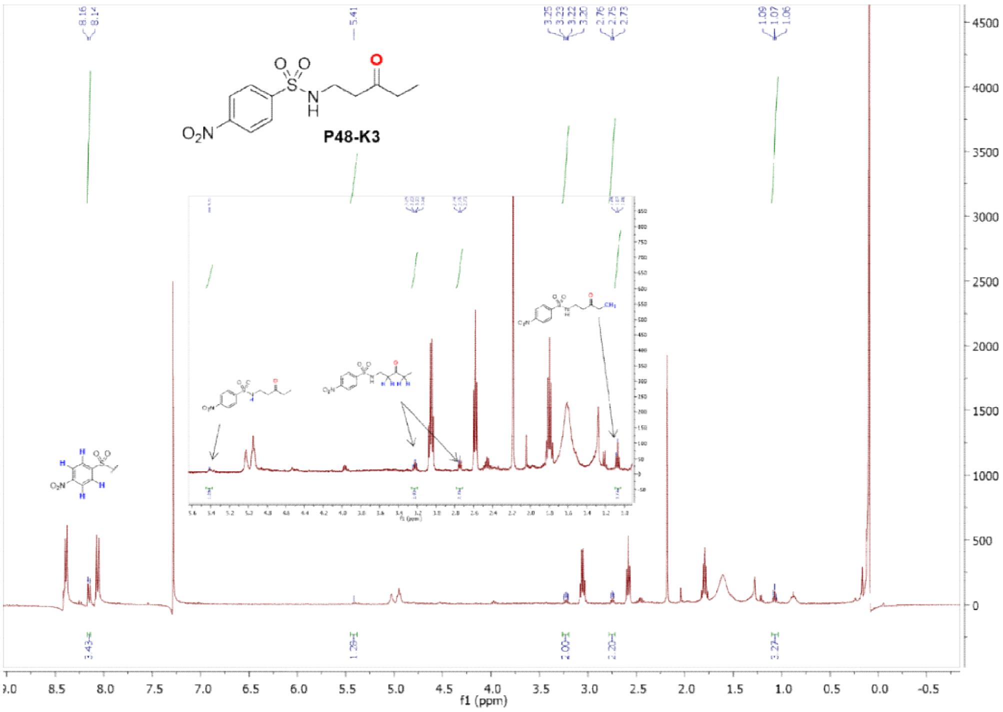

Figure S157. Crude mixture ^{1}H-NMR spectrum (400 MHz, $CDCl_3$) of 4-nitro-*N*-pentylbenzenesulfonamide (**S48**) oxidation in NFTBA: Identification of **P48-K3**.

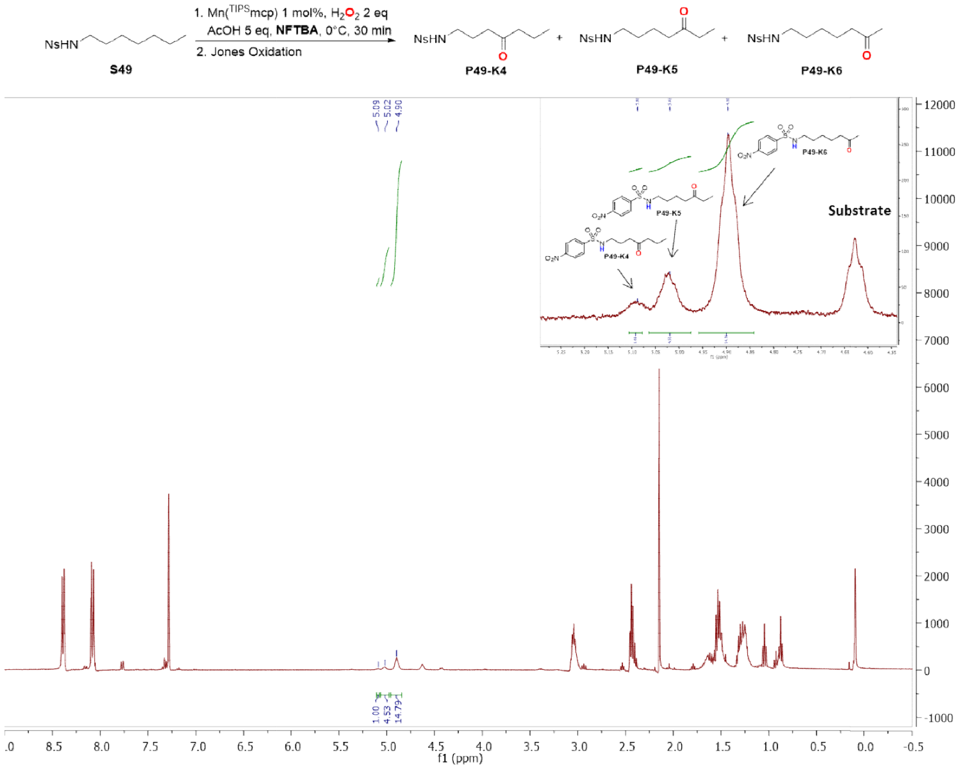

Figure S158. Crude mixture ^{1}H-NMR spectrum (400 MHz, $CDCl_3$) of 4-nitro-*N*-heptylbenzenesulfonamide (**S49**) oxidation in NFTBA.

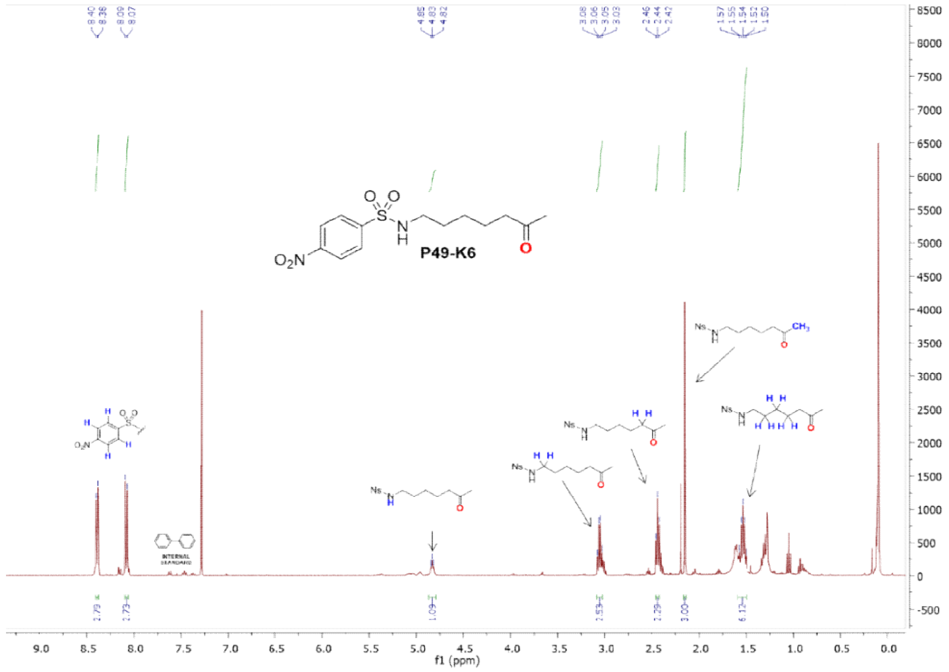

Figure S159. Crude mixture ^{1}H-NMR spectrum (400 MHz, $CDCl_3$) of 4-nitro-*N*-heptylbenzenesulfonamide (**S49**) oxidation in HFIP: Identification of **P49-K6**.

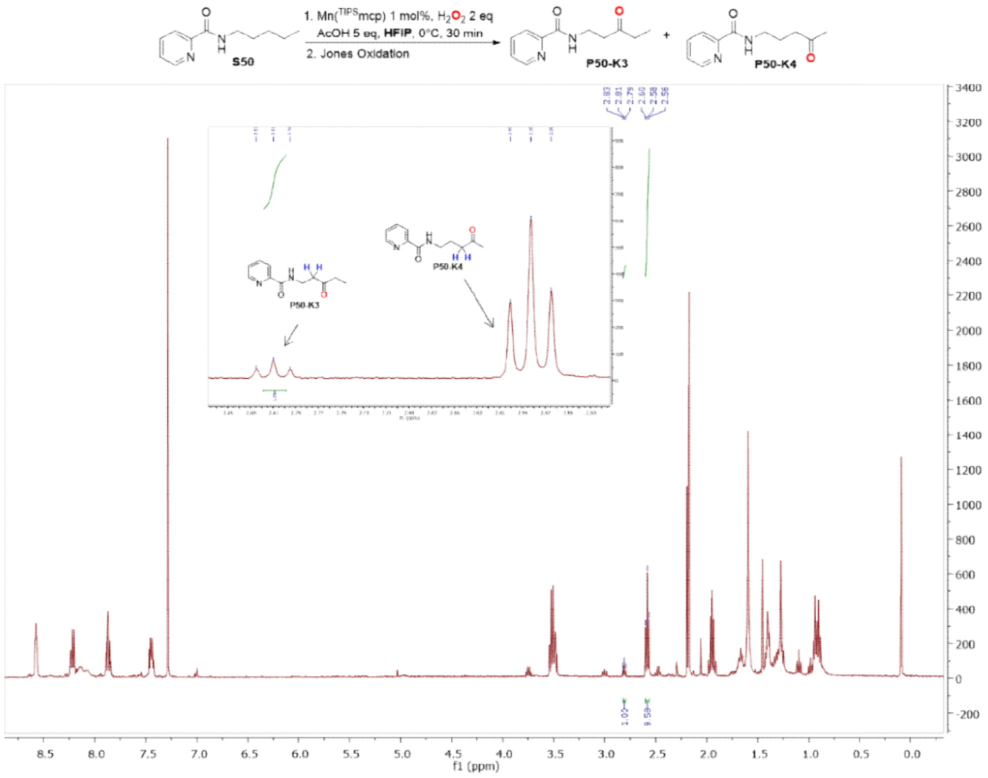

Figure S160. Crude mixture ^{1}H-NMR spectrum (400 MHz, $CDCl_3$) of *N*-pentyl 2-pyridinecarboxamide (**S50**) oxidation in HFIP.

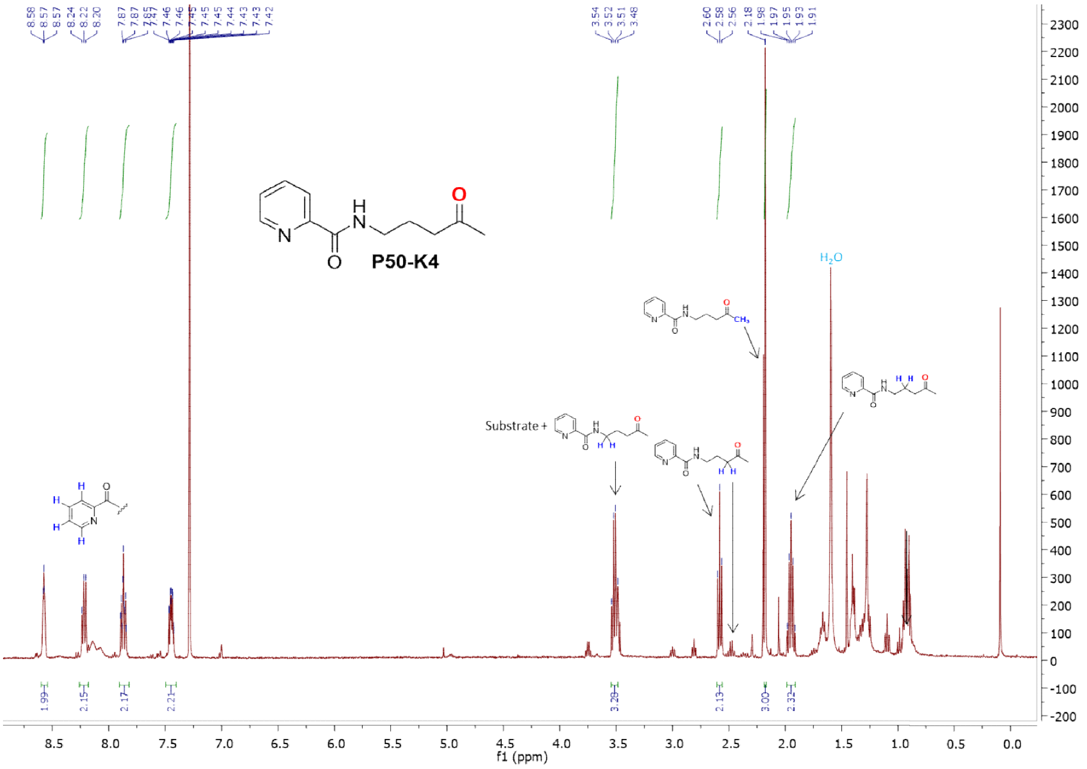

Figure S161. Crude mixture ^{1}H-NMR spectrum (400 MHz, $CDCl_3$) of *N*-pentyl 2-pyridinecarboxamide (**S50**) oxidation in HFIP: Identification of **P50-K4**.

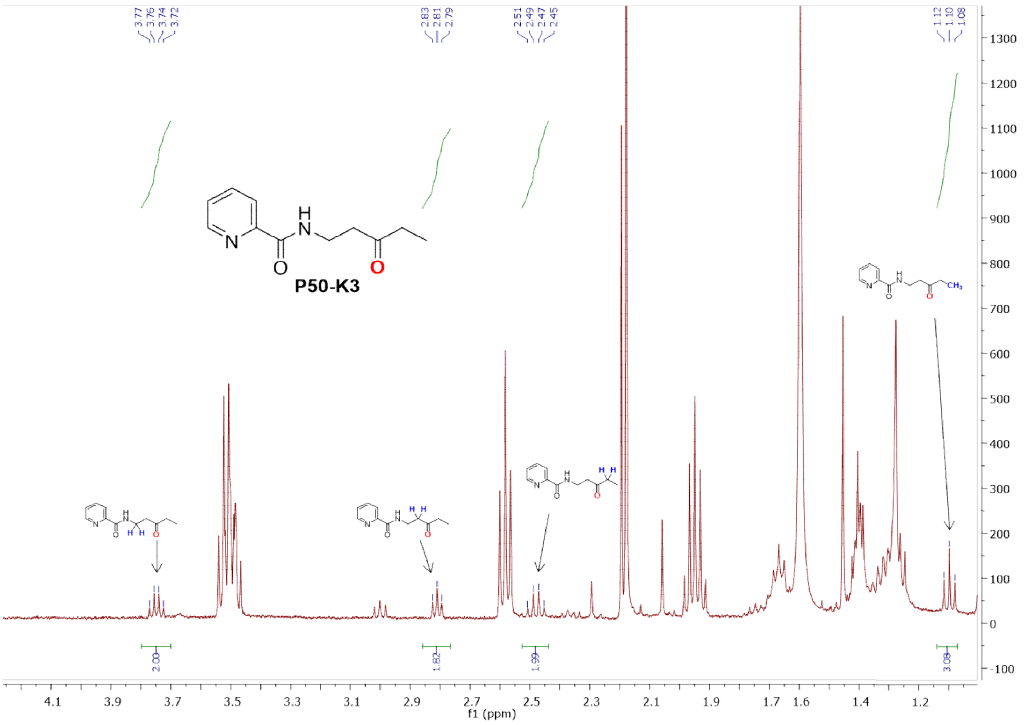

Figure S162. Crude mixture ^{1}H-NMR spectrum (400 MHz, $CDCl_3$) of *N*-pentyl 2-pyridinecarboxamide (**S50**) oxidation in HFIP: Identification of **P50-K3**.

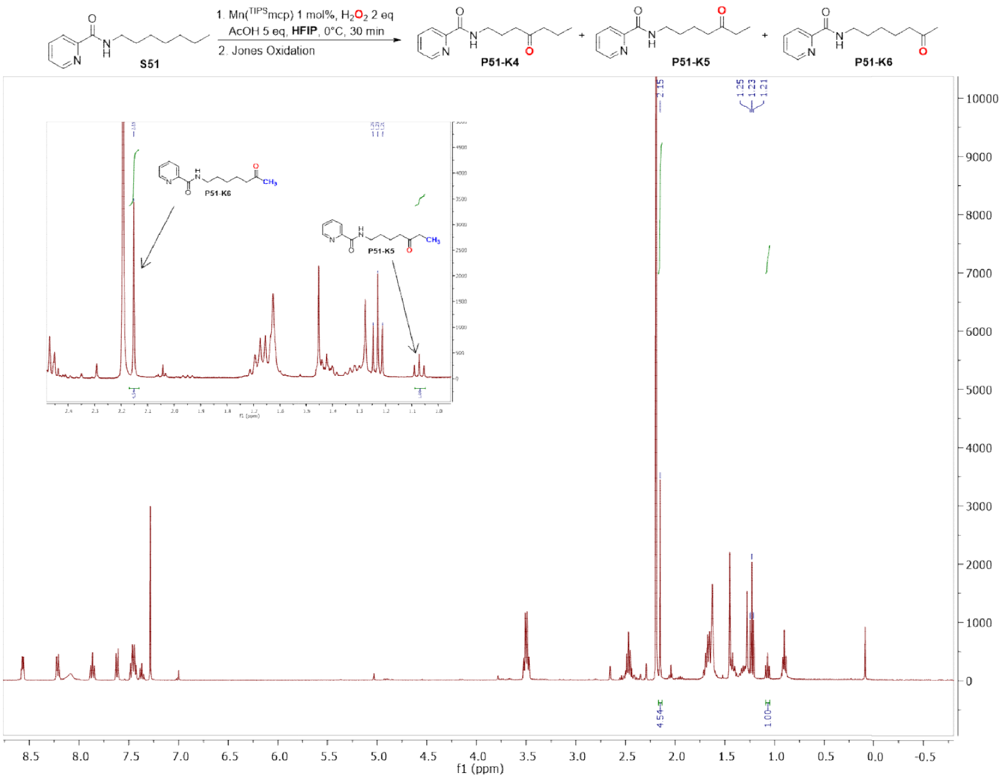

Figure S163. Crude mixture ^{1}H-NMR spectrum (400 MHz, $CDCl_3$) of *N*-heptyl 2-pyridinecarboxamide (**S51**) oxidation in HFIP.

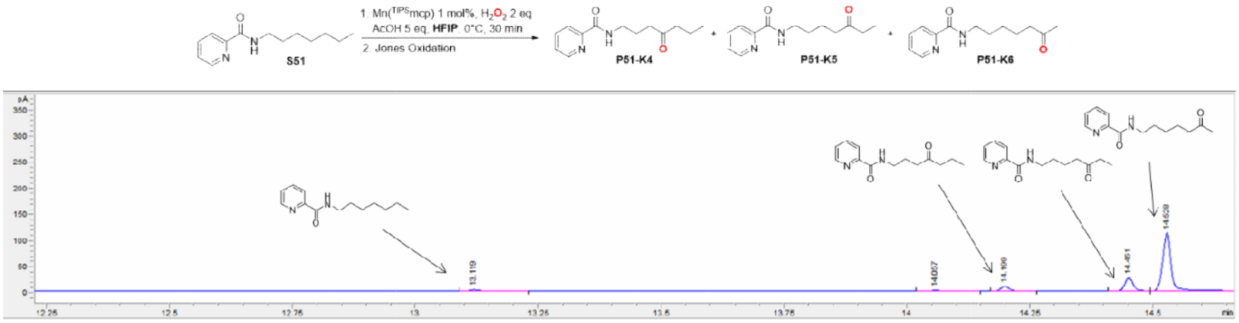

Figure S164. Crude mixture chromatogram of *N*-heptyl 2-pyridinecarboxamide (**S51**) oxidation in HFIP.

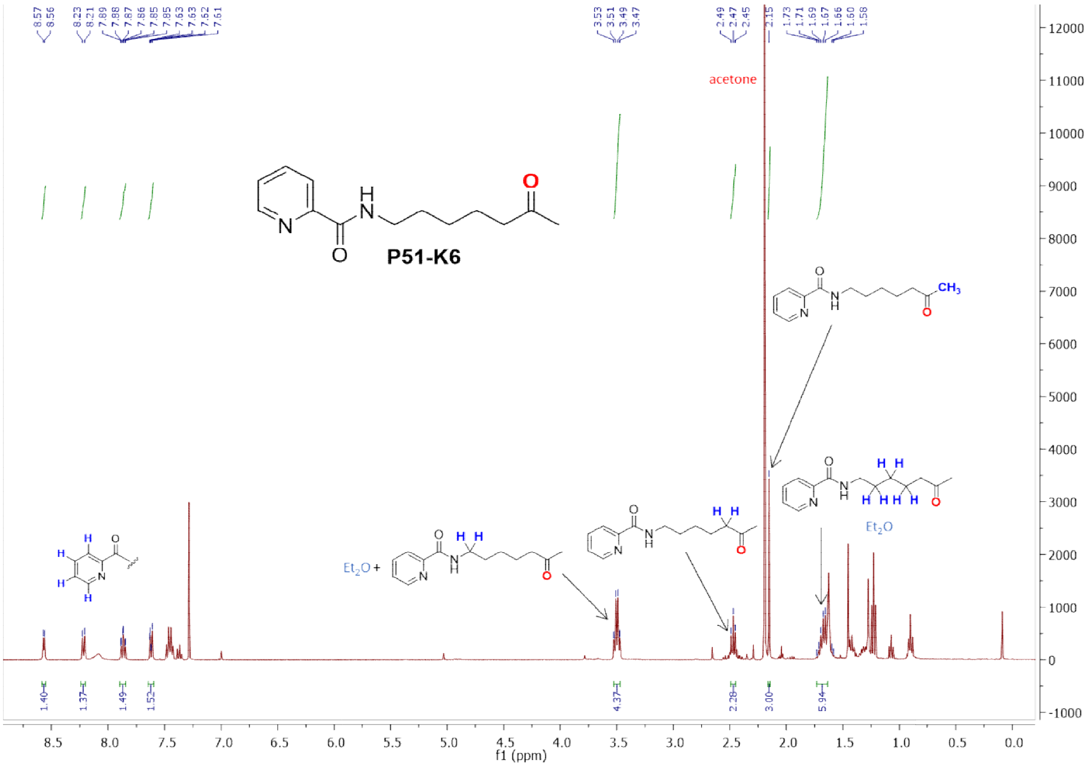

Figure S165. Crude mixture ^{1}H-NMR spectrum (400 MHz, $CDCl_3$) of *N*-heptyl 2-pyridinecarboxamide (**S51**) oxidation in HFIP: Identification of **P51-K6**.

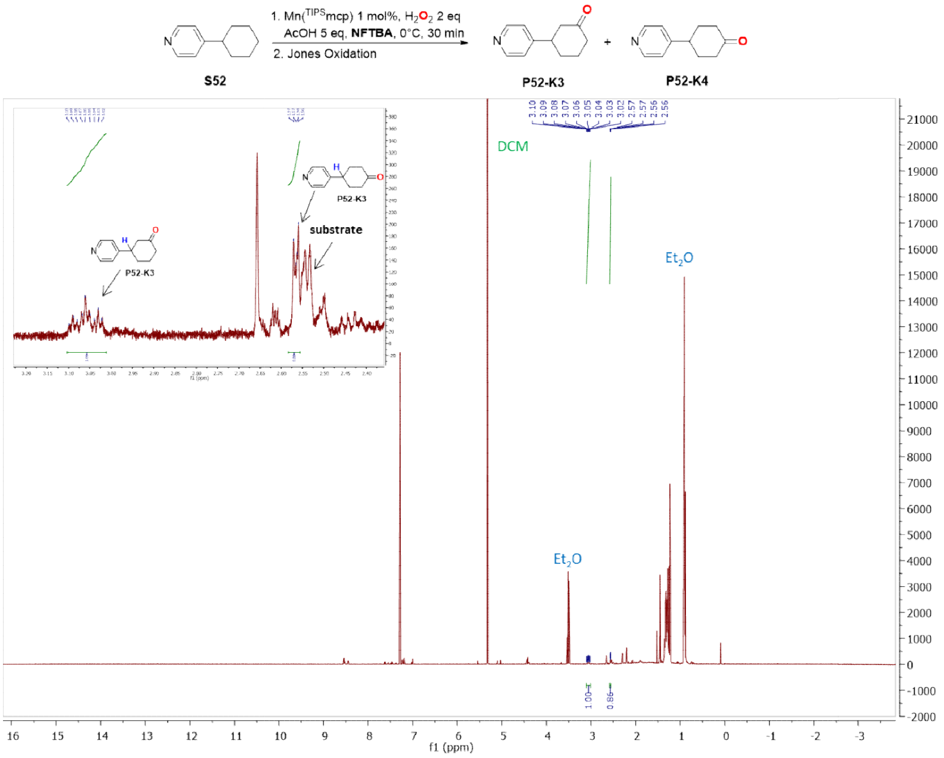

Figure S166. Crude mixture ^{1}H-NMR spectrum (400 MHz, $CDCl_3$) of 4-cyclohexylpyridine (**S52**) oxidation in NFTBA.

7. References

1. Fanourakis, A.; Williams, B. D.; Paterson, K. J.; Phipps, R. J. *J. Am. Chem. Soc*. **2021**, *143*, 10070-10076.

2. Lohr, T. R.; Li, Z.; Assary, R. S.; Curtiss, L. A.; Marks, T. J. *ACS catal*. **2015**, *5*, 3675-3679.

3. Basumatary, G.; Bez, G. *Tetrahedron Letters*, **2017**, *58*, 4312-4315.

4. Chen, M. S.; White, M. C. *Science*. **2010**, *327*, 566-571.

5. Hatano, M.;Yuji, T.; Yoshida, Y.; Toh, K; Yamashita, K.; Oguraa, Y.; Ishihara, K. *Green Chem*., **2018**, *20*, 1193-1198.

6. Coombera, C. E.; Portera, M. J.; Alieva, A. E.; Smith, P. D.; Sheppard, T. D. *Adv. Synth. Catal*. **2020**, *362*, 5105-5115.

7. Arzumanyan, A. V. *Tetrahedron Letters* **2017**, *8*, 4667-4671.

8. Wu, H.; Guo, W.; Stelck, D.; Li, Y.; Liu, C.; Zeng, Z. *Chem. Eur. J.* **2018**, *24*, 3444-3447.

9. Tanaka, S.; Nakashima, T.; Satou, N.; Oono, H.; Kon, Y.; Tamura, M.; Sato, K. *Tetrahedron Letters* **2019**, *60*, 2009-2013.

10. Griffin, J. D.; Vogt, D. B.; Du Bois, J.; Sigman, M. S. *ACS Catal*. **2021**, *11*, 10479-10486.

11. Hermange, P.; Lindhardt, A.; Taaning, R. H.; Bjerglund, K.; Lupp, D.; Skrydstrup, T. *J. Am. Chem. Soc*. **2011**, *133*, 6061-6071.

12. Minakawa, M.; Baek, H.; Yamada, Y. M. A.; Han, J. W.; Uozumi, Y. *Org. Lett*. **2013**, *15*, 5798-5801.

13. Lu, P.; Hou, T.; Gu, X.; Li, P. *Org. Lett*. **2015**, *17*, 1954-1957.

14. Chevella, D.; Thota, C.; Majumder, S. *Org. Biomol. Chem*. **2023**, *21*, 3837-3843.

15. Lux, M. C.; Jurczyk, J.; Lam, Y.; Song, Z. J.; Ma, C.; Roque, J. B.; Ham, J. S.; Sciammetta, N.; Adpressa, D.; Sarpong, R.; Yeung, C. S. *Org. Lett*. **2020**, *22*, 6578-6583.

16. Lin, Z.; Huang, L.; Yuan, G. *Chem. Commun*. **2021**, *57*, 3579-3582.

17. Zhu, M.; Fujita, K. I.; Yamaguchi, R. *Org. Lett*. **2010**, *12*, 1336-1339.

18. Bhuiyan, M. D. H.; Mahon, A. B.; Jensen, P.; Clegg, J. K.; Try, A. C. *Eur. J. Org. Chem*. **2009**, 687-698.

19. Palakurthy, N. B.; Mandal, B. *Tetrahedron Letters*. **2011**, *52*, 7132-7134.

20. Chang, D.; Zhao, R.; Wei, C.; Yao, Y.; Liu, Y.; Shi, L. *J. Org. Chem*. **2018**, *83*, 3305-3315.

21. Horn, A.; Dussault, P. A. *J. Org. Chem*. **2019**, *84*, 14611-14626.

22. Li, D.; Ma, T. K.; Scott, R. J.; Wilden, J. D. *Chem. Sci*. **2020**, *11*, 5333-5338.

23. Robin, A.; Köhler, V.; Jones, A.; Ali, A.; Kelly, P. P.; O'Reilly, E.; Turner, N. J.; Flitsch, S. L.; *Beilstein J. Org. Chem*. **2011**, *7*, 1494-1498.

24. Hu, Q. P.; Cheng, J.; Wang, Y.; Shi, J.; Wang, B. Q.; Hu, P.; Zhao, K. Q.; Pan, F. *Org. Lett*. **2021**, *23*, 4457-4462.

25. Nanjo, T.; de Lucca, E. C.; White, M. C. *J. Am. Chem. Soc*. **2017**, *139*, 14586-14591.

26. Zhou, F.; Ding, K.; Cai, Q. *Chem. Eur. J.* **2011**, *17*, 12268-12271.

27. Zhao, F.; Ai, H. J.; Wu, X. F. *Angew. Chem., Int. Ed*. **2022**, *61*, No. e202207970.

28. Majerski, K. M.; Margeta, R.; Veljković, J. *Synlett.* **2005**, *13*, 2089-2091.

29. Ratani, T. S.; Bachman, S.; Fu, G. C.; Peters, J. C. *J. Am. Chem. Soc*. **2015**, *137*, 13902-13907.

30. Milan, M.; Bietti, M.; Costas, M. *ACS Cent. Sci*. **2017**, *3*, 196-204.

31. Saito, M.; Kawamata, Y.; Meanwell, M.; Navratil, R.; Chiodi, D.; Carlson, E.; Hu1, P.; Chen, L.; Udyavara, S.; Kingston, C.; Tanwar, M.; Tyagi, S.; McKillican, B. P.; Gichinga, M. G.; Schmidt, M. A.; Eastgate, M. D.; Lamberto, M.; He1, C.; Tang, T.; Malapit, C. A.; Sigman, M. S.; Minteer, S. D.; Neurock, M.; Baran, P. S. *J. Am. Chem. Soc*. **2021**, *143*, 7859-7867.

32. Rao, S. N.; Mohan, D. C.; Adimurthy, S. *Org. Lett*. **2013**, *15*, 1496-1499.

33. Ju, M.; Guan, W.; Schomaker, J. M.; Harper, K. C. *Org. Lett*. **2019**, *21*, 8893-8898.

34. Chen, Q.; Len,T.; Knochel, P. *Angew. Chem. Int. Ed*. **2014**, *53*, 1-6.

35. Cussó, O.; Garcia-Bosch, I.; Font, D.; Ribas, X.; Lloret-Fillol, J.; Costas, M. *Org. Lett*. **2013**, *15*, 6158-6161.

36. Eisenbraun, E.J. *Org. Synth*. **1973**, *5*, 310-312.

37. Molander, G. A.; Alija, C. A. *J. Org. Chem*. **1998**, *63*, 4366-4373.

38. Herasymchuk, M.: Melnykov, P.K.; Yarmoliuk, D. V.; Serhiichuk, Y. D.; Kuchkovska, Y.O.; Holovach, S.; Volochnyuk, D.M.; Ryabukhin, S.V.; Grygorenko, O.O. *Eur. J. Org. Chem*. **2021**, *17*, 1-10.

39. Coxon, J. M.; Hartshorn, M. P.; Swallow, W. H. *J. Org. Chem*. **1974**, 39, 1143.

40. Pieter J. G.; Daniel B. A.; Floris P. J. T. R. *J. Org. Chem*. **2017**, *82*, 6671-6679.

41. Takahiro, N.; Tomoaki, O.; Kouichi, O.; Sakae U. *J. Org. Chem.* **1999**, *64*, 6750-6755.

42. Ken-ichi, I.; Takamitsu, U.; Kenji, H.; Sakamaki, M.; Takashi, S.; Akira, H. *Appl. Organometal. Chem*. **2007**, *21*, 1029-1032.
